# Development and Implementation of Video-Recorded Simulation Scenarios to Facilitate Case-Based Learning Discussions for Medical Students' Virtual Anesthesiology Clerkship

**DOI:** 10.15766/mep_2374-8265.11306

**Published:** 2023-04-04

**Authors:** Wendy Nguyen, Ilana Fromer, Mojca Remskar, Elena Zupfer

**Affiliations:** 1 Assistant Professor, Department of Anesthesiology, University of Minnesota Medical School; 2 Professor, Department of Anesthesiology, University of Minnesota Medical School

**Keywords:** Anesthesiology, Case-Based Learning, Online/Distance Learning, Simulation, Virtual Learning

## Abstract

**Introduction:**

The COVID-19 pandemic presented unique challenges to medical student education. Medical student activities involving direct patient contact were limited, challenging anesthesiology programs to develop innovative means of presenting a clinical experience to trainees. In response, the Department of Anesthesiology at the University of Minnesota Medical School quickly transitioned its introductory anesthesiology clerkship to be entirely virtual. We designed the resulting curriculum to provide medical students with the most experiential learning experience possible.

**Methods:**

We created and conducted a virtual curriculum for medical students that incorporated video-recorded simulation-based scenarios to facilitate case-based learning discussions (CBLDs). At the end of their 2-week rotation, students completed a postclerkship survey with Likert-scale questions and an open-ended question intended to elicit feedback and evaluate the efficacy of the virtual curriculum.

**Results:**

Twenty-eight medical students finished the 2-week virtual anesthesiology clerkship over eight blocks, with all 28 students completing the postclerkship survey. Survey responses demonstrated that the virtual clerkship met or exceeded expectations in all areas. A majority of students (74%, 14 of the 19 who answered the associated question) felt that the faculty-led CBLD exercises were informative. All 28 students agreed or strongly agreed that the virtual assignments were valuable and facilitated learning.

**Discussion:**

We successfully implemented a virtual anesthesiology clerkship curriculum in response to constraints presented by the COVID-19 pandemic. The virtual format provides trainees with a simulated clinical experience that can be utilized not only during future pandemics but also in modern training curricula.

## Educational Objectives

By the end of this activity, learners will be able to:

1. Utilize information from learning materials posted online to facilitate participation in case-based learning discussions (CBLDs).
2. Describe universal features of resuscitation of a patient in the perioperative setting.
3. Generate a differential diagnosis when presented with a scenario in a video-recorded simulation.
4. Actively participate virtually in PowerPoint-based CBLDs and video-recorded simulation-based CBLDs.
5. Develop skills in prioritizing treatment based on the scenario presented in a video-recorded simulation.
6. Describe the properties of intravenous and inhalation anesthetics along with their effects on different organ systems.
7. Identify predictors of difficult intubation and mask ventilation.
8. Apply the components of the American Society of Anesthesiologists’ Difficult Airway algorithm during management of a difficult airway.
9. Formulate an anesthetic plan based on a preanesthesia history and physical examination.

## Introduction

Clinical experience in medical student education is the cornerstone of transitioning from a learner/student to a resident trainee. The COVID-19 pandemic, with its associated personal protective equipment shortages and need to maintain social distancing, disrupted the existing model of clinical training.^1^ Additionally, the delay of surgical procedures to mitigate supply shortages and the spread of the COVID-19 virus further interrupted opportunities for clinical experience.^2^ On March 17, 2020, the AAMC provided guidance to medical schools recommending the removal of trainees from clinical settings.^3^ Two critical components of anesthesia instruction were significantly impacted by the removal of medical students from the clinical environment: simulation and teaching technical skills. Some simulation centers closed in response to the pandemic, while others pivoted to allow for COVID-19 preparation.^4^ Though many advancements in undergraduate and graduate medical education emerged as a result of the COVID-19 pandemic, the need to develop new programs quickly created time constraints that did not allow for prospective research.^4^

The University of California, San Francisco Medical Center is one example of a medical education program that quickly adopted a virtual training experience during the pandemic. It modified its internal medicine clerkship to include virtual rounds during which students prerounded using the electronic health record and then attended rounds virtually. Subsequently, students presented their patients virtually to receive feedback on their presentations and learn clinically relevant topics.^5^ Similarly, other medical schools used real-time videoconferencing to simulate morning conferences, bedside teaching, and modified patient encounters.^6–8^

Some institutions converted their simulation to telesimulation in which medical students or trainees participated virtually and simulation was carried out live by faculty or simulation staff.^9–11^ However, this modality still required simulation faculty or staff to be in proximity to one another, potentially risking COVID-19 infection.

Hanel and colleagues developed a simulation video in their emergency department in situ using real staff, including nurses, respiratory therapists, and physicians, intubating a COVID-infected patient with respiratory failure.^12^ The video was unrehearsed in order to elicit debrief discussions regarding process improvement. The recording was watched in person and was followed by a virtually facilitated debrief. Bilic, Nagji, and Hanel adapted the simulation video developed by Hanel and colleagues for their emergency medicine clerkship at McMaster University in lieu of in-person simulation experiences.^13^ In small virtual groups, a resident or faculty member showed medical students the 9-minute video with six freeze-frames containing discussion points that aligned with the video content. Although the survey response rate was not robust, medical students reported an increased level of comfort with the steps of a protected intubation.^13^

In adherence with AAMC recommendations, the University of Minnesota (UMN) Medical School halted all clinical clerkships mid-March 2020. To continue our elective introductory anesthesiology clerkship virtually, we used Kern's six steps^14^ to reengineer the clerkship, including the development of video-recorded simulations to use in case-based learning discussions (CBLDs) to facilitate learning of key clerkship objectives. While Bilic, Nagji, and Hanel incorporated a simulation video in their curriculum to promote instruction on a procedure commonly performed by anesthesiologists,^13^ we are not aware of any curricula or publications describing the development of anesthesiology simulation videos to facilitate CBLDs.

## Methods

From April to August 2020, eight blocks of medical students elected to participate in the 2-week virtual anesthesiology clerkship. During this time, they learned from a combination of didactics, readings, and CBLDs. We developed two types of CBLDs, both of which were delivered to medical students via Zoom meetings: video-recorded simulation-based CBLDs and PowerPoint-based CBLDs. This publication focuses on the development of video-recorded simulation-based CBLDs, a novel means of facilitating CBLDs through the creation of simulation videos using Kern's six-step approach to curriculum development for medical education.^14^

Regarding the development of PowerPoint-based CBLDs, the authors and other members of the medical student education committee created clinical scenarios to help illustrate the concepts covered in preexisting PowerPoint lectures. The scenarios attempted to carry medical students through the natural progression of an anesthetic case or preoperative evaluation. Members of the medical student education committee developed the original PowerPoints for the clerkship. We inserted segments of the clinical scenario and created questions related to the subsequent PowerPoint content (Appendices A–C). The PowerPoint-based CBLDs were delivered virtually by an anesthesiologist faculty member. Six core anesthesiology faculty members taught the virtual clerkship. Sessions lasted approximately 60–75 minutes.

### Virtual Clerkship Structure

Prior to the start of the clerkship, students received access to the Canvas Learning Management System (Instructure), an online learning platform, where the syllabus and learning materials were posted. Learning materials included three recorded PowerPoint lectures covering preoperative evaluation), inhaled and intravenous anesthetics, and airway management. Reading materials consisted of the corresponding chapters from Miller's *Basics of Anesthesia.*^15^ We encouraged students to go through these materials in advance of the faculty-delivered CBLDs. A total of six CBLDs were delivered to students via Zoom meetings over the course of the 2-week clerkship. These consisted of three PowerPoint-based CBLDs covering preoperative evaluation (Appendix A), inhaled and intravenous anesthetics (Appendix B), and airway management (Appendix C) and three video-recorded simulation-based CBLDs covering diagnosis and management of anaphylaxis (Appendix D), malignant hyperthermia (Appendix E), and unanticipated difficult airway (Appendix F). As much as possible, a single faculty member was responsible for the same CBLD throughout to increase faculty familiarity with the material. As a result, the timing of CBLDs depended on faculty availability.

### Step 1: Problem Identification and General Needs Assessment

#### Prepandemic situation

We offered the 2-week introductory anesthesiology clerkships each year from September to June. Up to four medical students at a time spent the majority of their 2-week rotation in the clinical setting. On the first day of the clerkship, a physician faculty member led the students through didactics consisting of lectures on core topics, an airway and peripheral intravenous access placement workshop, and a short, high-fidelity simulation experience involving induction of general anesthesia and intubation of a mannequin. We used the Canvas Learning Management System to post the syllabus and learning materials. Learning materials primarily consisted of book chapters from Miller's *Basics of Anesthesia.*^15^ Didactics were primarily lecture-based and used PowerPoint, covering topics including airway management, preoperative evaluation, and inhaled and intravenous anesthetics. The lectures were based on core topics found in Miller's *Basics of Anesthesia*.^15^

#### Ideal pandemic situation

We identified areas of the clerkship that would be most compromised if students learned from virtual didactics and readings only. The ideal approach mimicked the clinical environment in a virtual format as much as possible. Additionally, we used CBLDs in different ways to better engage the students.

### Step 2: Needs Assessment of Targeted Learners

Targeted learners were third- and fourth-year medical students at a tertiary major academic center. All had completed either their core surgery clerkship or core internal medicine clerkship and had been certified in American Heart Association Basic Life Support training twice. For feedback from these students, we reviewed past survey results from their clerkship regarding didactics and educational materials. These medical students appreciated the variety of learning materials available for self-study.

### Step 3: Goals and Measurable Objectives

We designed the simulation videos to promote higher-order learning that mimicked clinical decision-making in the operating room (OR) environment. These objectives are listed in the Educational Objectives above.

### Step 4: Educational Strategy

Case-based learning uses realistic cases and small-group discussion to encourage learners to apply their knowledge to clinical scenarios. It allows for more effective learning to take place as students achieve higher levels of critical thinking.^16^

#### Development of video-recorded simulation-based CBLDs

We used preexisting simulation scenarios developed by UMN physician anesthesiology faculty. Three physician anesthesiology faculty participated as actors in the simulation videos. The environment was a high-fidelity, simulated OR with the SimMan 3G (Laerdal) utilized as the simulated patient. One simulation technician controlled the SimMan 3G and vital signs using Laerdal software. Two cameras recorded the OR environment and the simulated patient vital signs. Three intraoperative scenarios were recorded: diagnosis and management of anaphylaxis (Appendix D), malignant hyperthermia (Appendix E), and unanticipated difficult airway (Appendix F). We watched these unedited videos and created a map of scenario events by writing out what was happening in the video and noting the corresponding time stamps. Based on our mapping of the videos, we chose eight to 12 key times representing decision-making branch points or opportunities to formulate differential diagnoses. Freeze slides were inserted in the videos at these key time stamps, along with content intended to help guide the medical students through the management of the patient in the simulation videos. Lastly, we developed video and debriefing guides to assist facilitators (Appendices G–I). Video specialists from UMN Academic Technology Support Services then edited the videos based on our mapping. The edited videos were approximately 7 minutes long. We used them with permission.

Medical students prepared for simulation-based CBLDs primarily by completing assigned *Basics of Anesthesia*^15^ readings and reviewing recorded PowerPoint lectures. Anesthesiology physician faculty facilitators met with the medical students virtually via Zoom. Two to six students participated at a time. During a short debrief (Appendices G–I), the facilitator discussed limitations of simulation including the inability to faithfully replicate the OR environment and workflow. Videos were played by the facilitators, who stopped at the freeze slides to initiate discussion among the medical students in order to answer questions posed. At the end of the videos, facilitators guided medical students through a discussion of takeaway points and debriefing questions (Appendices G–I). Simulation video viewing and intermittent discussions lasted about 20–30 minutes. The debriefing lasted about 10–15 minutes.

### Steps 5 and 6: Evaluation and Feedback

UMN Medical School administrators added questions specific to evaluating virtual curricula to the standard postclerkship survey. Administrators predetermined whether questions were assigned to a 3- or 5-point Likert-type scale. The surveys included one open-ended question for feedback (Appendix J). As part of the usual UMN clerkship evaluation process, we submitted clerkship-specific survey questions. To assess specific aspects of the new virtual curriculum and identify areas for improvement, we added three questions (Appendix K) to the survey.

UMN Medical School coordinators electronically distributed all survey questions and collected responses anonymously. Responses were distributed to clerkship coordinators periodically (i.e., not immediately after the end of the clerkship) in order to deliver the information in aggregate and to maintain anonymity. We coded the data from the open-ended questions by deducing their answers to a summative attribute and then categorizing the answers into groups for tabulation.^17^

## Results

From May 4 to August 21, 2020, 28 medical students rotated through the 2-week virtual introductory anesthesiology clerkship curriculum during one of eight blocks. Twenty-seven were fourth-year medical students, and one was a third-year medical student. All 28 completed the UMN Medical School postclerkship survey (100% response rate). A delay in the incorporation of our CBLD-specific survey resulted in 24 medical students receiving this survey. Nineteen medical students completed the CLBD-specific survey (79% response rate). Eleven medical students wrote comments. From the 11 comments, we coded 23 topics.

### Student Responses

#### General feedback

Medical students believed the virtual clerkship met or exceeded expectations in all areas: learning environment, organization, educational value, teaching, and feedback (Table 1). Four comments referenced the quality of organization. One medical student wrote that “the organization and expectations of students during this course was one of the best I've experienced thus far (in the virtual space).”

**Table 1.**
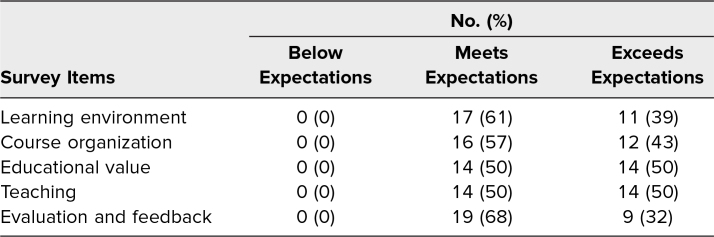
Medical Students’ General Perception of the Virtual Anesthesiology Clerkship (*N* = 28)

#### Curricula-specific feedback

All medical students agreed or strongly agreed that the objectives were clear and achieved, the assignments facilitated learning of the material, the resources were useful, and the clerkship was valuable (Table 2). Fourteen of the 19 UNM medical students answering the CBLD-specific survey questions (74%) agreed that the faculty-led CBLD sessions were informative (Table 3). Four comments indicated CBLD sessions were educationally stimulating, and another four cited appreciation of the variety of learning materials. One comment stated that the small size of the CBLD fostered participation. Three comments referred to excellent teaching from faculty. One medical student wrote that they “absolutely loved the interactive CBLD sessions. These were extremely helpful in learning the material, and were excellent ways to simulate clinical scenarios.” Eleven of the 19 students (58%) agreed that the utilization of video-recorded simulation-based CBLDs was an effective learning modality (Table 3). Seven (37%) agreed with the statement that video-recorded simulation-based CBLDs were more effective than other traditional learning modalities, while 10 (53%) were neutral (Table 3). Through informal feedback, faculty indicated that they found the videos easy to use and the freeze slides helped them standardize the discussions.

**Table 2.**
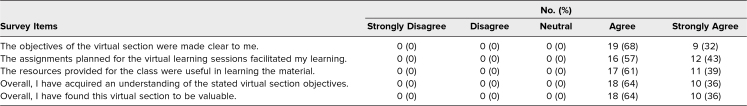
Medical Students’ Perception of Virtual-Specific Components of the Virtual Anesthesiology Clerkship (*N* = 28)

**Table 3.**
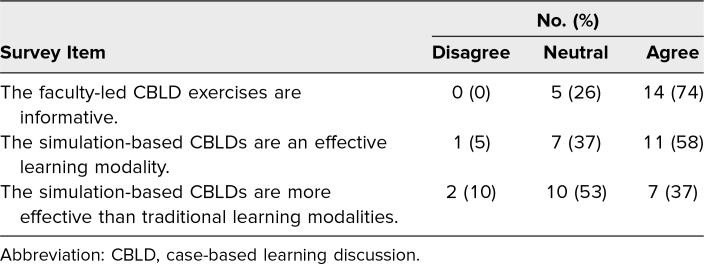
Medical Students’ Perception of the CBLDs Developed for the Virtual Anesthesiology Clerkship (*n* = 19)

#### Areas for curricular improvement

Two medical students indicated that the assessment tools could be better aligned with the virtual curriculum. One medical student wanted more didactics. There was one comment regarding logistical problems during the CBLD. One faculty member noted technical issues with the volume of the audio when playing the simulation over Zoom.

## Discussion

We filled a gap in clinical medical education at UMN Medical School caused by the COVID-19 pandemic by adapting our clerkship to work in a virtual format. More specifically, we acted out, recorded, and edited our simulation scenarios to facilitate CBLDs and give medical students insight into the role of an anesthesiologist in clinical scenarios.

The COVID-19 pandemic created an opportunity to revamp and reinvigorate our curriculum to meet the demands of going virtual. A small, dedicated team revamped the curriculum in a short period of time to minimize the impact on medical students. The response rate for the postclerkship survey was robust. On several measures, the medical students rated the virtual clerkship very favorably. The CBLDs, both PowerPoint- and simulation video-based, were highly rated by medical students (Table 3). Feedback focused on the CBLDs and other learning materials.

As the medical students did not spend any time in the OR environment and the simulation center was shut down, the video-recorded simulation-based CBLDs filled a void that other learning modalities could not. The CBLDs were successful because they allowed not only for active processing but also for visual and auditory information processing, thus optimizing the germane cognitive load associated with cognitive load theory.^18^ Our use of video segmentation via the introduction of freeze slides allowed for the management of intrinsic cognitive load.^18^ Additionally, the freeze slides introduced signaling, which enabled facilitators to highlight important concepts and manage intrinsic cognitive load.^18^

There were multiple limitations associated with an entirely virtual clerkship. Students did not have the opportunity to interact with multiple anesthesiologists, did not experience the day-to-day flow of ORs, and did not gain a sense of the level of interdisciplinary teamwork associated with anesthesiology and patient care. The virtual clerkship did not include direct patient care, which is a tenet of apprenticeship and cognitive theories of cognitive and workplace learning.^5^ Moreover, traditional anesthesiology clerkships provide a unique opportunity for medical students to practice procedural skills in a safe, controlled, clinical environment.^19,20^ Although we covered the process of an induction of general anesthesia with endotracheal intubation in one of the simulation videos, the acquisition of procedural skills requires hands-on learning.^21^ Also, the video-recorded simulation-based CBLDs did not approximate the flow of the OR as the scenarios were paused for intermittent discussion. Additionally, there were minor audio issues associated with facilitating the CBLDs and watching the videos. These audio issues could be ameliorated by ensuring the use of computers with high-quality audio. Although we did not experience any significant technological difficulties, a virtual clerkship could potentially be subject to lapses in Wi-Fi connection, lack of internet access, or various personal computer difficulties.

The simulated OR environment's fidelity to reality in our videos was affected by time constraints. The AAMC recommendation to halt clinical training in March 2020 prompted us to create, develop, and implement a virtual curriculum as quickly as possible. As a result, there were multiple departures from reality in the videos that may have been misleading for students. This is especially important as our videos may have represented the first exposure to the OR for some of the medical students. We attempted to address these shortcomings in the debriefing. Given more time, we would have edited the videos to recreate the OR environment more faithfully.

This was a single-institution experience with a small sample size. We have reported how the development and implementation of this virtual clerkship unfolded. Another limitation imposed by the pandemic was the inability to include a comparison group since learners were barred from participating in in-person simulation.^4^ Additionally, there were no in-person clerkship medical students who could serve as direct controls. Our CBLD-specific survey questions were not distributed to all the medical students, so the views on the curricular components we did receive may not have been representative of all those participating in the virtual clerkship. Further research with larger groups might further elucidate the value of this curriculum. Finally, with more time, we could have gathered evidence to support the validity of both the medical school's and our own virtual clerkship-specific survey questions.

In the fall of 2021, we transitioned back to in-person learning for our introductory clerkship. Students returned to the ORs and resumed in-person simulation sessions. While the OR environment affords students the best opportunity to observe the teamwork, professionalism, and communication necessary for safe patient care, there are aspects of virtual learning that we have found to be valuable. Given the favorable survey results from the virtual clerkship, we have incorporated the PowerPoint-based CBLD learning into our current clinical curriculum. We continue to meet virtually for the CBLD sessions given their convenience for faculty and medical students amid the COVID pandemic.

New pandemics, as well as the ongoing COVID pandemic, will likely warrant completing clerkships virtually in the future. Additionally, our simulation videos could help facilitate learning about anesthesiology in areas where simulation centers are not available. The virtual curriculum let us accommodate more medical students than we typically allow for at one time during the in-person clerkship. In the future, the virtual clerkship could be implemented on an even wider scale and include more medical students.

The COVID-19 pandemic challenged us to rapidly evolve our curriculum and the way we deliver it. Fortunately, technology gave us both the ability to record and edit simulation videos and a reliable virtual learning environment in which to utilize them. Our results demonstrate that we executed a compelling virtual anesthesiology clerkship. Overall, medical students rated the learning experience very highly. Lessons learned from this experience have improved our in-person curriculum.

## Appendices


Preoperative Evaluation - CBLD 1.pptxInhaled and Intravenous Anesthetics - CBLD 2.pptxAirway Management - CBLD 3.pptxScenario 1.mp4Scenario 2.mp4Scenario 3.mp4Scenario Debrief 1.docxScenario Debrief 2.docxScenario Debrief 3.docxClerkship Survey Questions.docxCBLD-Specific Survey Questions.docx
*All appendices are peer reviewed as integral parts of the Original Publication.*

## References

[1] Rose S. Medical student education in the time of COVID-19. JAMA. 2020;323(21):2131–2132. 10.1001/jama.2020.5227 32232420

[2] COVID-19: elective case triage guidelines for surgical care. American College of Surgeons. March 24, 2020. Accessed January 12, 2023. https://www.facs.org/for-medical-professionals/covid-19/clinical-guidance/elective-case/

[3] Whelan A, Prescott J, Young G, Catanese VM, McKinney R. *Guidance on Medical Students’ Participation in Direct In-person Patient Contact Activities.* Association of American Medical Colleges; 2020. Accessed January 12, 2023. https://www.aamc.org/system/files/2020-08/meded-August-14-Guidance-on-Medical-Students-on-Clinical-Rotations.pdf

[4] Martinelli SM, Chen F, Isaak RS, Huffmyer JL, Neves SE, Mitchell JD. Educating anesthesiologists during the coronavirus disease 2019 pandemic and beyond. Anesth Analg. 2021;132(3):585–593. 10.1213/ANE.0000000000005333 33201006

[5] Sukumar S, Zakaria A, Lai CJ, Sakumoto M, Khanna R, Choi N. Designing and implementing a novel virtual rounds curriculum for medical students’ internal medicine clerkship during the COVID-19 pandemic. MedEdPORTAL. 2021;17:11106. 10.15766/mep_2374-8265.11106 33768143 PMC7970635

[6] Murdock HM, Penner JC, Le S, Nematollahi S. Virtual Morning Report during COVID-19: a novel model for case-based teaching conferences. Med Educ. 2020;54(9):851–852. 10.1111/medu.14226 32403168 PMC7273056

[7] Hofmann H, Harding C, Youm J, Wiechmann W. Virtual bedside teaching rounds with patients with COVID-19. Med Educ. 2020;54(10):959–960. 10.1111/medu.14223 32403185 PMC7273015

[8] Tsang ACO, Lee PP, Chen JY, Leung GKK. From bedside to webside: a neurological clinical teaching experience. Med Educ. 2020;54(7):660. 10.1111/medu.14175 32285492 PMC7262309

[9] Ray JM, Wong AH, Yang TJ, et al. Virtual telesimulation for medical students during the COVID-19 pandemic. Acad Med. 2021;96(10):1431–1435. 10.1097/ACM.0000000000004129 33883398 PMC8475640

[10] Yang T, Buck S, Evans L, Auerbach M. A telesimulation elective to provide medical students with pediatric patient care experiences during the COVID pandemic. Pediatr Emerg Care. 2021;37(2):119–122. 10.1097/PEC.0000000000002311 33181792 PMC7850555

[11] Patel SM, Miller CR, Schiavi A, Toy S, Schwengel DA. The sim must go on: adapting resident education to the COVID-19 pandemic using telesimulation. Adv Simul (Lond). 2020;5:26. 10.1186/s41077-020-00146-w 32999738 PMC7522907

[12] Hanel E, Bilic M, Hassall K, et al. Virtual application of in situ simulation during a pandemic. CJEM. 2020;22(5):563–566. 10.1017/cem.2020.375 PMC7218188 32327002

[13] Bilic M, Nagji A, Hanel E. Video in situ simulation for medical student education during the COVID-19 pandemic. Can Med Educ J. 2021;12(4):141–142. 10.36834/cmej.71741 34567316 PMC8463235

[14] Thomas PA, Kern DE, Hughes MT, Chen BY, eds. Curriculum Development for Medical Education: A Six-Step Approach. 3rd ed. Johns Hopkins University Press; 2016.

[15] Pardo MC Jr, Miller RD. Basics of Anesthesia. 7th ed. Elsevier; 2018.

[16] Thistlethwaite JE, Davies D, Ekeocha S, et al. The effectiveness of case-based learning in health professional education. A BEME systematic review: BEME Guide no. 23. Med Teach. 2012;34(6):e421–e444. 10.3109/0142159X.2012.680939 22578051

[17] Merriam SB, Tisdell EJ. Qualitative Research: A Guide to Design and Implementation. 4th ed. Jossey-Bass; 2016.

[18] Young TP, Guptill M, Thomas T, Mellick L. Effective educational videos in emergency medicine. AEM Educ Train. 2018;2(S1):S17–S24. 10.1002/aet2.10210 30607375 PMC6304276

[19] Curry SE. Teaching medical students clinical anesthesia. Anesth Analg. 2018;126(5):1687–1694. 10.1213/ANE.0000000000002802 29401078

[20] Prys-Roberts C. Role of anaesthesiologists in undergraduate medical education. Curr Opin Anaesthesiol. 2000;13(6):653–657. 10.1097/00001503-200012000-00008 17016371

[21] Burgess A, van Diggele C, Roberts C, Mellis C. Tips for teaching procedural skills. BMC Med Educ. 2020;20(suppl 2):458. 10.1186/s12909-020-02284-1 33272273 PMC7712522

