# Supplementary material for: Development and Implementation of Video-Recorded Simulation Scenarios to Facilitate Case-Based Learning Discussions for Medical Students' Virtual Anesthesiology Clerkship
Source: MedEdPORTAL. 2023 Apr 4;19:11306. doi: 10.15766/mep_2374-8265.11306 (PMC10070881; doi:10.15766/mep_2374-8265.11306)
Supplement: Supplementary file 1 — Preoperative Evaluation - CBLD 1.pptxInhaled and Intravenous Anesthetics - CBLD 2.pptxAirway Management - CBLD 3.pptxScenario 1.mp4Scenario 2.mp4Scenario 3.mp4Scenario Debrief 1.docxScenario Debrief 2.docxScenario Debrief 3.docxClerkship Survey Questions.docxCBLD-Specific Survey Questions.docx [file mep_2374-8265.11306-s001.zip › A. Preoperative Evaluation - CBLD 1.pptx]

## Slide 1
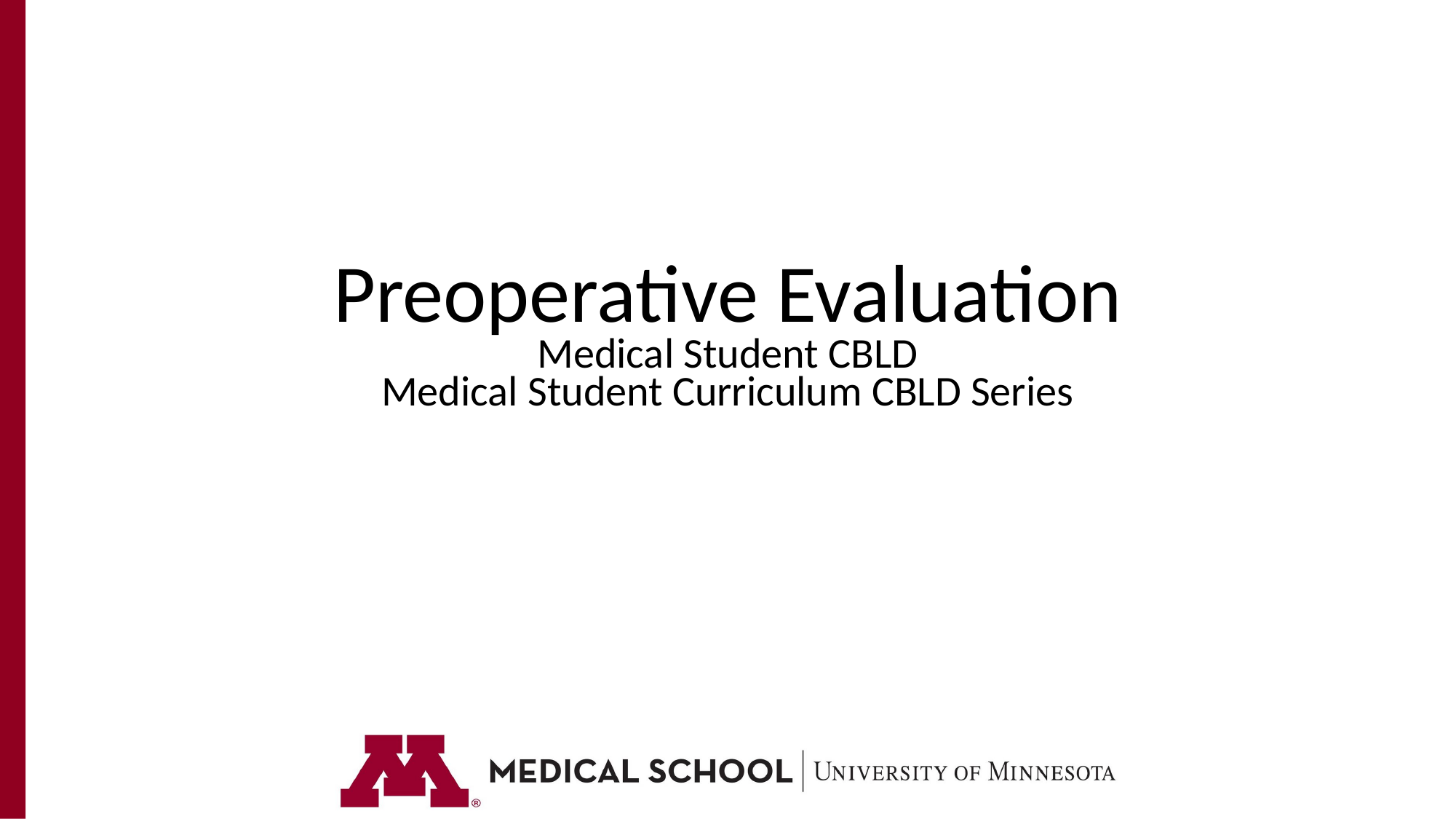

# Preoperative Evaluation Medical Student CBLD Medical Student Curriculum CBLD Series

## Slide 2
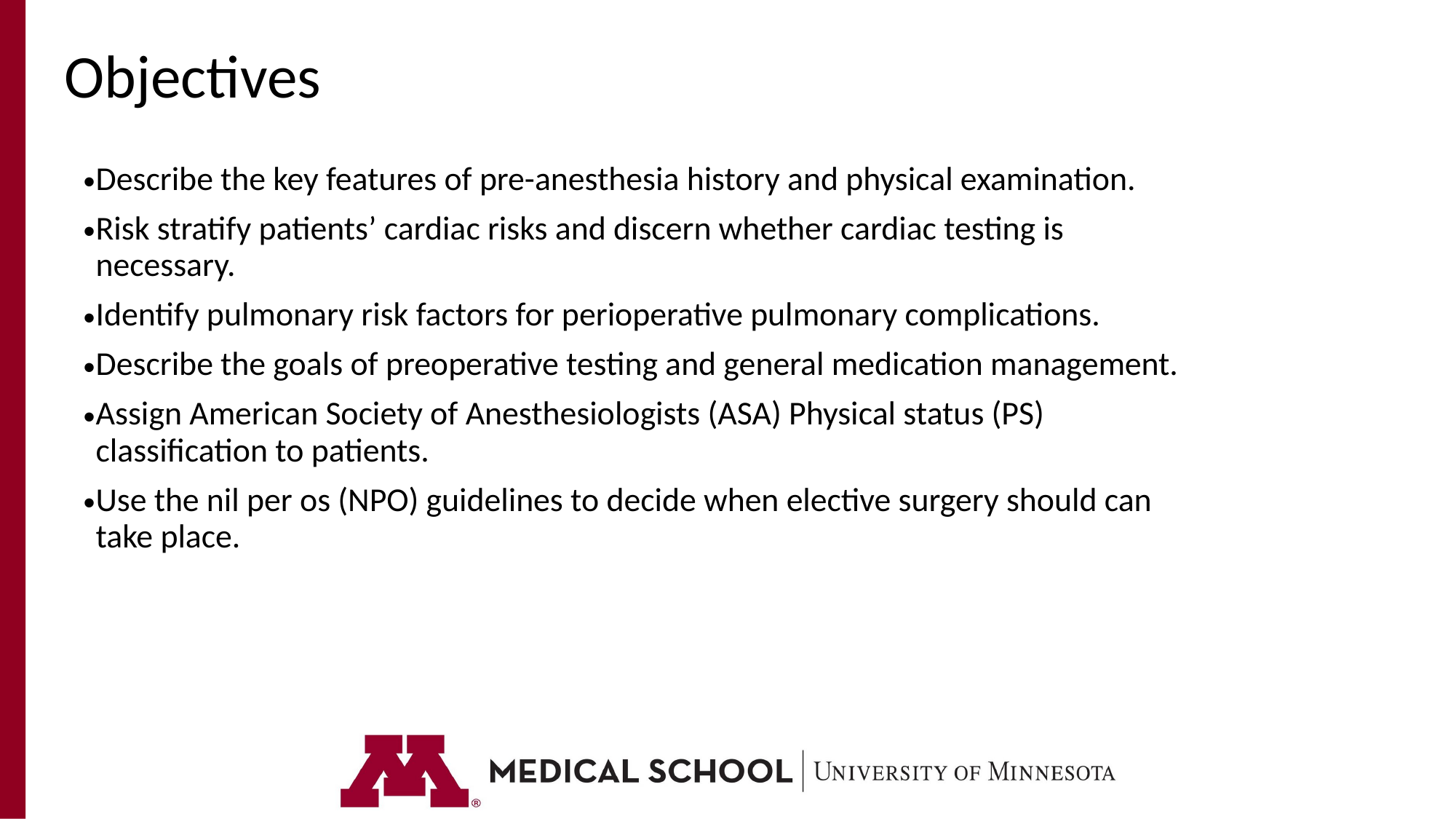

# Objectives
Describe the key features of pre-anesthesia history and physical examination.
Risk stratify patients’ cardiac risks and discern whether cardiac testing is necessary.
Identify pulmonary risk factors for perioperative pulmonary complications.
Describe the goals of preoperative testing and general medication management.
Assign American Society of Anesthesiologists (ASA) Physical status (PS) classification to patients.
Use the nil per os (NPO) guidelines to decide when elective surgery should can take place.

## Slide 3
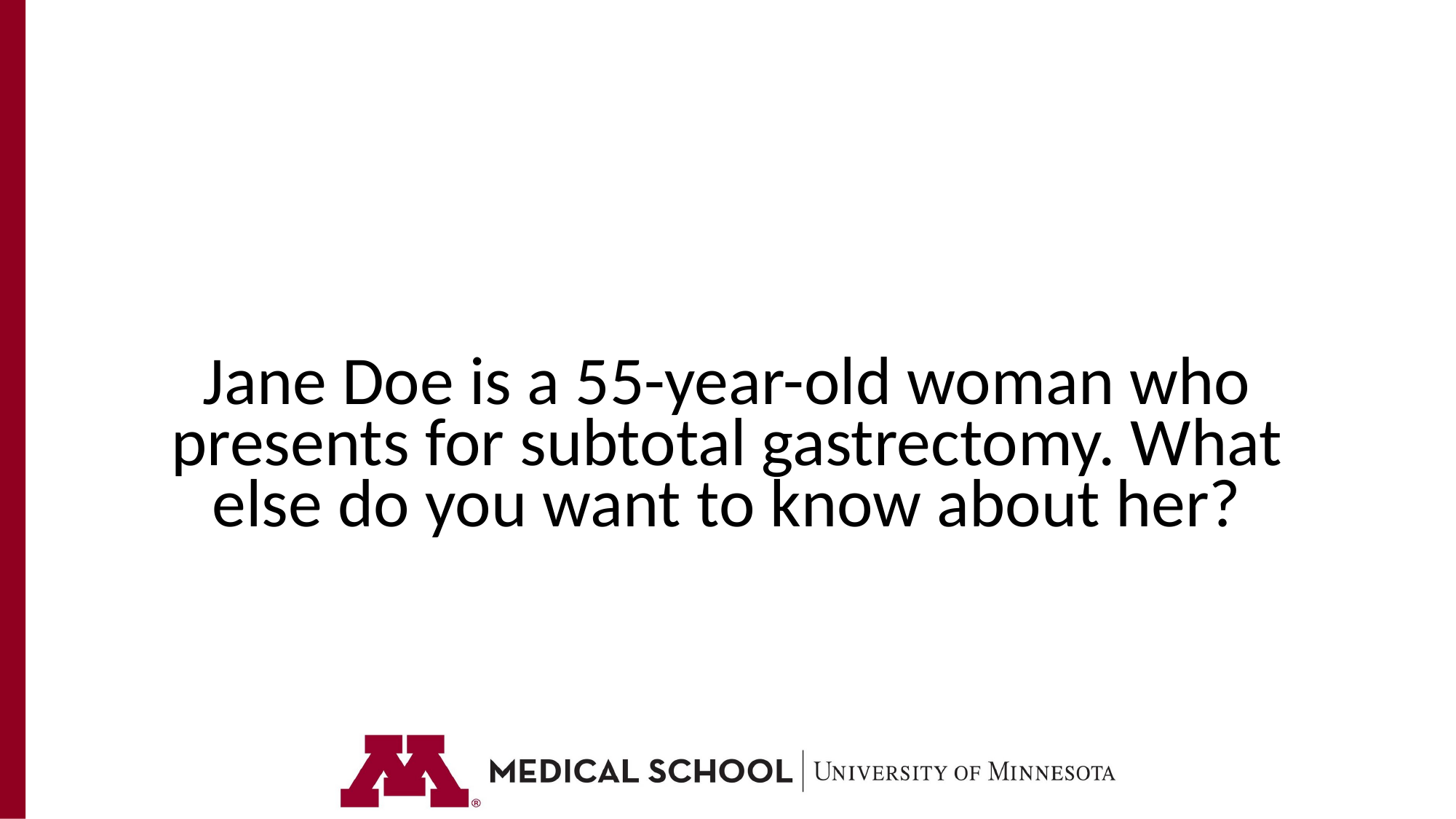

# Jane Doe is a 55-year-old woman who presents for subtotal gastrectomy. What else do you want to know about her?

## Slide 4
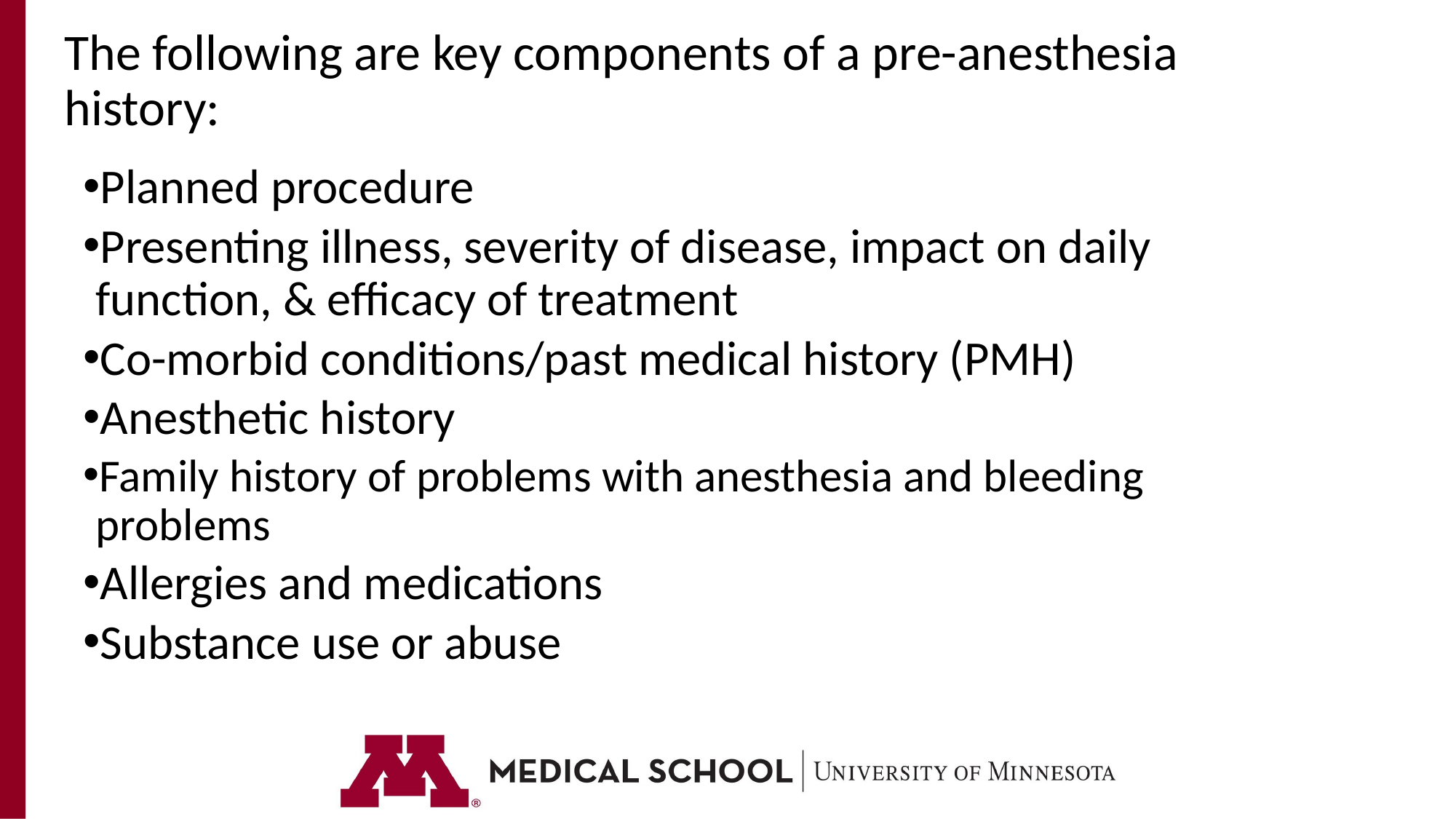

# The following are key components of a pre-anesthesia history:
Planned procedure
Presenting illness, severity of disease, impact on daily function, & efficacy of treatment
Co-morbid conditions/past medical history (PMH)
Anesthetic history
Family history of problems with anesthesia and bleeding problems
Allergies and medications
Substance use or abuse

## Slide 5
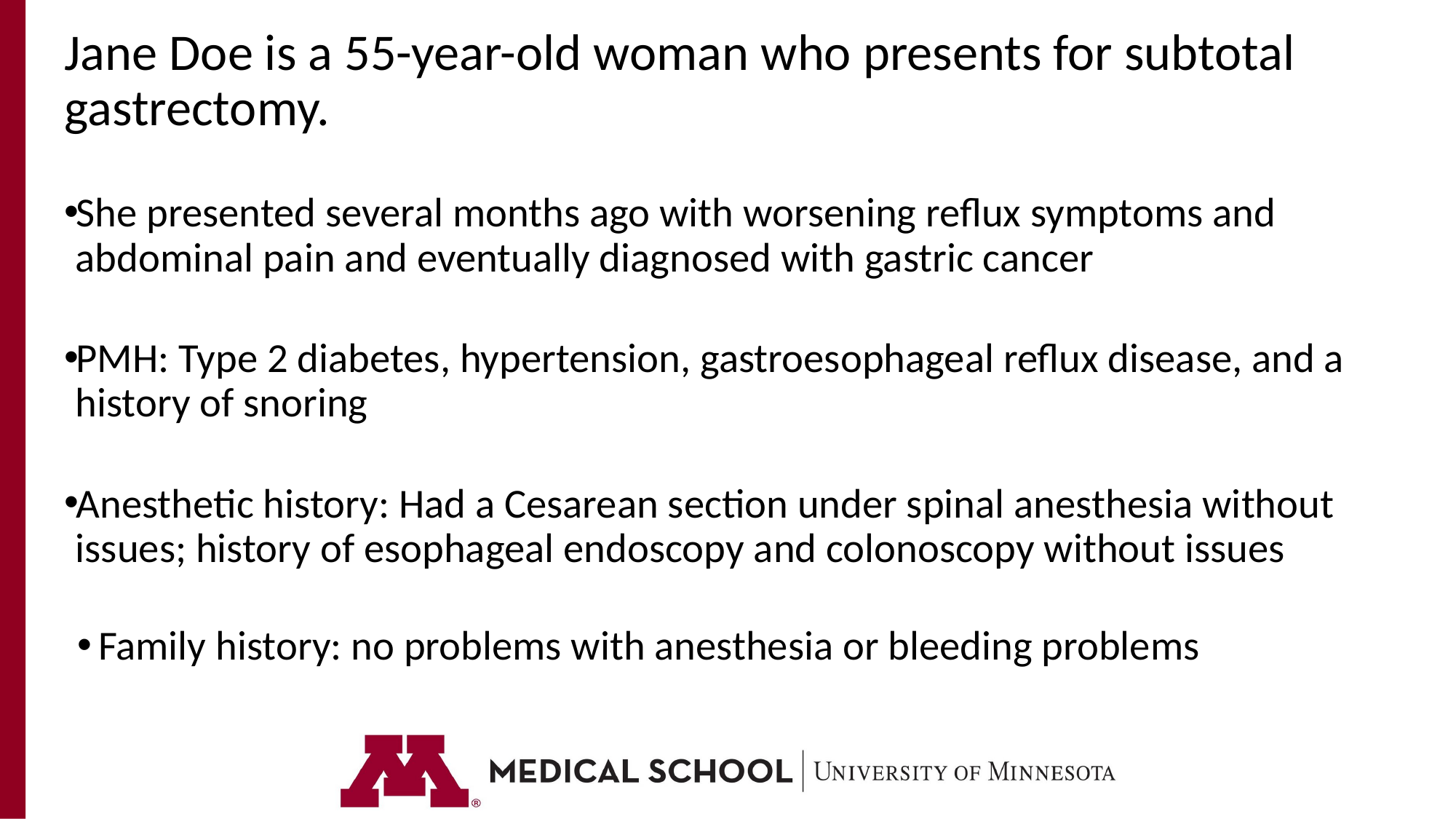

# Jane Doe is a 55-year-old woman who presents for subtotal gastrectomy.
She presented several months ago with worsening reflux symptoms and abdominal pain and eventually diagnosed with gastric cancer
PMH: Type 2 diabetes, hypertension, gastroesophageal reflux disease, and a history of snoring
Anesthetic history: Had a Cesarean section under spinal anesthesia without issues; history of esophageal endoscopy and colonoscopy without issues
Family history: no problems with anesthesia or bleeding problems

## Slide 6
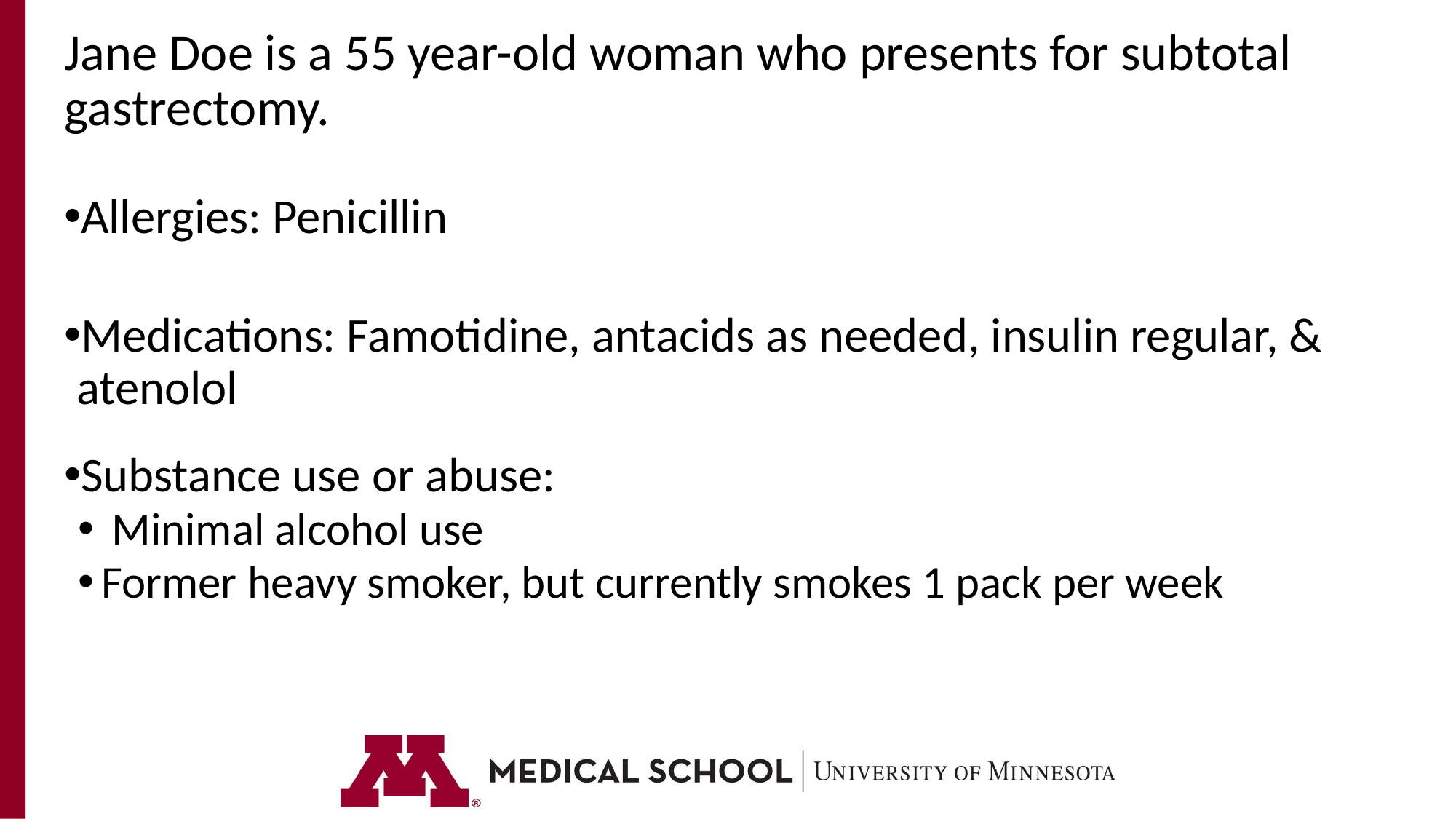

# Jane Doe is a 55 year-old woman who presents for subtotal gastrectomy.
Allergies: Penicillin
Medications: Famotidine, antacids as needed, insulin regular, & atenolol
Substance use or abuse:
 Minimal alcohol use
Former heavy smoker, but currently smokes 1 pack per week

## Slide 7
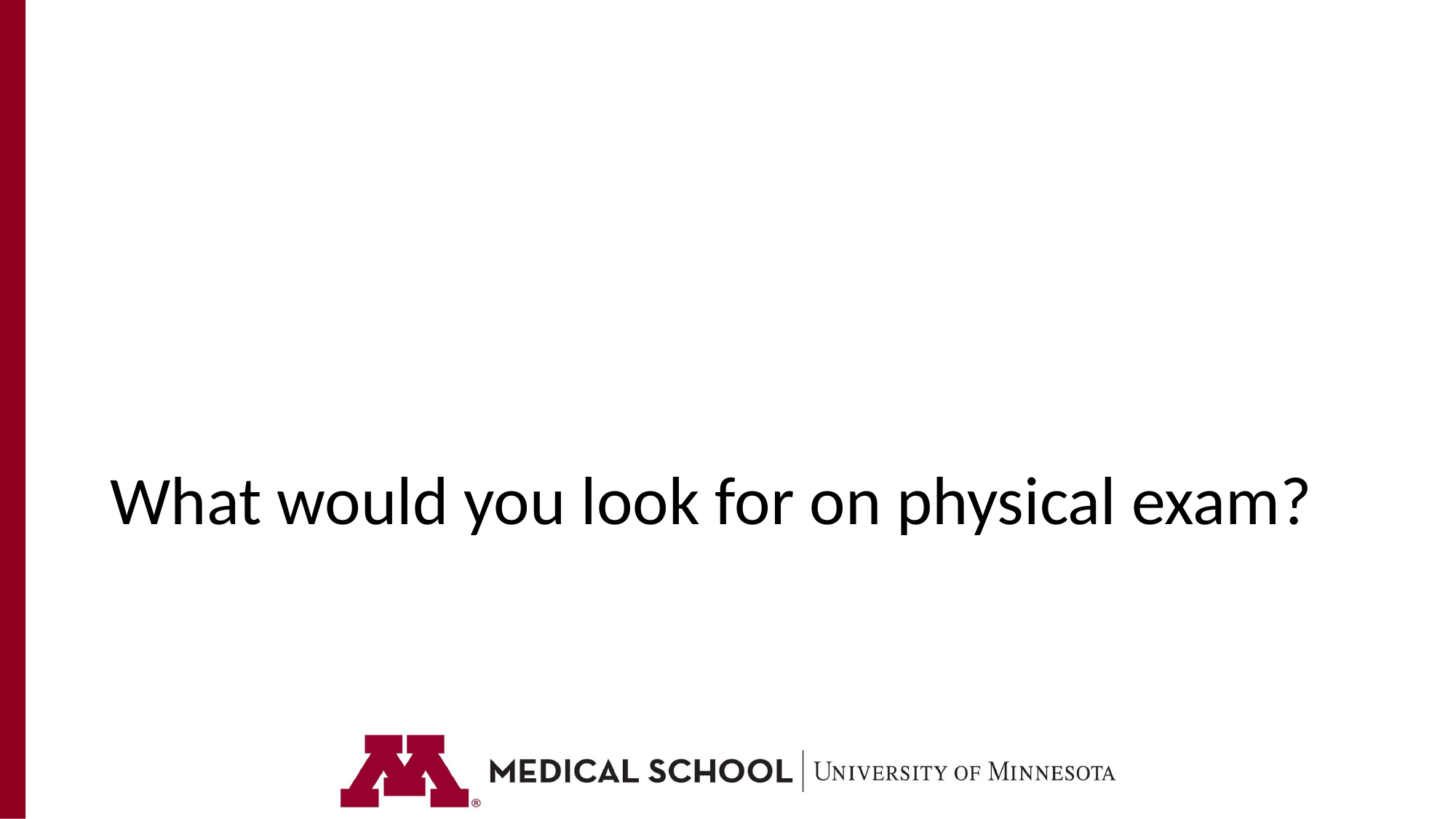

# What would you look for on physical exam?

## Slide 8
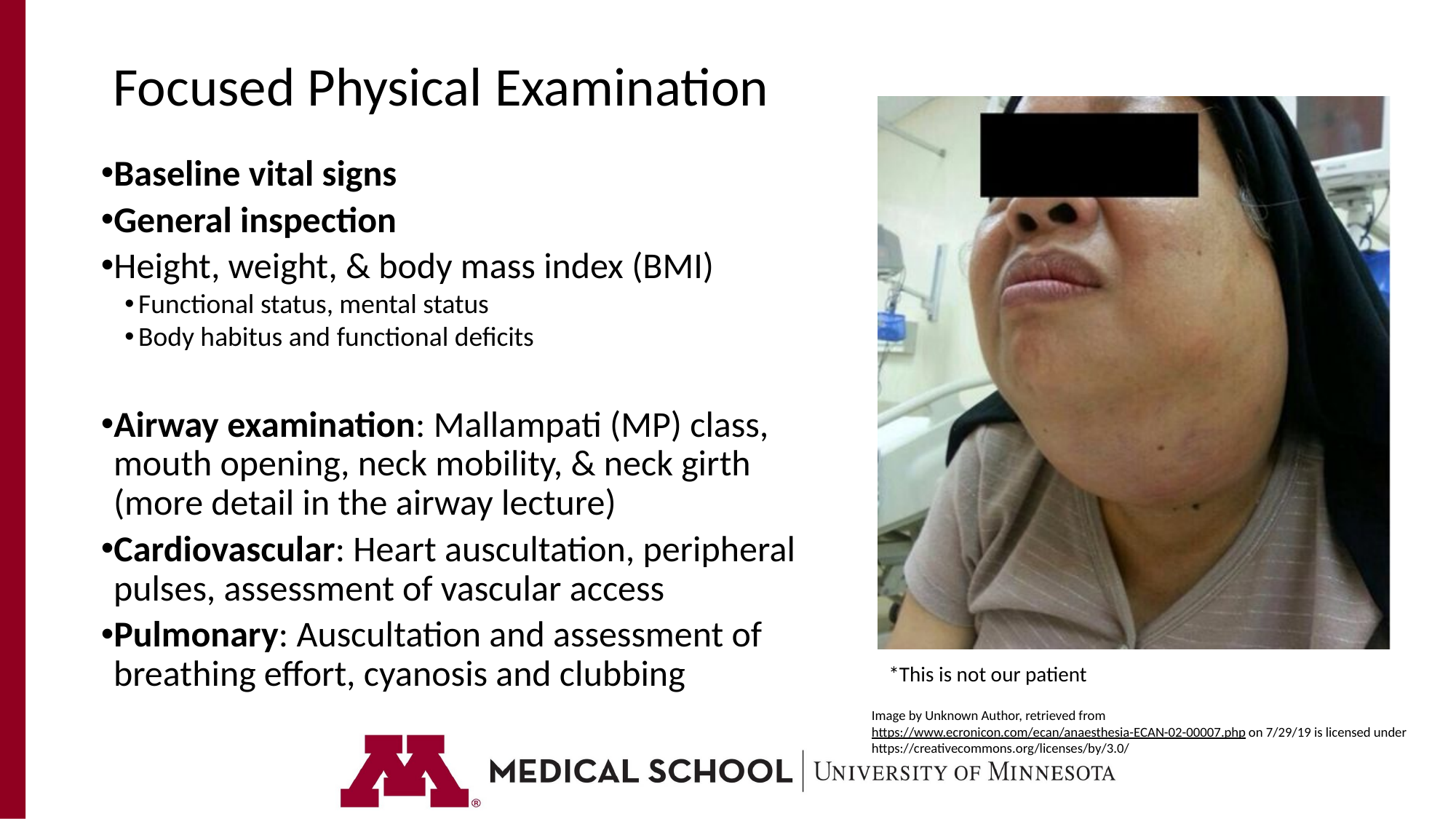

# Focused Physical Examination
Baseline vital signs
General inspection
Height, weight, & body mass index (BMI)
Functional status, mental status
Body habitus and functional deficits
Airway examination: Mallampati (MP) class, mouth opening, neck mobility, & neck girth (more detail in the airway lecture)
Cardiovascular: Heart auscultation, peripheral pulses, assessment of vascular access
Pulmonary: Auscultation and assessment of breathing effort, cyanosis and clubbing
*This is not our patient
Image by Unknown Author, retrieved from https://www.ecronicon.com/ecan/anaesthesia-ECAN-02-00007.php on 7/29/19 is licensed under https://creativecommons.org/licenses/by/3.0/

## Slide 9
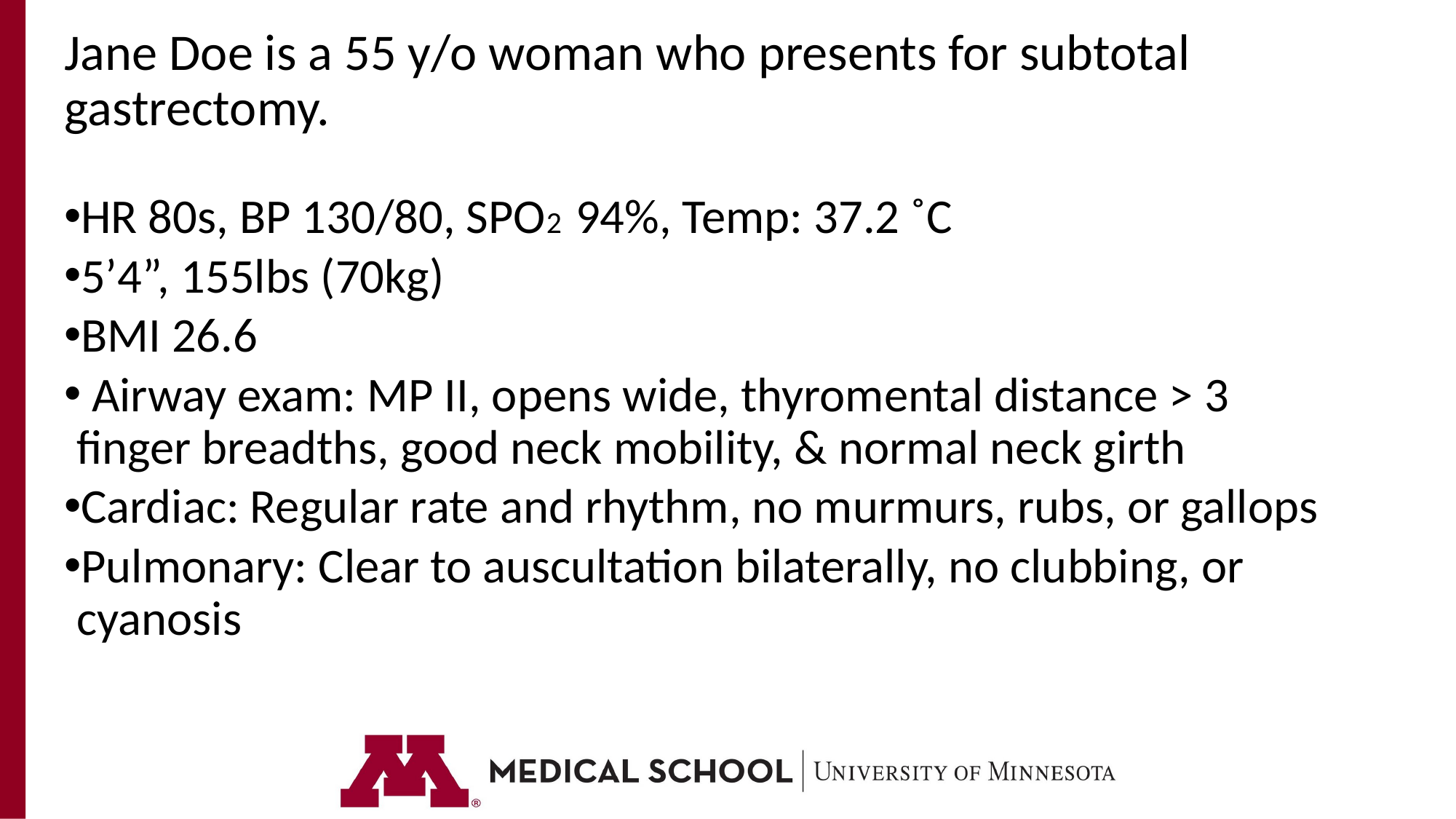

# Jane Doe is a 55 y/o woman who presents for subtotal gastrectomy.
HR 80s, BP 130/80, SPO2 94%, Temp: 37.2 ˚C
5’4”, 155lbs (70kg)
BMI 26.6
 Airway exam: MP II, opens wide, thyromental distance > 3 finger breadths, good neck mobility, & normal neck girth
Cardiac: Regular rate and rhythm, no murmurs, rubs, or gallops
Pulmonary: Clear to auscultation bilaterally, no clubbing, or cyanosis

## Slide 10
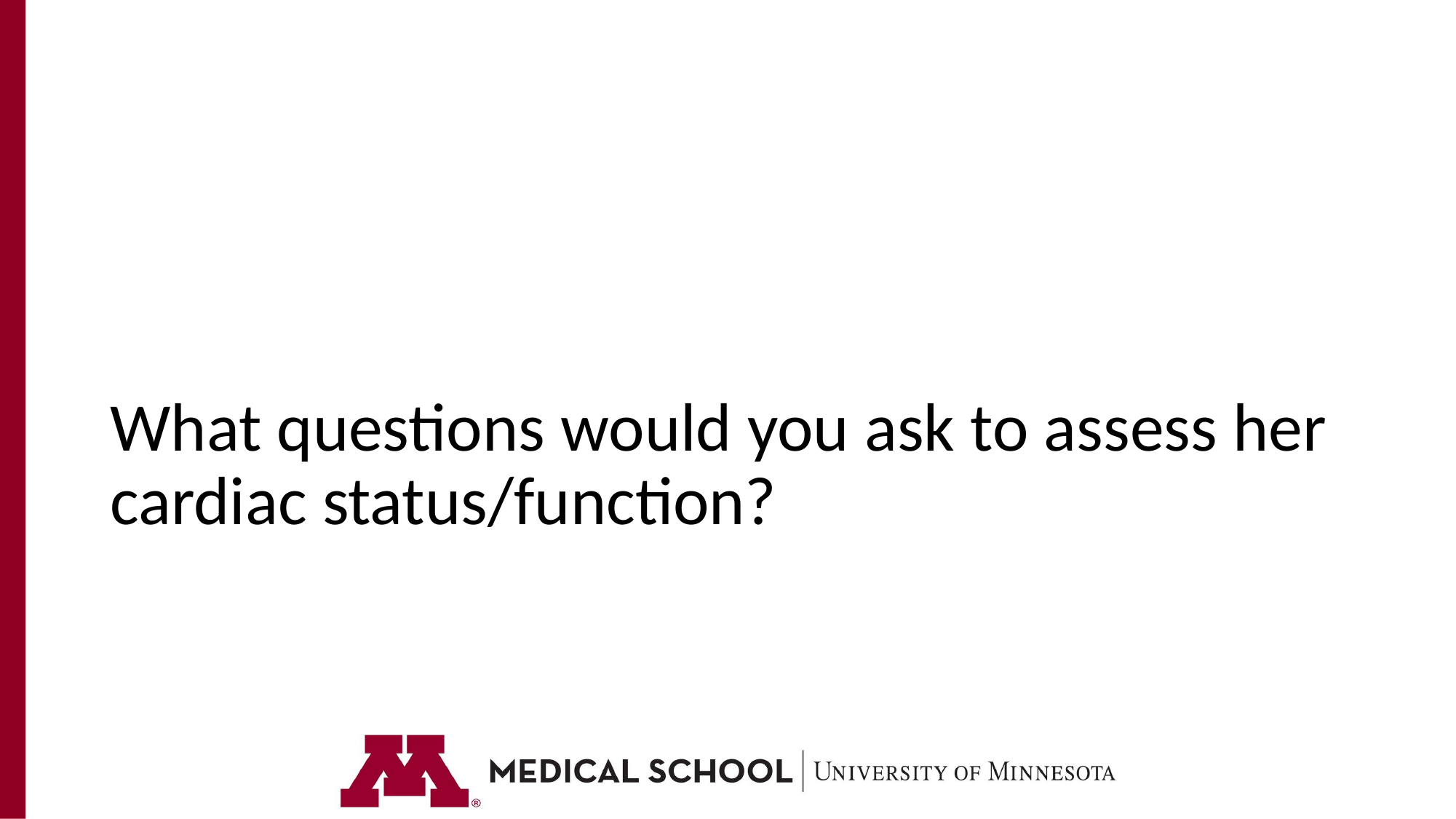

# What questions would you ask to assess her cardiac status/function?

## Slide 11
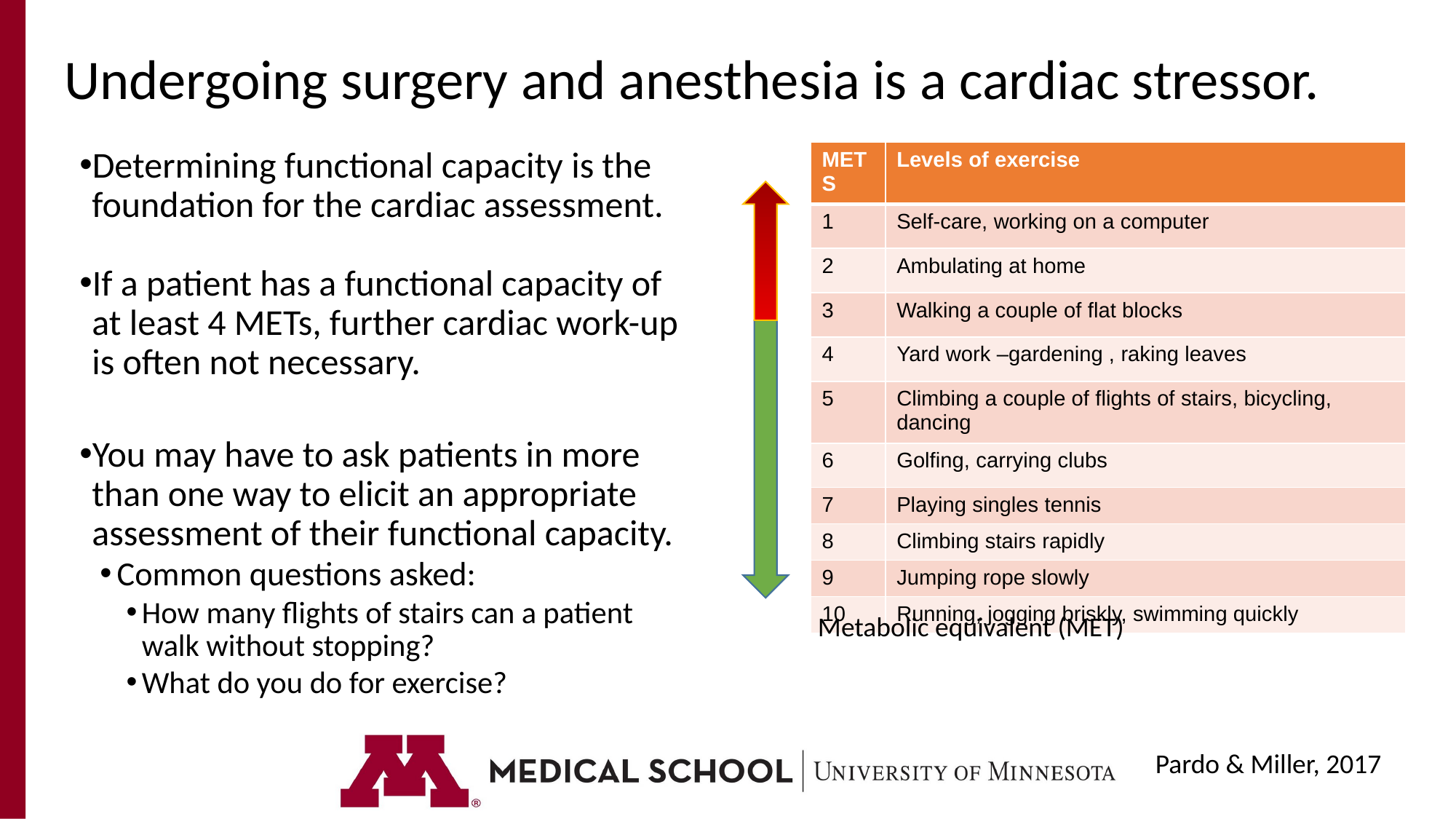

# Undergoing surgery and anesthesia is a cardiac stressor.
Determining functional capacity is the foundation for the cardiac assessment.
If a patient has a functional capacity of at least 4 METs, further cardiac work-up is often not necessary.
You may have to ask patients in more than one way to elicit an appropriate assessment of their functional capacity.
Common questions asked:
How many flights of stairs can a patient walk without stopping?
What do you do for exercise?
| METS | Levels of exercise |
| --- | --- |
| 1 | Self-care, working on a computer |
| 2 | Ambulating at home |
| 3 | Walking a couple of flat blocks |
| 4 | Yard work –gardening , raking leaves |
| 5 | Climbing a couple of flights of stairs, bicycling, dancing |
| 6 | Golfing, carrying clubs |
| 7 | Playing singles tennis |
| 8 | Climbing stairs rapidly |
| 9 | Jumping rope slowly |
| 10 | Running, jogging briskly, swimming quickly |
Metabolic equivalent (MET)
Pardo & Miller, 2017

## Slide 12
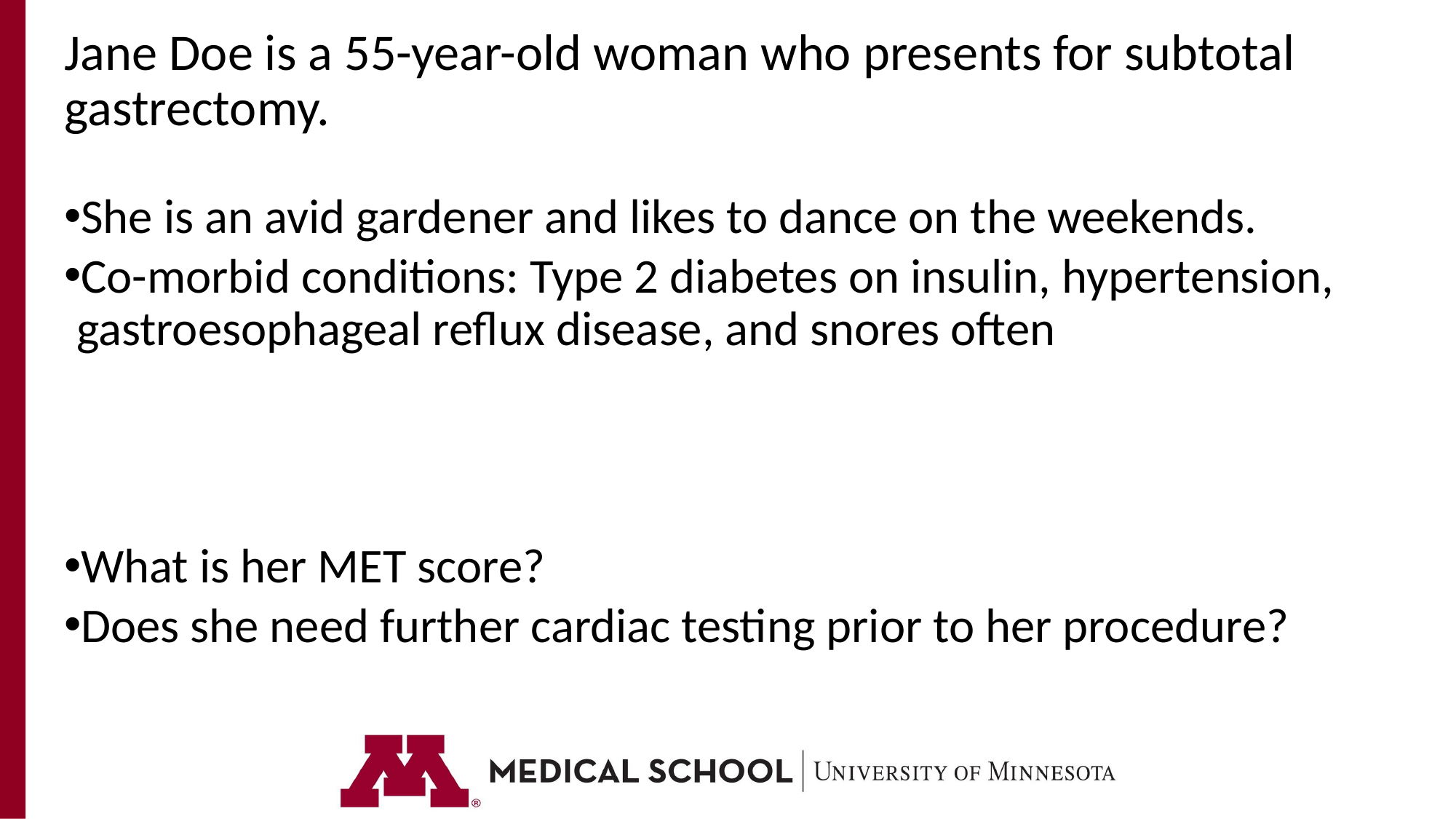

# Jane Doe is a 55-year-old woman who presents for subtotal gastrectomy.
She is an avid gardener and likes to dance on the weekends.
Co-morbid conditions: Type 2 diabetes on insulin, hypertension, gastroesophageal reflux disease, and snores often
What is her MET score?
Does she need further cardiac testing prior to her procedure?

## Slide 13
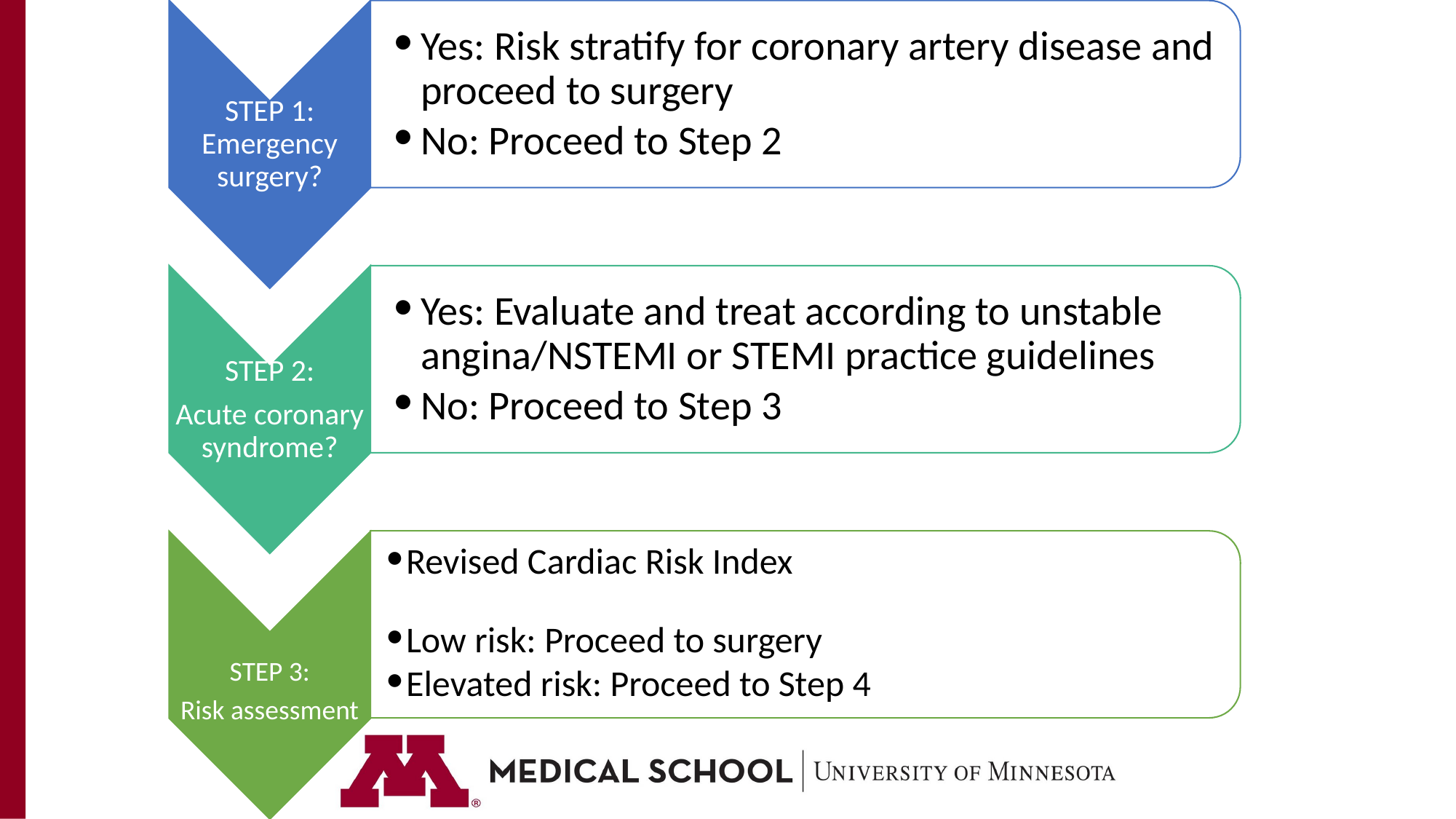

Yes: Risk stratify for coronary artery disease and proceed to surgery
No: Proceed to Step 2
STEP 1: Emergency surgery?
Yes: Evaluate and treat according to unstable angina/NSTEMI or STEMI practice guidelines
No: Proceed to Step 3
STEP 2:
Acute coronary syndrome?
Revised Cardiac Risk Index
Low risk: Proceed to surgery
Elevated risk: Proceed to Step 4
STEP 3:
Risk assessment

## Slide 14
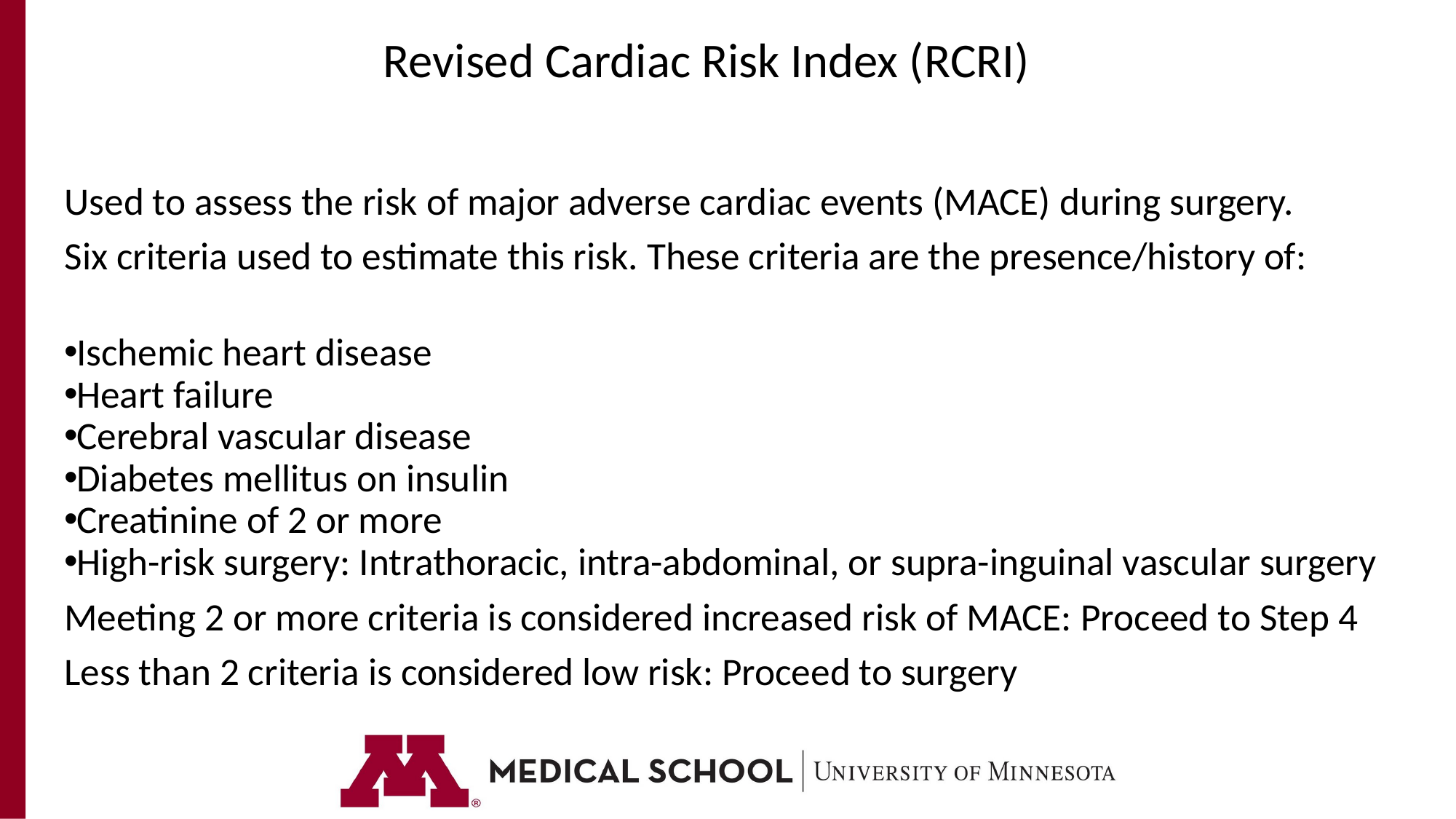

# Revised Cardiac Risk Index (RCRI)
Used to assess the risk of major adverse cardiac events (MACE) during surgery.
Six criteria used to estimate this risk. These criteria are the presence/history of:
Ischemic heart disease
Heart failure
Cerebral vascular disease
Diabetes mellitus on insulin
Creatinine of 2 or more
High-risk surgery: Intrathoracic, intra-abdominal, or supra-inguinal vascular surgery
Meeting 2 or more criteria is considered increased risk of MACE: Proceed to Step 4
Less than 2 criteria is considered low risk: Proceed to surgery

## Slide 15
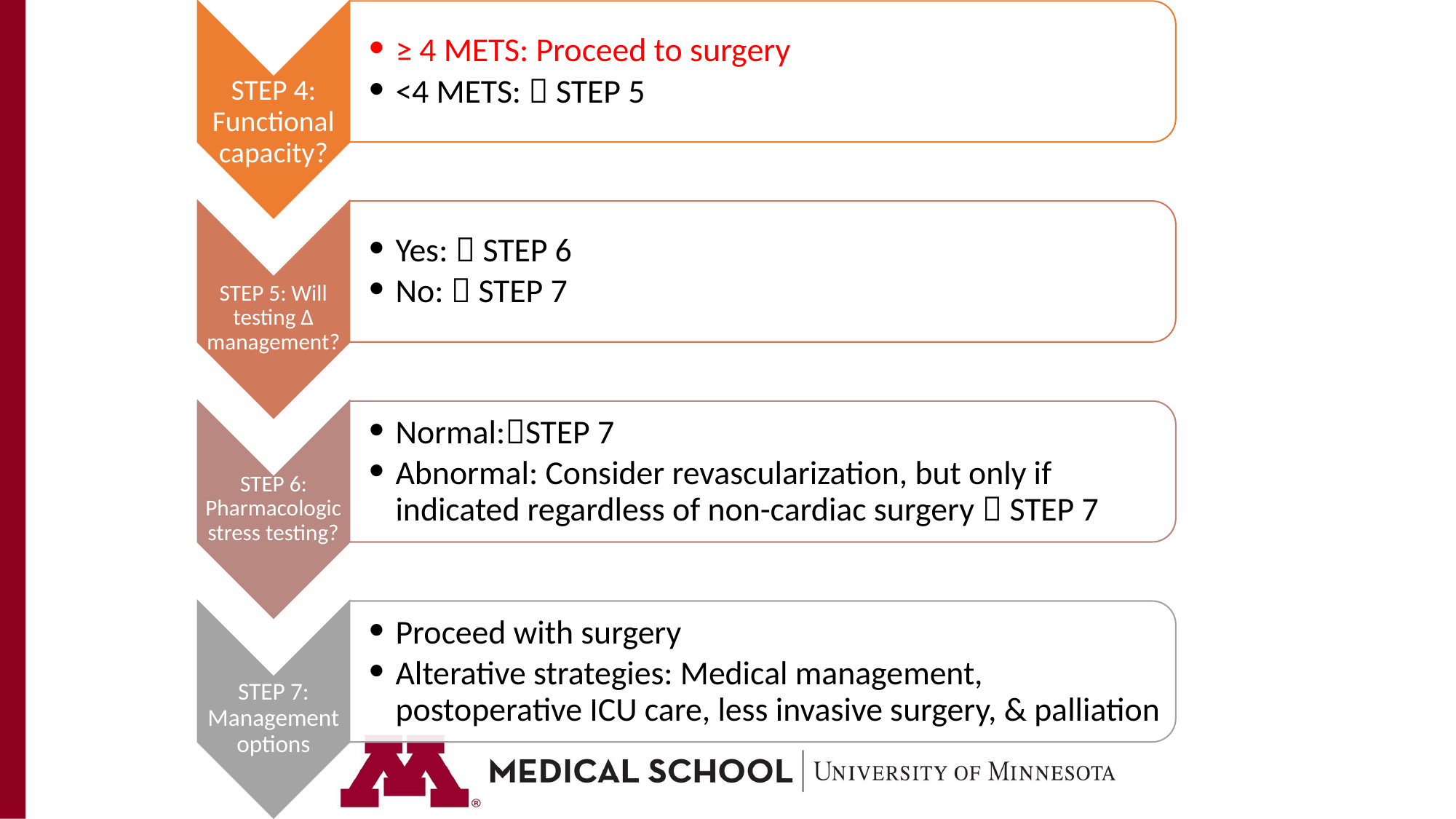

≥ 4 METS: Proceed to surgery
<4 METS:  STEP 5
STEP 4: Functional capacity?
Yes:  STEP 6
No:  STEP 7
STEP 5: Will testing Δ management?
Normal:STEP 7
Abnormal: Consider revascularization, but only if indicated regardless of non-cardiac surgery  STEP 7
STEP 6: Pharmacologic stress testing?
Proceed with surgery
Alterative strategies: Medical management, postoperative ICU care, less invasive surgery, & palliation
STEP 7: Management options

## Slide 16
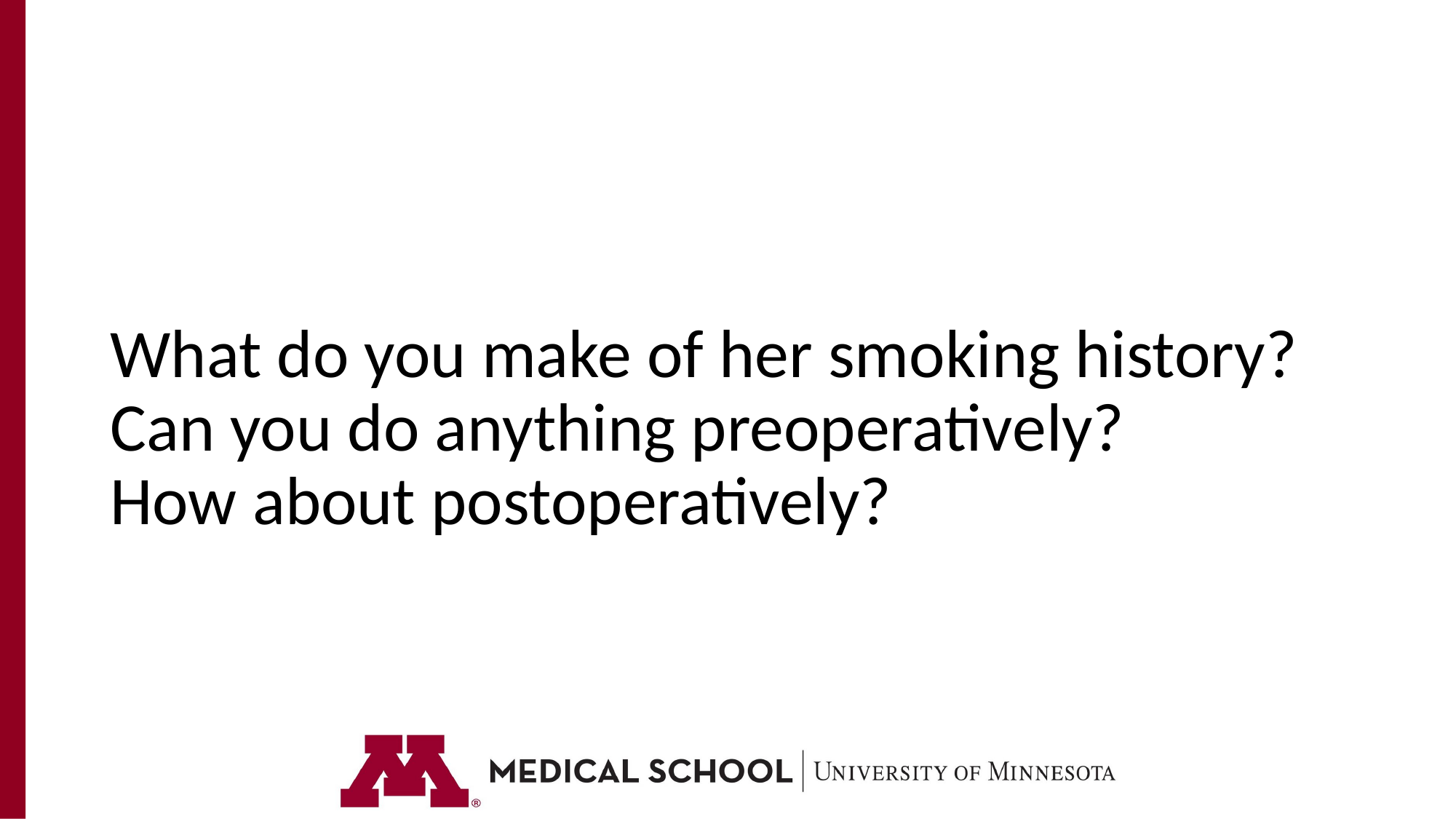

# What do you make of her smoking history? Can you do anything preoperatively? How about postoperatively?

## Slide 17
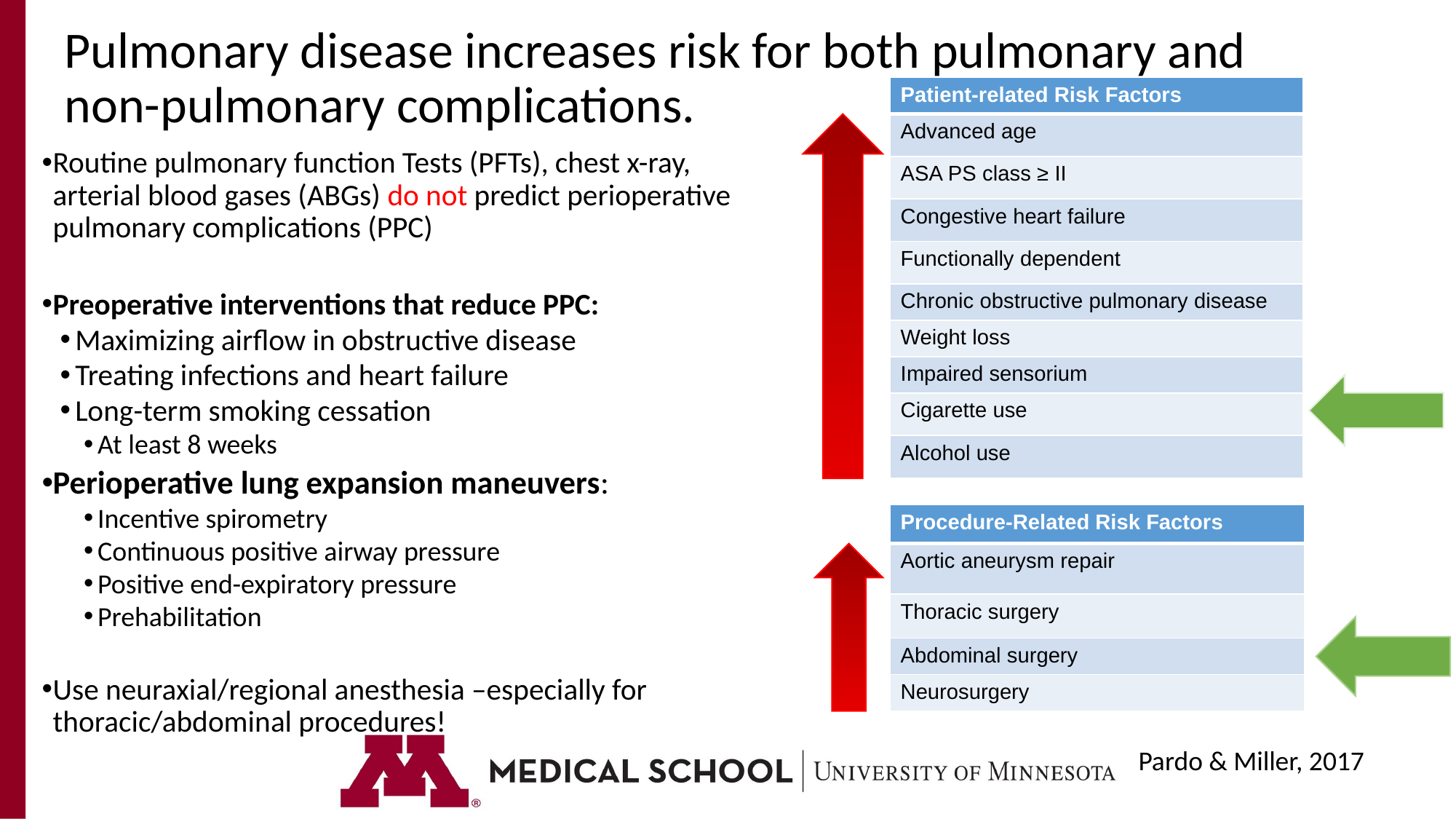

# Pulmonary disease increases risk for both pulmonary and non-pulmonary complications.
| Patient-related Risk Factors |
| --- |
| Advanced age |
| ASA PS class ≥ II |
| Congestive heart failure |
| Functionally dependent |
| Chronic obstructive pulmonary disease |
| Weight loss |
| Impaired sensorium |
| Cigarette use |
| Alcohol use |
Routine pulmonary function Tests (PFTs), chest x-ray, arterial blood gases (ABGs) do not predict perioperative pulmonary complications (PPC)
Preoperative interventions that reduce PPC:
Maximizing airflow in obstructive disease
Treating infections and heart failure
Long-term smoking cessation
At least 8 weeks
Perioperative lung expansion maneuvers:
Incentive spirometry
Continuous positive airway pressure
Positive end-expiratory pressure
Prehabilitation
Use neuraxial/regional anesthesia –especially for thoracic/abdominal procedures!
| Procedure-Related Risk Factors |
| --- |
| Aortic aneurysm repair |
| Thoracic surgery |
| Abdominal surgery |
| Neurosurgery |
Pardo & Miller, 2017

## Slide 18
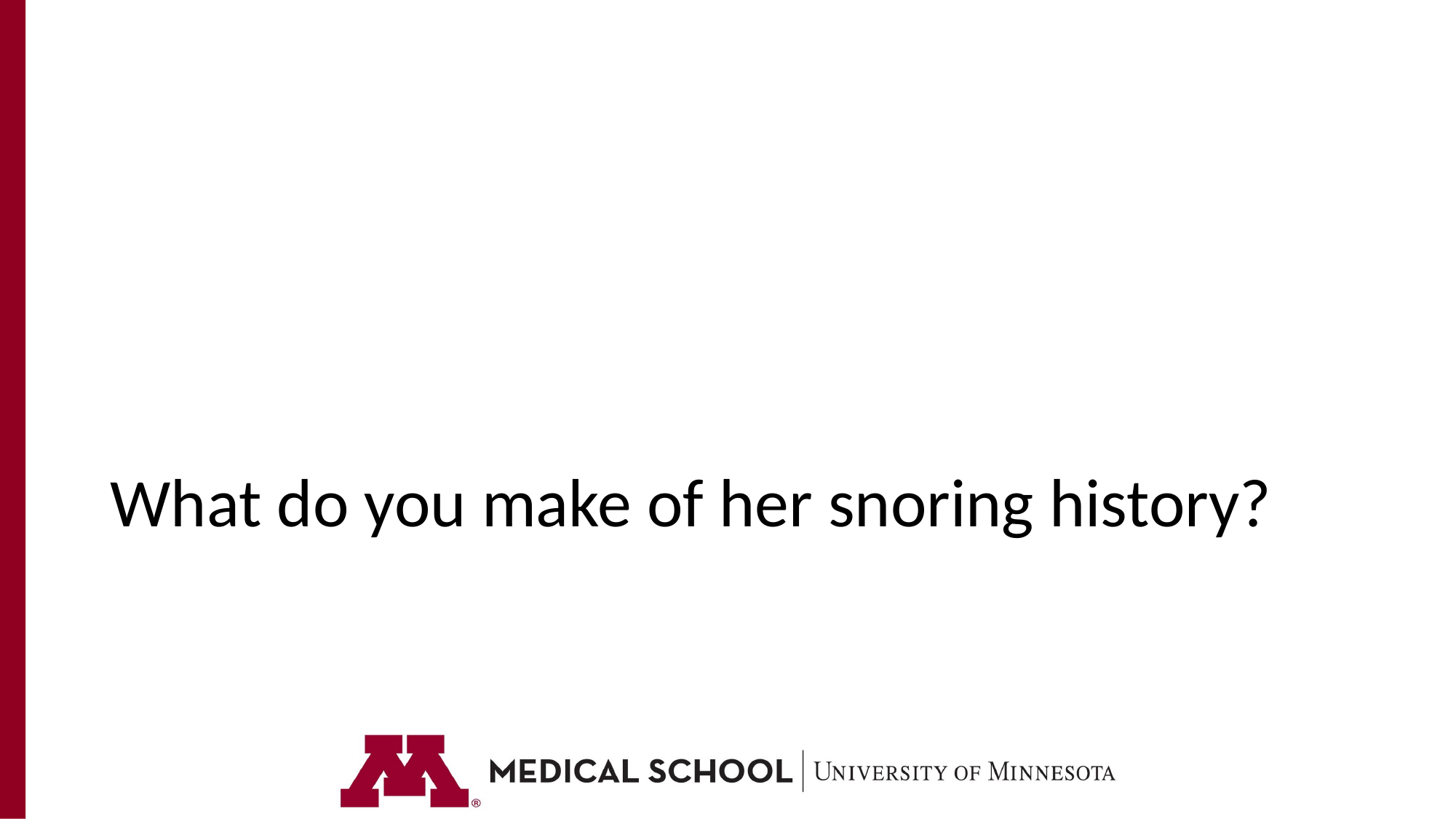

# What do you make of her snoring history?

## Slide 19
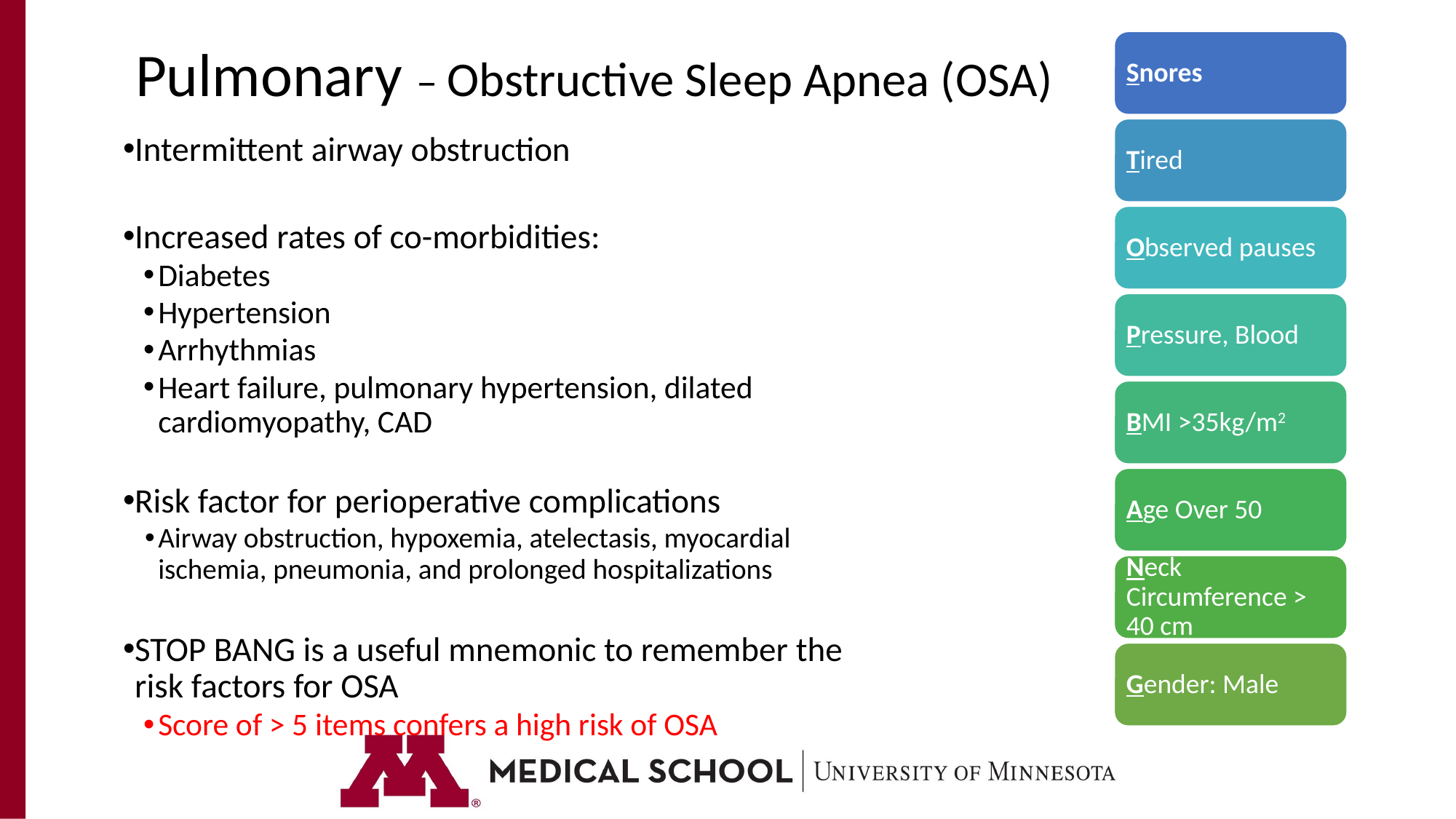

# Pulmonary – Obstructive Sleep Apnea (OSA)
Snores
Tired
Observed pauses
Pressure, Blood
BMI >35kg/m2
Age Over 50
Neck Circumference > 40 cm
Gender: Male
Intermittent airway obstruction
Increased rates of co-morbidities:
Diabetes
Hypertension
Arrhythmias
Heart failure, pulmonary hypertension, dilated cardiomyopathy, CAD
Risk factor for perioperative complications
Airway obstruction, hypoxemia, atelectasis, myocardial ischemia, pneumonia, and prolonged hospitalizations
STOP BANG is a useful mnemonic to remember the risk factors for OSA
Score of > 5 items confers a high risk of OSA

## Slide 20
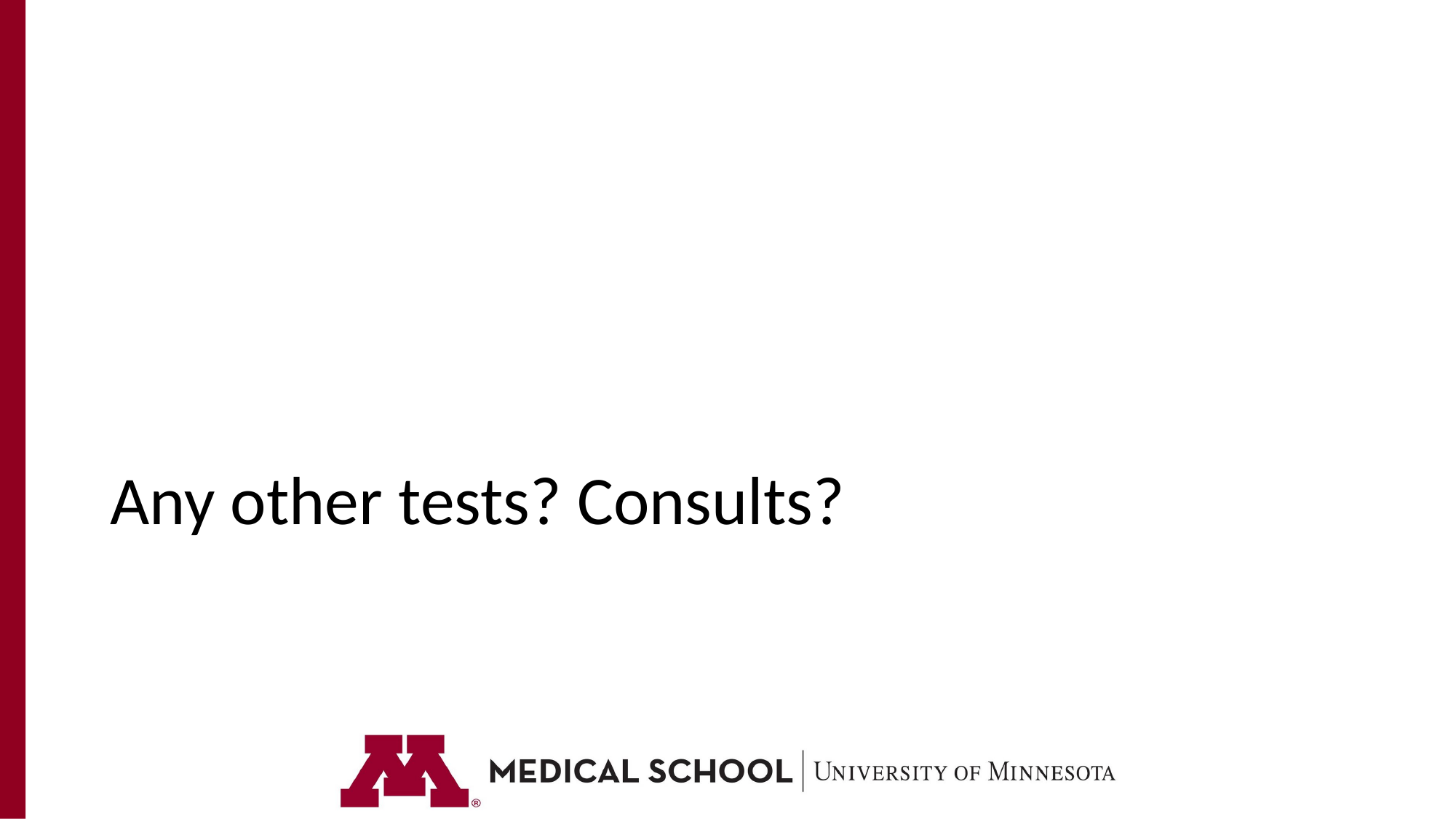

# Any other tests? Consults?

## Slide 21
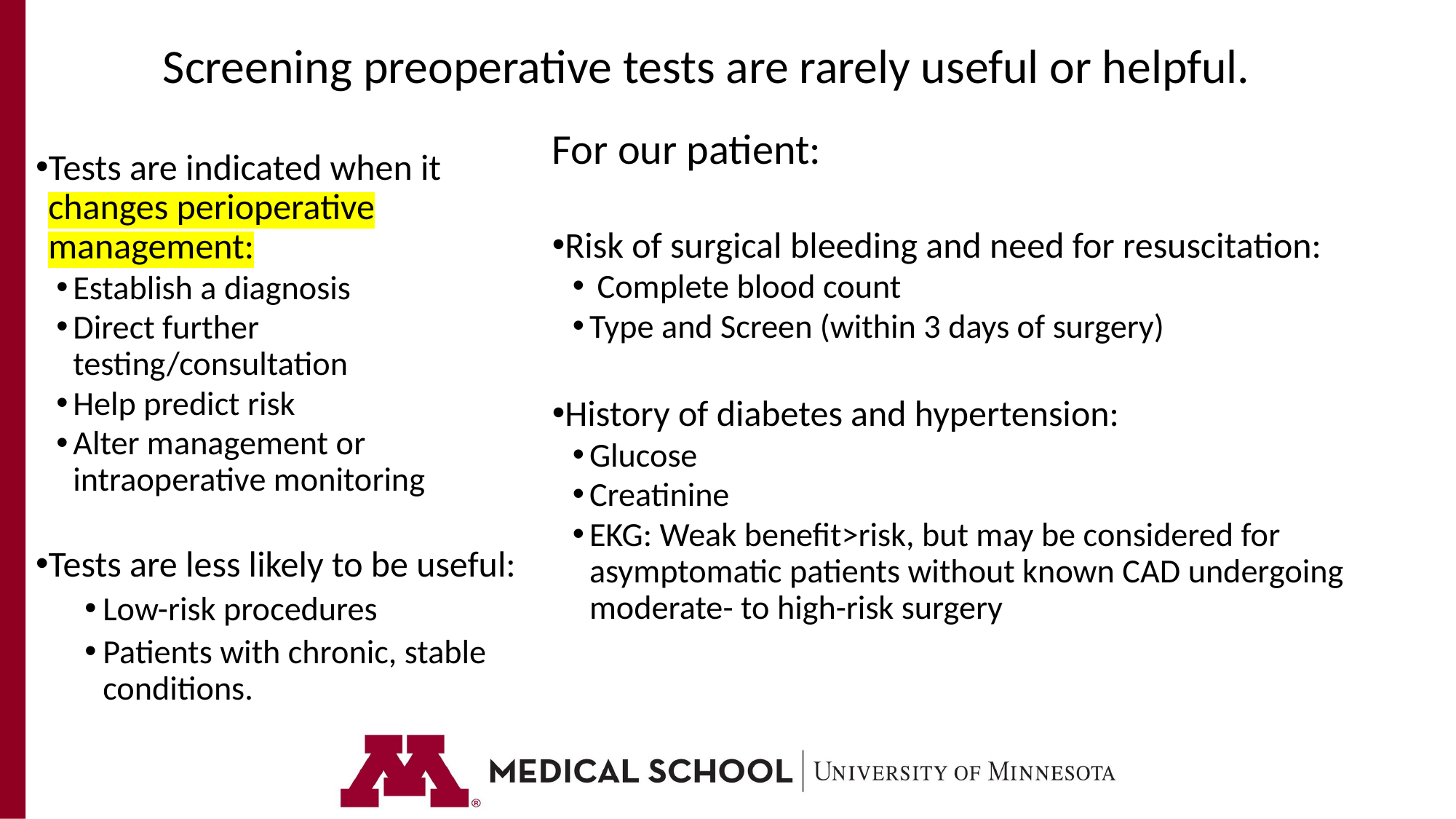

# Screening preoperative tests are rarely useful or helpful.
For our patient:
Risk of surgical bleeding and need for resuscitation:
 Complete blood count
Type and Screen (within 3 days of surgery)
History of diabetes and hypertension:
Glucose
Creatinine
EKG: Weak benefit>risk, but may be considered for asymptomatic patients without known CAD undergoing moderate- to high-risk surgery
Tests are indicated when it changes perioperative management:
Establish a diagnosis
Direct further testing/consultation
Help predict risk
Alter management or intraoperative monitoring
Tests are less likely to be useful:
Low-risk procedures
Patients with chronic, stable conditions.

## Slide 22
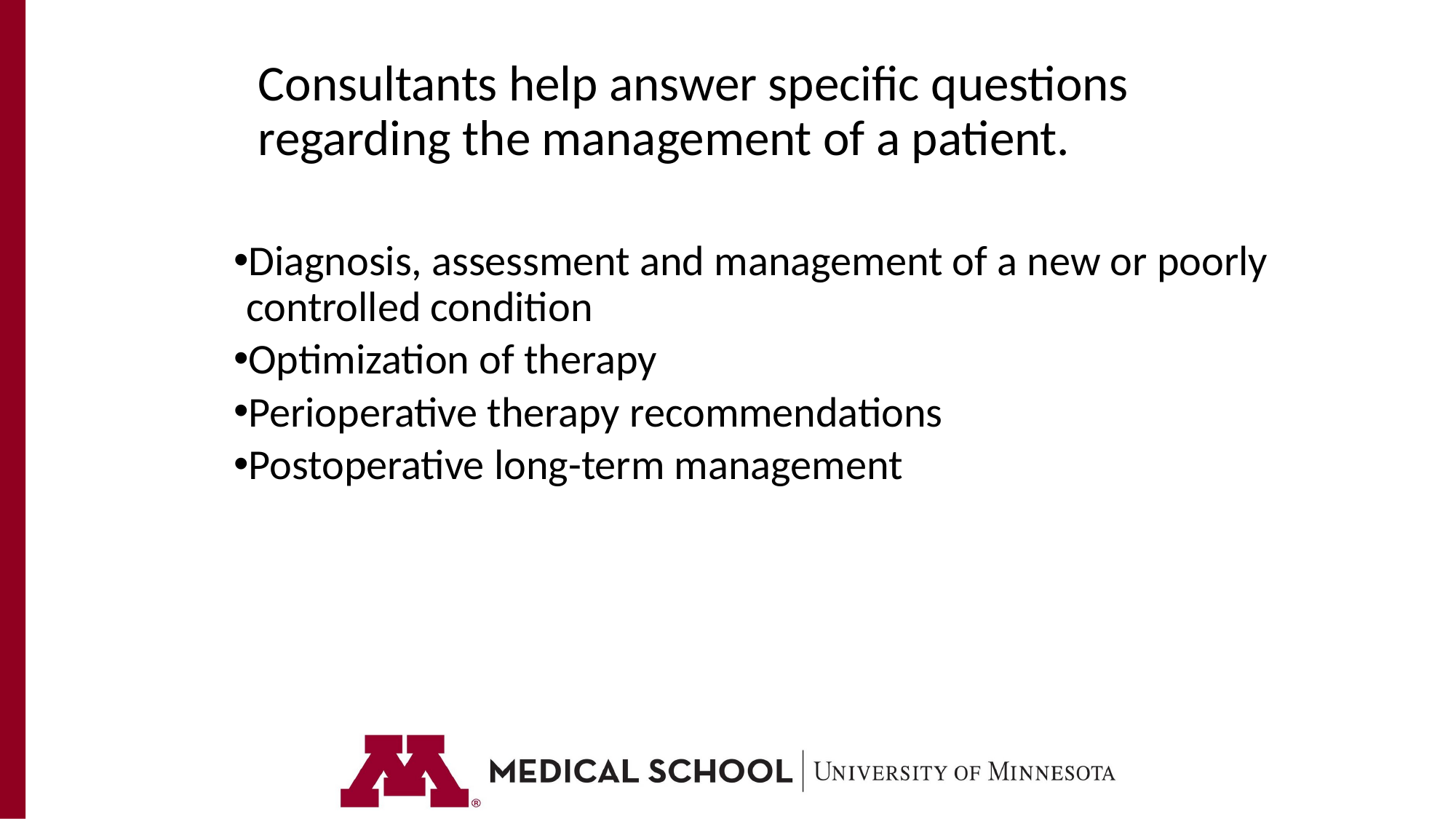

# Consultants help answer specific questions regarding the management of a patient.
Diagnosis, assessment and management of a new or poorly controlled condition
Optimization of therapy
Perioperative therapy recommendations
Postoperative long-term management

## Slide 23
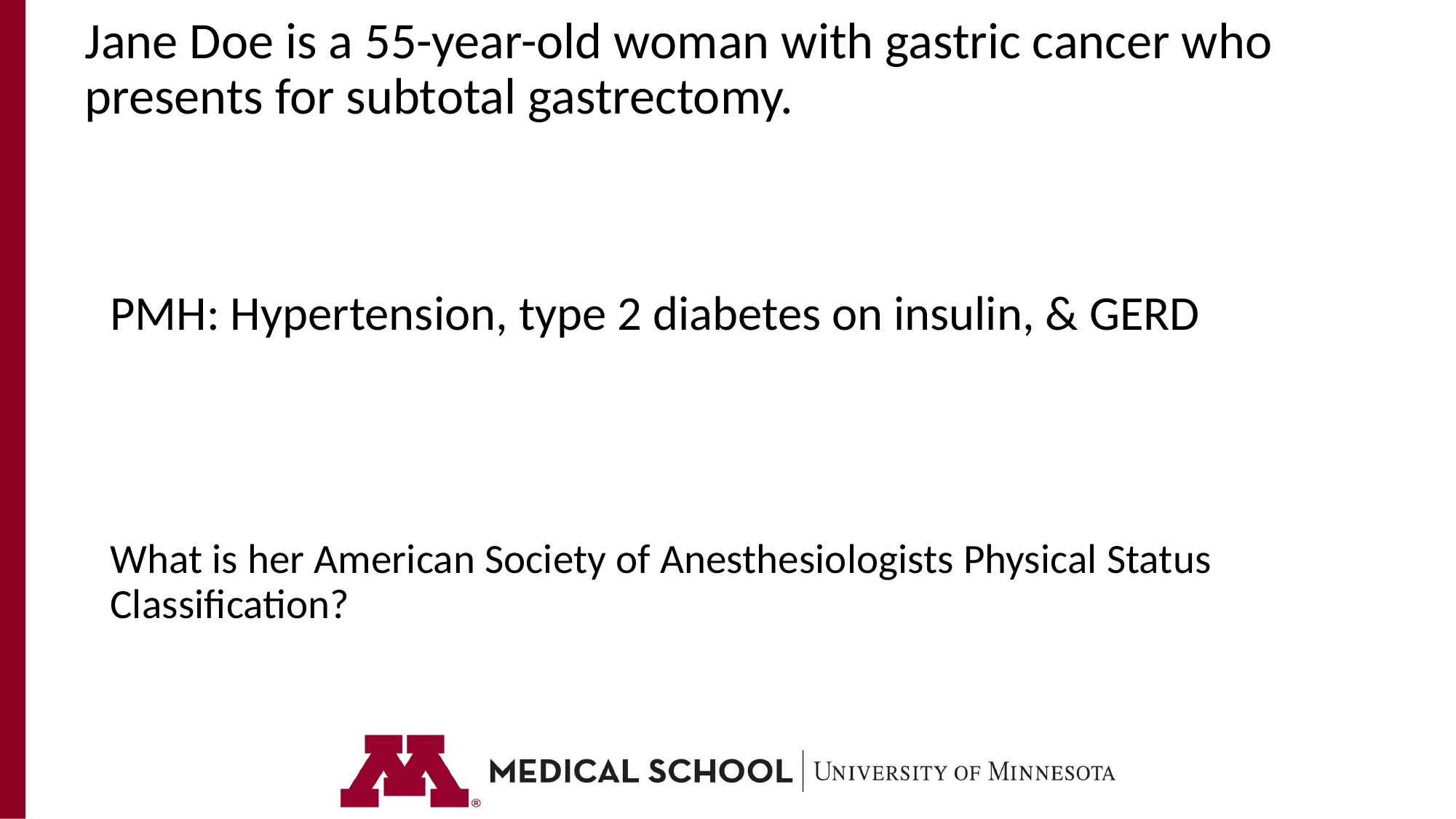

Jane Doe is a 55-year-old woman with gastric cancer who presents for subtotal gastrectomy.
PMH: Hypertension, type 2 diabetes on insulin, & GERD
# What is her American Society of Anesthesiologists Physical Status Classification?

## Slide 24
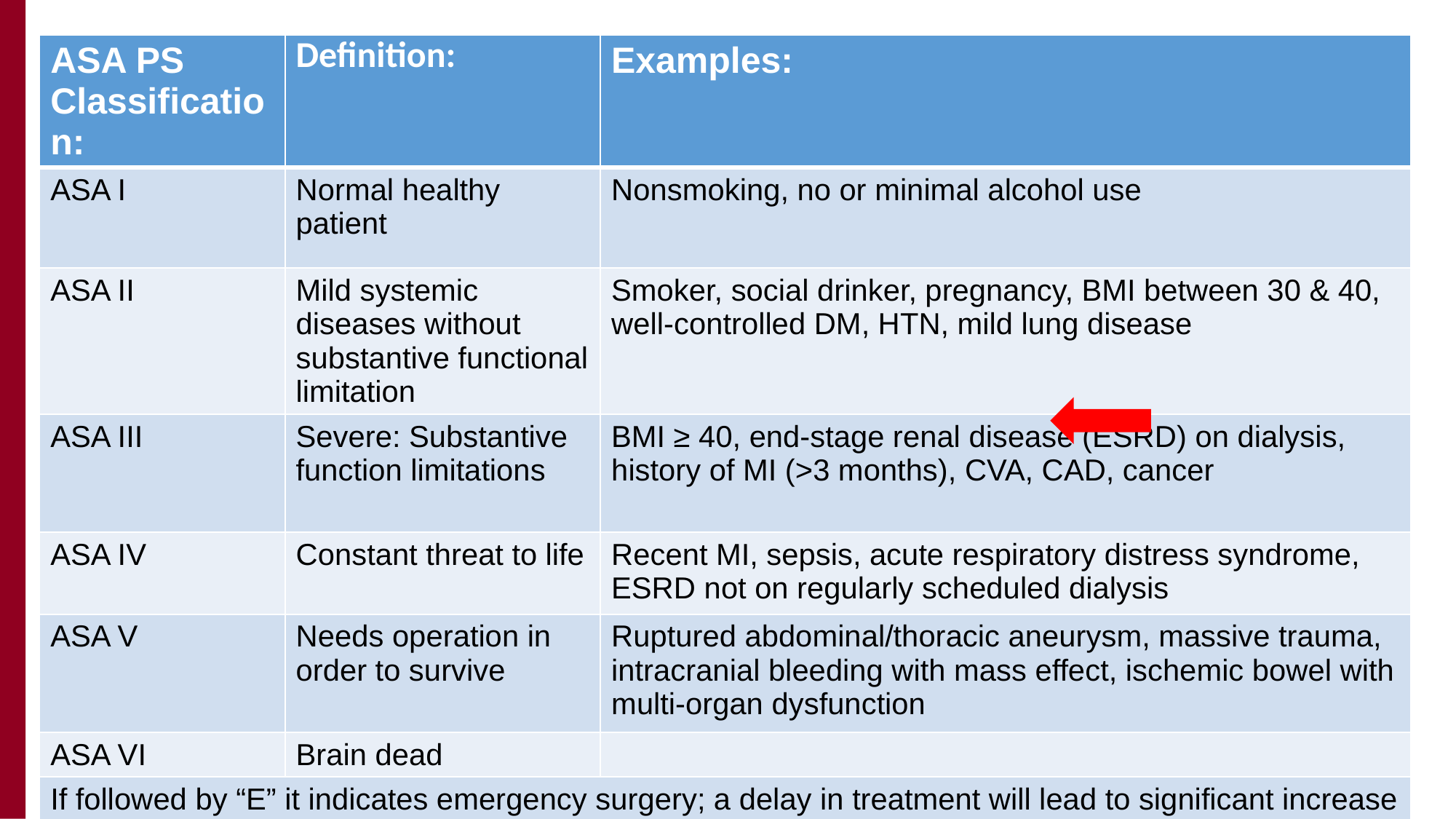

| ASA PS Classification: | Definition: | Examples: |
| --- | --- | --- |
| ASA I | Normal healthy patient | Nonsmoking, no or minimal alcohol use |
| ASA II | Mild systemic diseases without substantive functional limitation | Smoker, social drinker, pregnancy, BMI between 30 & 40, well-controlled DM, HTN, mild lung disease |
| ASA III | Severe: Substantive function limitations | BMI ≥ 40, end-stage renal disease (ESRD) on dialysis, history of MI (>3 months), CVA, CAD, cancer |
| ASA IV | Constant threat to life | Recent MI, sepsis, acute respiratory distress syndrome, ESRD not on regularly scheduled dialysis |
| ASA V | Needs operation in order to survive | Ruptured abdominal/thoracic aneurysm, massive trauma, intracranial bleeding with mass effect, ischemic bowel with multi-organ dysfunction |
| ASA VI | Brain dead | |
| If followed by “E” it indicates emergency surgery; a delay in treatment will lead to significant increase in threat to life or body part. | | |

## Slide 25
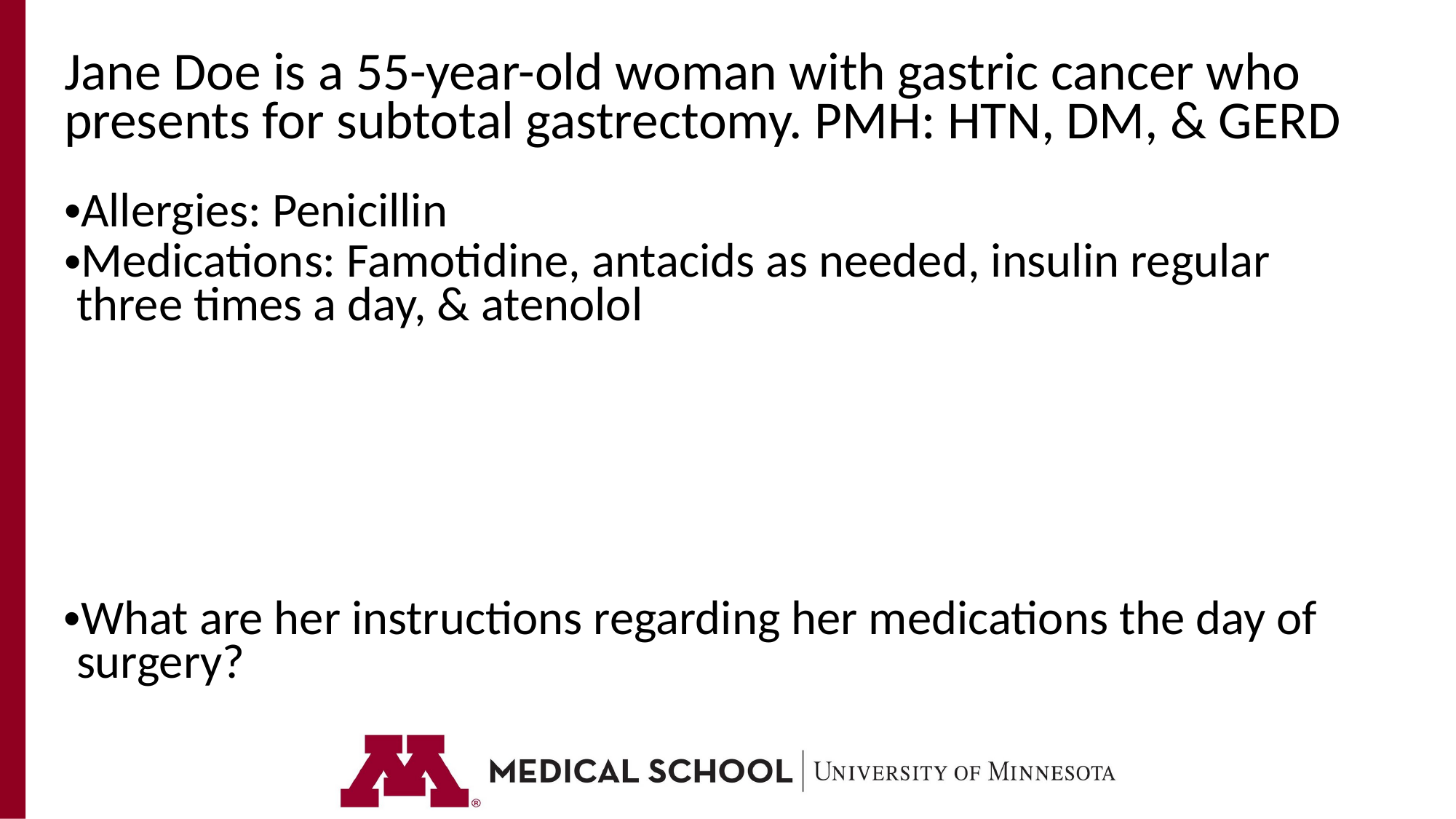

Jane Doe is a 55-year-old woman with gastric cancer who presents for subtotal gastrectomy. PMH: HTN, DM, & GERD
Allergies: Penicillin
Medications: Famotidine, antacids as needed, insulin regular three times a day, & atenolol
What are her instructions regarding her medications the day of surgery?

## Slide 26
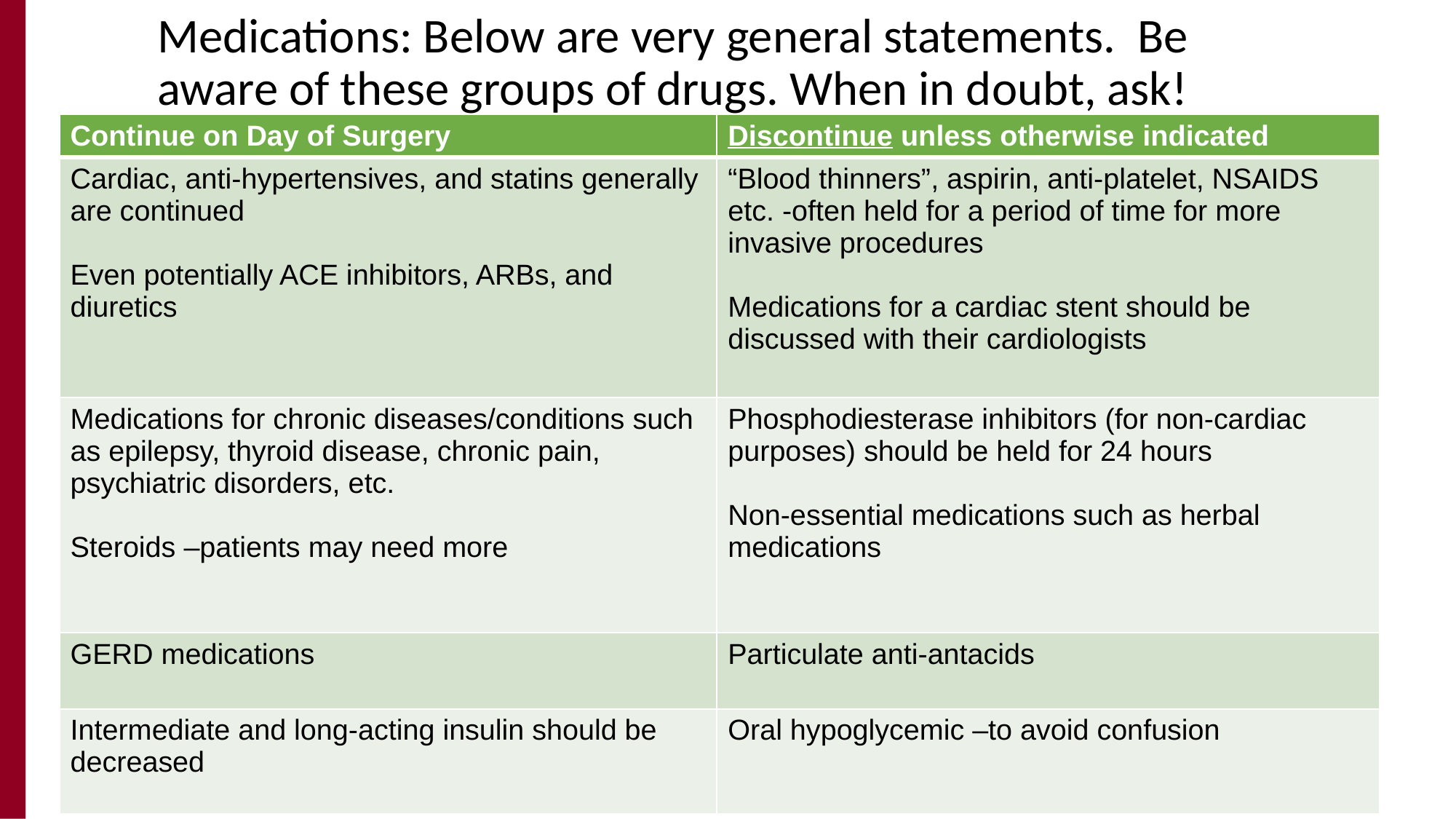

# Medications: Below are very general statements. Be aware of these groups of drugs. When in doubt, ask!
| Continue on Day of Surgery | Discontinue unless otherwise indicated |
| --- | --- |
| Cardiac, anti-hypertensives, and statins generally are continued Even potentially ACE inhibitors, ARBs, and diuretics | “Blood thinners”, aspirin, anti-platelet, NSAIDS etc. -often held for a period of time for more invasive procedures Medications for a cardiac stent should be discussed with their cardiologists |
| Medications for chronic diseases/conditions such as epilepsy, thyroid disease, chronic pain, psychiatric disorders, etc. Steroids –patients may need more | Phosphodiesterase inhibitors (for non-cardiac purposes) should be held for 24 hours Non-essential medications such as herbal medications |
| GERD medications | Particulate anti-antacids |
| Intermediate and long-acting insulin should be decreased | Oral hypoglycemic –to avoid confusion |

## Slide 27
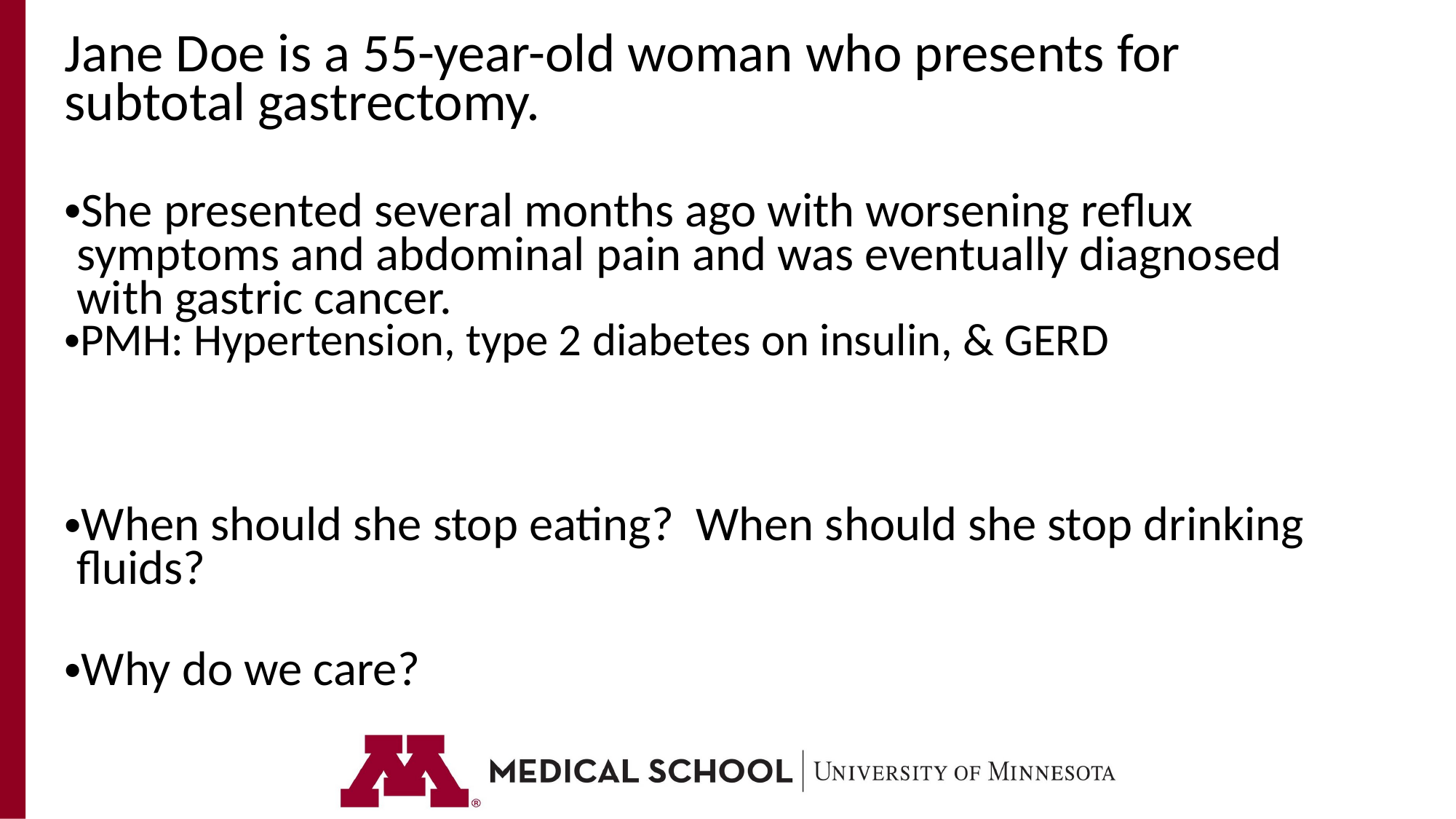

# Jane Doe is a 55-year-old woman who presents for subtotal gastrectomy.
She presented several months ago with worsening reflux symptoms and abdominal pain and was eventually diagnosed with gastric cancer.
PMH: Hypertension, type 2 diabetes on insulin, & GERD
When should she stop eating? When should she stop drinking fluids?
Why do we care?

## Slide 28
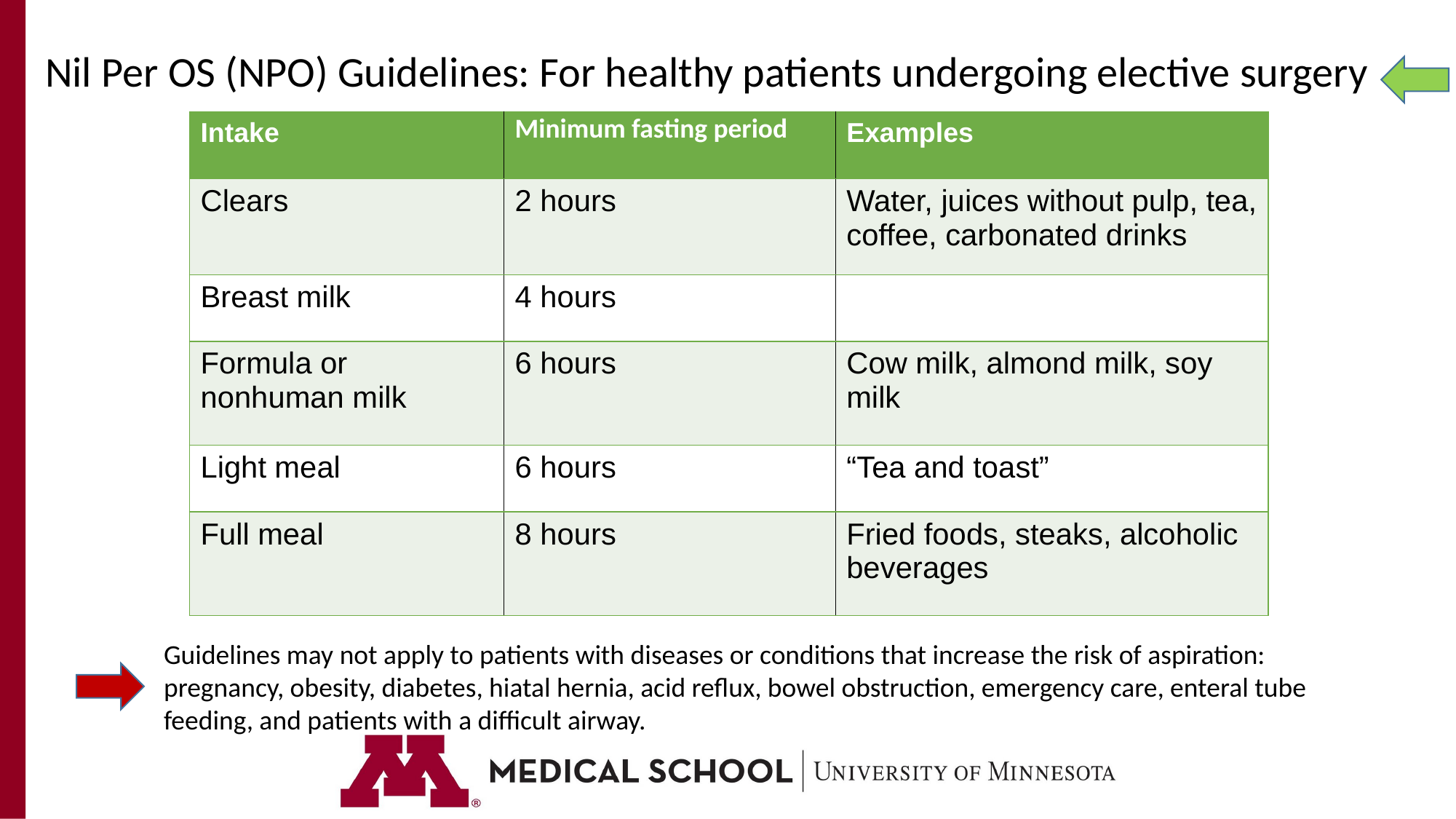

# Nil Per OS (NPO) Guidelines: For healthy patients undergoing elective surgery
| Intake | Minimum fasting period | Examples |
| --- | --- | --- |
| Clears | 2 hours | Water, juices without pulp, tea, coffee, carbonated drinks |
| Breast milk | 4 hours | |
| Formula or nonhuman milk | 6 hours | Cow milk, almond milk, soy milk |
| Light meal | 6 hours | “Tea and toast” |
| Full meal | 8 hours | Fried foods, steaks, alcoholic beverages |
Guidelines may not apply to patients with diseases or conditions that increase the risk of aspiration: pregnancy, obesity, diabetes, hiatal hernia, acid reflux, bowel obstruction, emergency care, enteral tube feeding, and patients with a difficult airway.

## Slide 29
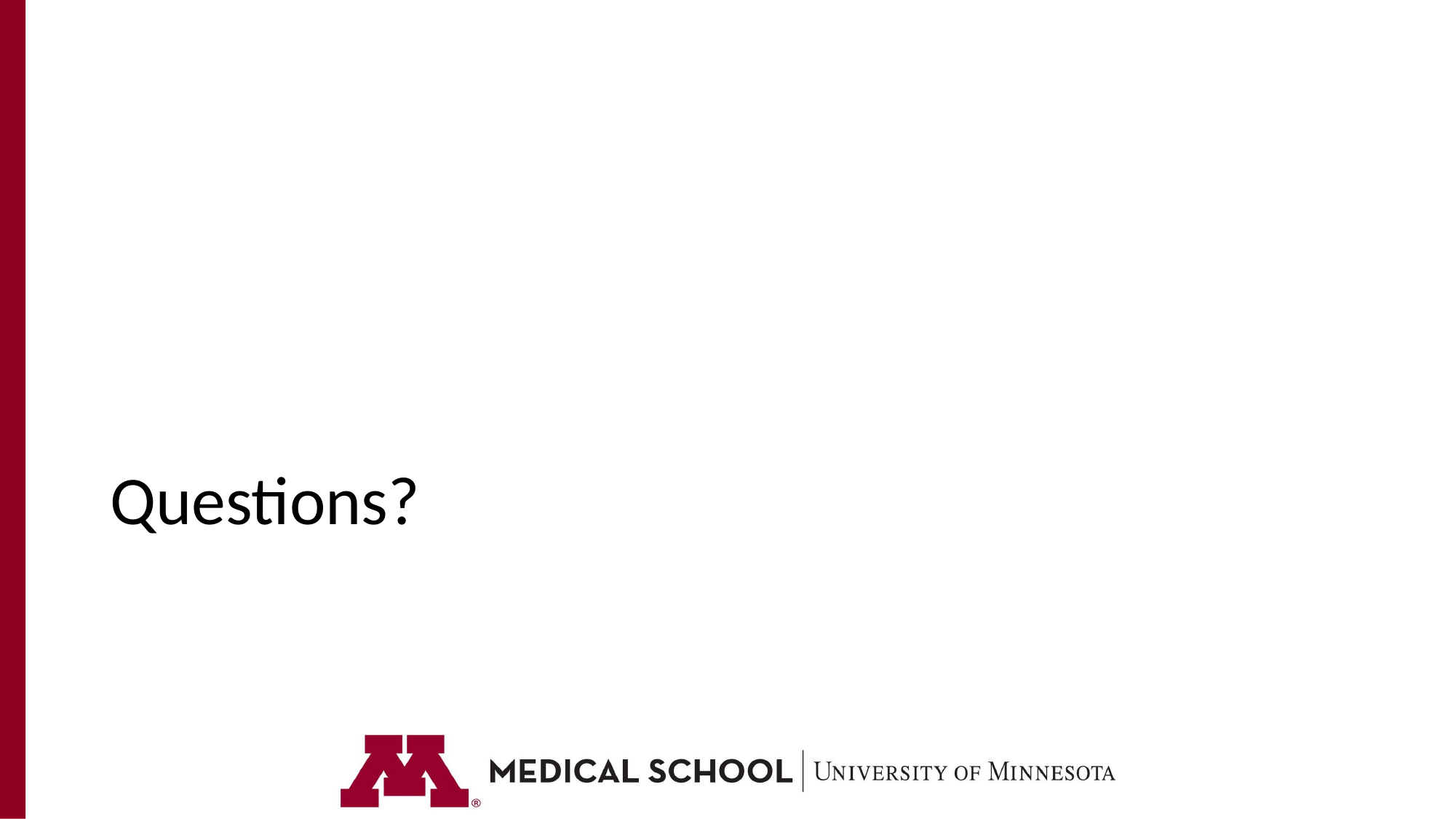

# Questions?

## Slide 30
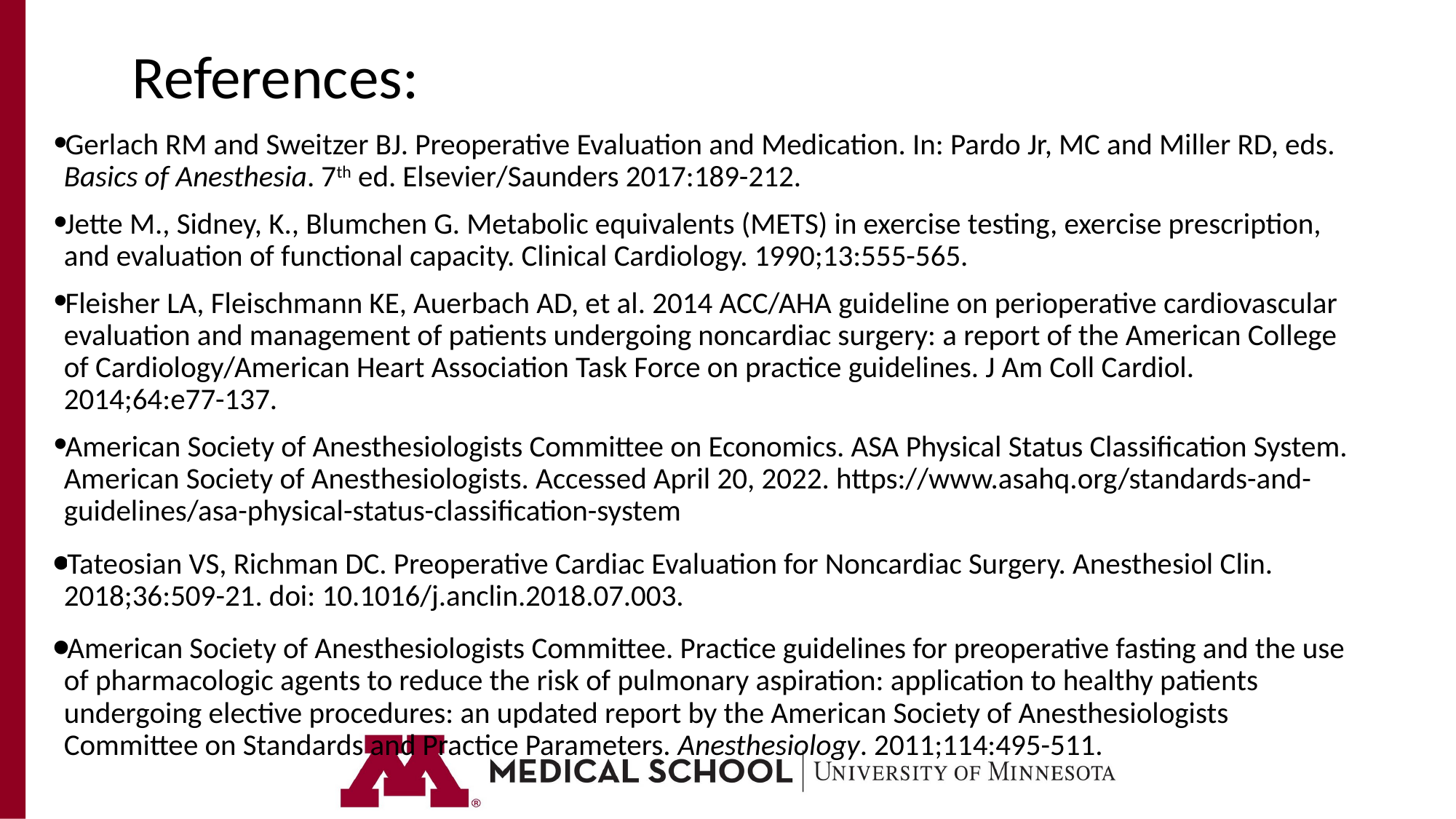

# References:
Gerlach RM and Sweitzer BJ. Preoperative Evaluation and Medication. In: Pardo Jr, MC and Miller RD, eds. Basics of Anesthesia. 7th ed. Elsevier/Saunders 2017:189-212.
Jette M., Sidney, K., Blumchen G. Metabolic equivalents (METS) in exercise testing, exercise prescription, and evaluation of functional capacity. Clinical Cardiology. 1990;13:555-565.
Fleisher LA, Fleischmann KE, Auerbach AD, et al. 2014 ACC/AHA guideline on perioperative cardiovascular evaluation and management of patients undergoing noncardiac surgery: a report of the American College of Cardiology/American Heart Association Task Force on practice guidelines. J Am Coll Cardiol. 2014;64:e77-137.
American Society of Anesthesiologists Committee on Economics. ASA Physical Status Classification System. American Society of Anesthesiologists. Accessed April 20, 2022. https://www.asahq.org/standards-and-guidelines/asa-physical-status-classification-system
Tateosian VS, Richman DC. Preoperative Cardiac Evaluation for Noncardiac Surgery. Anesthesiol Clin. 2018;36:509-21. doi: 10.1016/j.anclin.2018.07.003.
American Society of Anesthesiologists Committee. Practice guidelines for preoperative fasting and the use of pharmacologic agents to reduce the risk of pulmonary aspiration: application to healthy patients undergoing elective procedures: an updated report by the American Society of Anesthesiologists Committee on Standards and Practice Parameters. Anesthesiology. 2011;114:495-511.
