# Supplementary material for: Development and Implementation of Video-Recorded Simulation Scenarios to Facilitate Case-Based Learning Discussions for Medical Students' Virtual Anesthesiology Clerkship
Source: MedEdPORTAL. 2023 Apr 4;19:11306. doi: 10.15766/mep_2374-8265.11306 (PMC10070881; doi:10.15766/mep_2374-8265.11306)
Supplement: Supplementary file 1 — Preoperative Evaluation - CBLD 1.pptxInhaled and Intravenous Anesthetics - CBLD 2.pptxAirway Management - CBLD 3.pptxScenario 1.mp4Scenario 2.mp4Scenario 3.mp4Scenario Debrief 1.docxScenario Debrief 2.docxScenario Debrief 3.docxClerkship Survey Questions.docxCBLD-Specific Survey Questions.docx [file mep_2374-8265.11306-s001.zip › B. Inhaled and Intravenous Anesthetics - CBLD 2.pptx]

## Slide 1
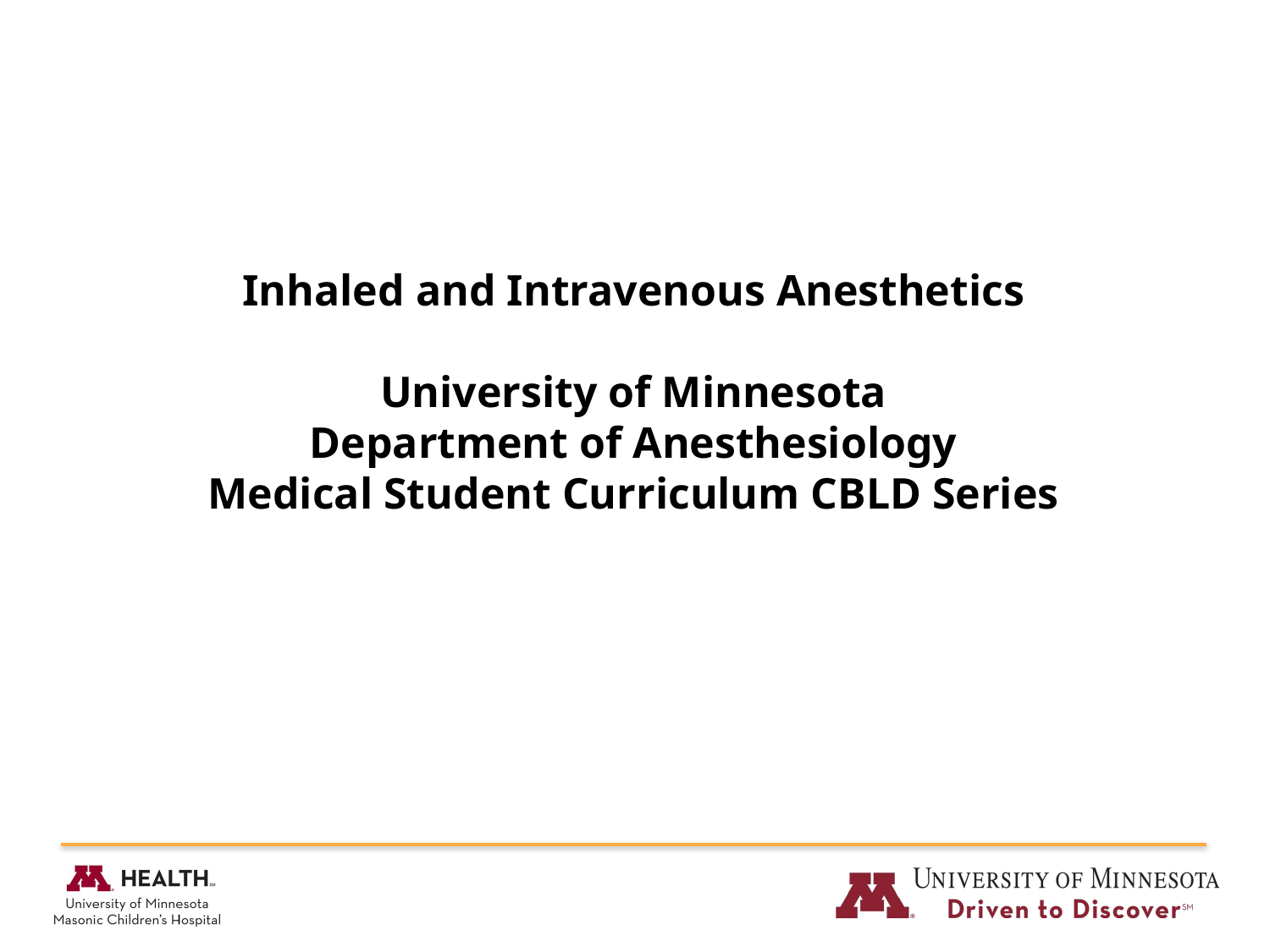

Inhaled and Intravenous Anesthetics
University of Minnesota
Department of Anesthesiology
Medical Student Curriculum CBLD Series

## Slide 2
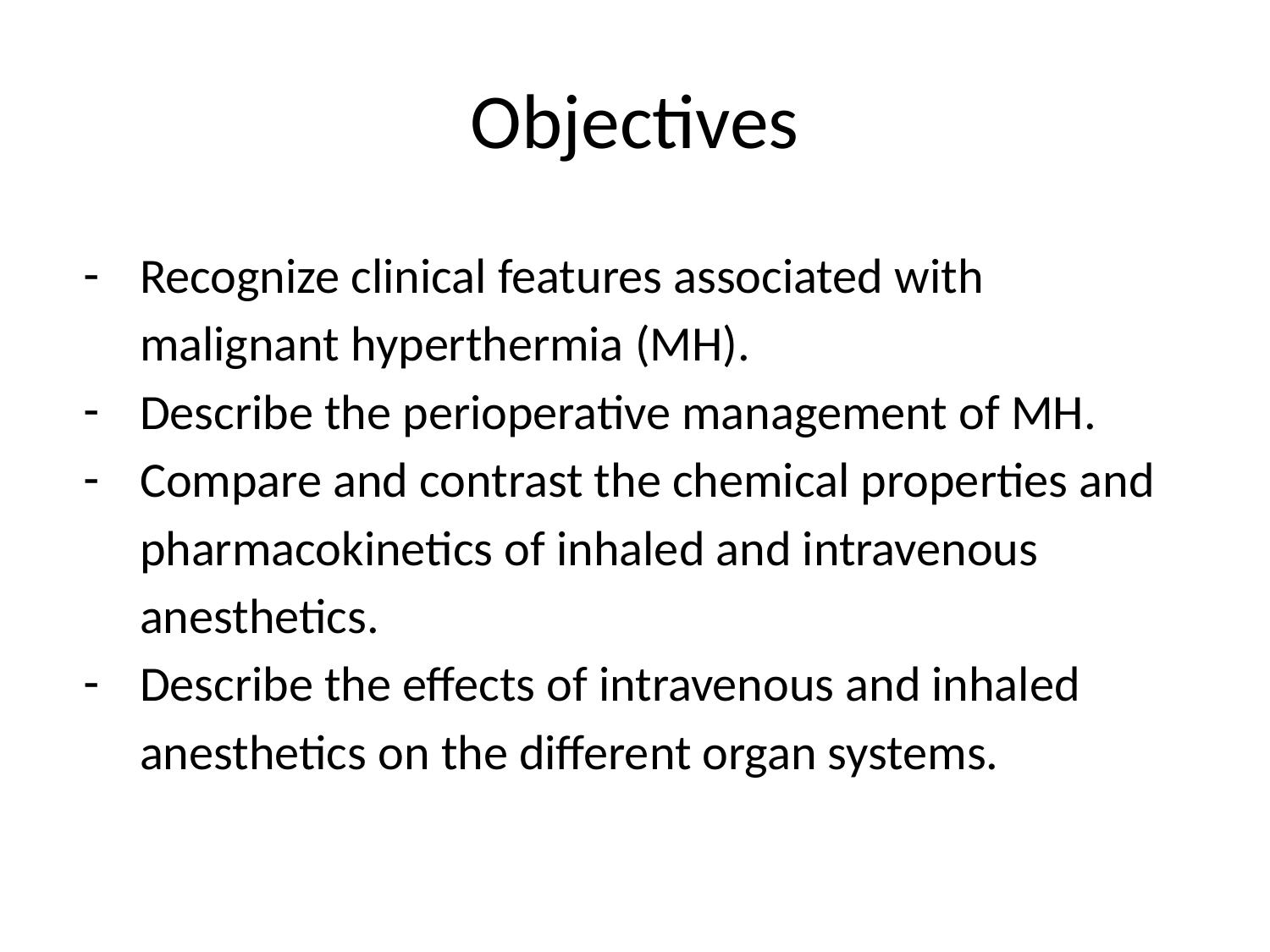

# Objectives
Recognize clinical features associated with malignant hyperthermia (MH).
Describe the perioperative management of MH.
Compare and contrast the chemical properties and pharmacokinetics of inhaled and intravenous anesthetics.
Describe the effects of intravenous and inhaled anesthetics on the different organ systems.

## Slide 3
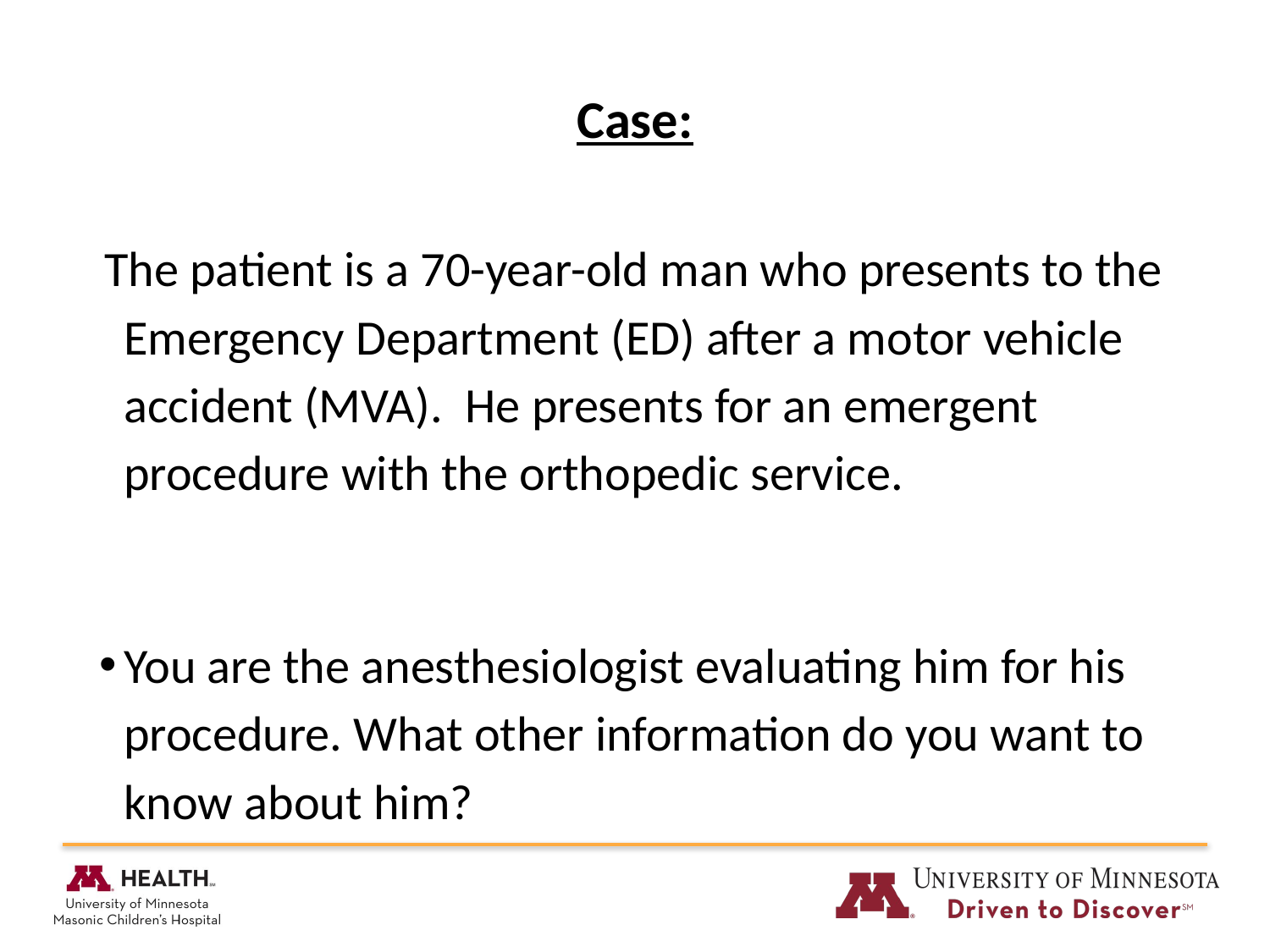

# Case:
The patient is a 70-year-old man who presents to the Emergency Department (ED) after a motor vehicle accident (MVA). He presents for an emergent procedure with the orthopedic service.
You are the anesthesiologist evaluating him for his procedure. What other information do you want to know about him?

## Slide 4
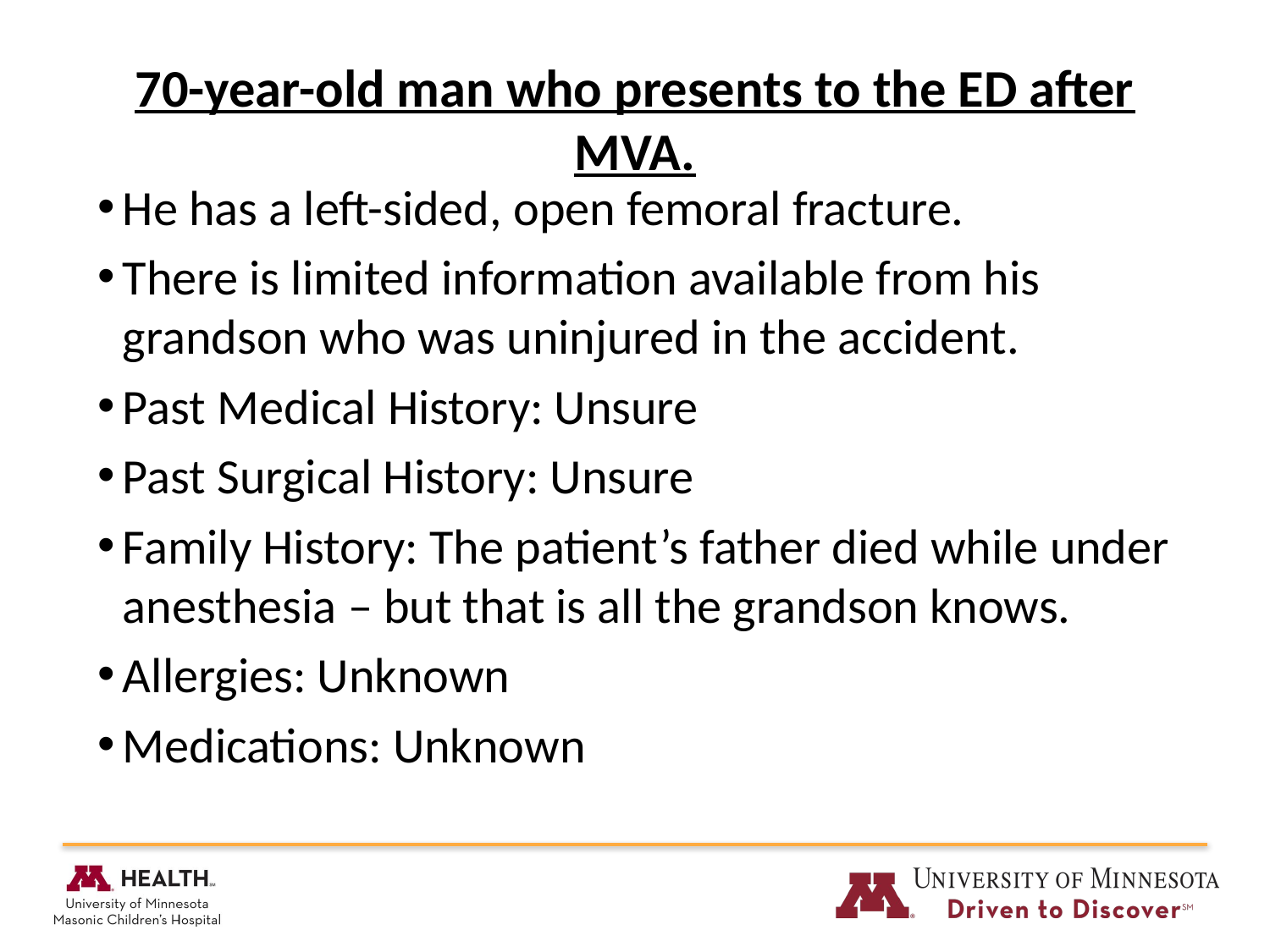

# 70-year-old man who presents to the ED after MVA.
He has a left-sided, open femoral fracture.
There is limited information available from his grandson who was uninjured in the accident.
Past Medical History: Unsure
Past Surgical History: Unsure
Family History: The patient’s father died while under anesthesia – but that is all the grandson knows.
Allergies: Unknown
Medications: Unknown

## Slide 5
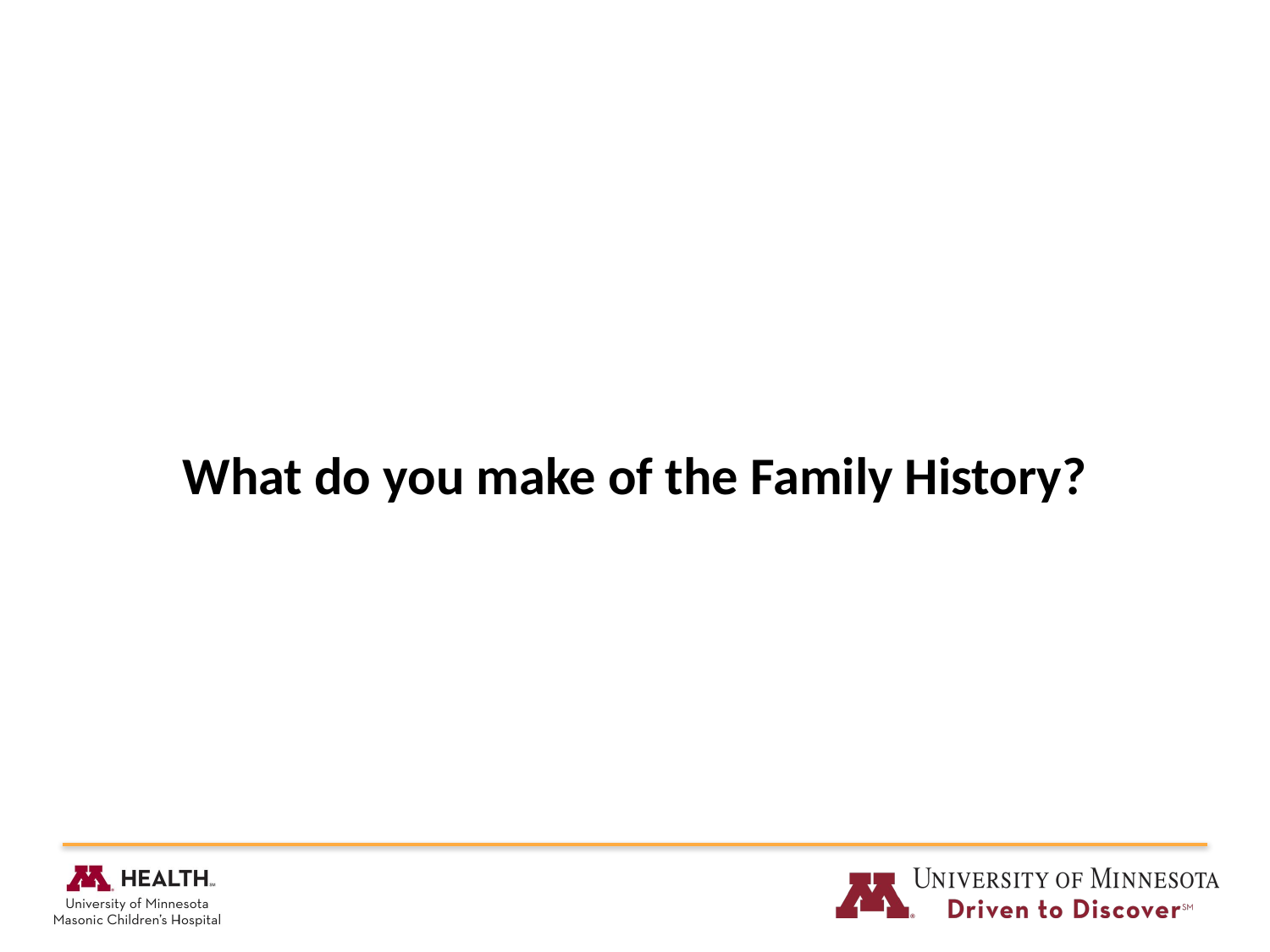

# What do you make of the Family History?

## Slide 6
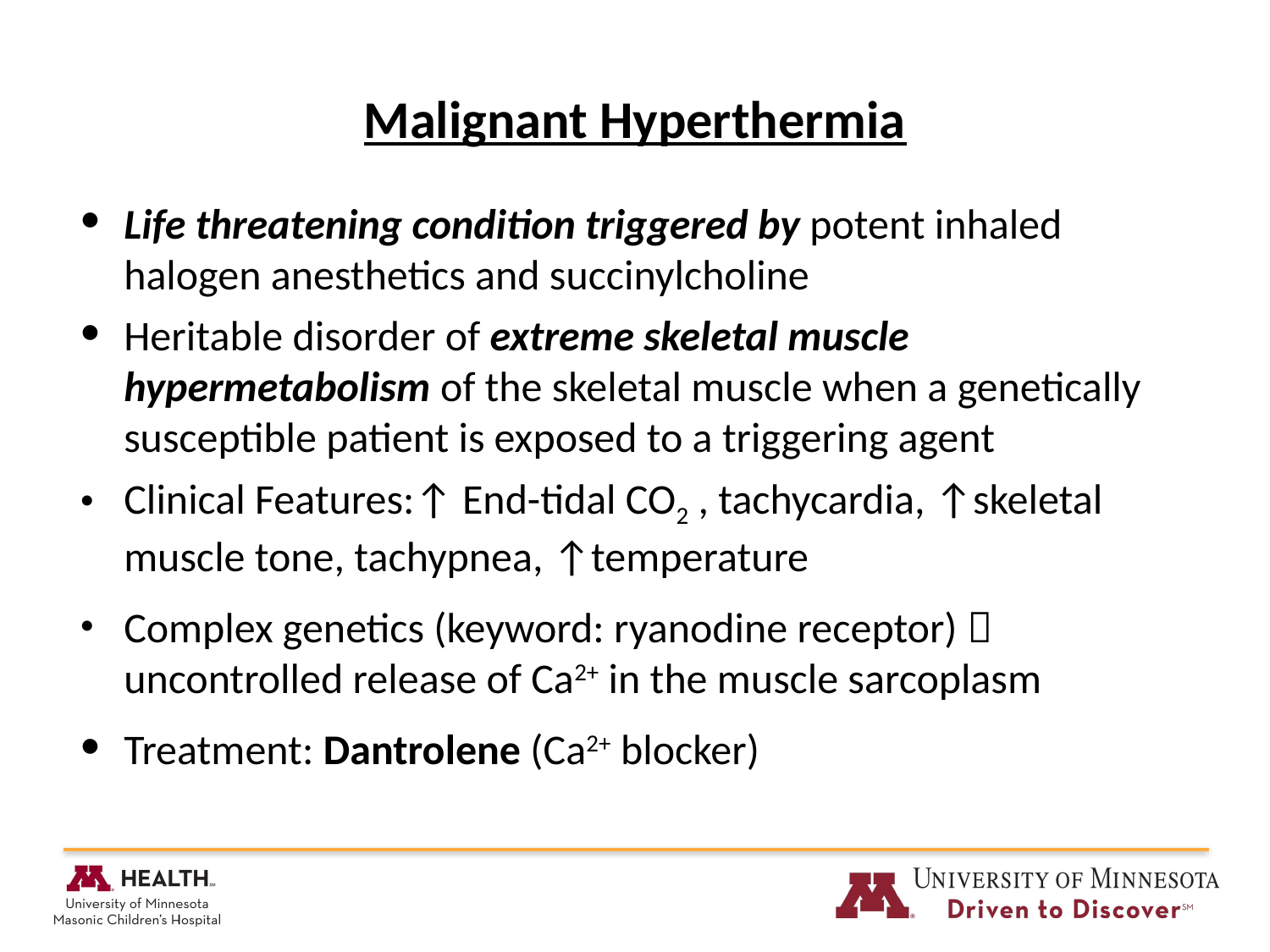

# Malignant Hyperthermia
Life threatening condition triggered by potent inhaled halogen anesthetics and succinylcholine
Heritable disorder of extreme skeletal muscle hypermetabolism of the skeletal muscle when a genetically susceptible patient is exposed to a triggering agent
Clinical Features:↑ End-tidal CO2 , tachycardia, ↑skeletal muscle tone, tachypnea, ↑temperature
Complex genetics (keyword: ryanodine receptor)  uncontrolled release of Ca2+ in the muscle sarcoplasm
Treatment: Dantrolene (Ca2+ blocker)

## Slide 7
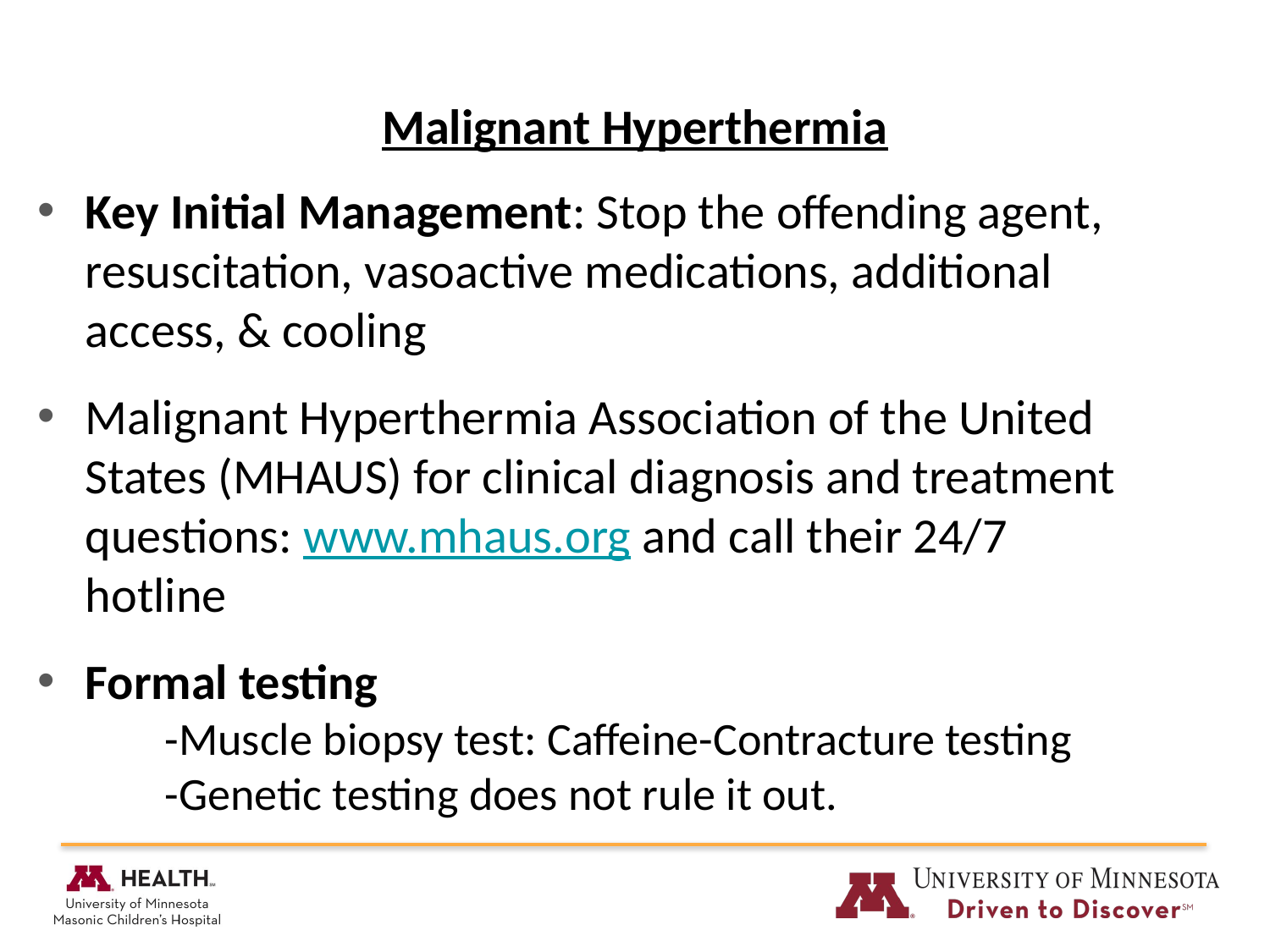

# Malignant Hyperthermia
Key Initial Management: Stop the offending agent, resuscitation, vasoactive medications, additional access, & cooling
Malignant Hyperthermia Association of the United States (MHAUS) for clinical diagnosis and treatment questions: www.mhaus.org and call their 24/7 hotline
Formal testing
	-Muscle biopsy test: Caffeine-Contracture testing
	-Genetic testing does not rule it out.

## Slide 8
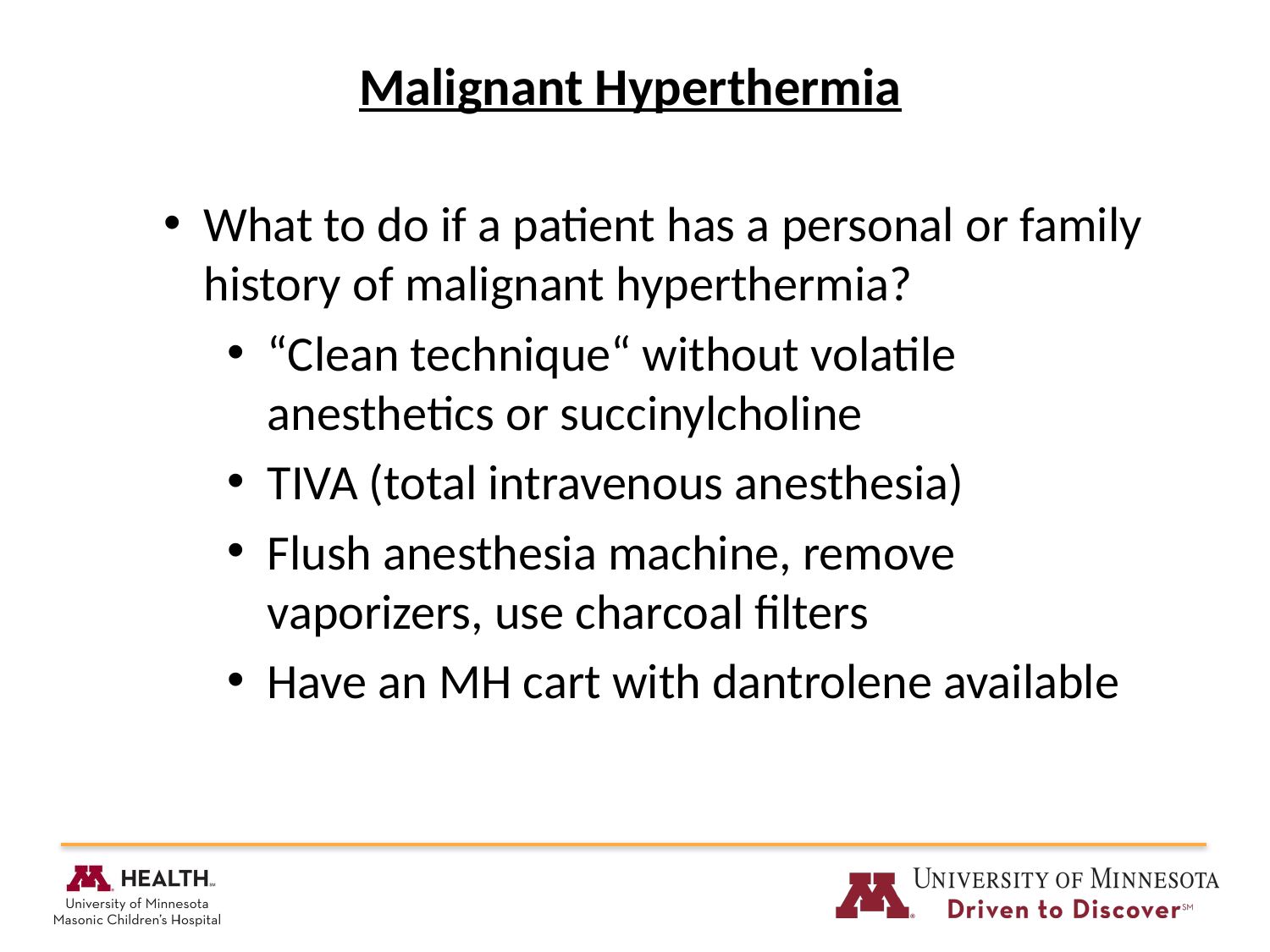

Malignant Hyperthermia
What to do if a patient has a personal or family history of malignant hyperthermia?
“Clean technique“ without volatile anesthetics or succinylcholine
TIVA (total intravenous anesthesia)
Flush anesthesia machine, remove vaporizers, use charcoal filters
Have an MH cart with dantrolene available

## Slide 9
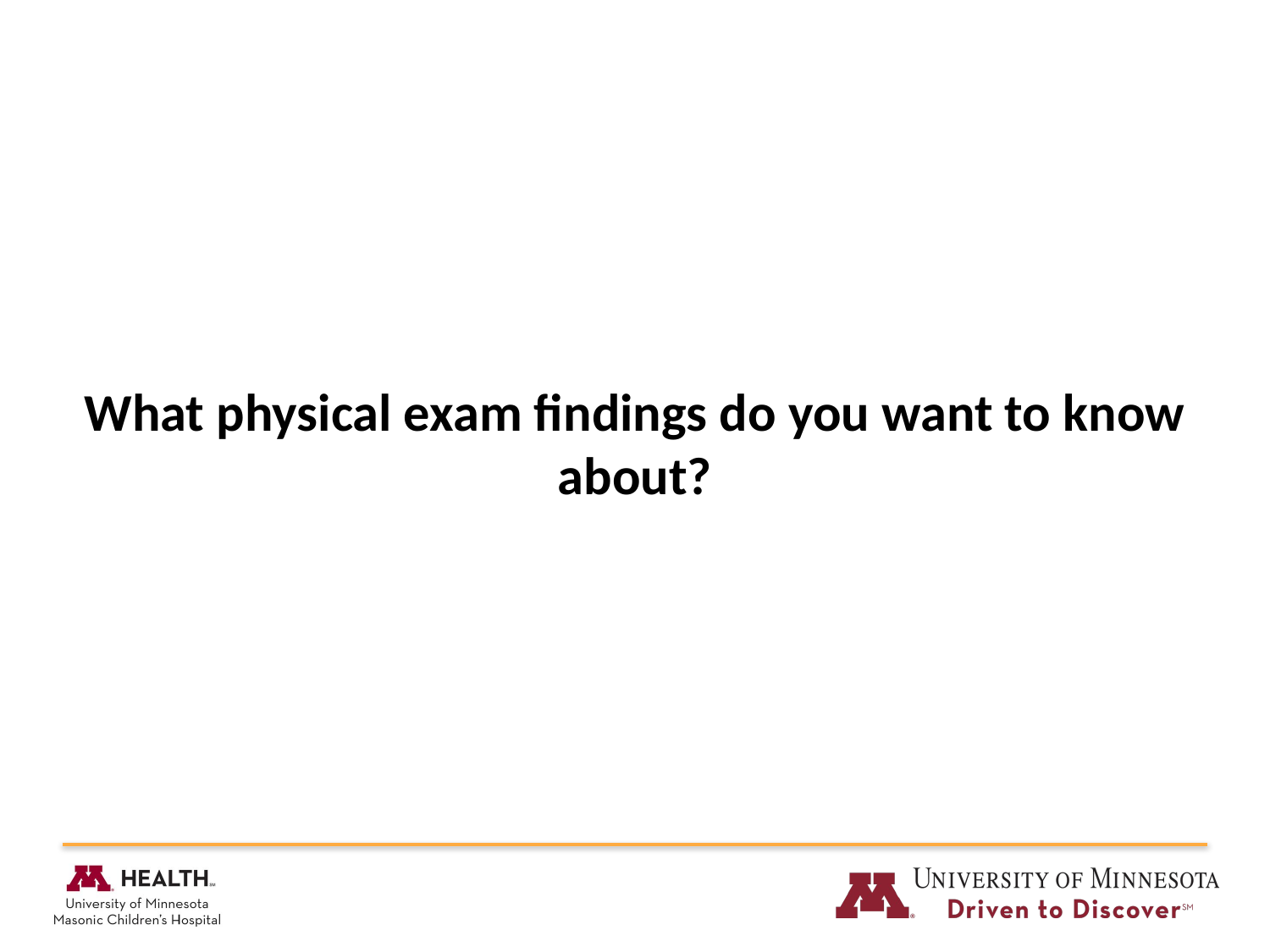

# What physical exam findings do you want to know about?

## Slide 10
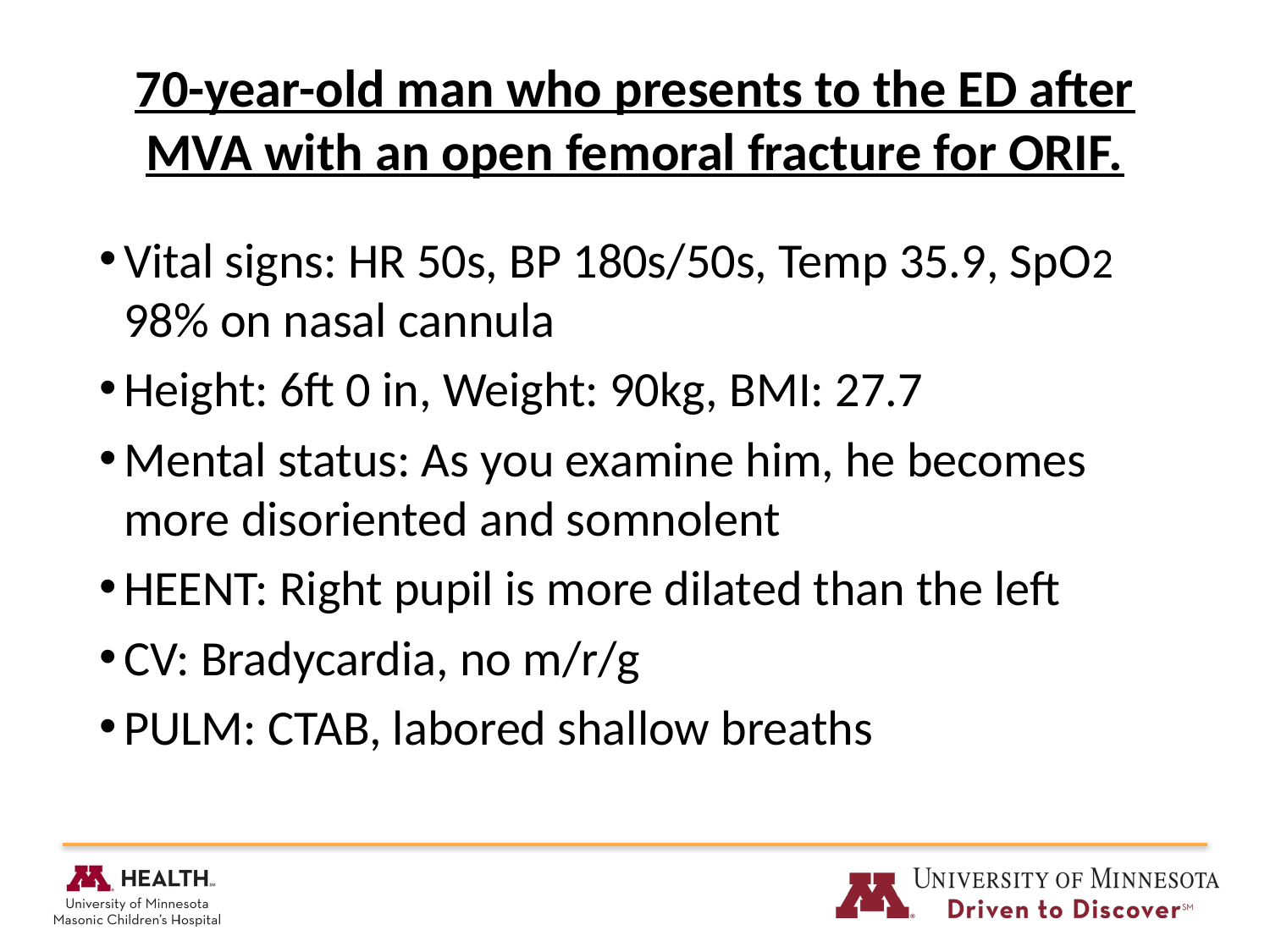

# 70-year-old man who presents to the ED after MVA with an open femoral fracture for ORIF.
Vital signs: HR 50s, BP 180s/50s, Temp 35.9, SpO2 98% on nasal cannula
Height: 6ft 0 in, Weight: 90kg, BMI: 27.7
Mental status: As you examine him, he becomes more disoriented and somnolent
HEENT: Right pupil is more dilated than the left
CV: Bradycardia, no m/r/g
PULM: CTAB, labored shallow breaths

## Slide 11
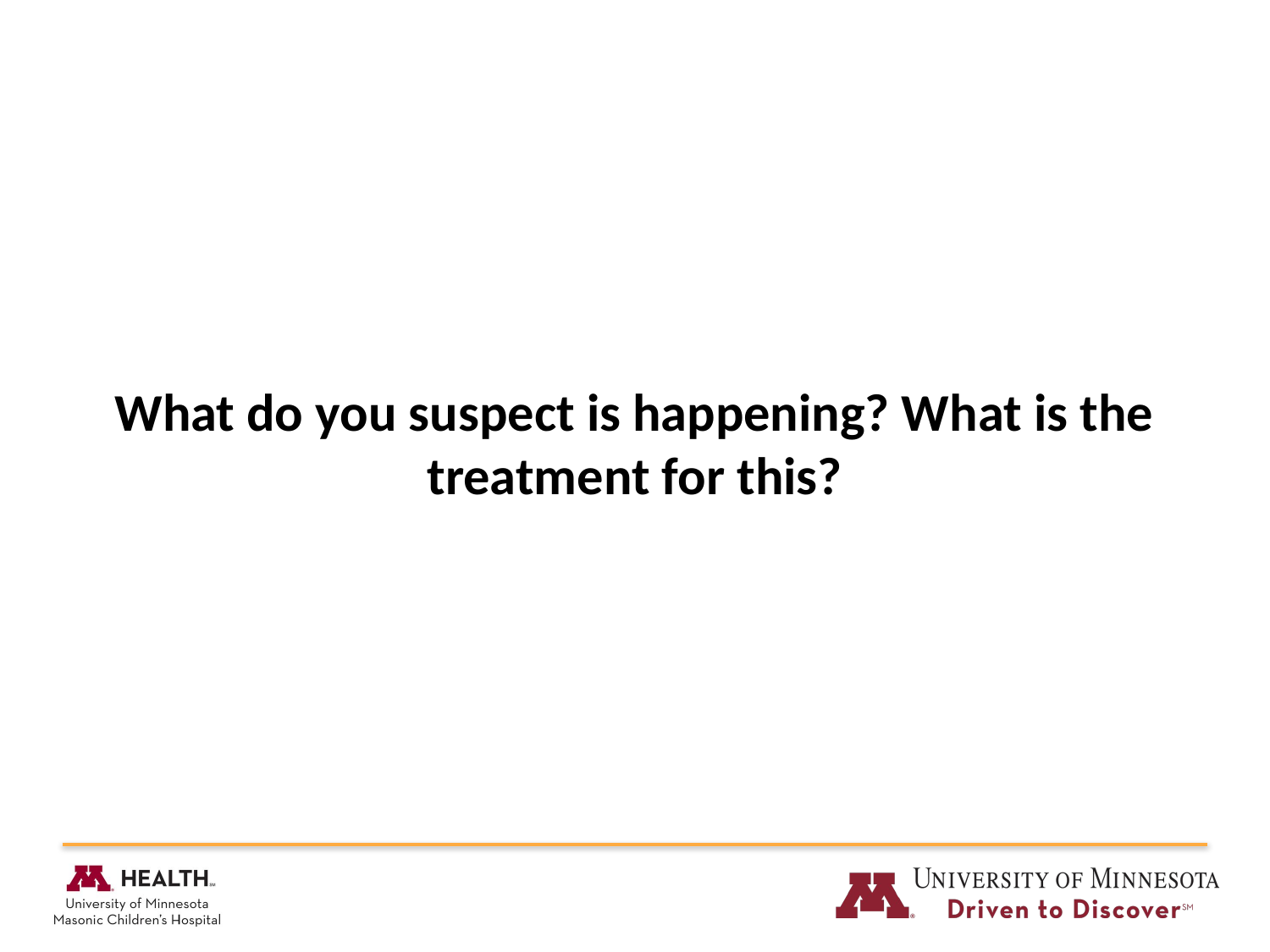

# What do you suspect is happening? What is the treatment for this?

## Slide 12
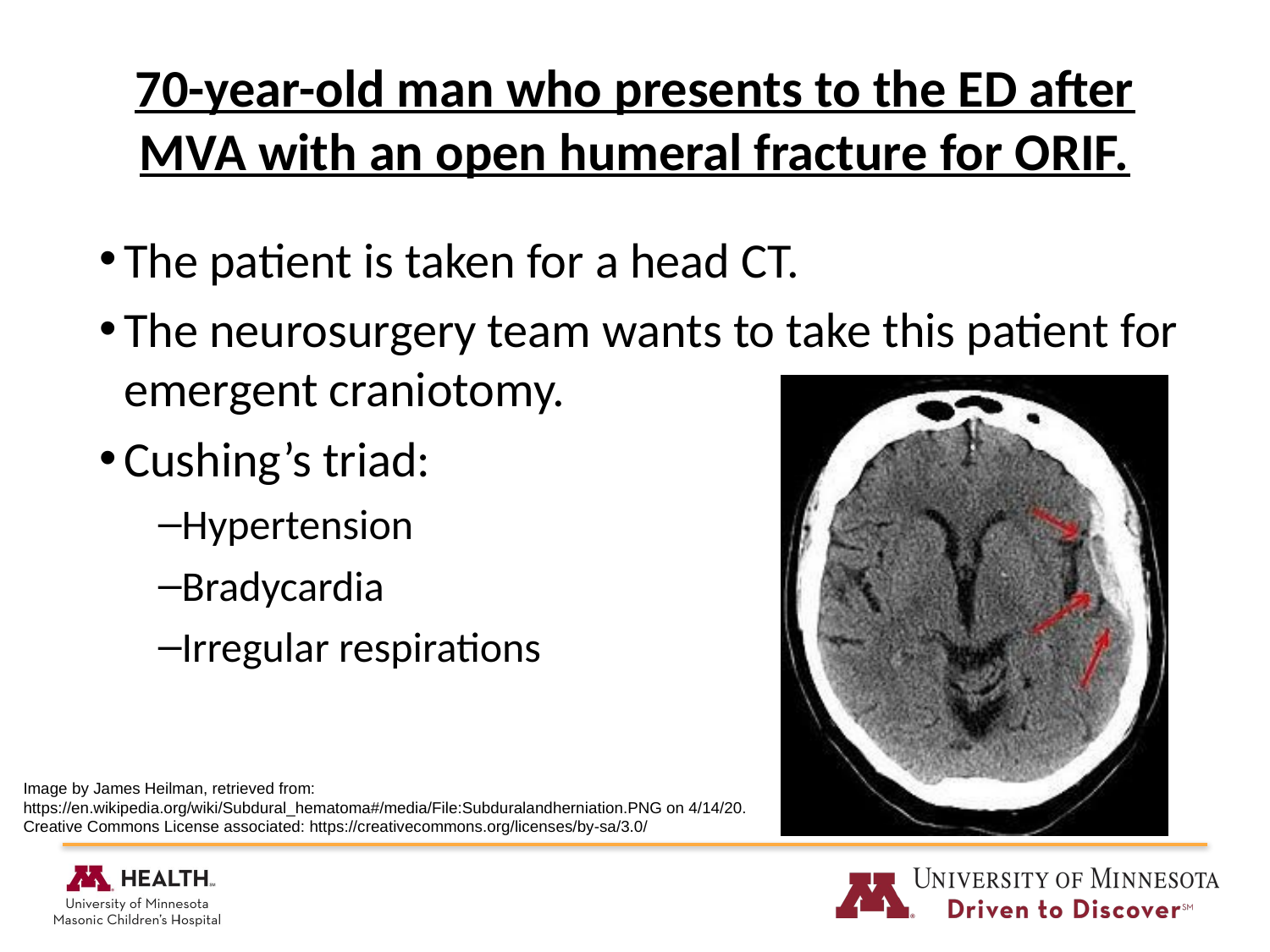

# 70-year-old man who presents to the ED after MVA with an open humeral fracture for ORIF.
The patient is taken for a head CT.
The neurosurgery team wants to take this patient for emergent craniotomy.
Cushing’s triad:
Hypertension
Bradycardia
Irregular respirations
Image by James Heilman, retrieved from:
https://en.wikipedia.org/wiki/Subdural_hematoma#/media/File:Subduralandherniation.PNG on 4/14/20. Creative Commons License associated: https://creativecommons.org/licenses/by-sa/3.0/

## Slide 13
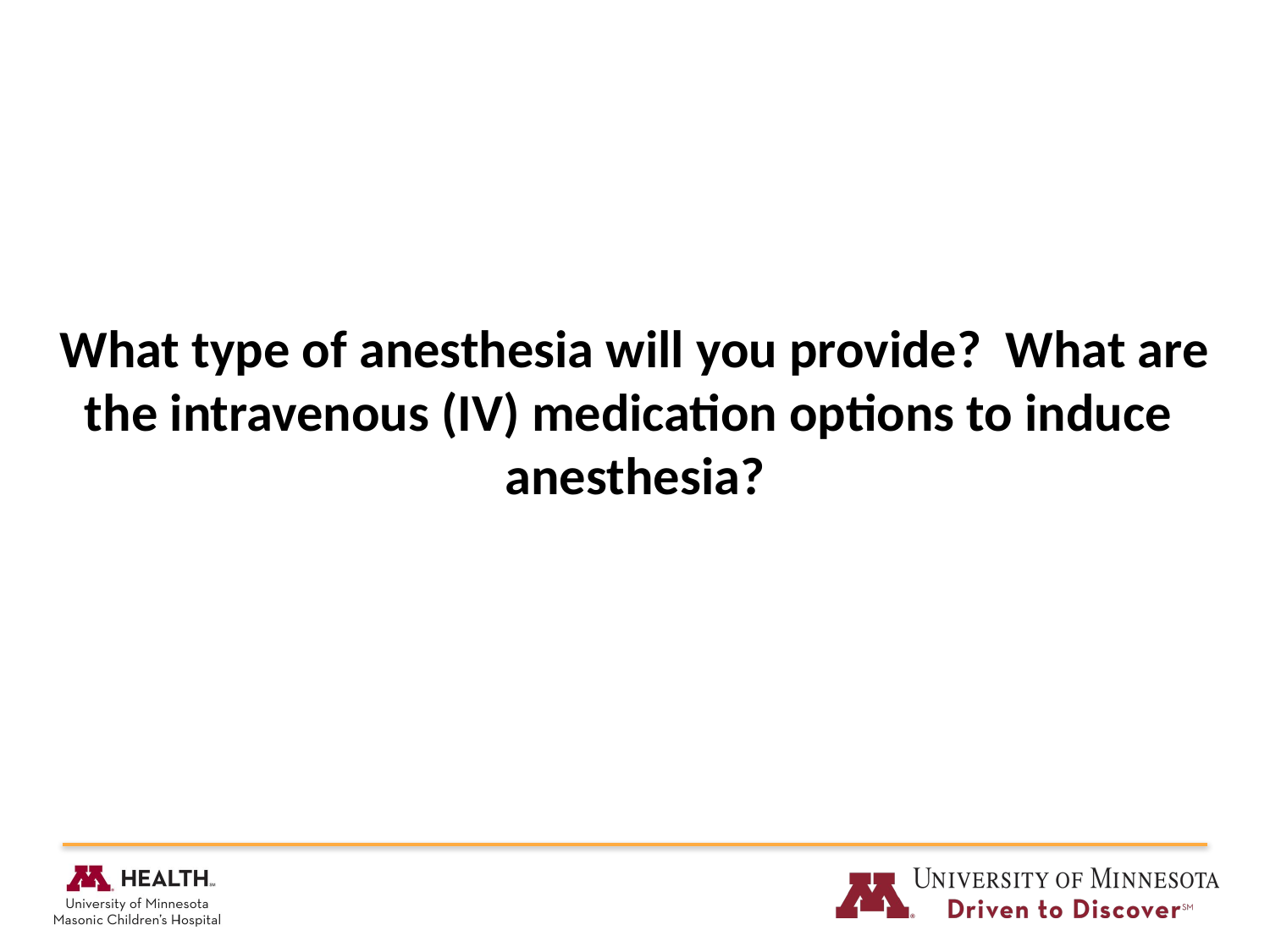

# What type of anesthesia will you provide? What are the intravenous (IV) medication options to induce anesthesia?

## Slide 14
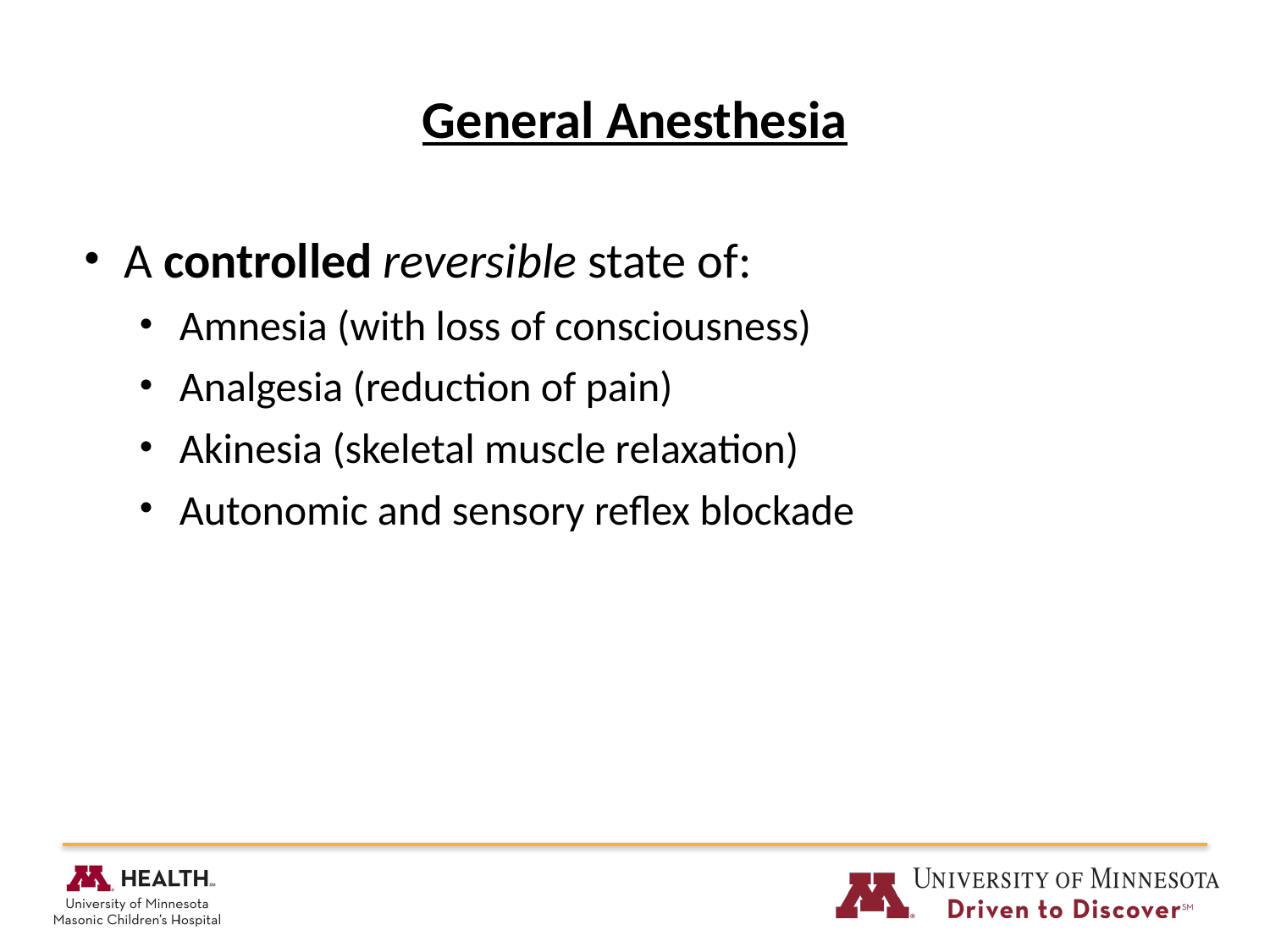

# General Anesthesia
A controlled reversible state of:
Amnesia (with loss of consciousness)
Analgesia (reduction of pain)
Akinesia (skeletal muscle relaxation)
Autonomic and sensory reflex blockade

## Slide 15
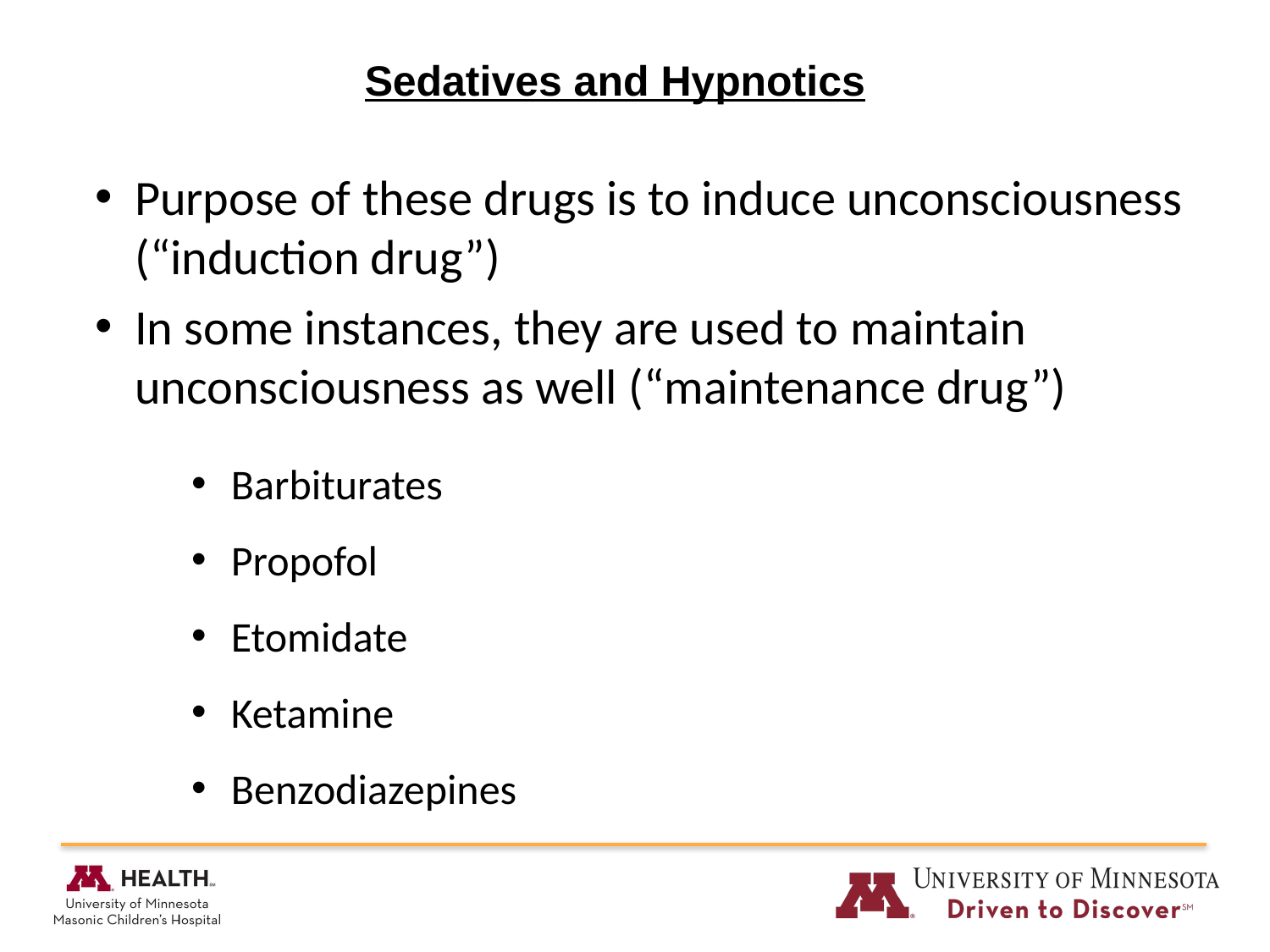

Sedatives and Hypnotics
Purpose of these drugs is to induce unconsciousness (“induction drug”)
In some instances, they are used to maintain unconsciousness as well (“maintenance drug”)
Barbiturates
Propofol
Etomidate
Ketamine
Benzodiazepines

## Slide 16
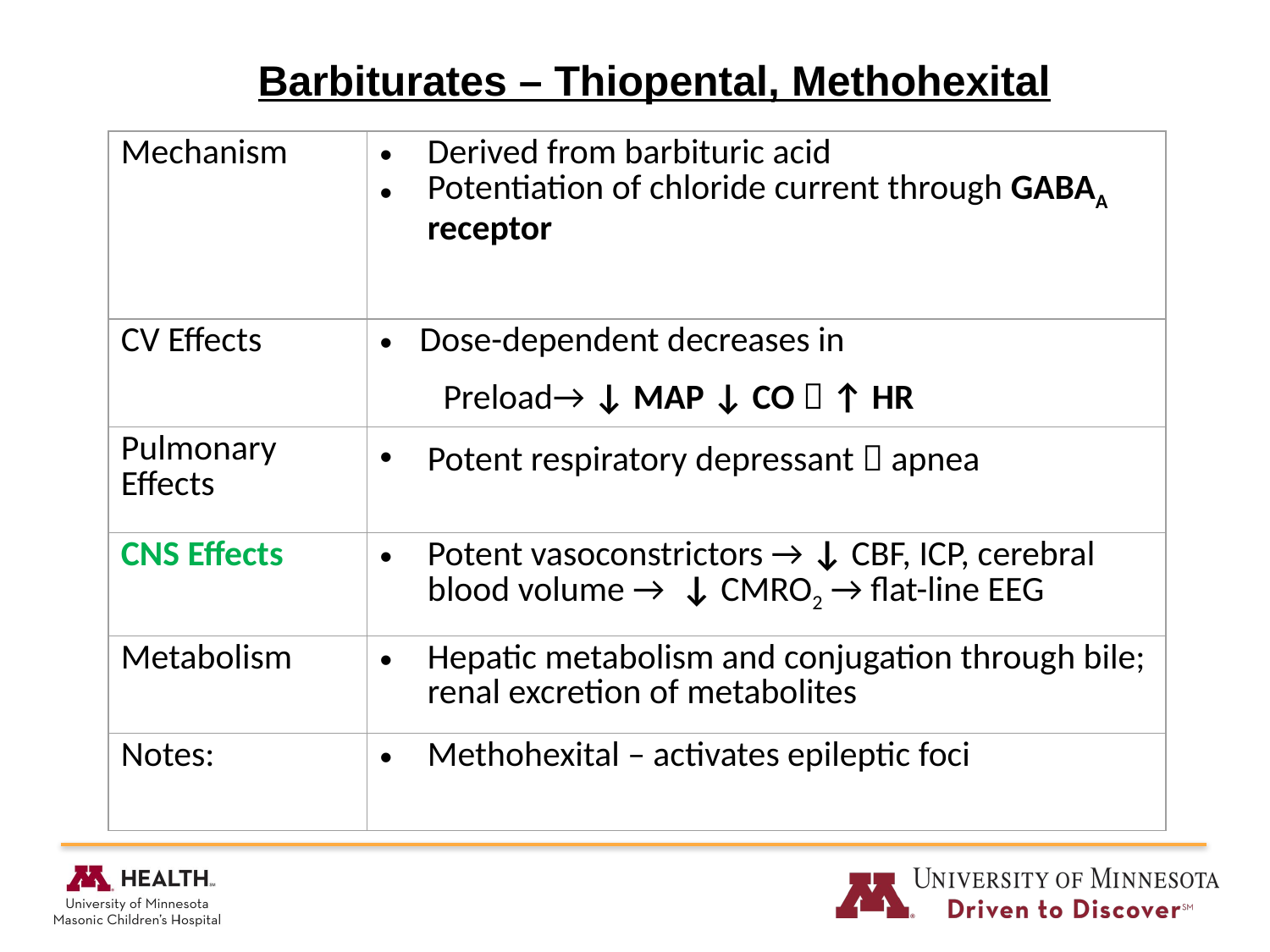

Barbiturates – Thiopental, Methohexital
| Mechanism | Derived from barbituric acid Potentiation of chloride current through GABAA receptor |
| --- | --- |
| CV Effects | Dose-dependent decreases in Preload→ ↓ MAP ↓ CO  ↑ HR |
| Pulmonary Effects | Potent respiratory depressant  apnea |
| CNS Effects | Potent vasoconstrictors → ↓ CBF, ICP, cerebral blood volume → ↓ CMRO2 → flat-line EEG |
| Metabolism | Hepatic metabolism and conjugation through bile; renal excretion of metabolites |
| Notes: | Methohexital – activates epileptic foci |

## Slide 17
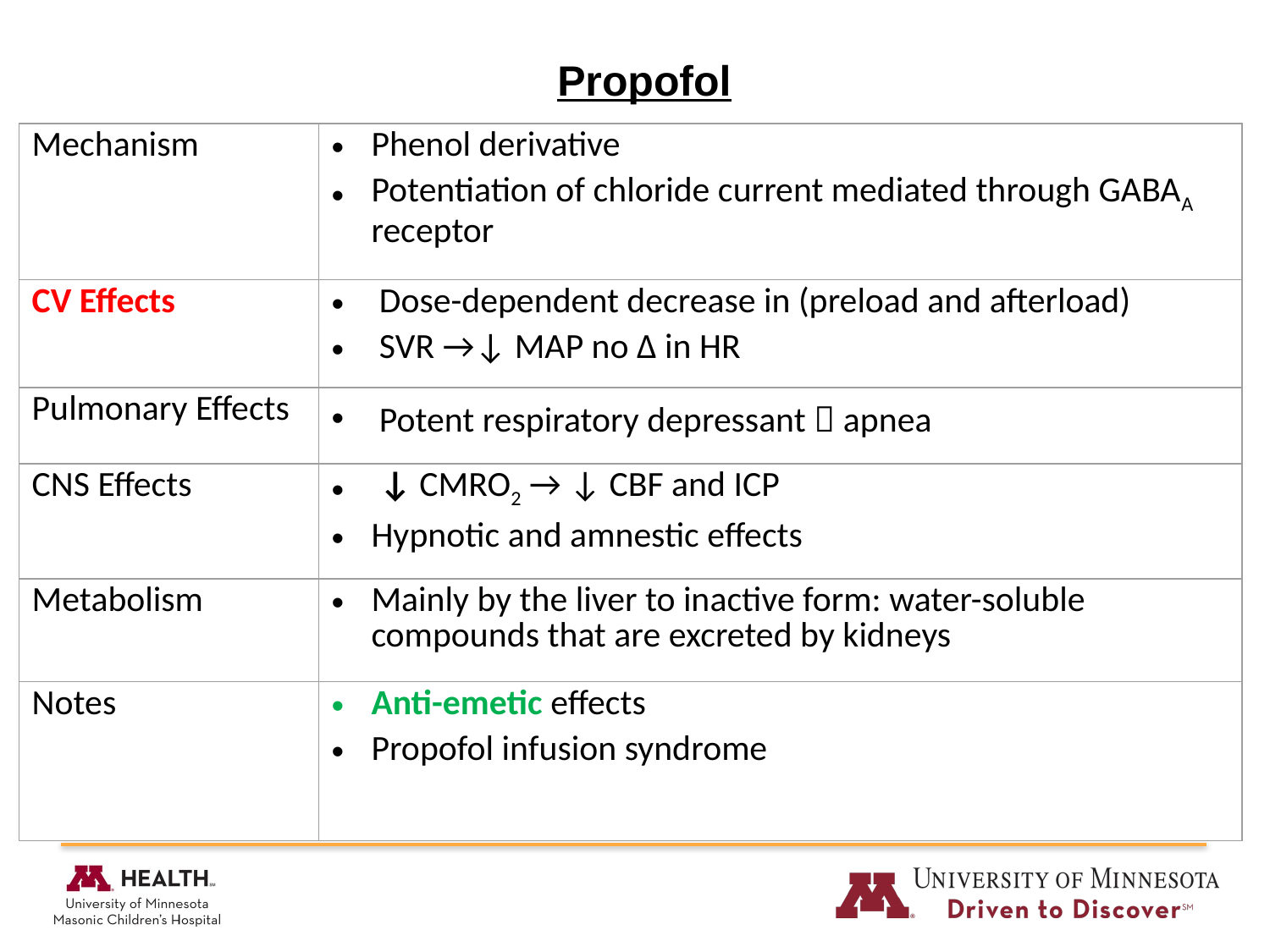

Propofol
| Mechanism | Phenol derivative Potentiation of chloride current mediated through GABAA receptor |
| --- | --- |
| CV Effects | Dose-dependent decrease in (preload and afterload) SVR →↓ MAP no ∆ in HR |
| Pulmonary Effects | Potent respiratory depressant  apnea |
| CNS Effects | ↓ CMRO2 → ↓ CBF and ICP Hypnotic and amnestic effects |
| Metabolism | Mainly by the liver to inactive form: water-soluble compounds that are excreted by kidneys |
| Notes | Anti-emetic effects Propofol infusion syndrome |

## Slide 18
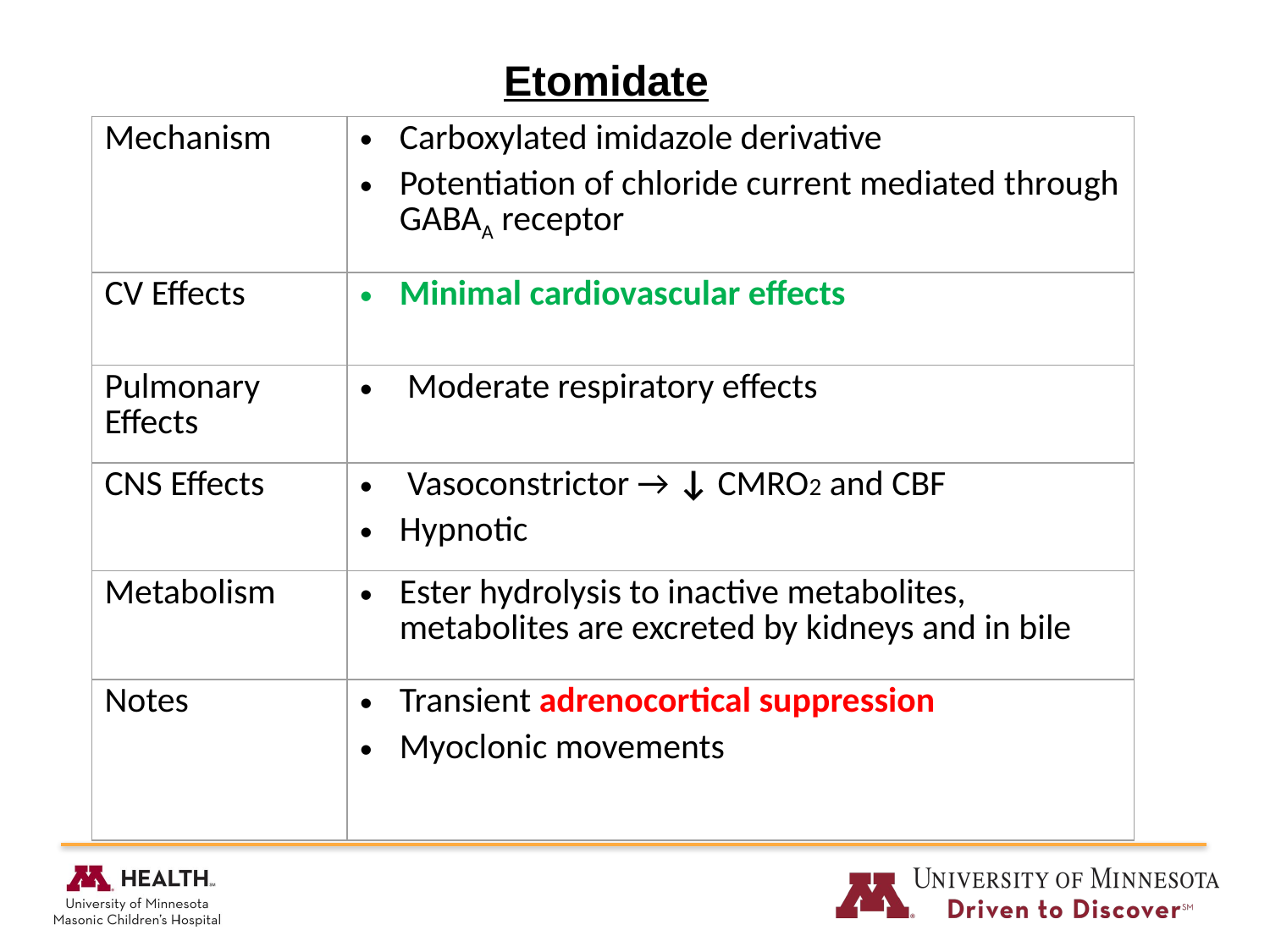

Etomidate
| Mechanism | Carboxylated imidazole derivative Potentiation of chloride current mediated through GABAA receptor |
| --- | --- |
| CV Effects | Minimal cardiovascular effects |
| Pulmonary Effects | Moderate respiratory effects |
| CNS Effects | Vasoconstrictor → ↓ CMRO2 and CBF Hypnotic |
| Metabolism | Ester hydrolysis to inactive metabolites, metabolites are excreted by kidneys and in bile |
| Notes | Transient adrenocortical suppression Myoclonic movements |

## Slide 19
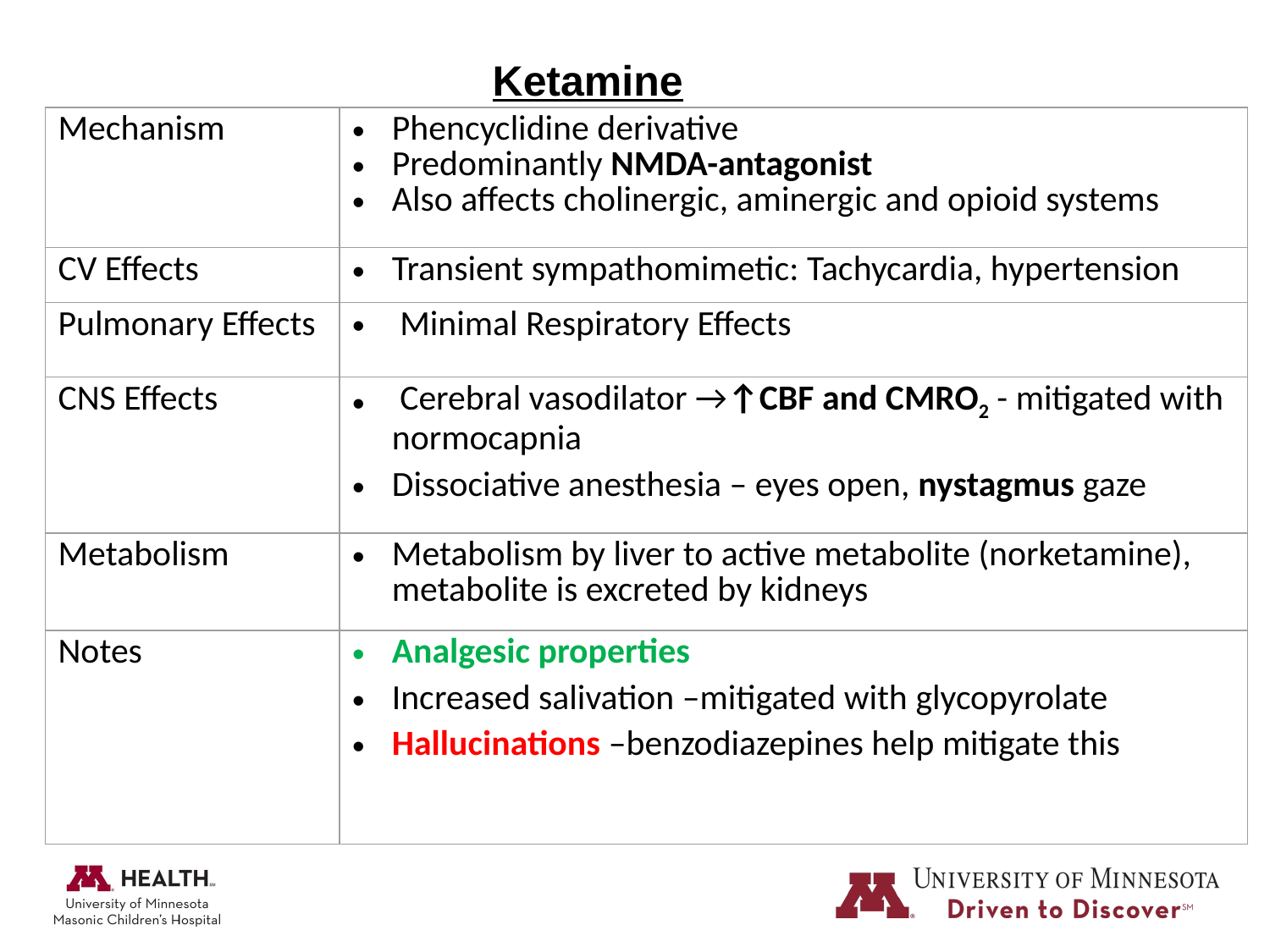

Ketamine
| Mechanism | Phencyclidine derivative Predominantly NMDA-antagonist Also affects cholinergic, aminergic and opioid systems |
| --- | --- |
| CV Effects | Transient sympathomimetic: Tachycardia, hypertension |
| Pulmonary Effects | Minimal Respiratory Effects |
| CNS Effects | Cerebral vasodilator →↑CBF and CMRO2 - mitigated with normocapnia Dissociative anesthesia – eyes open, nystagmus gaze |
| Metabolism | Metabolism by liver to active metabolite (norketamine), metabolite is excreted by kidneys |
| Notes | Analgesic properties Increased salivation –mitigated with glycopyrolate Hallucinations –benzodiazepines help mitigate this |

## Slide 20
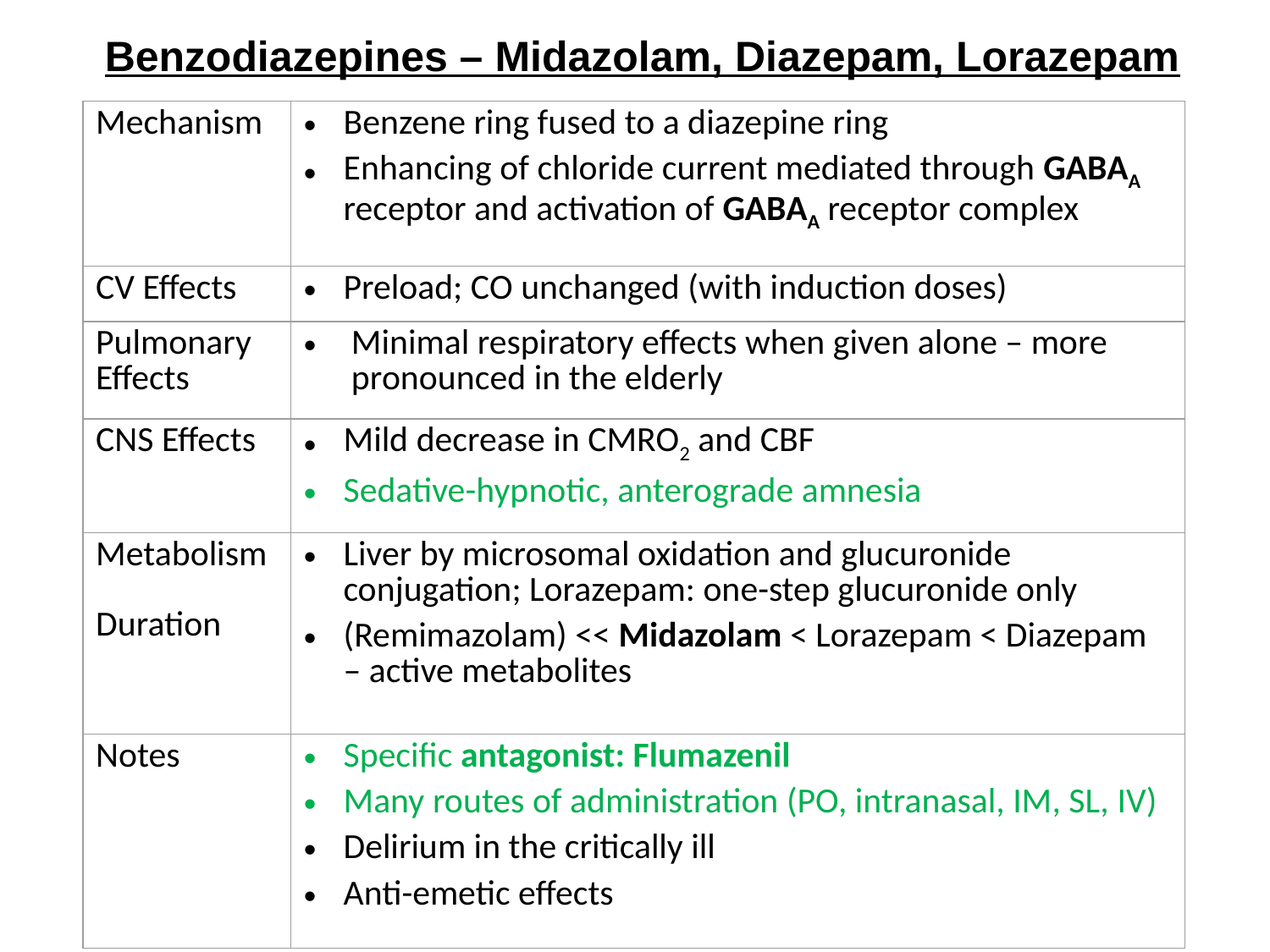

Benzodiazepines – Midazolam, Diazepam, Lorazepam
| Mechanism | Benzene ring fused to a diazepine ring Enhancing of chloride current mediated through GABAA receptor and activation of GABAA receptor complex |
| --- | --- |
| CV Effects | Preload; CO unchanged (with induction doses) |
| Pulmonary Effects | Minimal respiratory effects when given alone – more pronounced in the elderly |
| CNS Effects | Mild decrease in CMRO2 and CBF Sedative-hypnotic, anterograde amnesia |
| Metabolism Duration | Liver by microsomal oxidation and glucuronide conjugation; Lorazepam: one-step glucuronide only (Remimazolam) << Midazolam < Lorazepam < Diazepam – active metabolites |
| Notes | Specific antagonist: Flumazenil Many routes of administration (PO, intranasal, IM, SL, IV) Delirium in the critically ill Anti-emetic effects |

## Slide 21
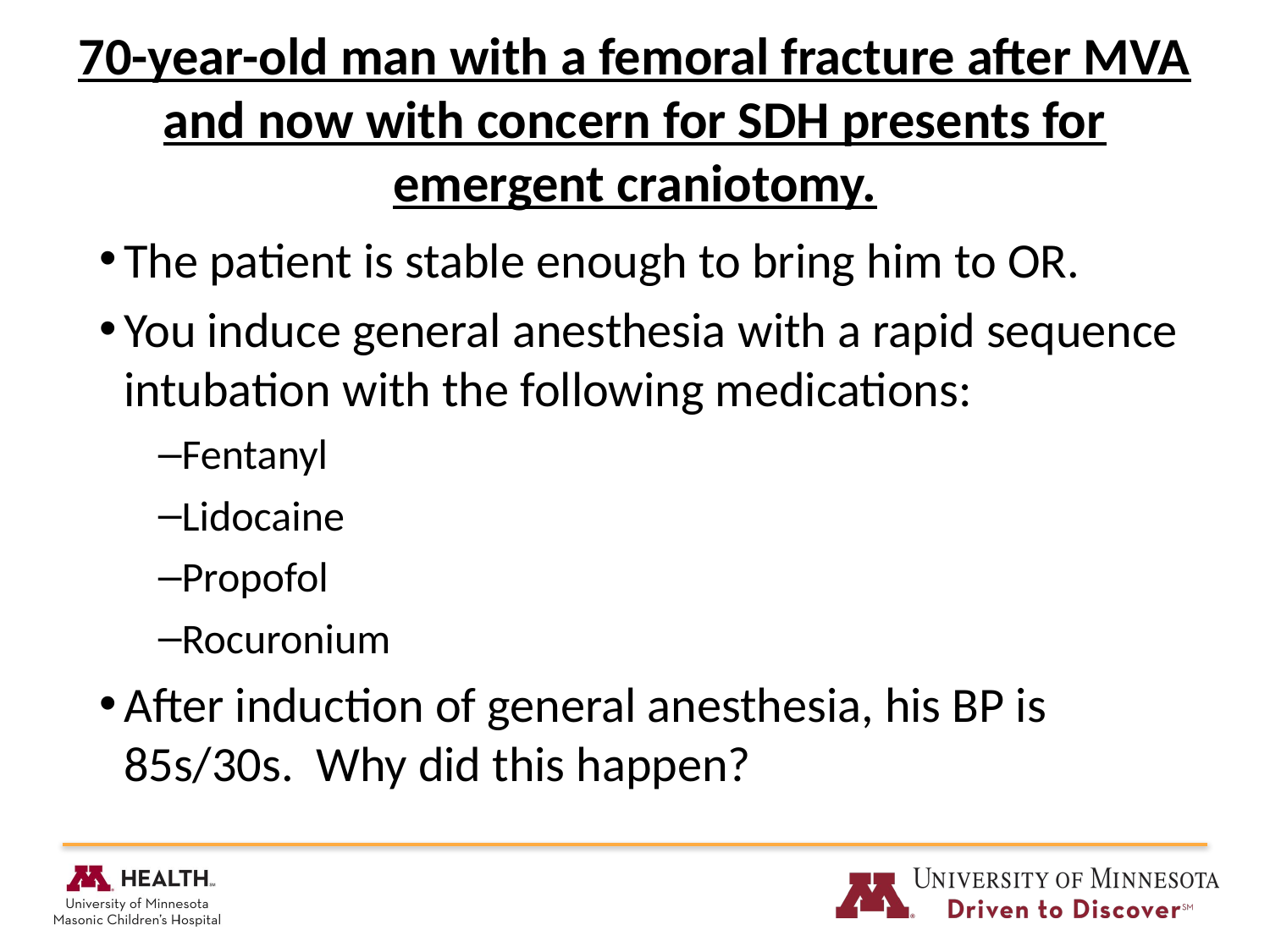

# 70-year-old man with a femoral fracture after MVA and now with concern for SDH presents for emergent craniotomy.
The patient is stable enough to bring him to OR.
You induce general anesthesia with a rapid sequence intubation with the following medications:
Fentanyl
Lidocaine
Propofol
Rocuronium
After induction of general anesthesia, his BP is 85s/30s. Why did this happen?

## Slide 22
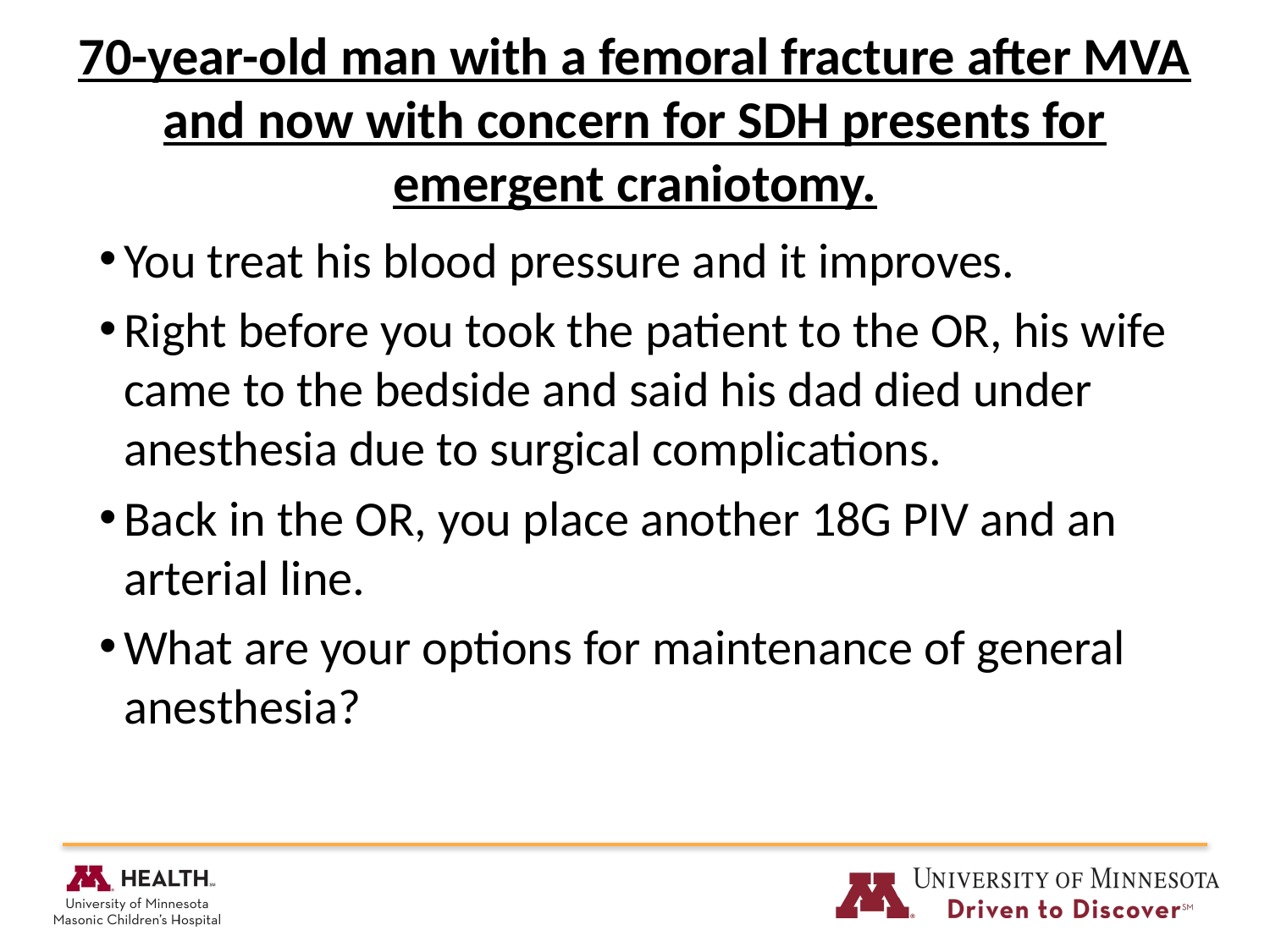

# 70-year-old man with a femoral fracture after MVA and now with concern for SDH presents for emergent craniotomy.
You treat his blood pressure and it improves.
Right before you took the patient to the OR, his wife came to the bedside and said his dad died under anesthesia due to surgical complications.
Back in the OR, you place another 18G PIV and an arterial line.
What are your options for maintenance of general anesthesia?

## Slide 23
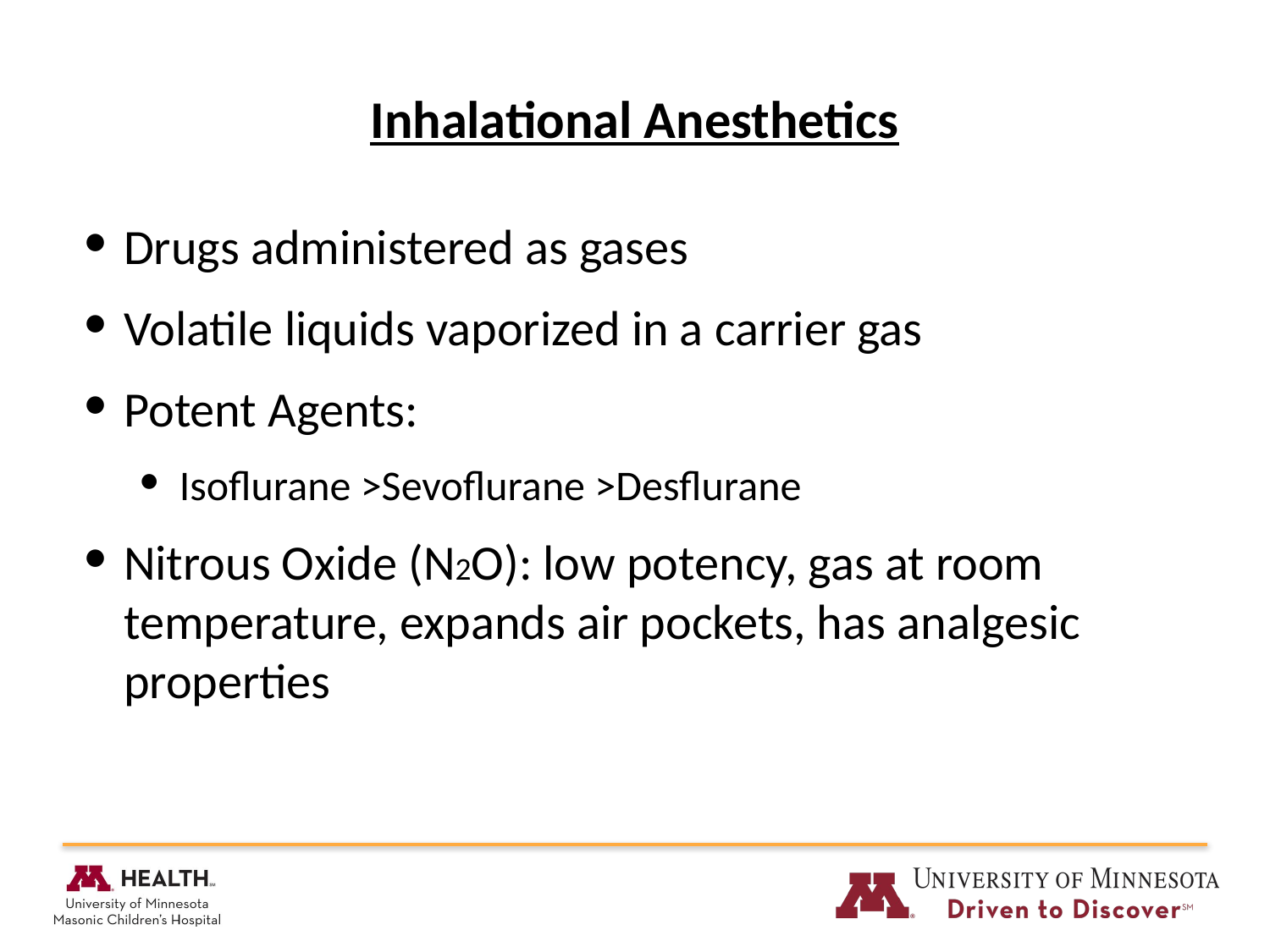

# Inhalational Anesthetics
Drugs administered as gases
Volatile liquids vaporized in a carrier gas
Potent Agents:
Isoflurane >Sevoflurane >Desflurane
Nitrous Oxide (N2O): low potency, gas at room temperature, expands air pockets, has analgesic properties
 adjunct

## Slide 24
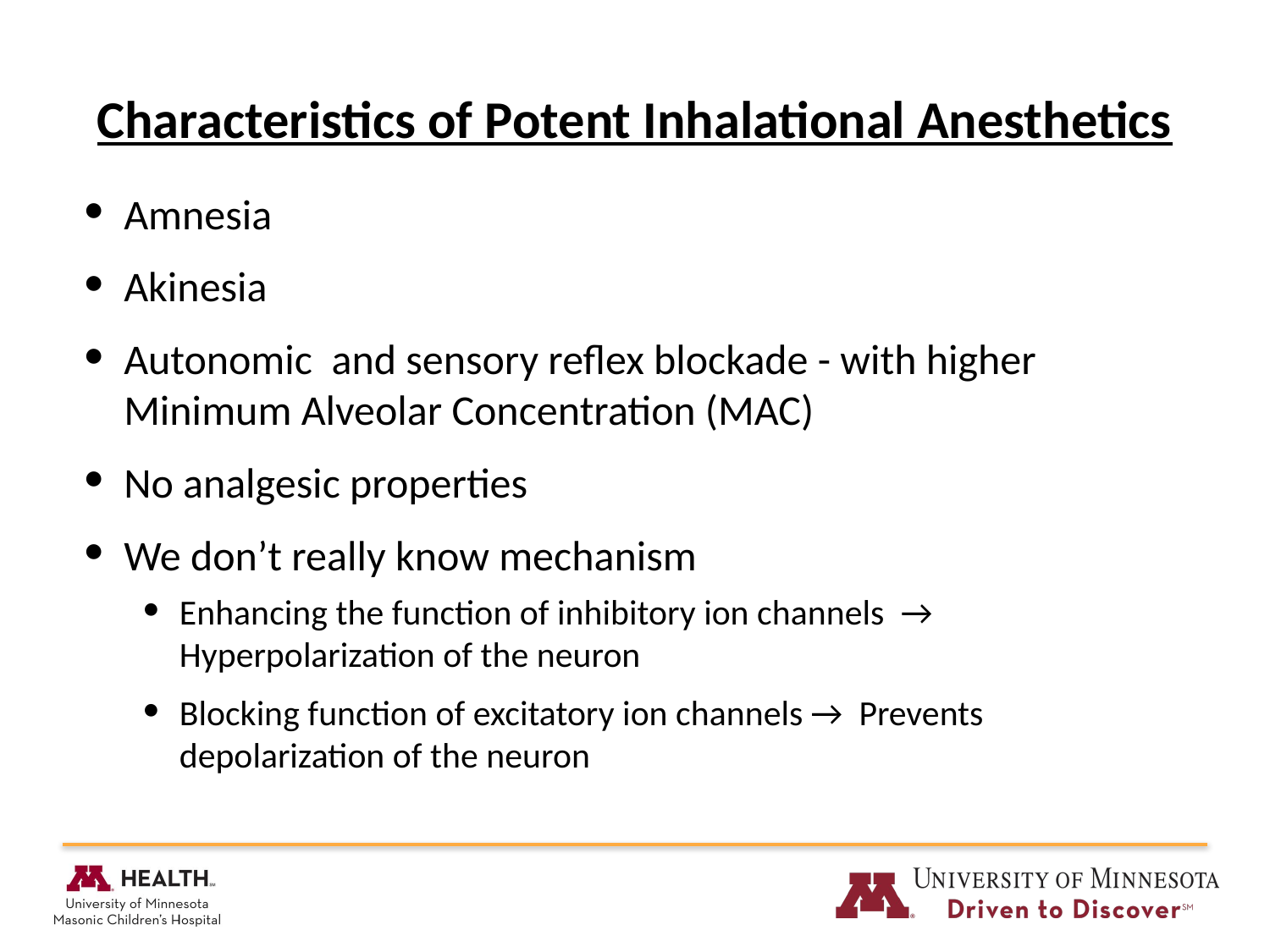

# Characteristics of Potent Inhalational Anesthetics
Amnesia
Akinesia
Autonomic and sensory reflex blockade - with higher Minimum Alveolar Concentration (MAC)
No analgesic properties
We don’t really know mechanism
Enhancing the function of inhibitory ion channels → Hyperpolarization of the neuron
Blocking function of excitatory ion channels → Prevents depolarization of the neuron

## Slide 25
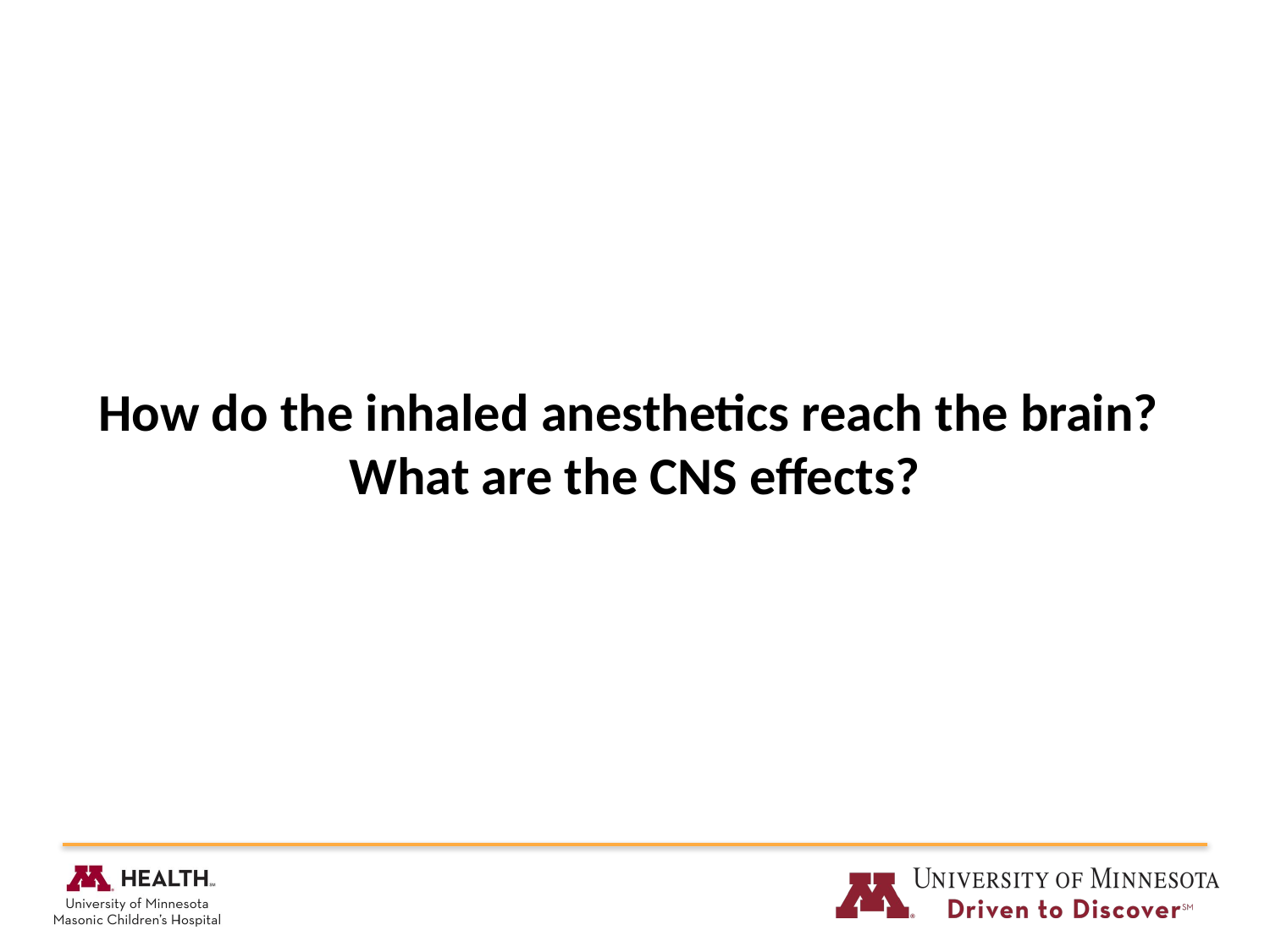

# How do the inhaled anesthetics reach the brain? What are the CNS effects?

## Slide 26
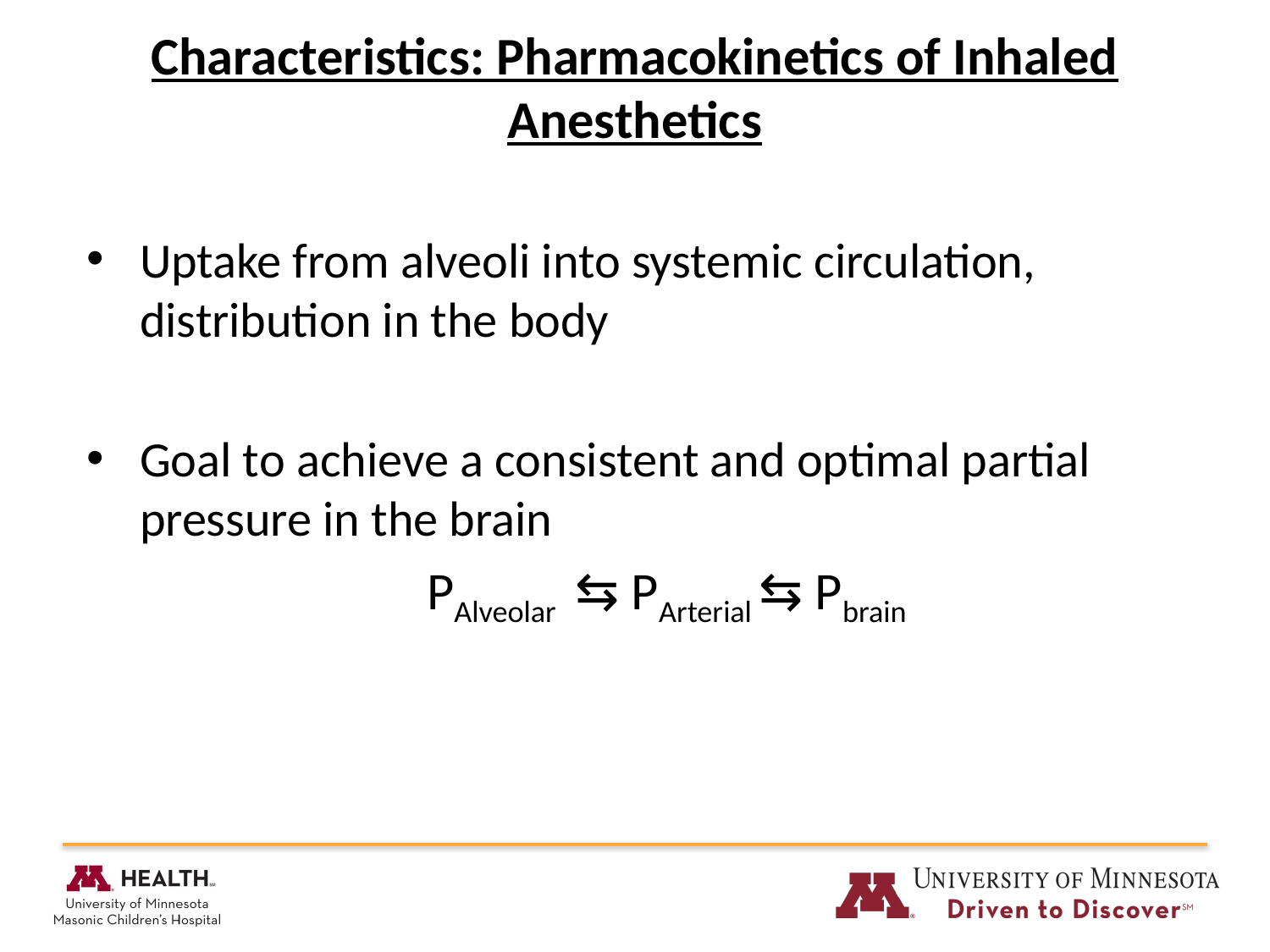

# Characteristics: Pharmacokinetics of Inhaled Anesthetics
Uptake from alveoli into systemic circulation, distribution in the body
Goal to achieve a consistent and optimal partial pressure in the brain
PAlveolar ⇆ PArterial ⇆ Pbrain

## Slide 27
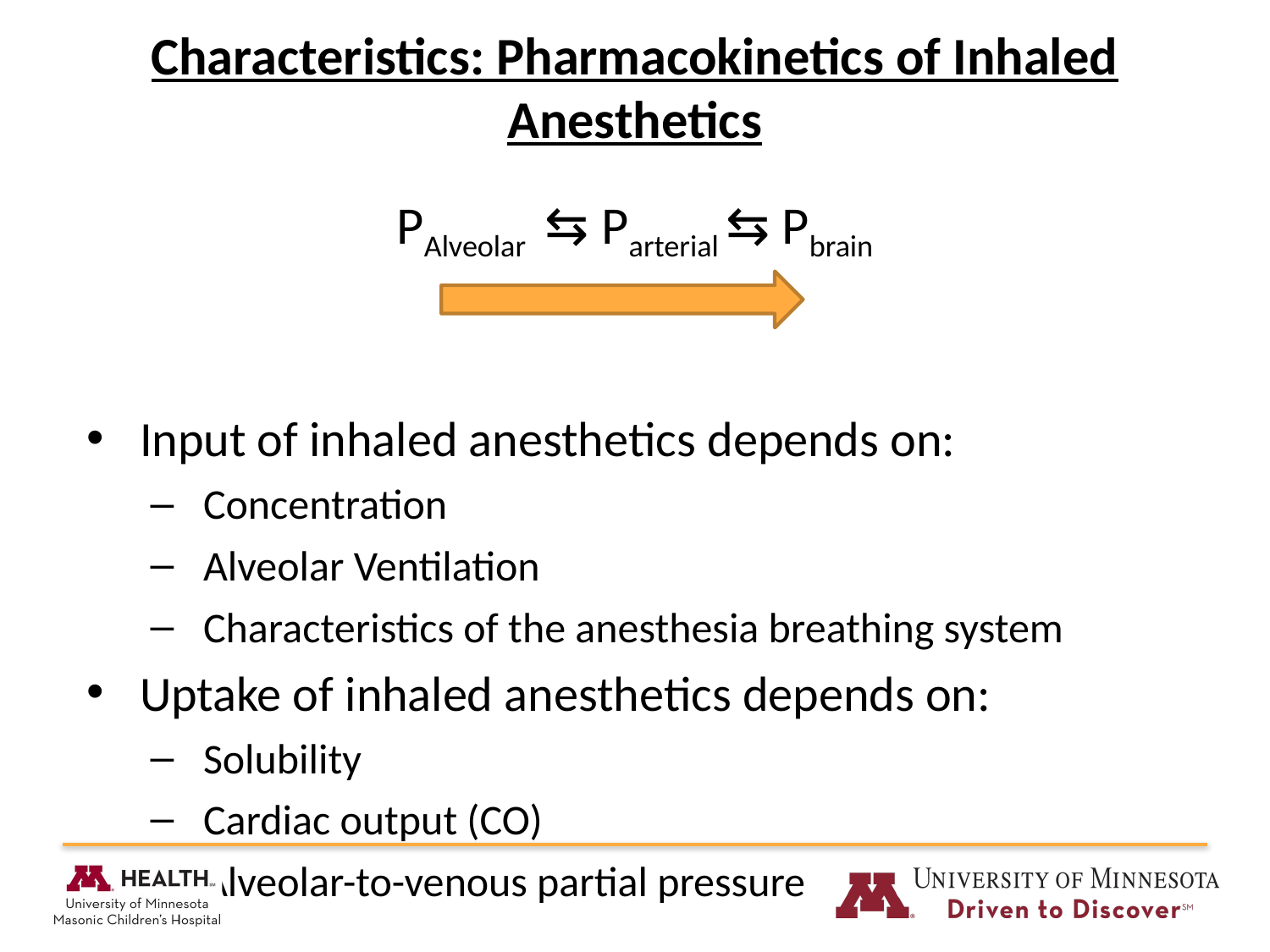

# Characteristics: Pharmacokinetics of Inhaled Anesthetics
PAlveolar ⇆ Parterial ⇆ Pbrain
Input of inhaled anesthetics depends on:
Concentration
Alveolar Ventilation
Characteristics of the anesthesia breathing system
Uptake of inhaled anesthetics depends on:
Solubility
Cardiac output (CO)
Alveolar-to-venous partial pressure

## Slide 28
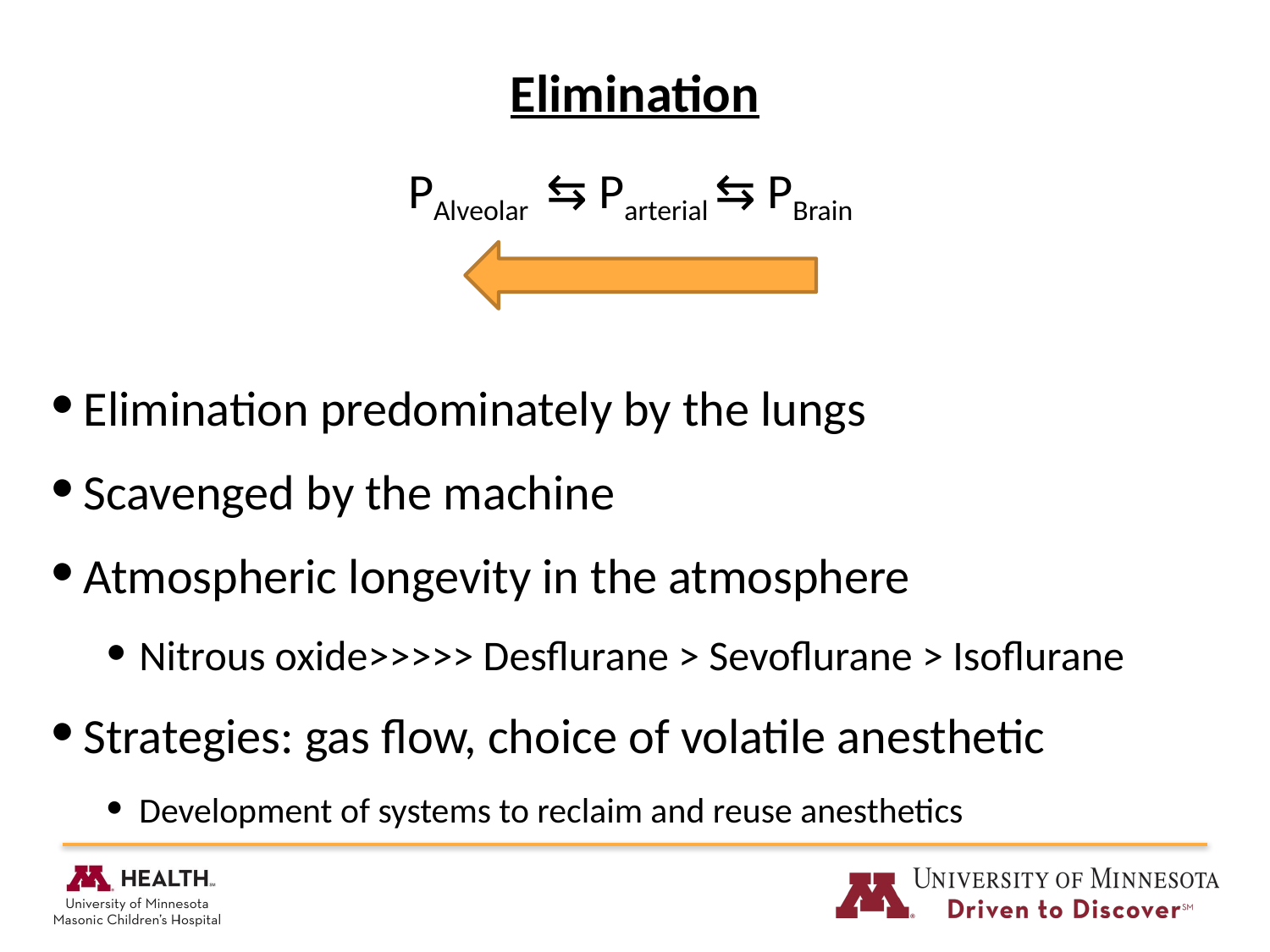

# Elimination
PAlveolar ⇆ Parterial ⇆ PBrain
Elimination predominately by the lungs
Scavenged by the machine
Atmospheric longevity in the atmosphere
Nitrous oxide>>>>> Desflurane > Sevoflurane > Isoflurane
Strategies: gas flow, choice of volatile anesthetic
Development of systems to reclaim and reuse anesthetics

## Slide 29
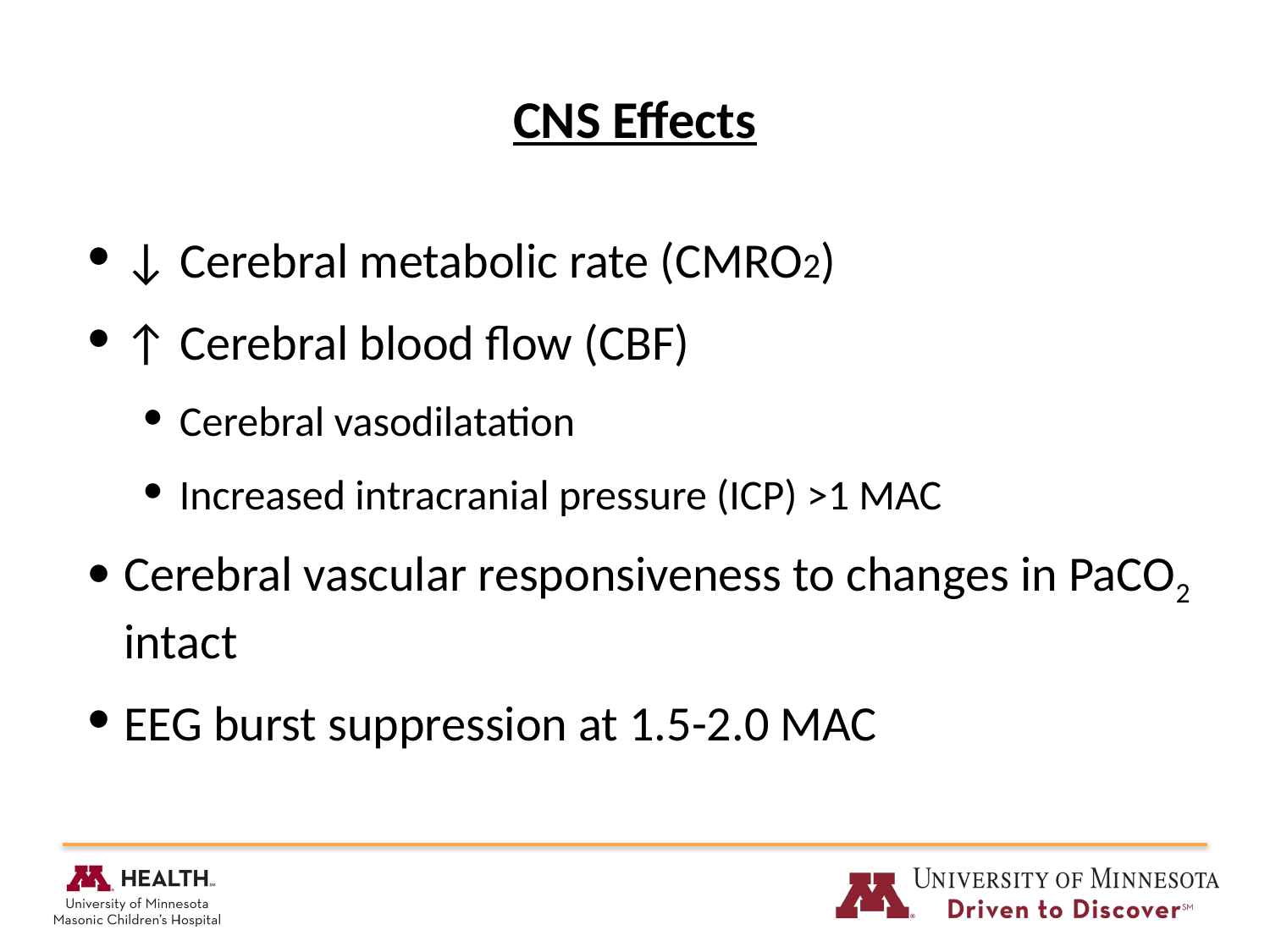

# CNS Effects
↓ Cerebral metabolic rate (CMRO2)
↑ Cerebral blood flow (CBF)
Cerebral vasodilatation
Increased intracranial pressure (ICP) >1 MAC
Cerebral vascular responsiveness to changes in PaCO2 intact
EEG burst suppression at 1.5-2.0 MAC

## Slide 30
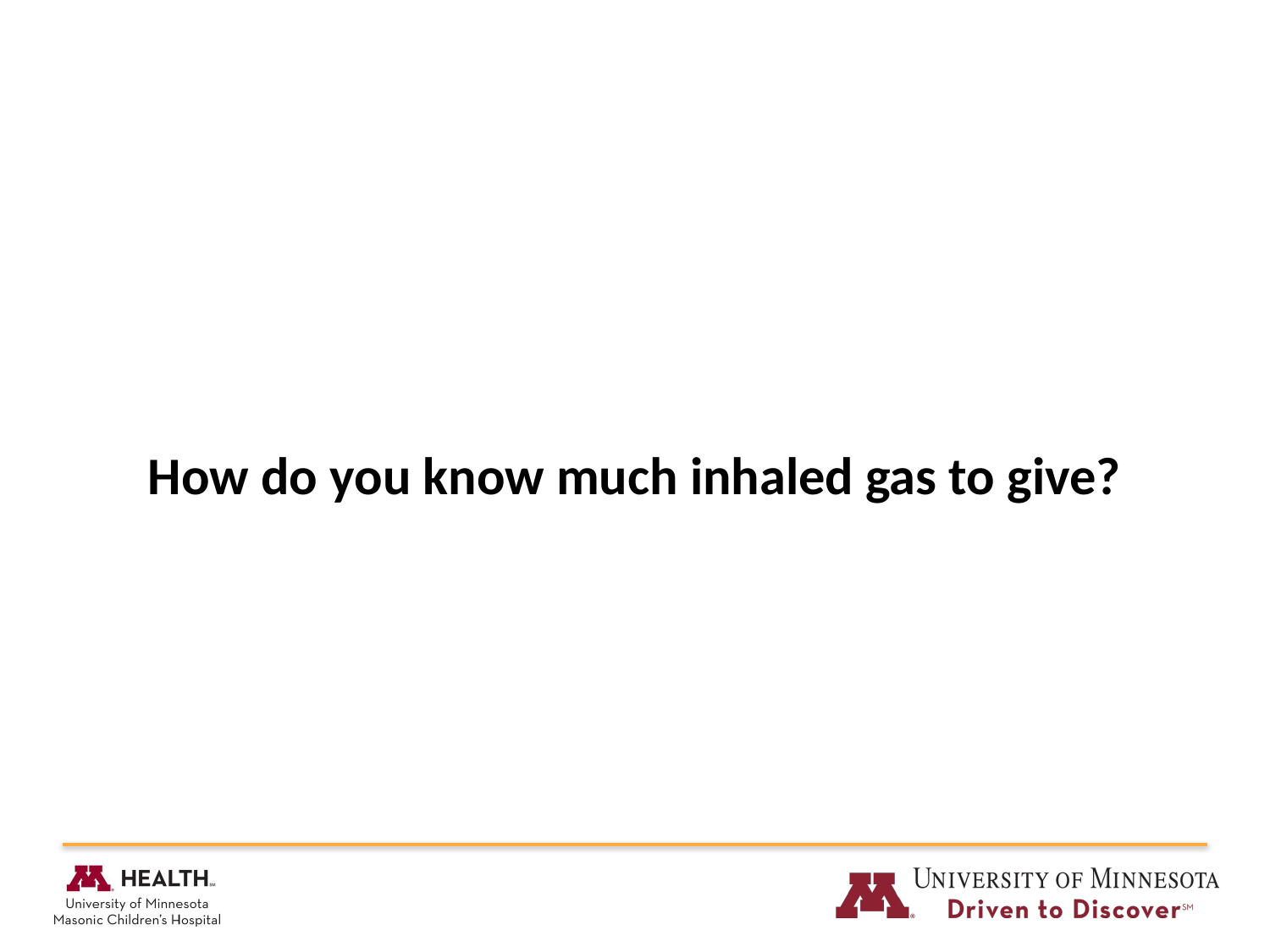

# How do you know much inhaled gas to give?

## Slide 31
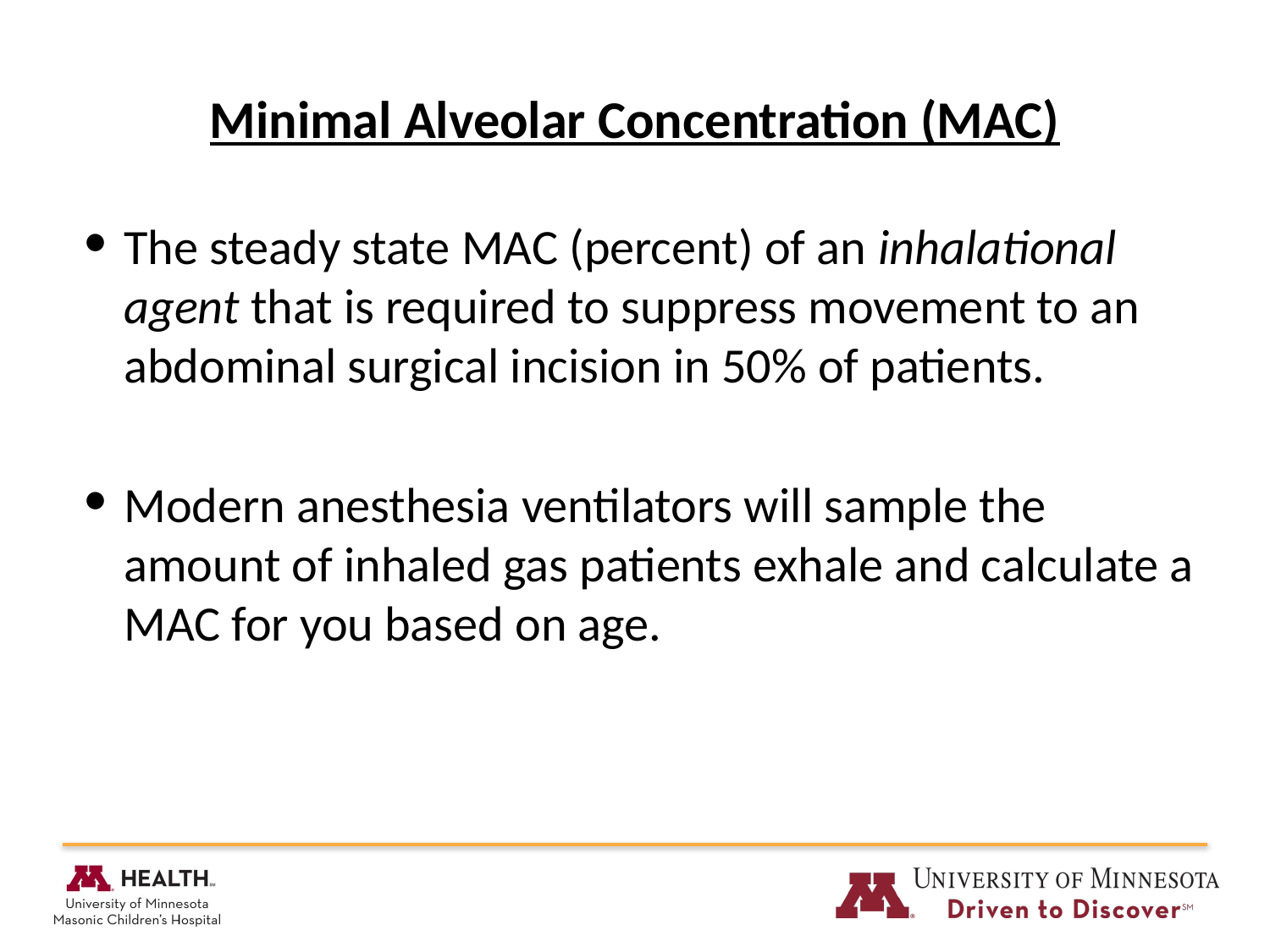

# Minimal Alveolar Concentration (MAC)
The steady state MAC (percent) of an inhalational agent that is required to suppress movement to an abdominal surgical incision in 50% of patients.
Modern anesthesia ventilators will sample the amount of inhaled gas patients exhale and calculate a MAC for you based on age.
 adjunct

## Slide 32
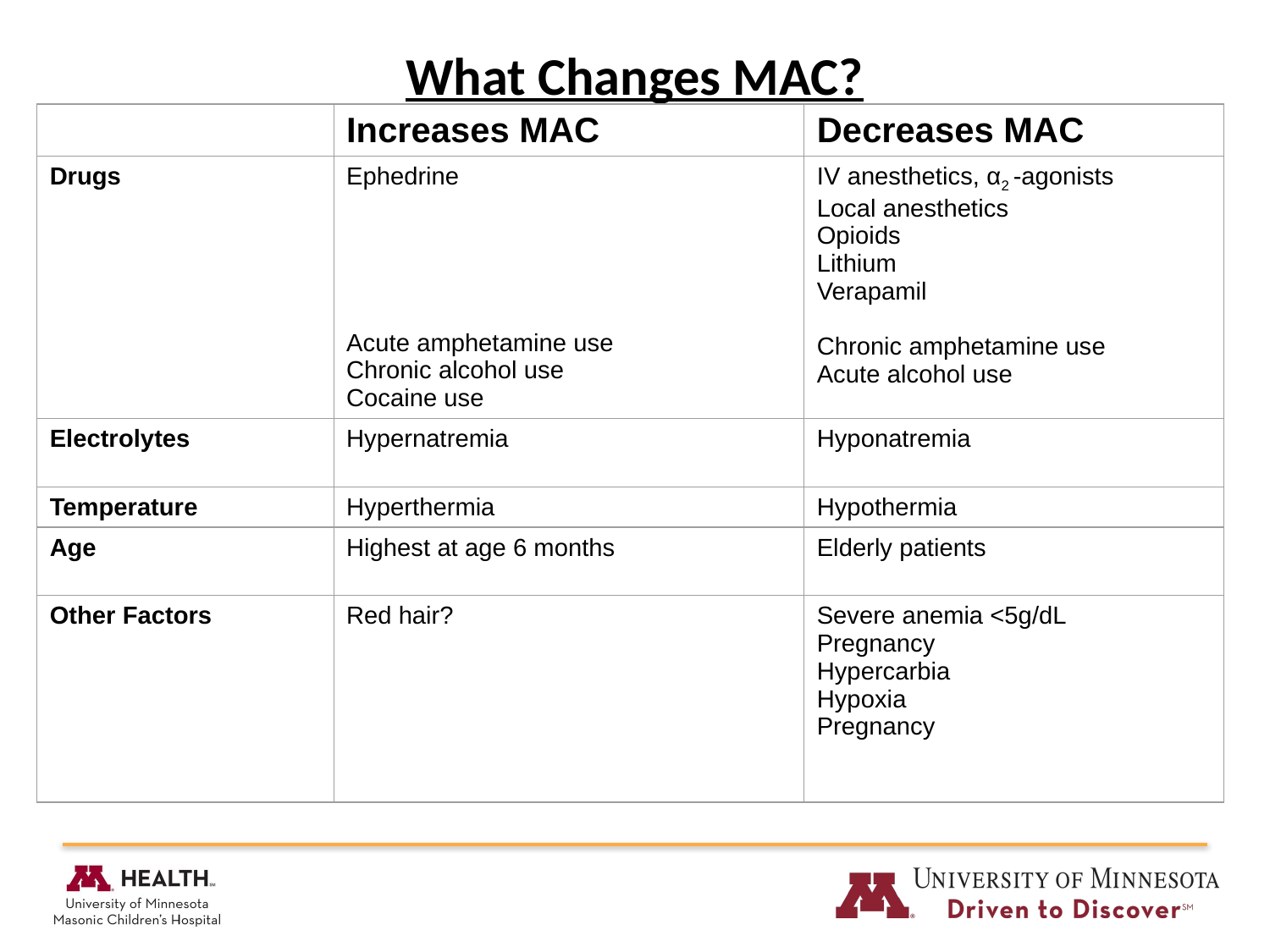

# What Changes MAC?
| | Increases MAC | Decreases MAC |
| --- | --- | --- |
| Drugs | Ephedrine Acute amphetamine use Chronic alcohol use Cocaine use | IV anesthetics, α2 -agonists Local anesthetics Opioids Lithium Verapamil Chronic amphetamine use Acute alcohol use |
| Electrolytes | Hypernatremia | Hyponatremia |
| Temperature | Hyperthermia | Hypothermia |
| Age | Highest at age 6 months | Elderly patients |
| Other Factors | Red hair? | Severe anemia <5g/dL Pregnancy Hypercarbia Hypoxia Pregnancy |

## Slide 33
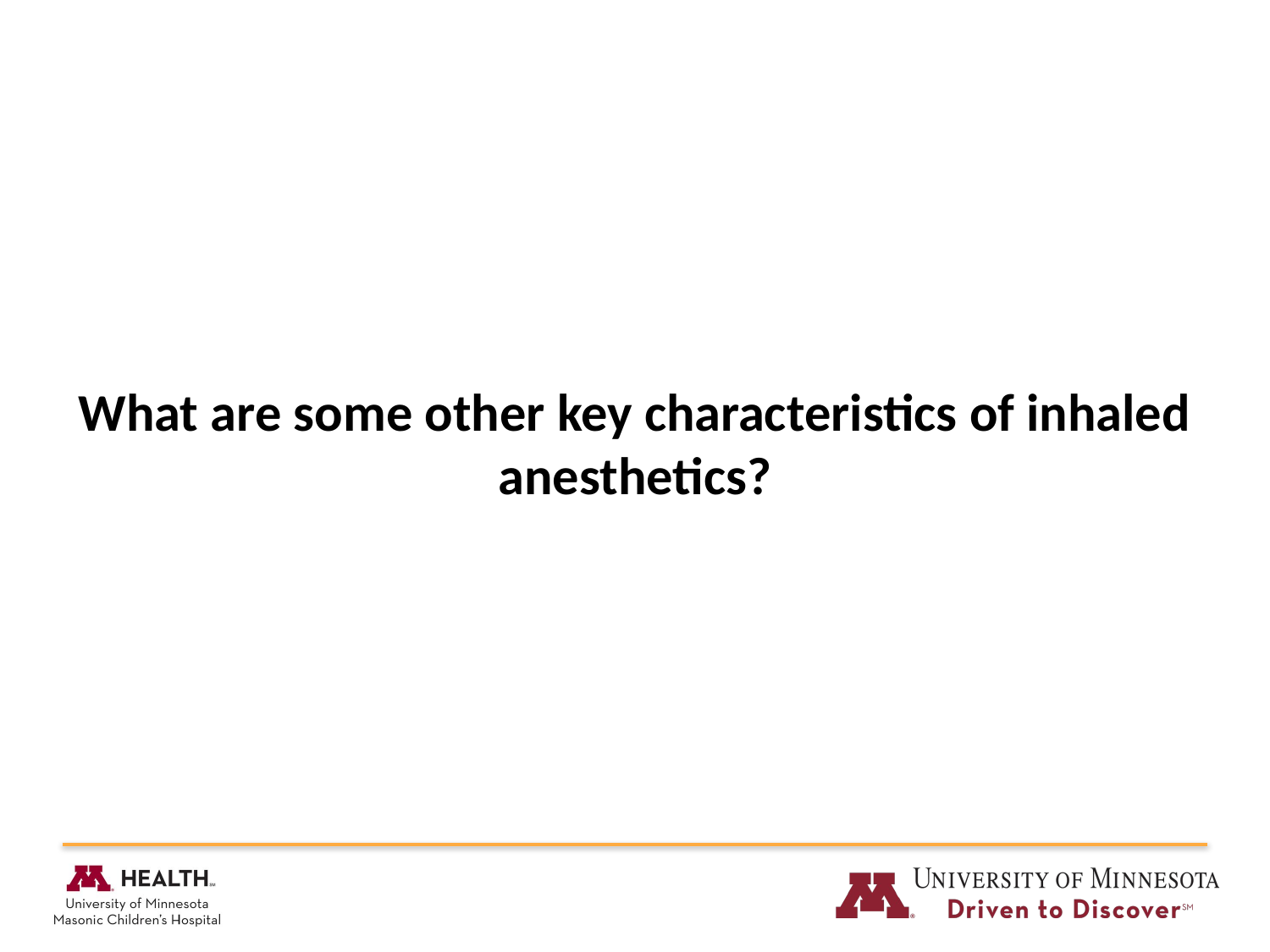

# What are some other key characteristics of inhaled anesthetics?

## Slide 34
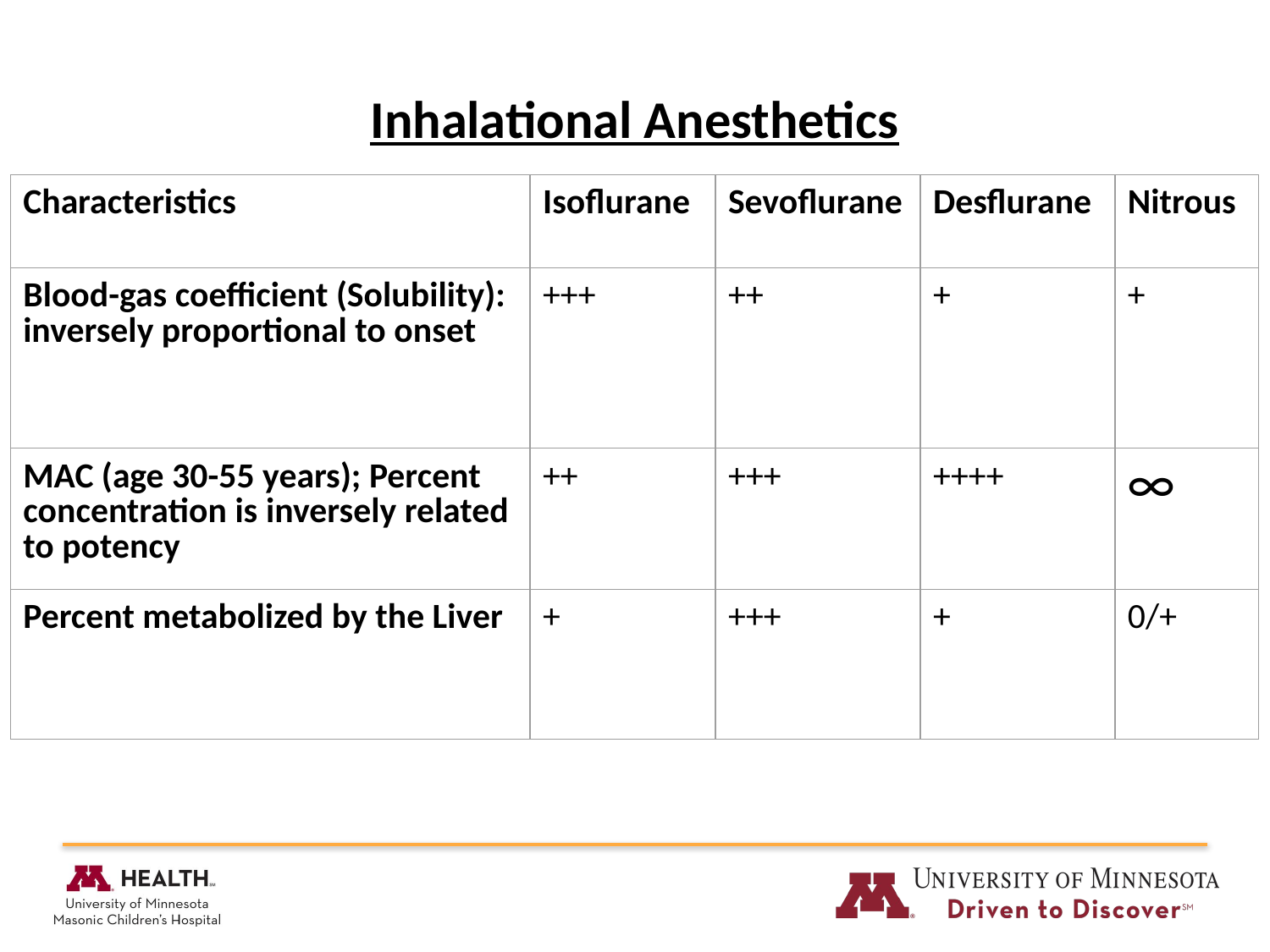

# Inhalational Anesthetics
adjunct
| Characteristics | Isoflurane | Sevoflurane | Desflurane | Nitrous |
| --- | --- | --- | --- | --- |
| Blood-gas coefficient (Solubility): inversely proportional to onset | +++ | ++ | + | + |
| MAC (age 30-55 years); Percent concentration is inversely related to potency | ++ | +++ | ++++ | ∞ |
| Percent metabolized by the Liver | + | +++ | + | 0/+ |

## Slide 35
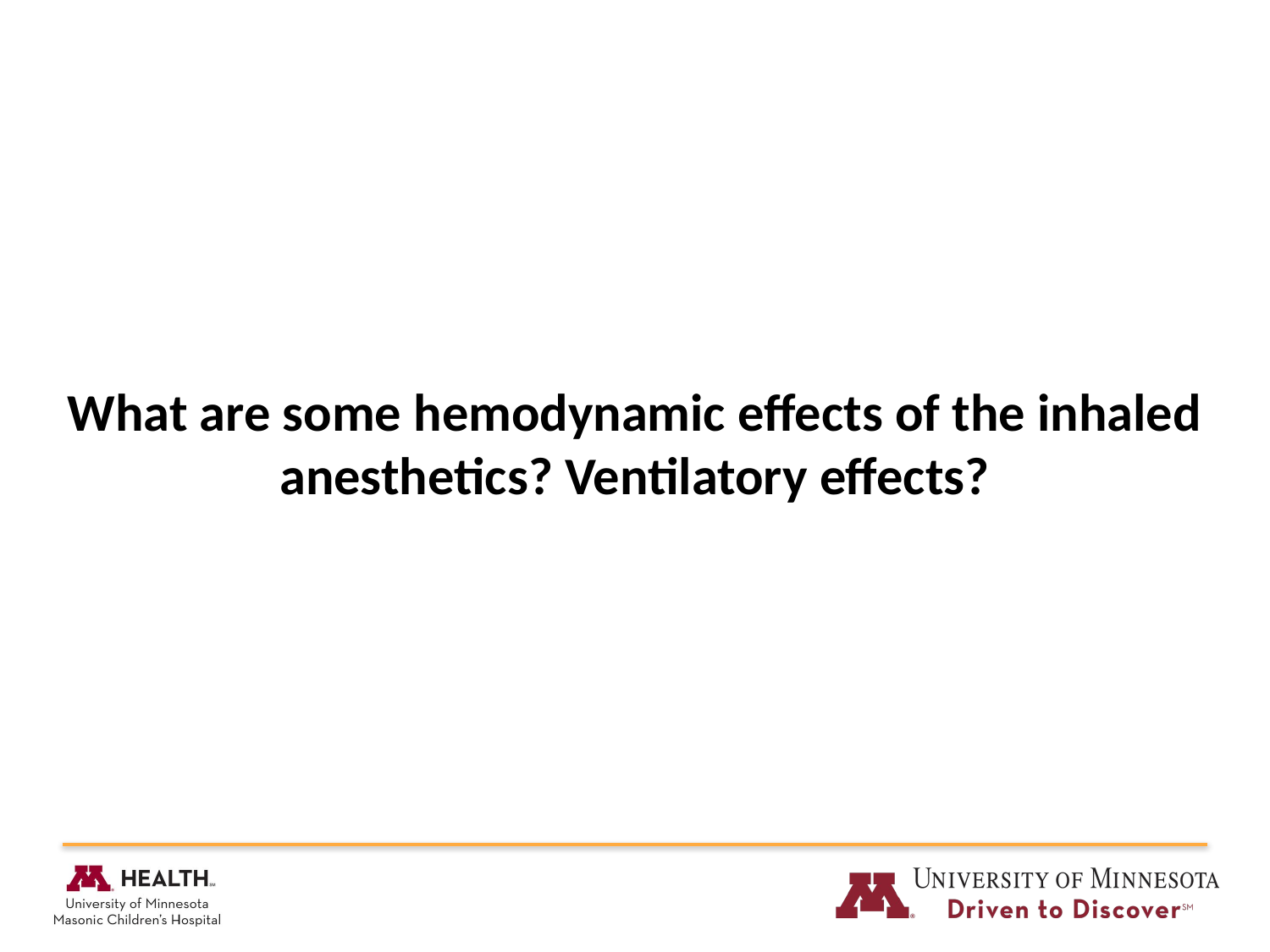

# What are some hemodynamic effects of the inhaled anesthetics? Ventilatory effects?

## Slide 36
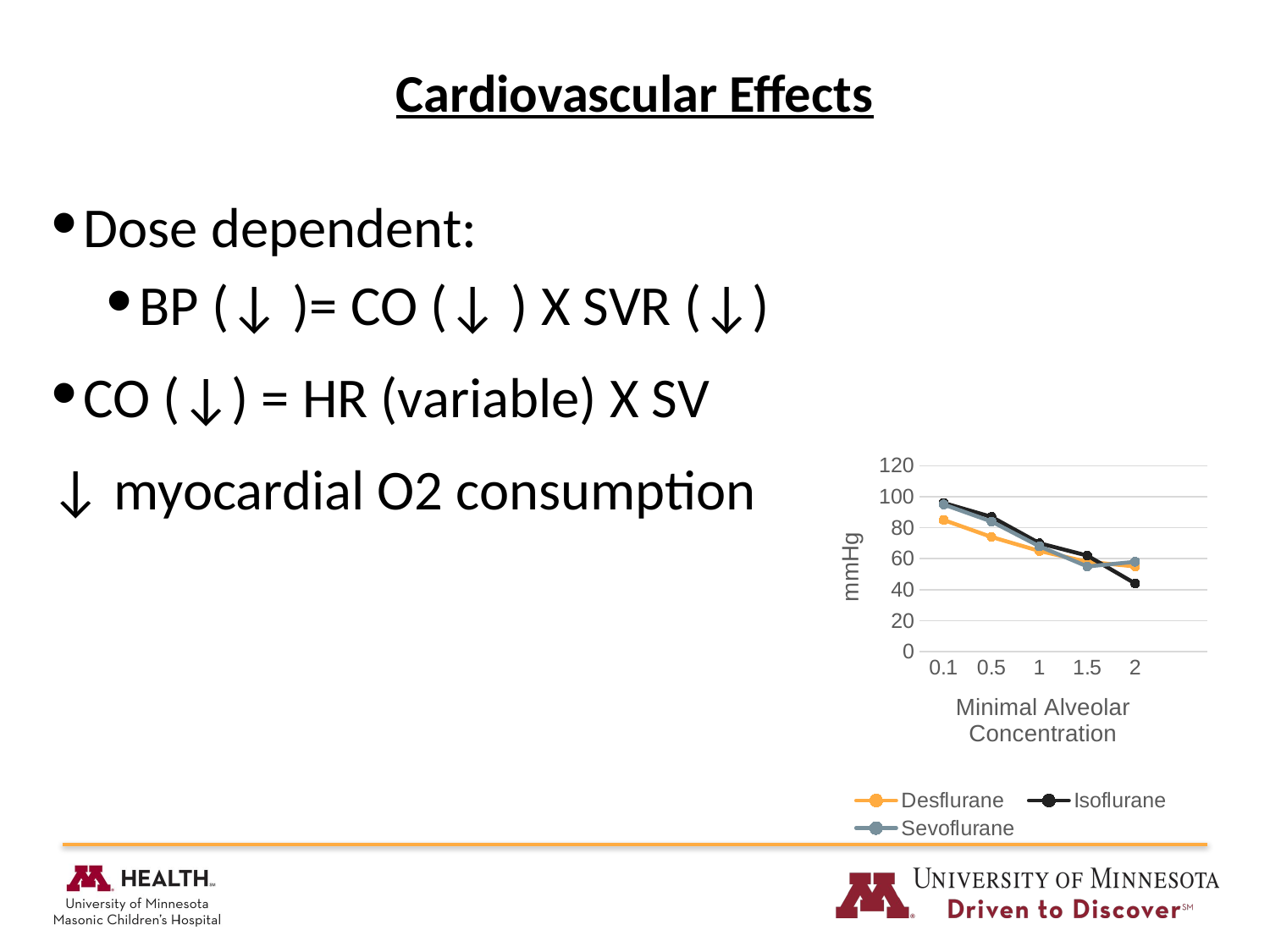

# Cardiovascular Effects
Dose dependent:
BP (↓ )= CO (↓ ) X SVR (↓)
CO (↓) = HR (variable) X SV
↓ myocardial O2 consumption
### Chart
| Category | Desflurane | Isoflurane | Sevoflurane |
|---|---|---|---|
| 0.1 | 85.0 | 96.0 | 95.0 |
| 0.5 | 74.0 | 87.0 | 84.0 |
| 1 | 65.0 | 70.0 | 68.0 |
| 1.5 | 58.0 | 62.0 | 55.0 |
| 2 | 55.0 | 44.0 | 58.0 |
| | None | None | None |

## Slide 37
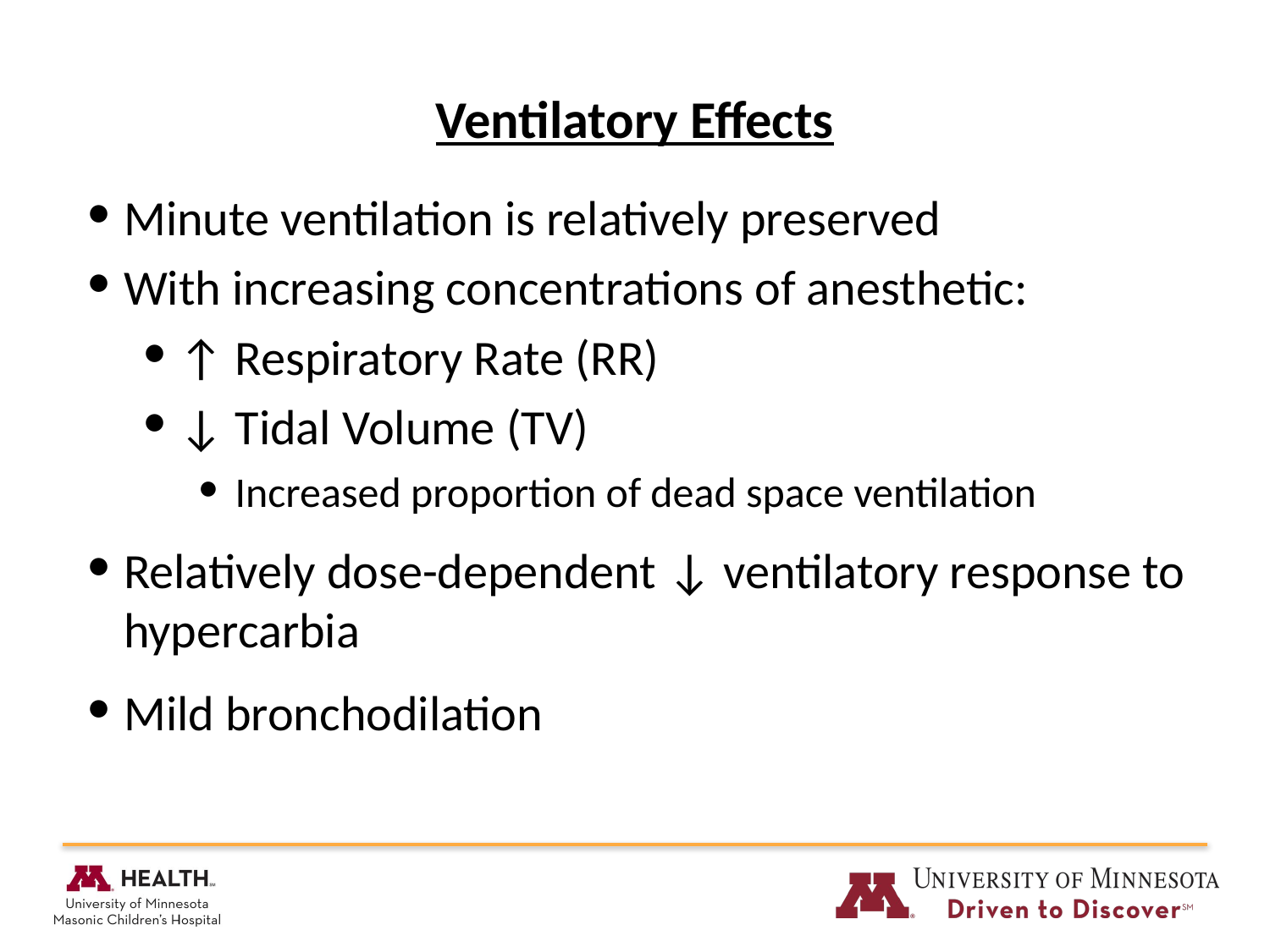

# Ventilatory Effects
Minute ventilation is relatively preserved
With increasing concentrations of anesthetic:
↑ Respiratory Rate (RR)
↓ Tidal Volume (TV)
Increased proportion of dead space ventilation
Relatively dose-dependent ↓ ventilatory response to hypercarbia
Mild bronchodilation

## Slide 38
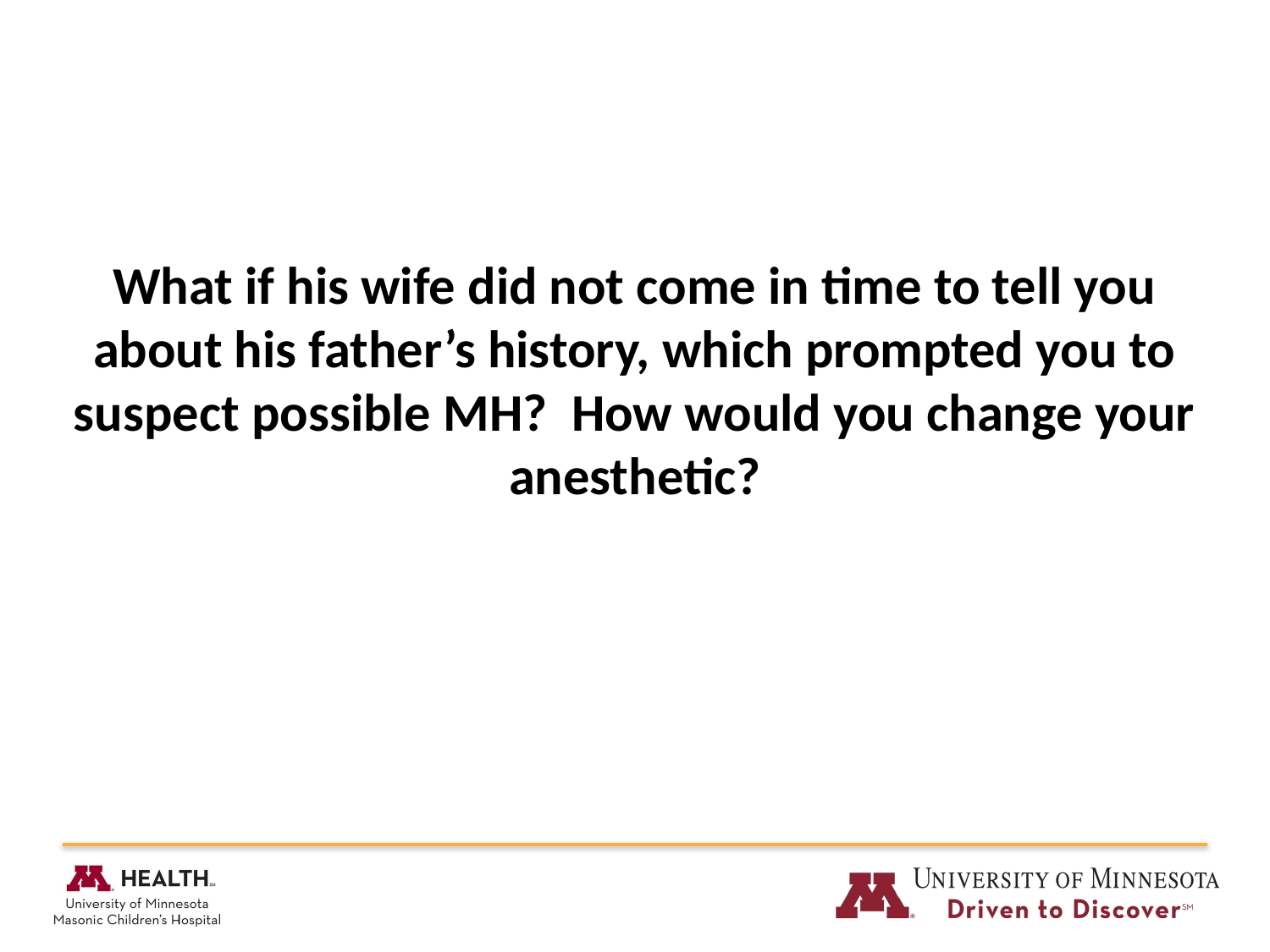

# What if his wife did not come in time to tell you about his father’s history, which prompted you to suspect possible MH? How would you change your anesthetic?

## Slide 39
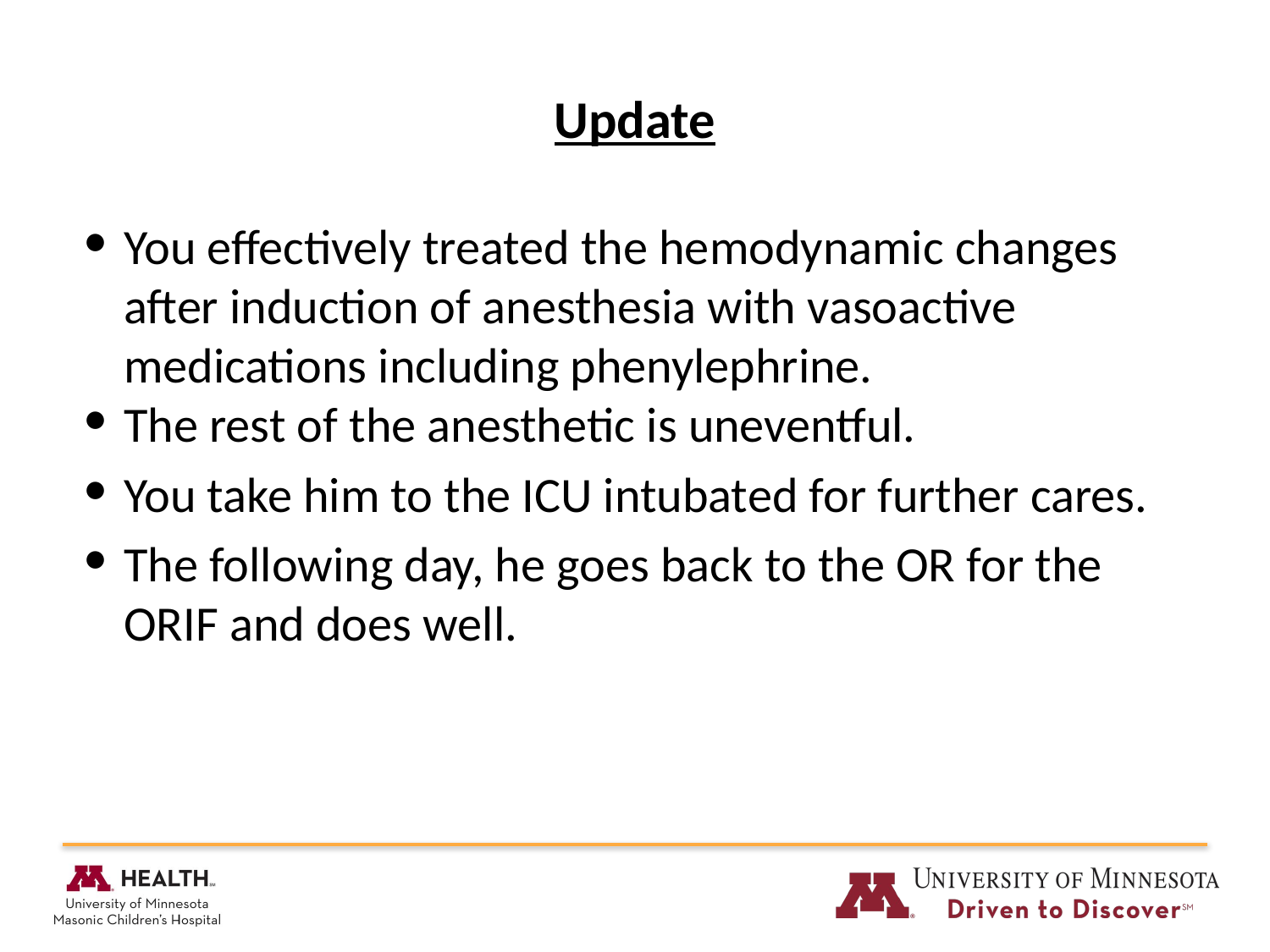

# Update
You effectively treated the hemodynamic changes after induction of anesthesia with vasoactive medications including phenylephrine.
The rest of the anesthetic is uneventful.
You take him to the ICU intubated for further cares.
The following day, he goes back to the OR for the ORIF and does well.
adjunct

## Slide 40
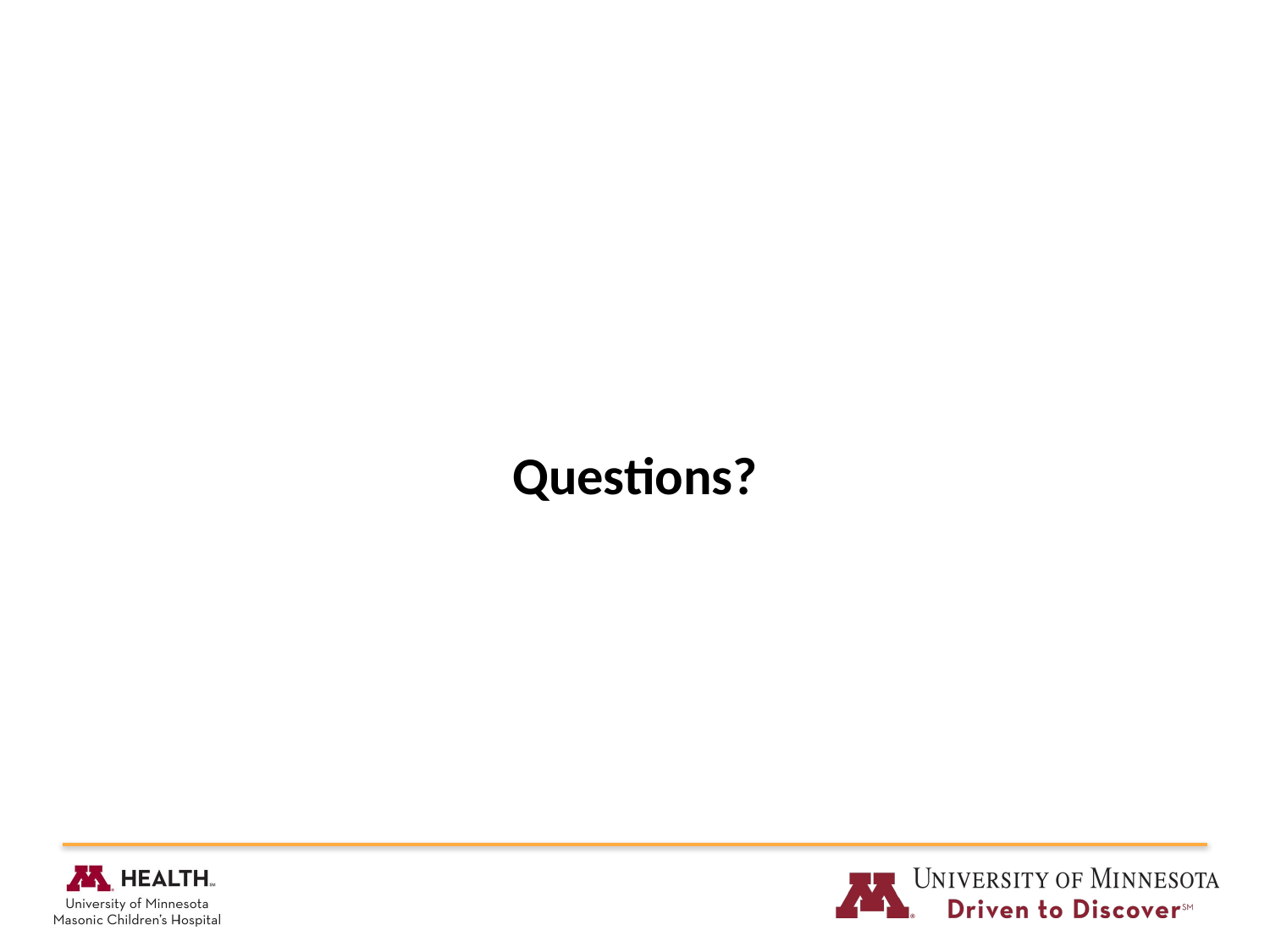

# Questions?

## Slide 41
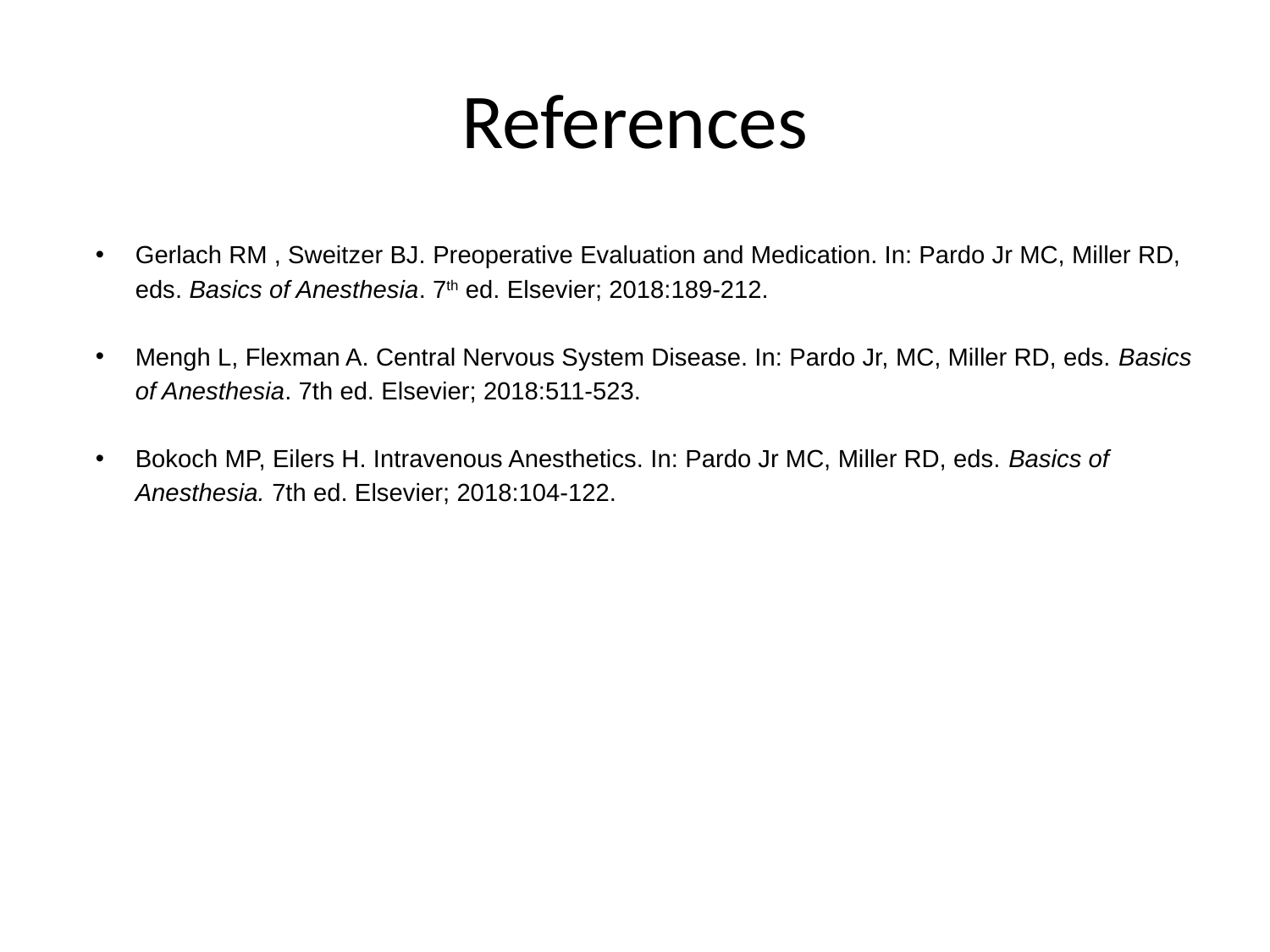

# References
Gerlach RM , Sweitzer BJ. Preoperative Evaluation and Medication. In: Pardo Jr MC, Miller RD, eds. Basics of Anesthesia. 7th ed. Elsevier; 2018:189-212.
Mengh L, Flexman A. Central Nervous System Disease. In: Pardo Jr, MC, Miller RD, eds. Basics of Anesthesia. 7th ed. Elsevier; 2018:511-523.
Bokoch MP, Eilers H. Intravenous Anesthetics. In: Pardo Jr MC, Miller RD, eds. Basics of Anesthesia. 7th ed. Elsevier; 2018:104-122.
