# Supplementary material for: Development and Implementation of Video-Recorded Simulation Scenarios to Facilitate Case-Based Learning Discussions for Medical Students' Virtual Anesthesiology Clerkship
Source: MedEdPORTAL. 2023 Apr 4;19:11306. doi: 10.15766/mep_2374-8265.11306 (PMC10070881; doi:10.15766/mep_2374-8265.11306)
Supplement: Supplementary file 1 — Preoperative Evaluation - CBLD 1.pptxInhaled and Intravenous Anesthetics - CBLD 2.pptxAirway Management - CBLD 3.pptxScenario 1.mp4Scenario 2.mp4Scenario 3.mp4Scenario Debrief 1.docxScenario Debrief 2.docxScenario Debrief 3.docxClerkship Survey Questions.docxCBLD-Specific Survey Questions.docx [file mep_2374-8265.11306-s001.zip › C. Airway Management - CBLD 3.pptx]

## Slide 1
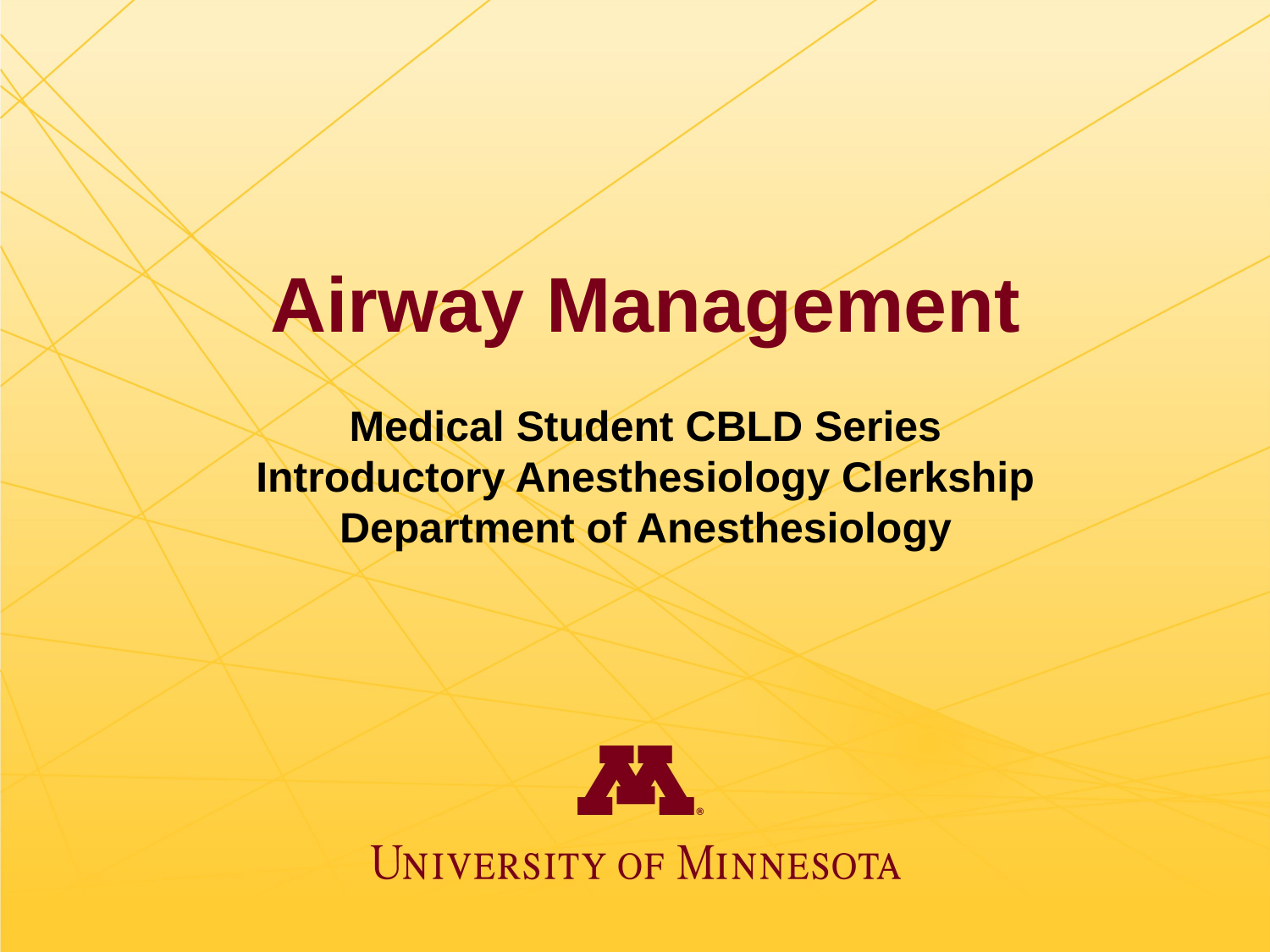

# Airway Management Medical Student CBLD Series Introductory Anesthesiology Clerkship Department of Anesthesiology

## Slide 2
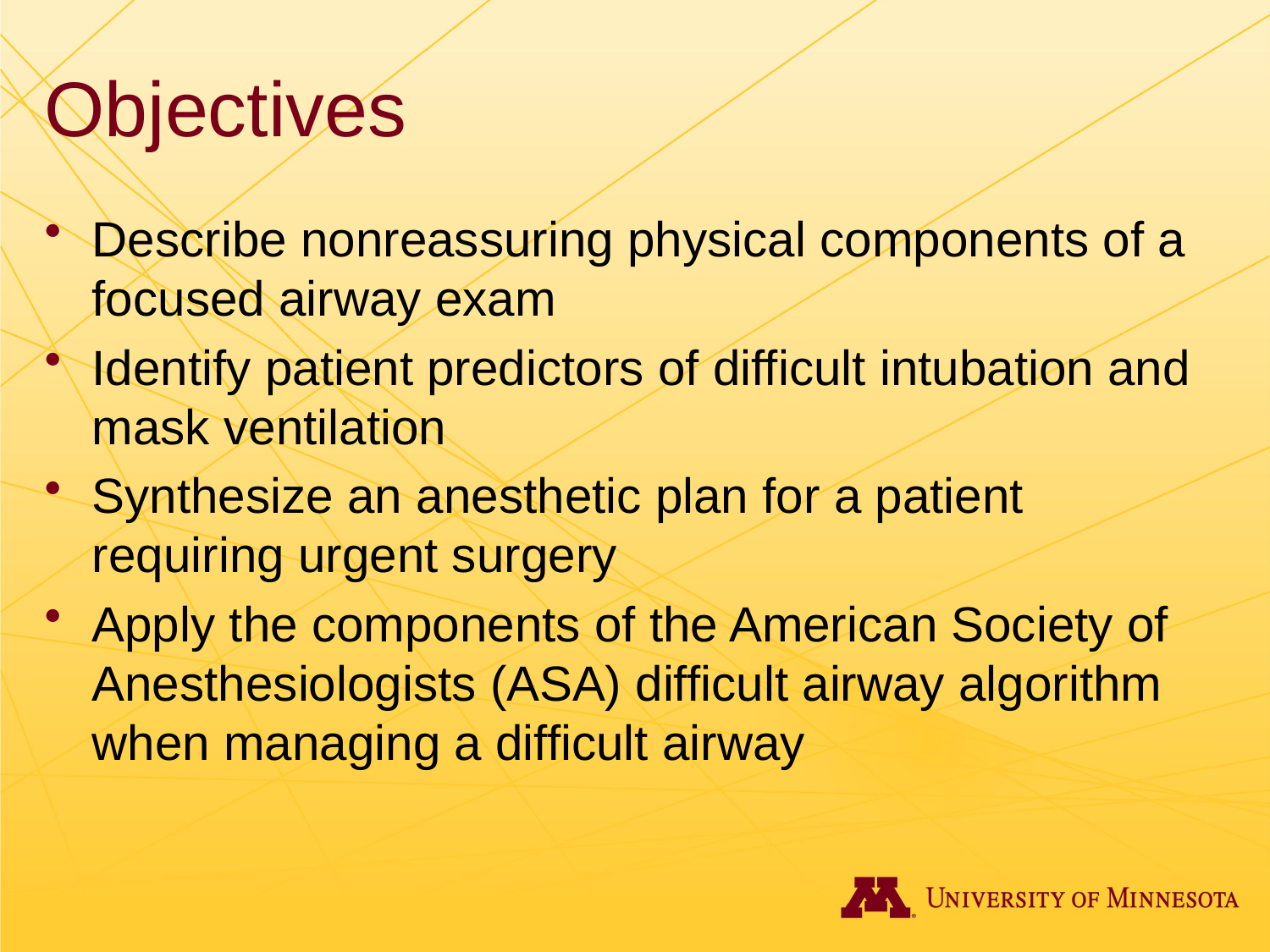

# Objectives
Describe nonreassuring physical components of a focused airway exam
Identify patient predictors of difficult intubation and mask ventilation
Synthesize an anesthetic plan for a patient requiring urgent surgery
Apply the components of the American Society of Anesthesiologists (ASA) difficult airway algorithm when managing a difficult airway

## Slide 3
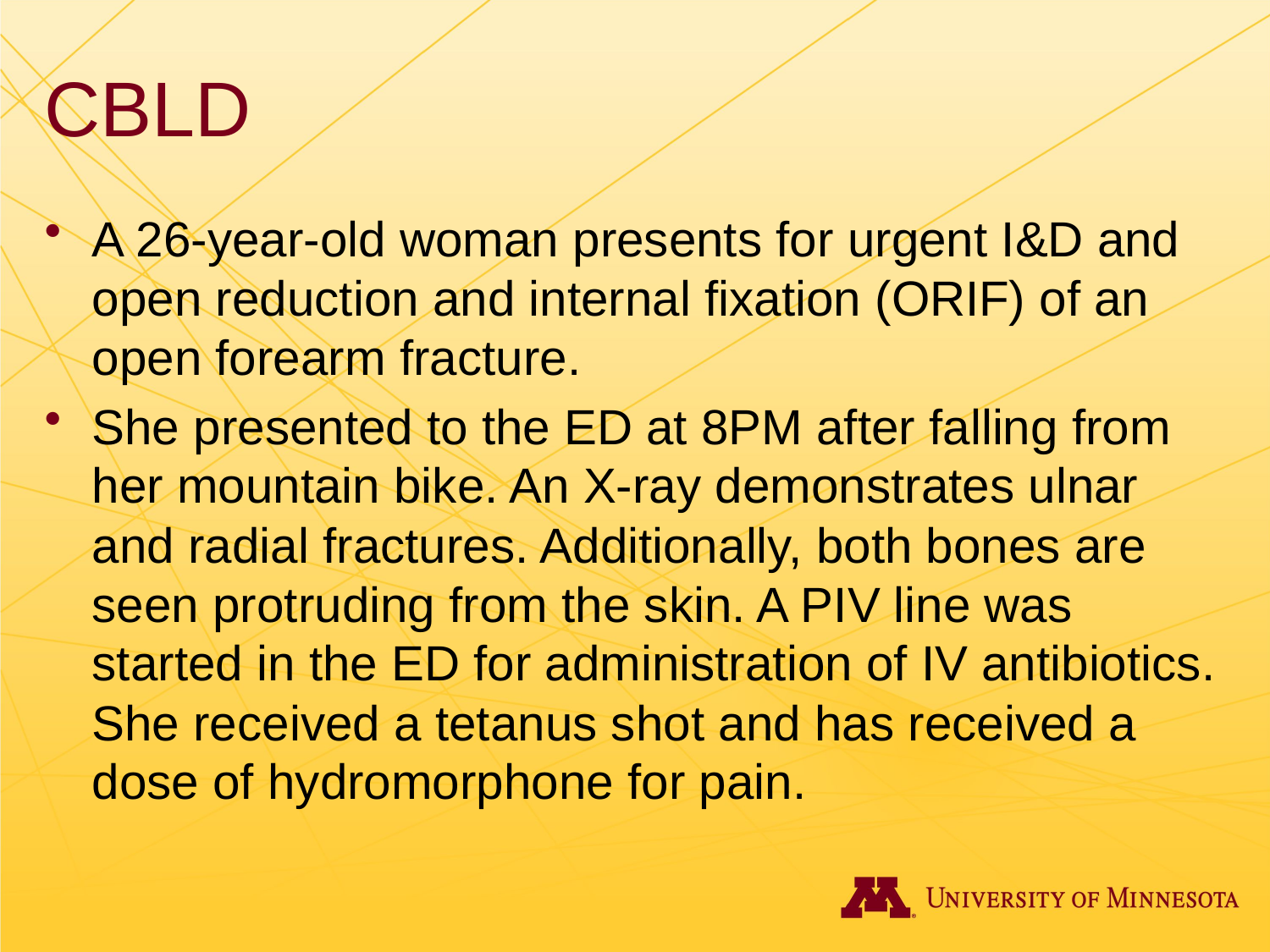

# CBLD
A 26-year-old woman presents for urgent I&D and open reduction and internal fixation (ORIF) of an open forearm fracture.
She presented to the ED at 8PM after falling from her mountain bike. An X-ray demonstrates ulnar and radial fractures. Additionally, both bones are seen protruding from the skin. A PIV line was started in the ED for administration of IV antibiotics. She received a tetanus shot and has received a dose of hydromorphone for pain.

## Slide 4
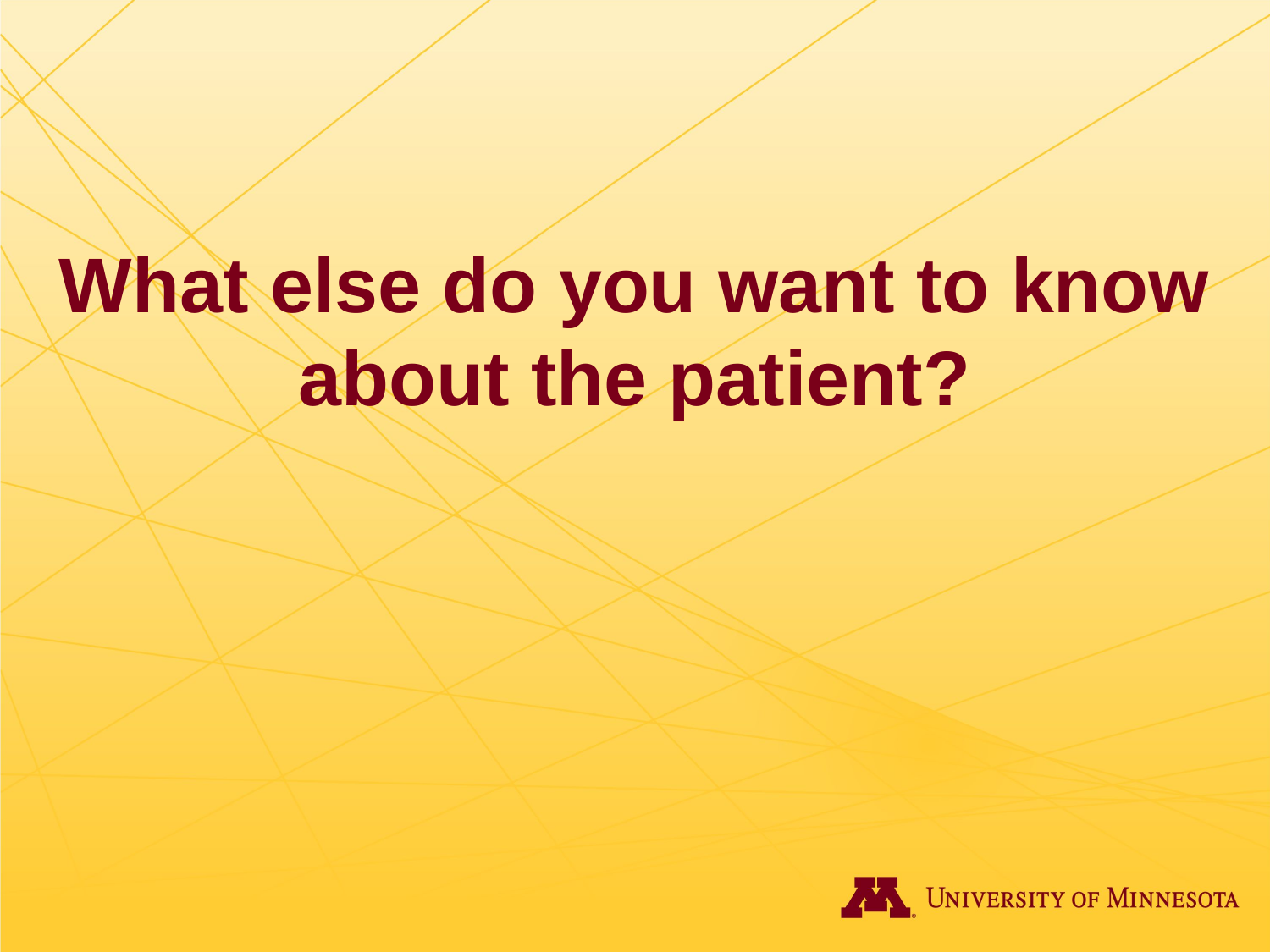

# What else do you want to know about the patient?

## Slide 5
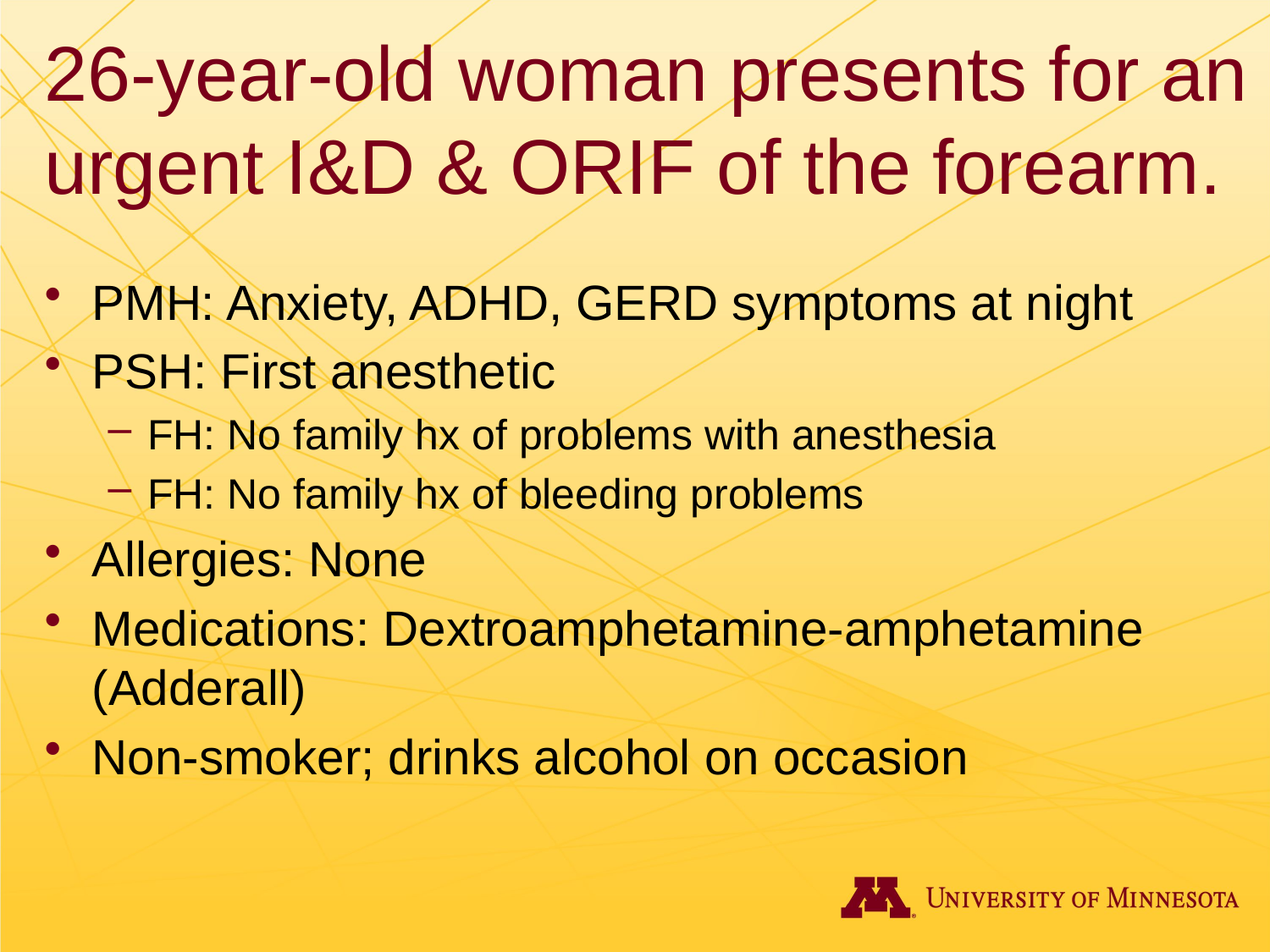

# 26-year-old woman presents for an urgent I&D & ORIF of the forearm.
PMH: Anxiety, ADHD, GERD symptoms at night
PSH: First anesthetic
FH: No family hx of problems with anesthesia
FH: No family hx of bleeding problems
Allergies: None
Medications: Dextroamphetamine-amphetamine (Adderall)
Non-smoker; drinks alcohol on occasion

## Slide 6
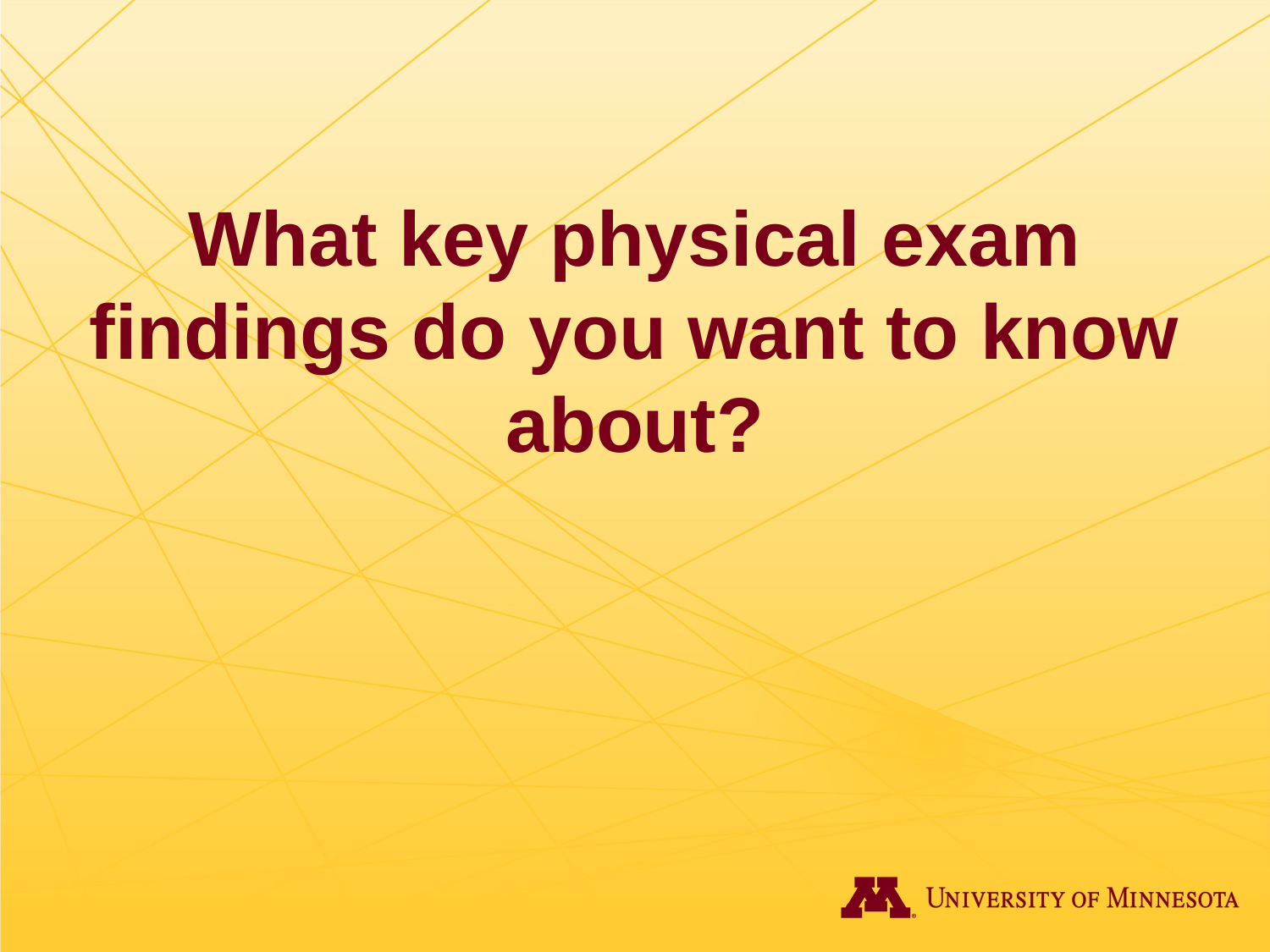

# What key physical exam findings do you want to know about?

## Slide 7
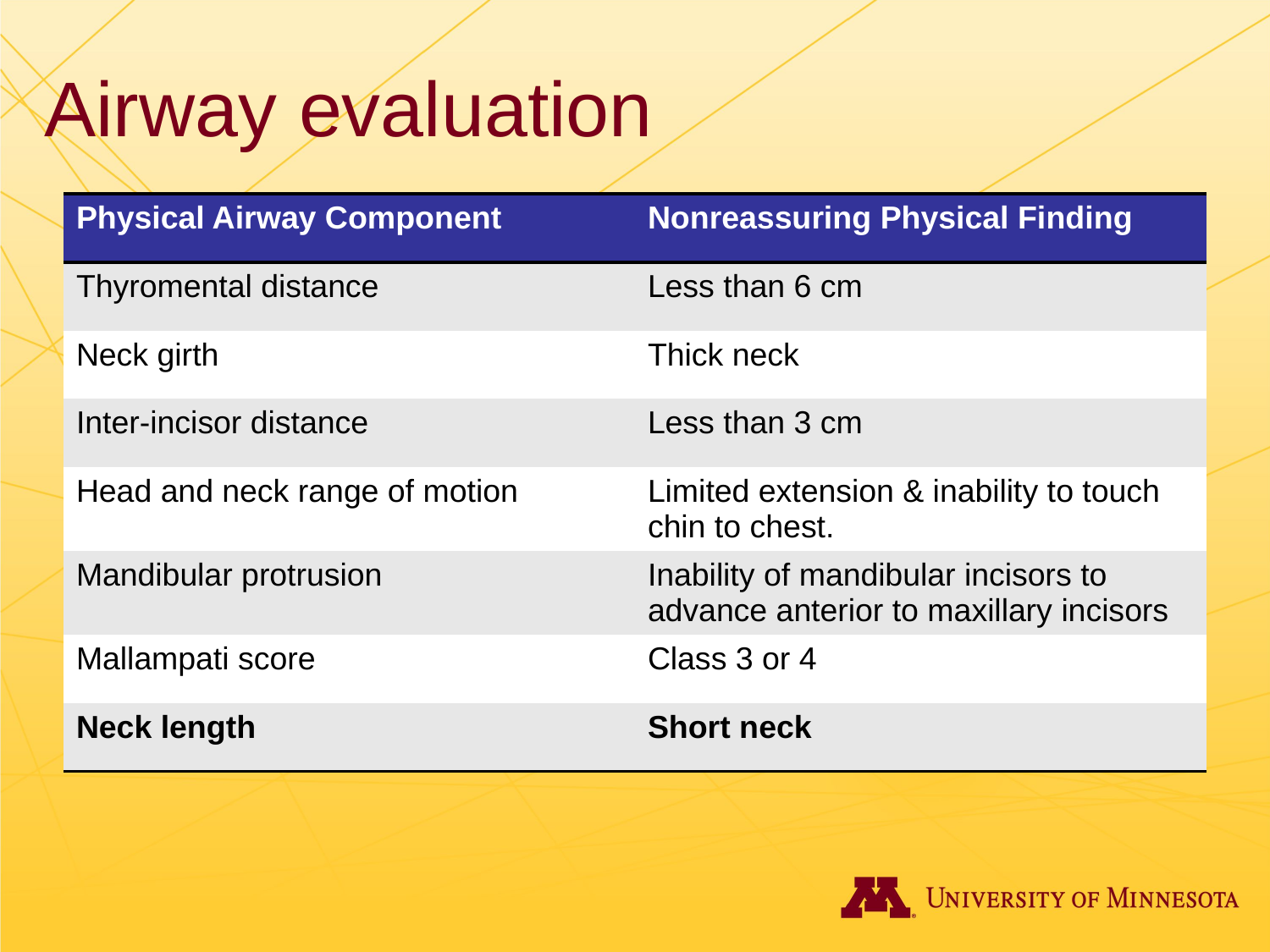

# Airway evaluation
| Physical Airway Component | Nonreassuring Physical Finding |
| --- | --- |
| Thyromental distance | Less than 6 cm |
| Neck girth | Thick neck |
| Inter-incisor distance | Less than 3 cm |
| Head and neck range of motion | Limited extension & inability to touch chin to chest. |
| Mandibular protrusion | Inability of mandibular incisors to advance anterior to maxillary incisors |
| Mallampati score | Class 3 or 4 |
| Neck length | Short neck |

## Slide 8
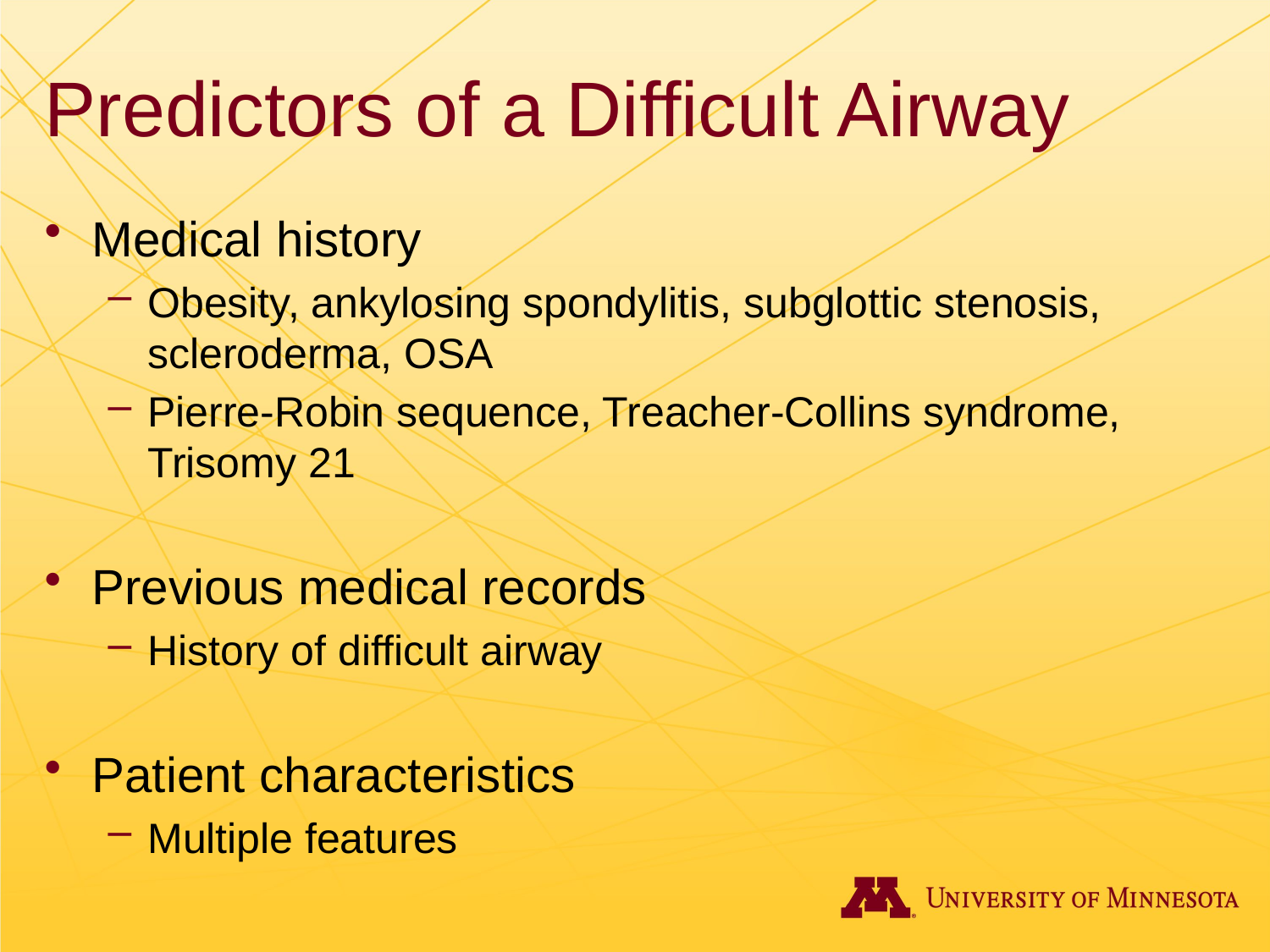

# Predictors of a Difficult Airway
Medical history
Obesity, ankylosing spondylitis, subglottic stenosis, scleroderma, OSA
Pierre-Robin sequence, Treacher-Collins syndrome, Trisomy 21
Previous medical records
History of difficult airway
Patient characteristics
Multiple features

## Slide 9
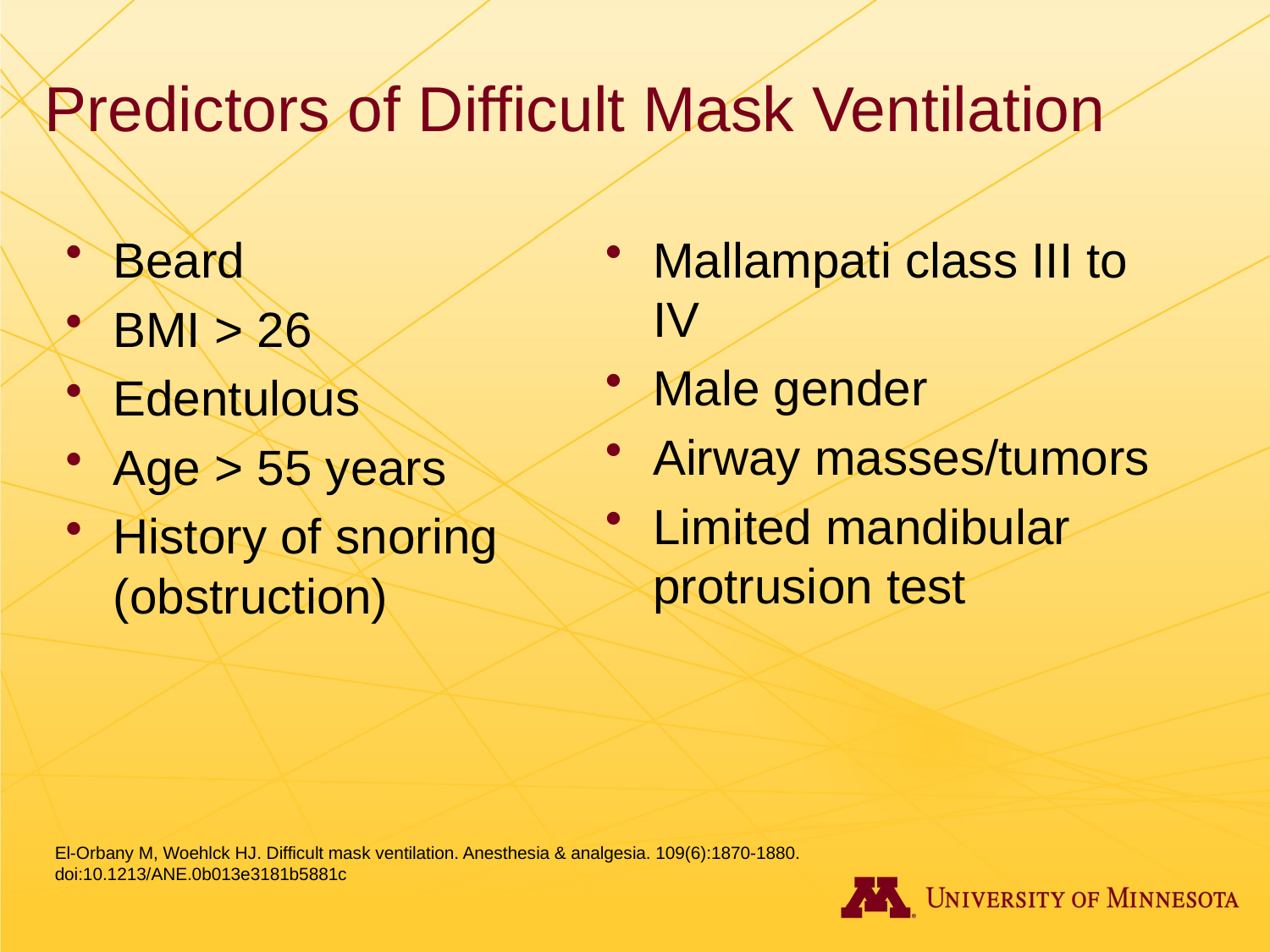

# Predictors of Difficult Mask Ventilation
Beard
BMI > 26
Edentulous
Age > 55 years
History of snoring (obstruction)
Mallampati class III to IV
Male gender
Airway masses/tumors
Limited mandibular protrusion test
El-Orbany M, Woehlck HJ. Difficult mask ventilation. Anesthesia & analgesia. 109(6):1870-1880. doi:10.1213/ANE.0b013e3181b5881c

## Slide 10
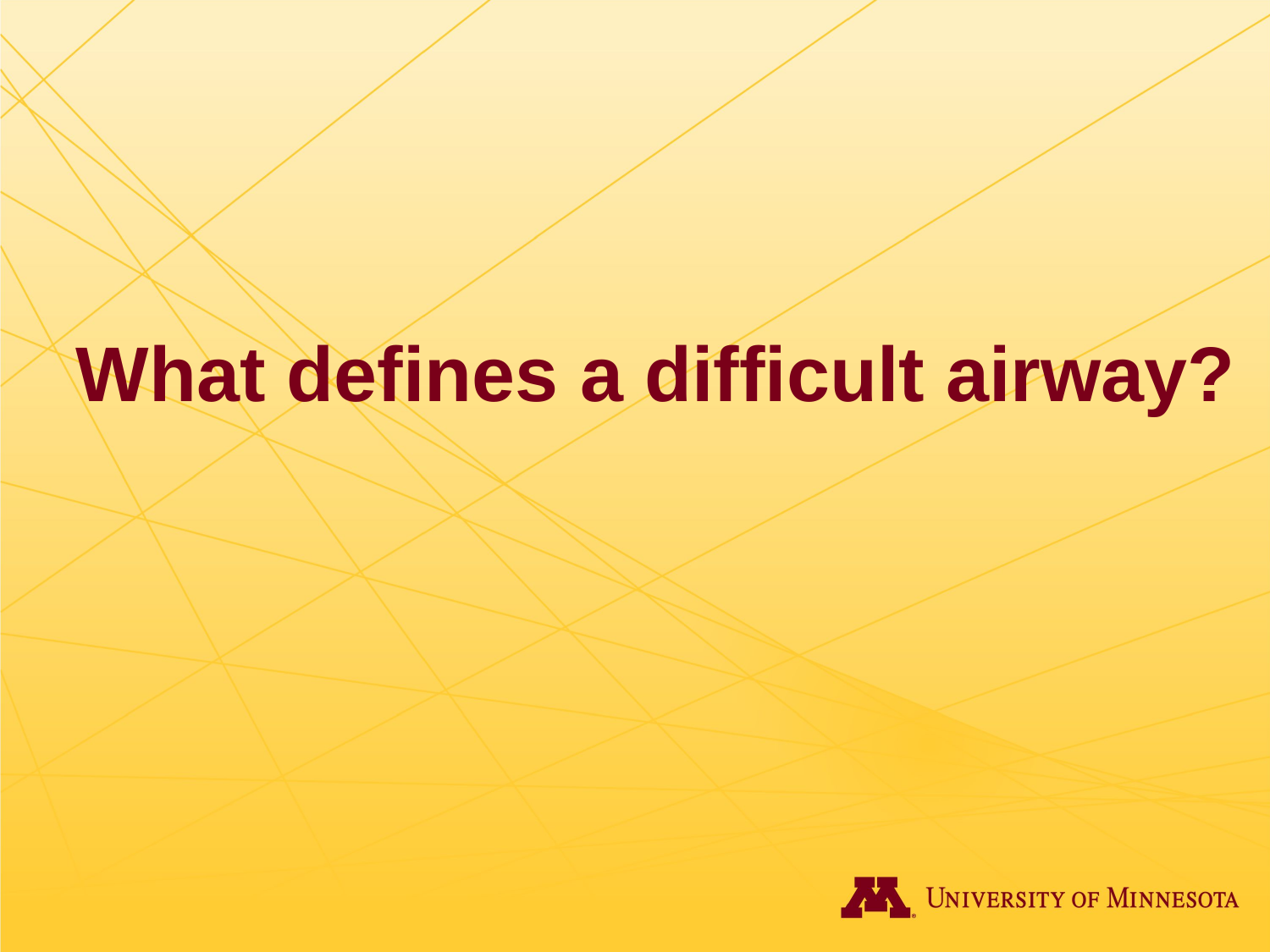

# What defines a difficult airway?

## Slide 11
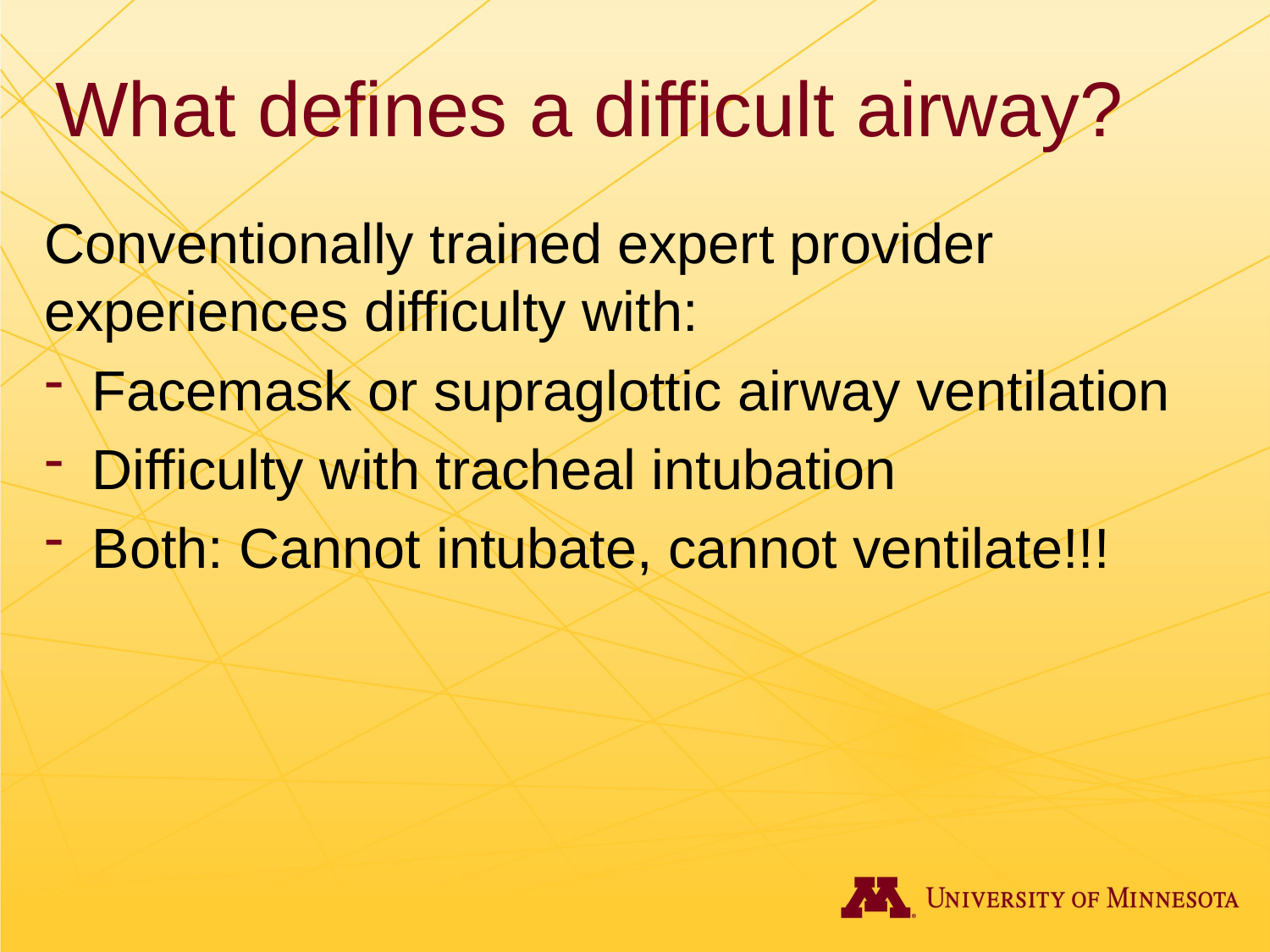

# What defines a difficult airway?
Conventionally trained expert provider experiences difficulty with:
Facemask or supraglottic airway ventilation
Difficulty with tracheal intubation
Both: Cannot intubate, cannot ventilate!!!

## Slide 12
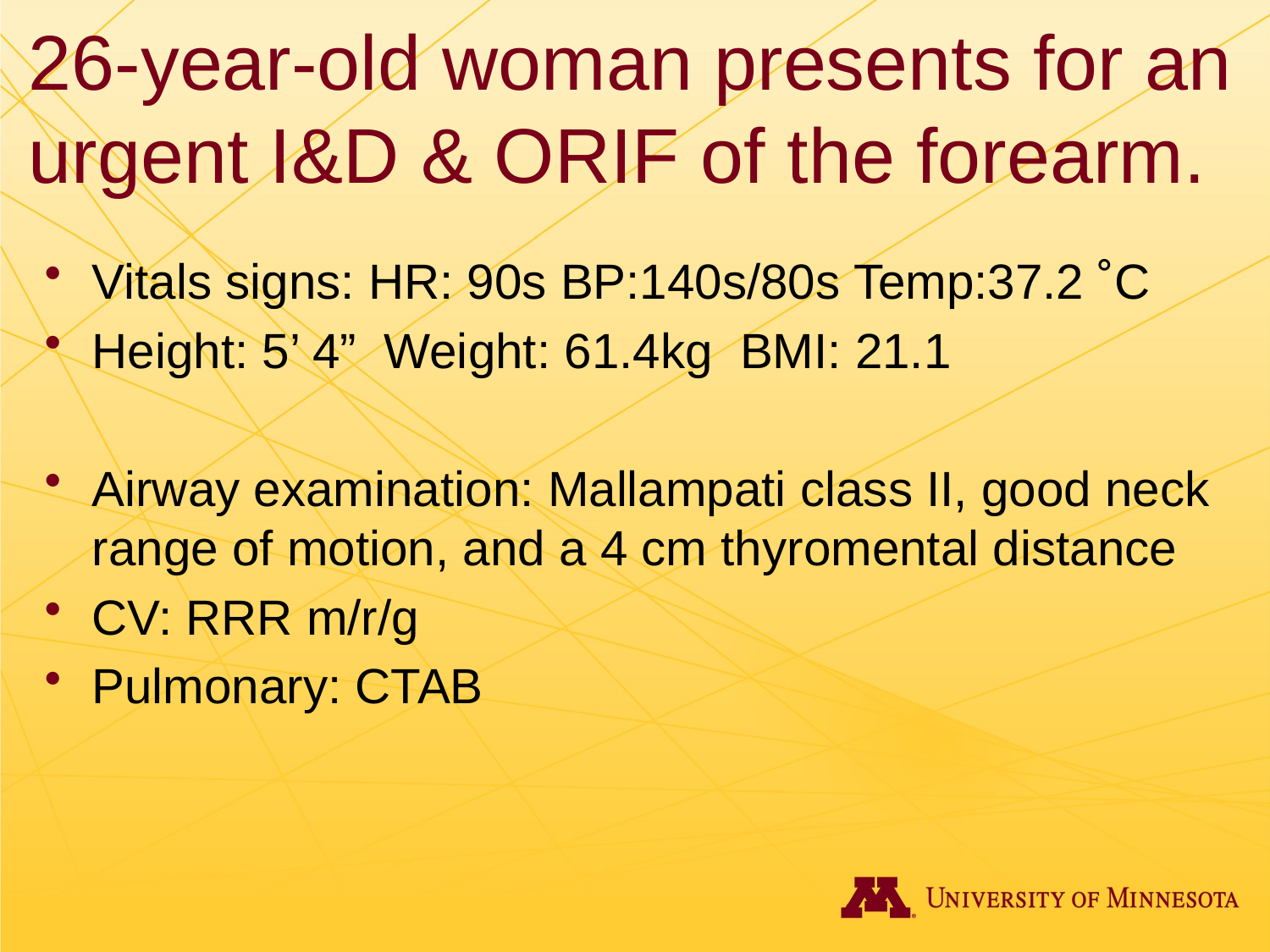

# 26-year-old woman presents for an urgent I&D & ORIF of the forearm.
Vitals signs: HR: 90s BP:140s/80s Temp:37.2 ˚C
Height: 5’ 4” Weight: 61.4kg BMI: 21.1
Airway examination: Mallampati class II, good neck range of motion, and a 4 cm thyromental distance
CV: RRR m/r/g
Pulmonary: CTAB

## Slide 13
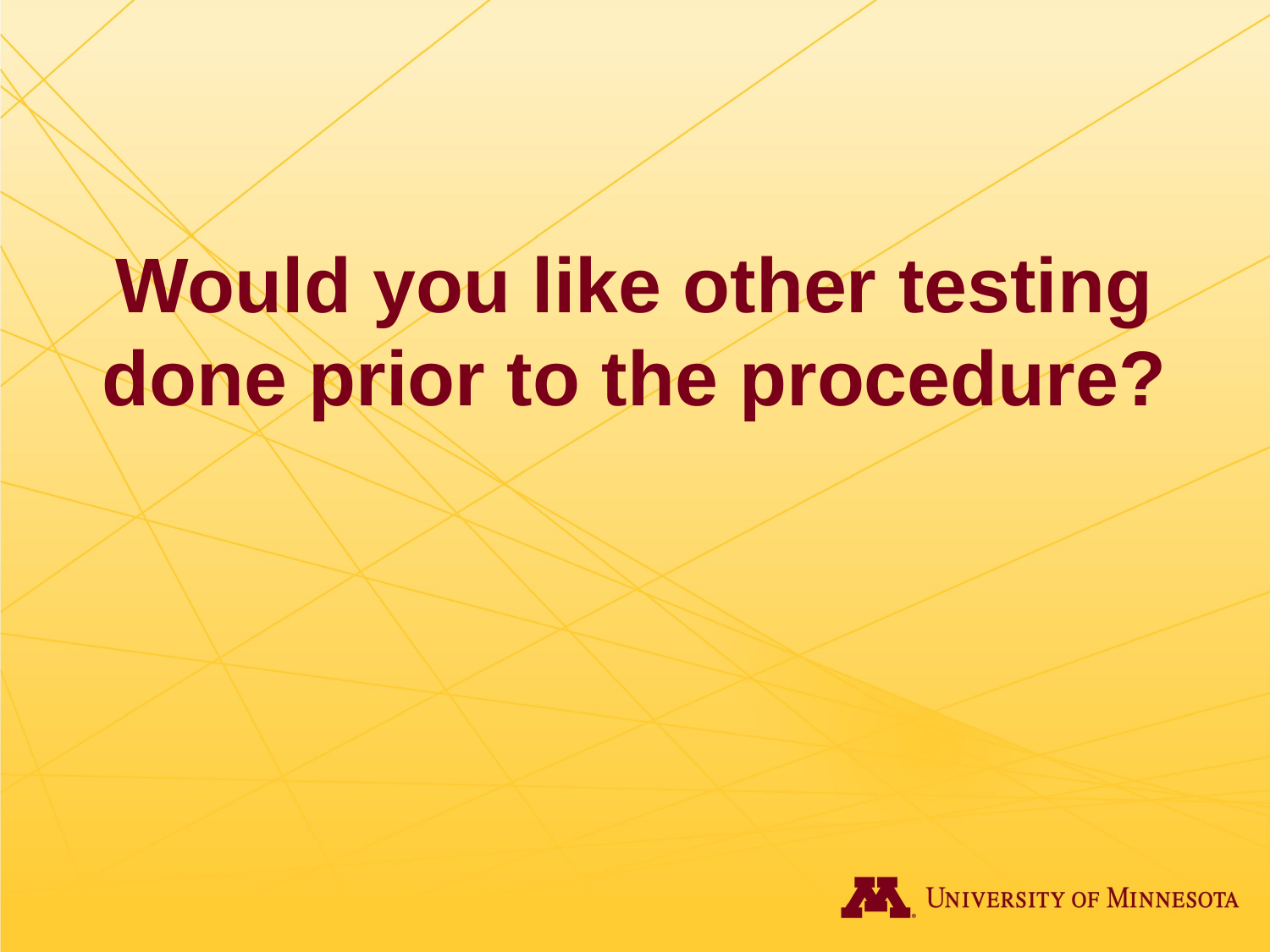

# Would you like other testing done prior to the procedure?

## Slide 14
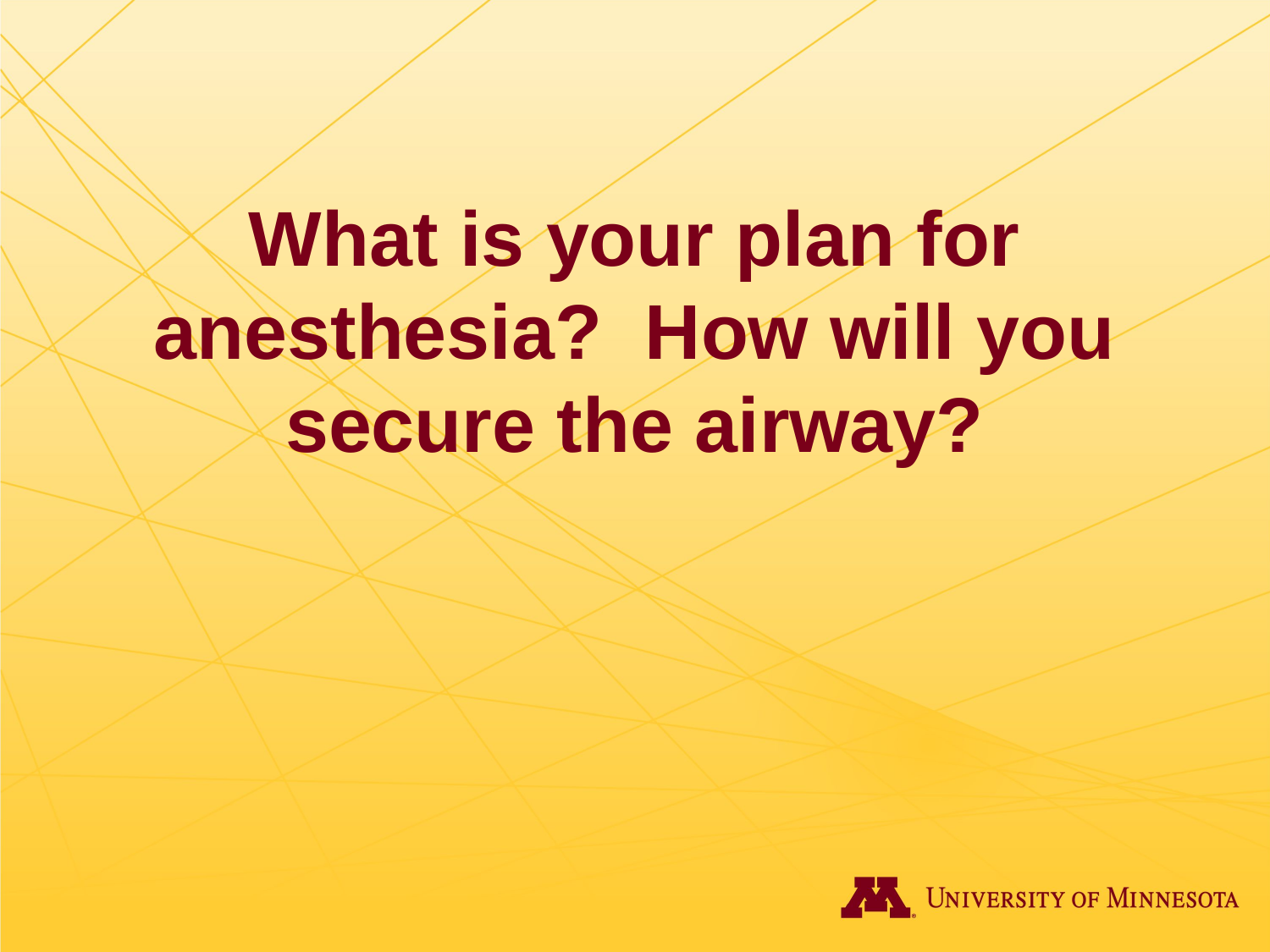

# What is your plan for anesthesia? How will you secure the airway?

## Slide 15
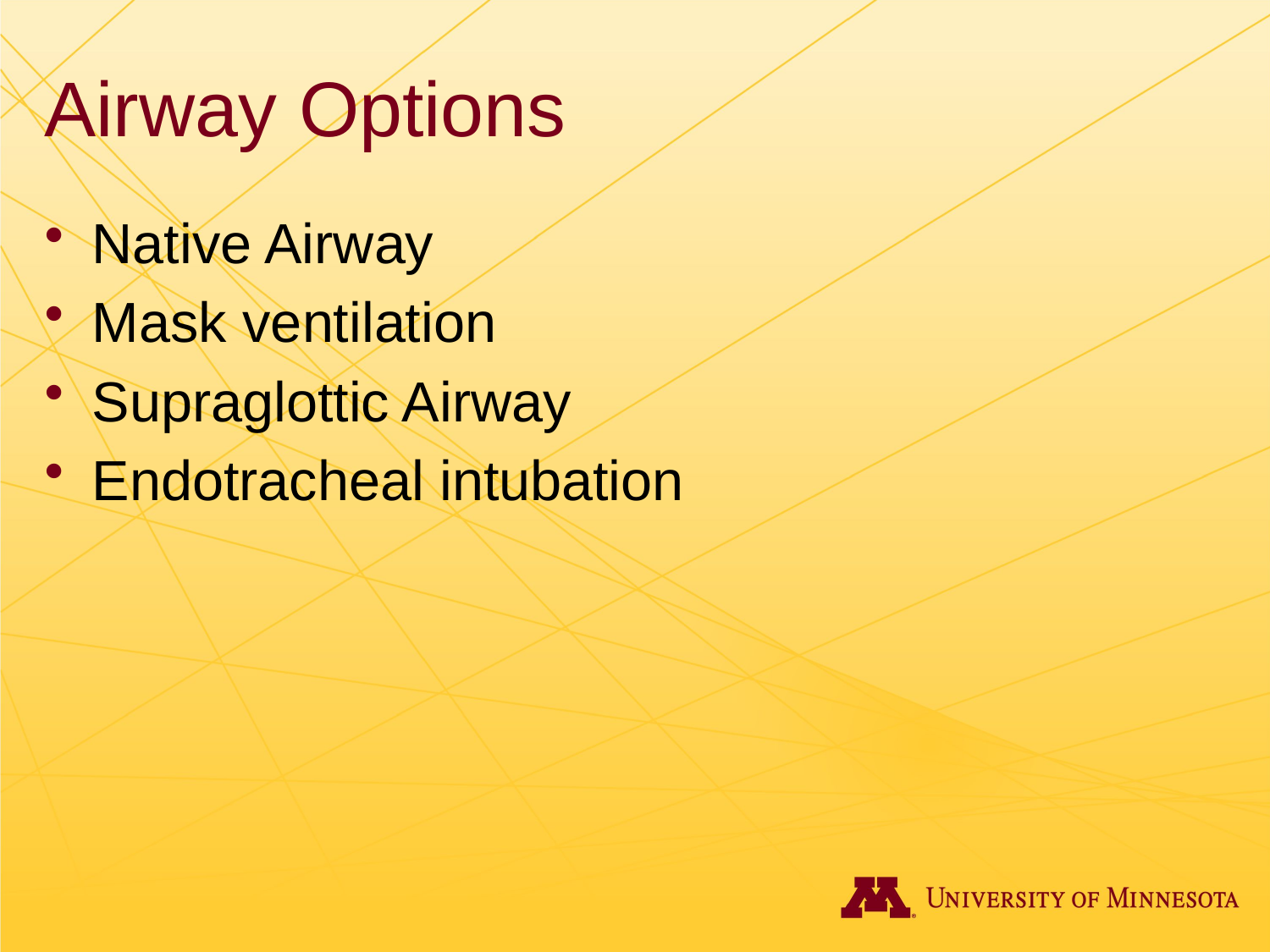

# Airway Options
Native Airway
Mask ventilation
Supraglottic Airway
Endotracheal intubation

## Slide 16
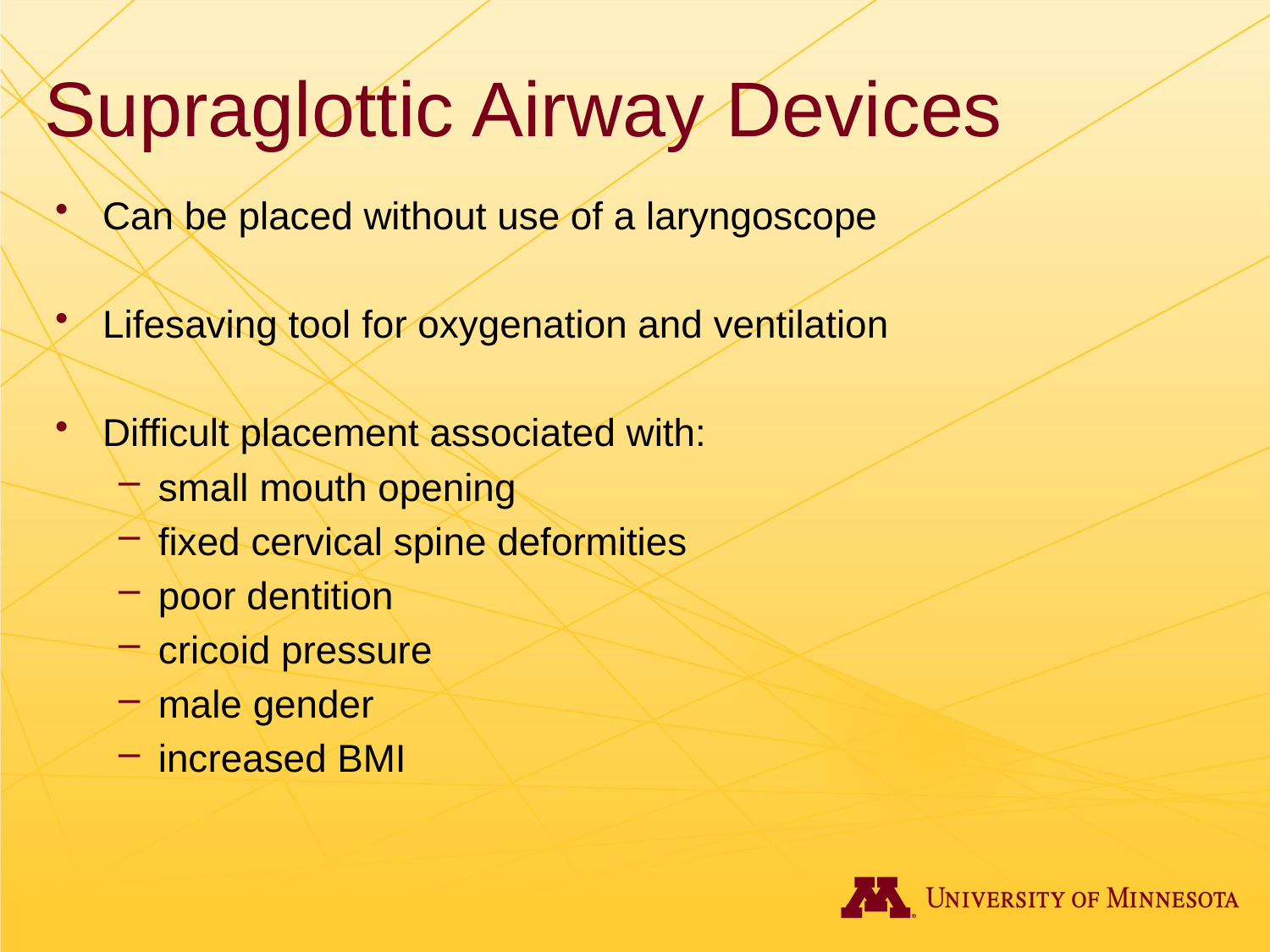

# Supraglottic Airway Devices
Can be placed without use of a laryngoscope
Lifesaving tool for oxygenation and ventilation
Difficult placement associated with:
small mouth opening
fixed cervical spine deformities
poor dentition
cricoid pressure
male gender
increased BMI

## Slide 17
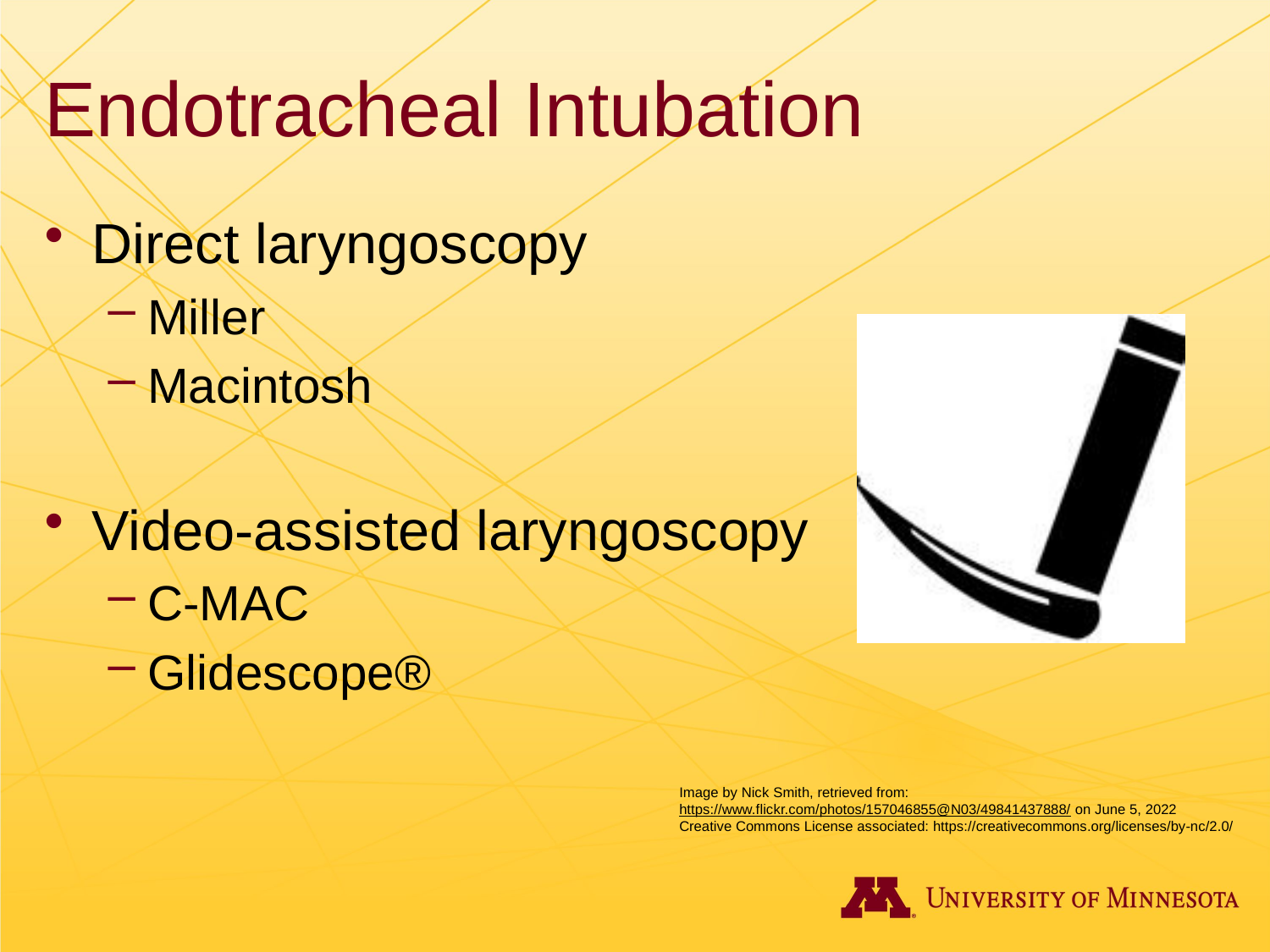

# Endotracheal Intubation
Direct laryngoscopy
Miller
Macintosh
Video-assisted laryngoscopy
C-MAC
Glidescope®
Image by Nick Smith, retrieved from:
https://www.flickr.com/photos/157046855@N03/49841437888/ on June 5, 2022
Creative Commons License associated: https://creativecommons.org/licenses/by-nc/2.0/

## Slide 18
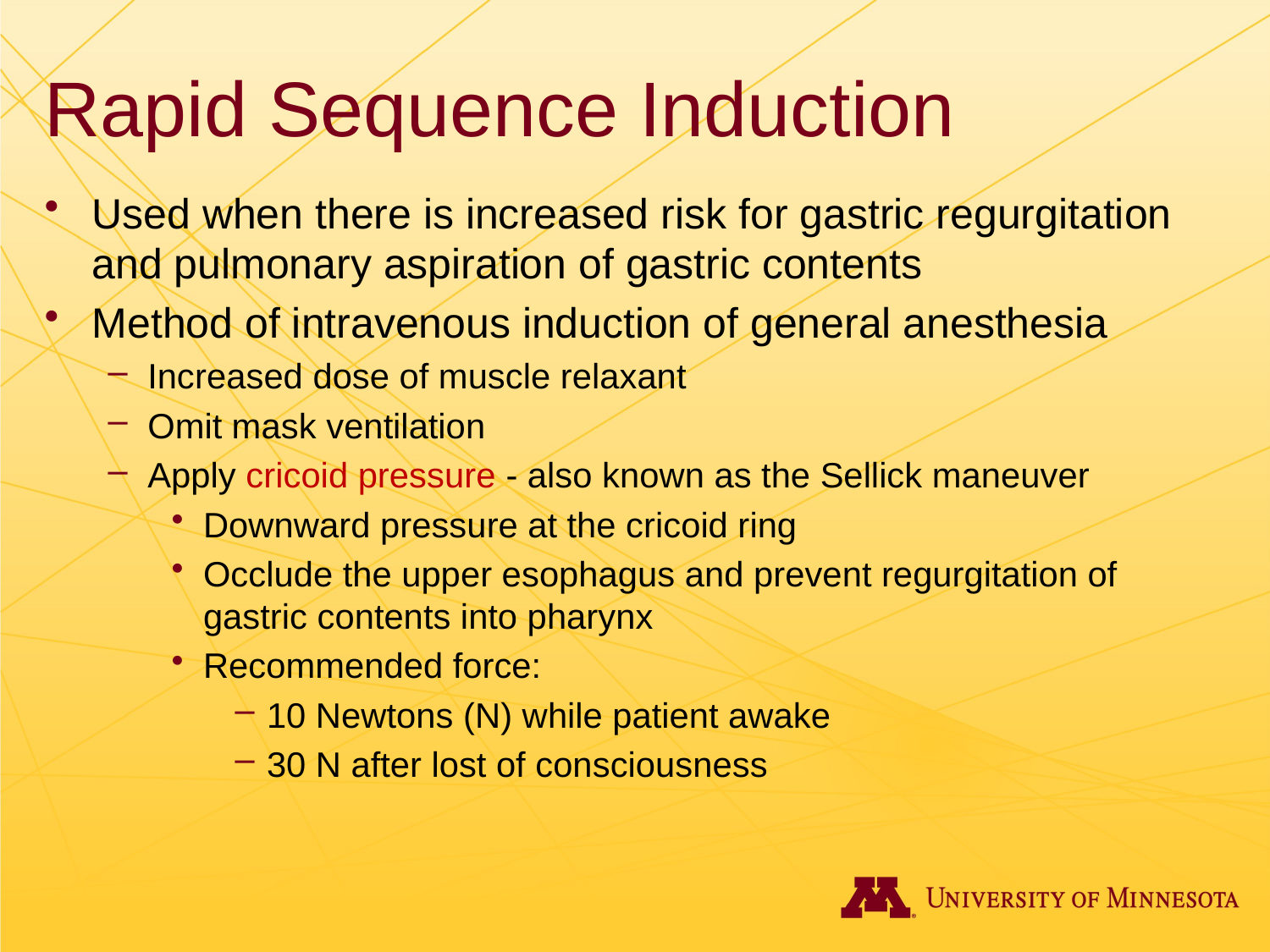

# Rapid Sequence Induction
Used when there is increased risk for gastric regurgitation and pulmonary aspiration of gastric contents
Method of intravenous induction of general anesthesia
Increased dose of muscle relaxant
Omit mask ventilation
Apply cricoid pressure - also known as the Sellick maneuver
Downward pressure at the cricoid ring
Occlude the upper esophagus and prevent regurgitation of gastric contents into pharynx
Recommended force:
10 Newtons (N) while patient awake
30 N after lost of consciousness

## Slide 19
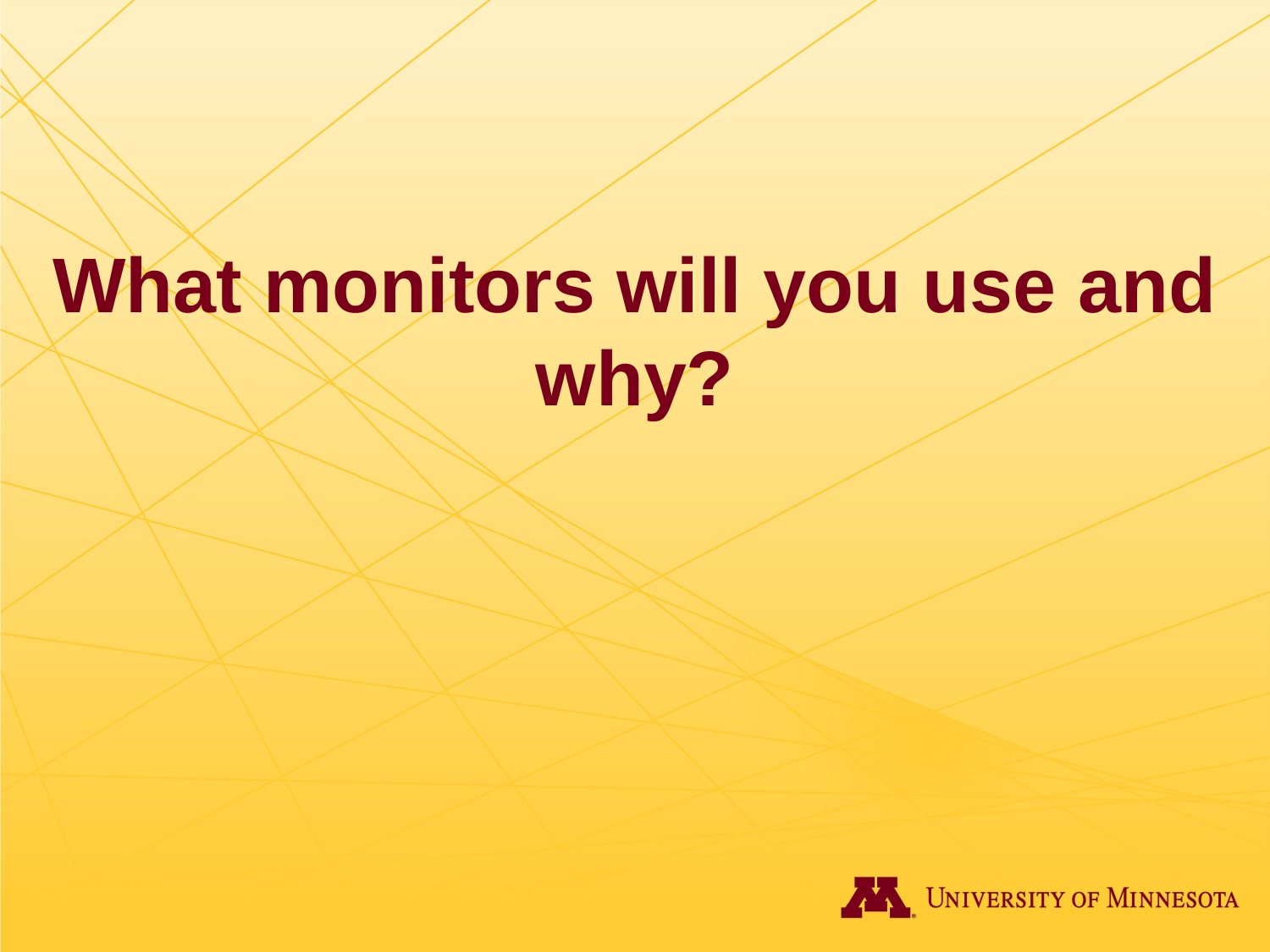

# What monitors will you use and why?

## Slide 20
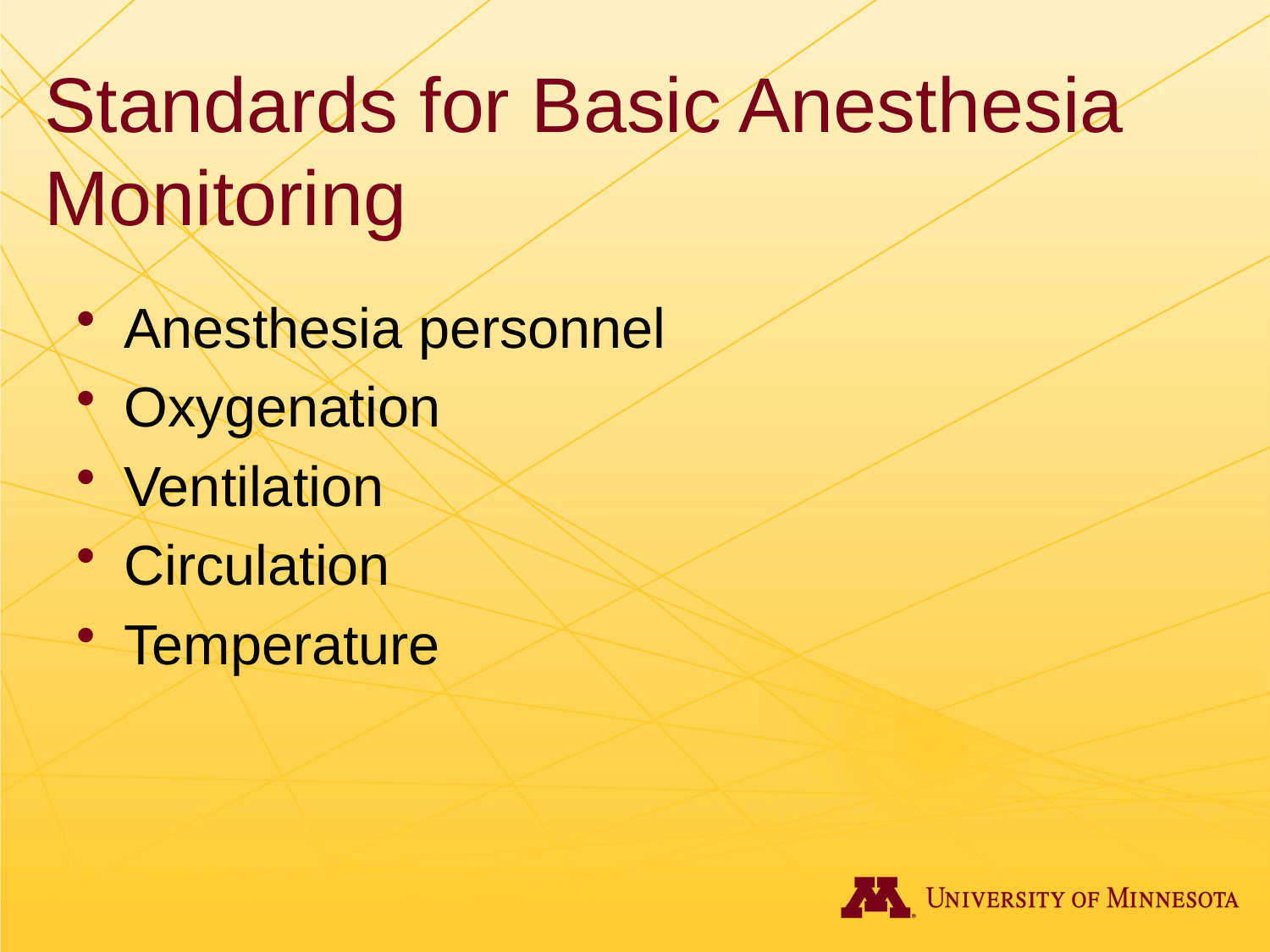

# Standards for Basic Anesthesia Monitoring
Anesthesia personnel
Oxygenation
Ventilation
Circulation
Temperature

## Slide 21
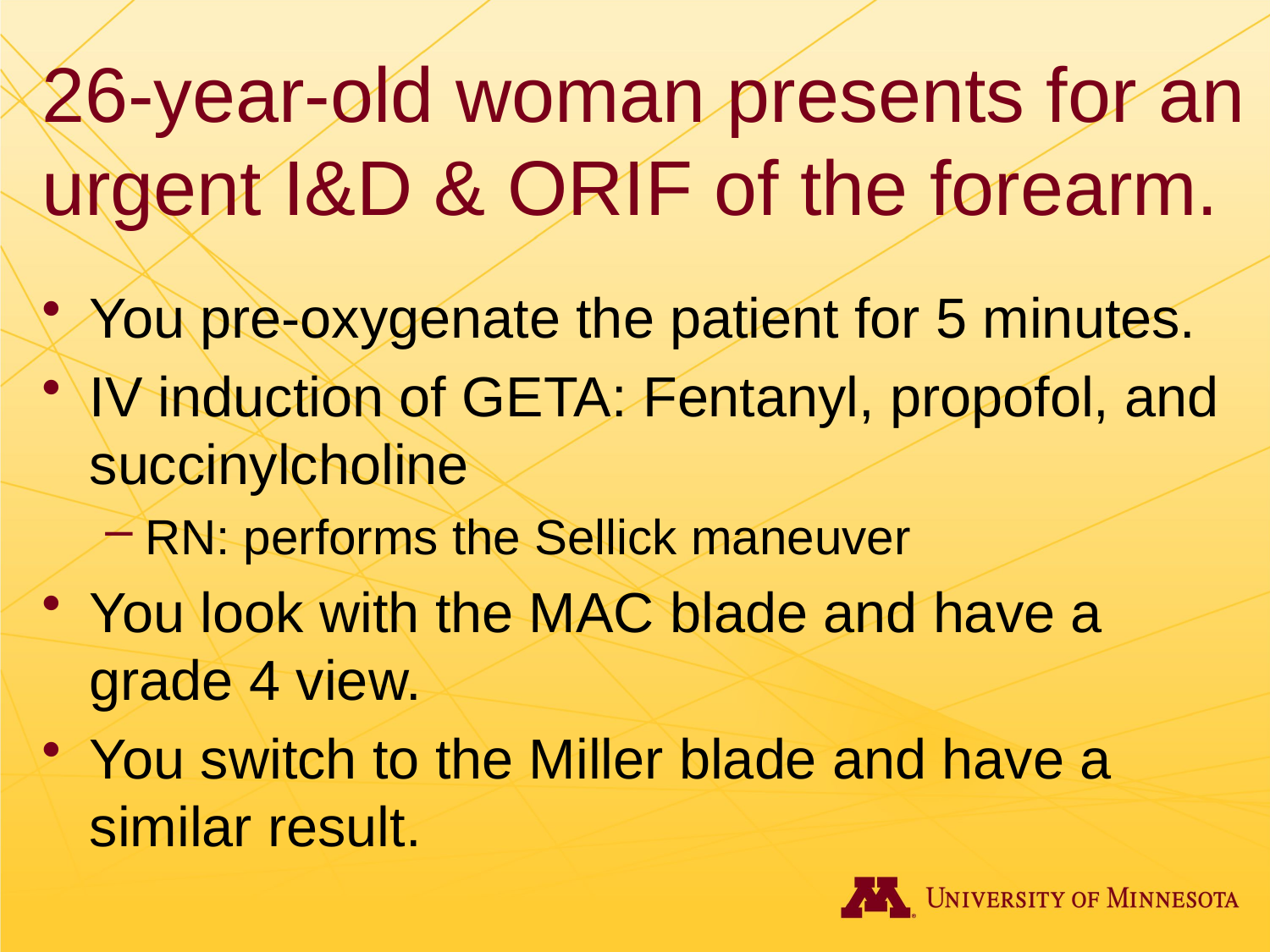

# 26-year-old woman presents for an urgent I&D & ORIF of the forearm.
You pre-oxygenate the patient for 5 minutes.
IV induction of GETA: Fentanyl, propofol, and succinylcholine
RN: performs the Sellick maneuver
You look with the MAC blade and have a grade 4 view.
You switch to the Miller blade and have a similar result.

## Slide 22
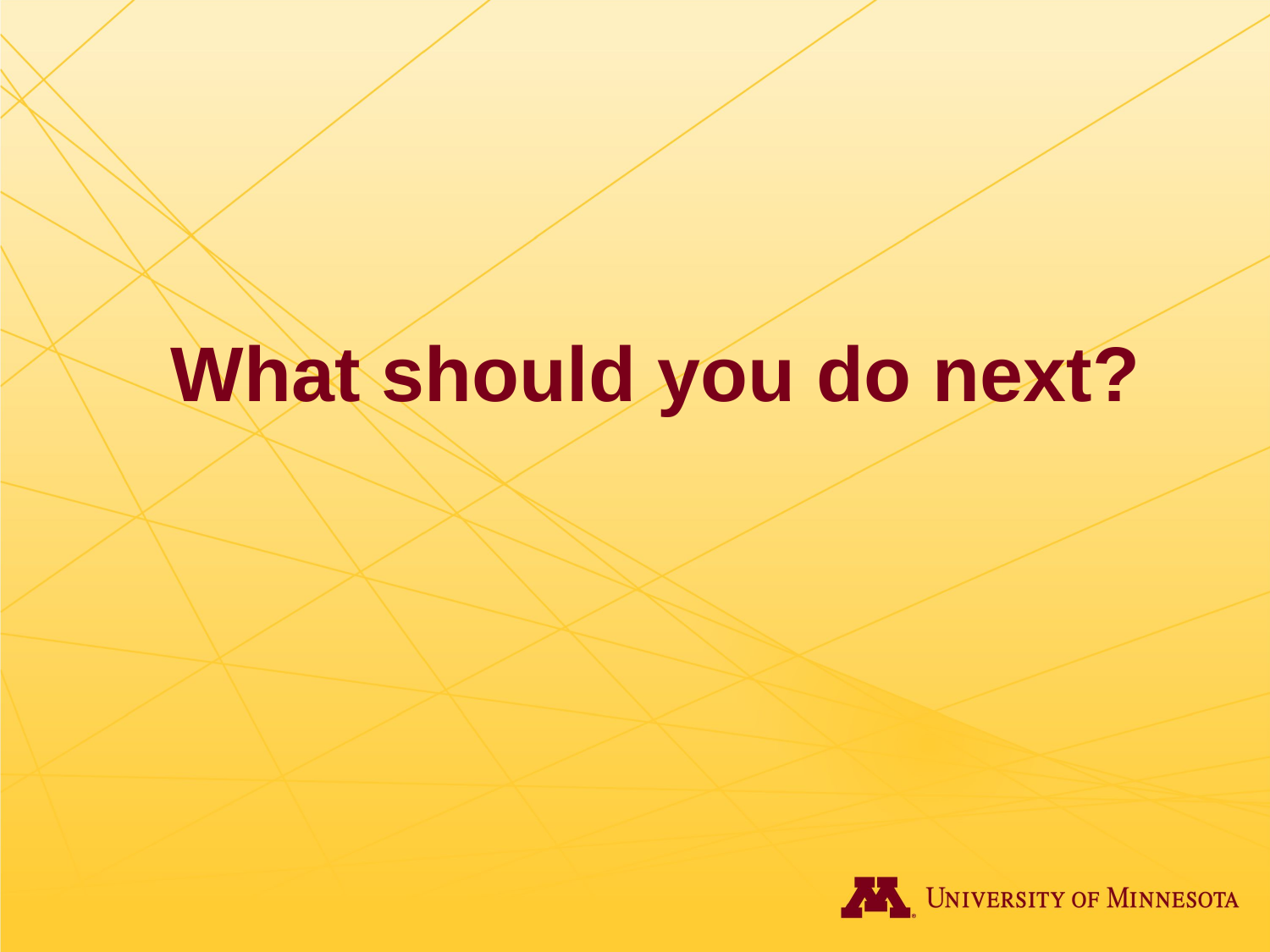

# What should you do next?

## Slide 23
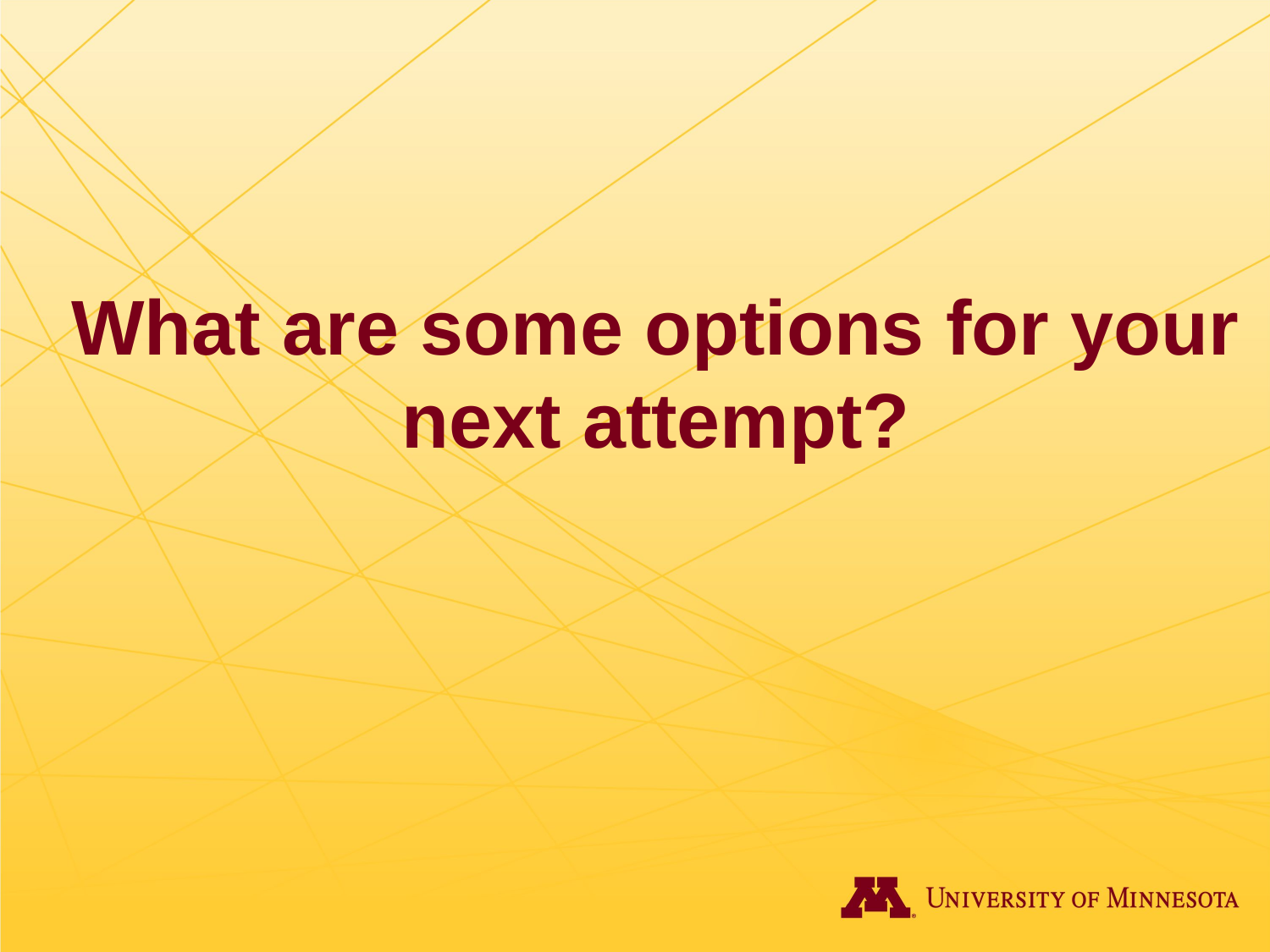

# What are some options for your next attempt?

## Slide 24
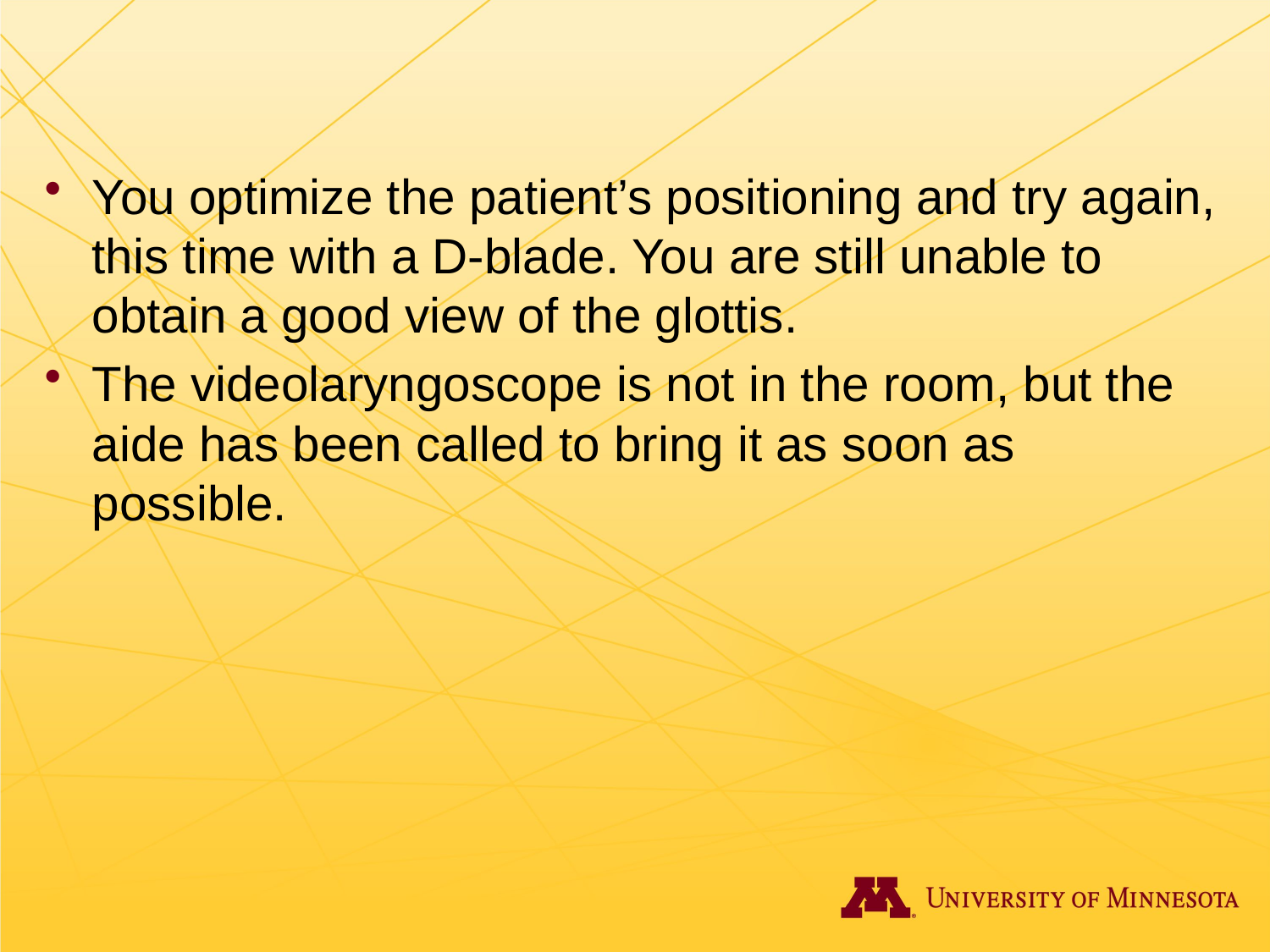

You optimize the patient’s positioning and try again, this time with a D-blade. You are still unable to obtain a good view of the glottis.
The videolaryngoscope is not in the room, but the aide has been called to bring it as soon as possible.

## Slide 25
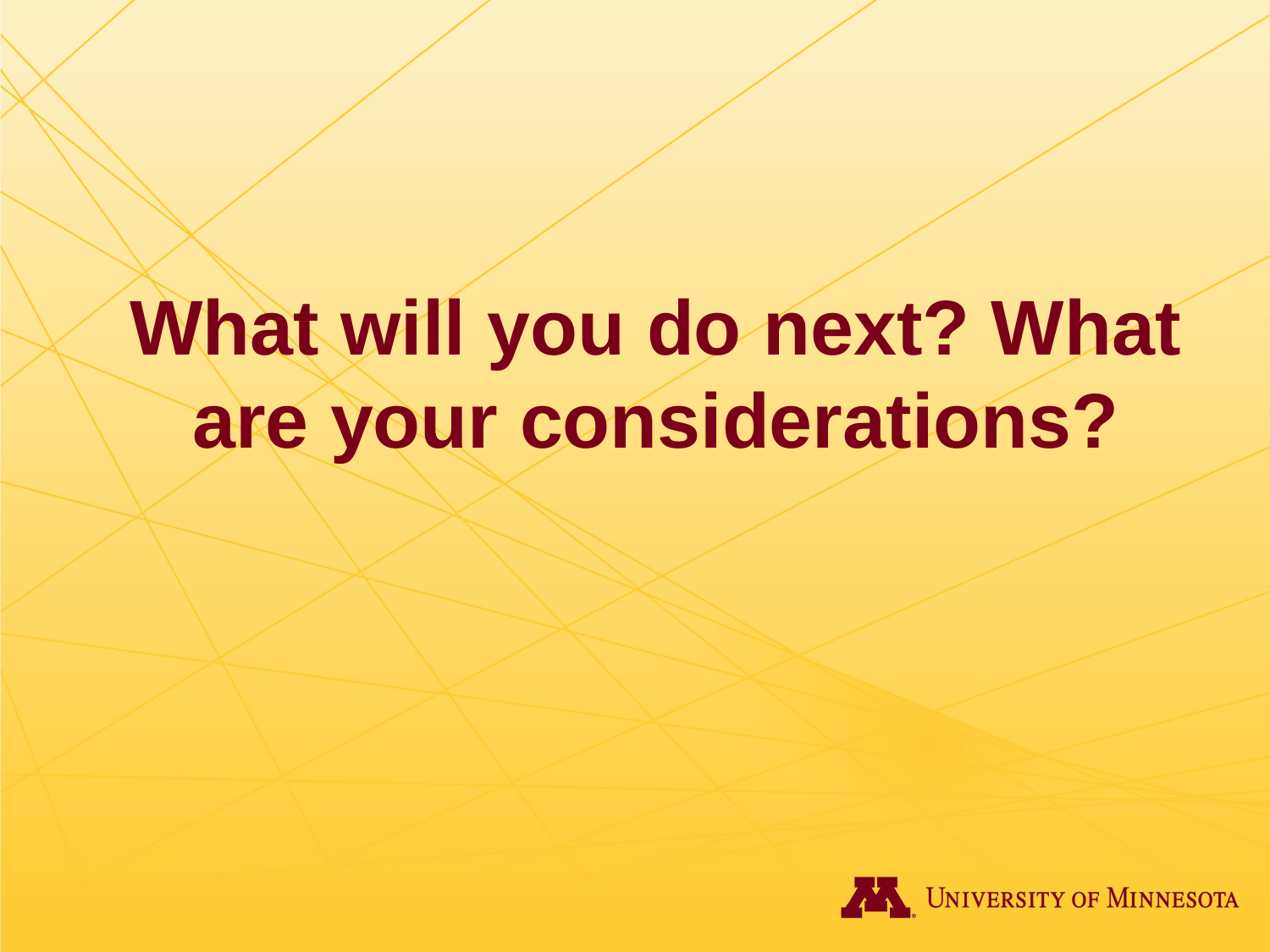

# What will you do next? What are your considerations?

## Slide 26
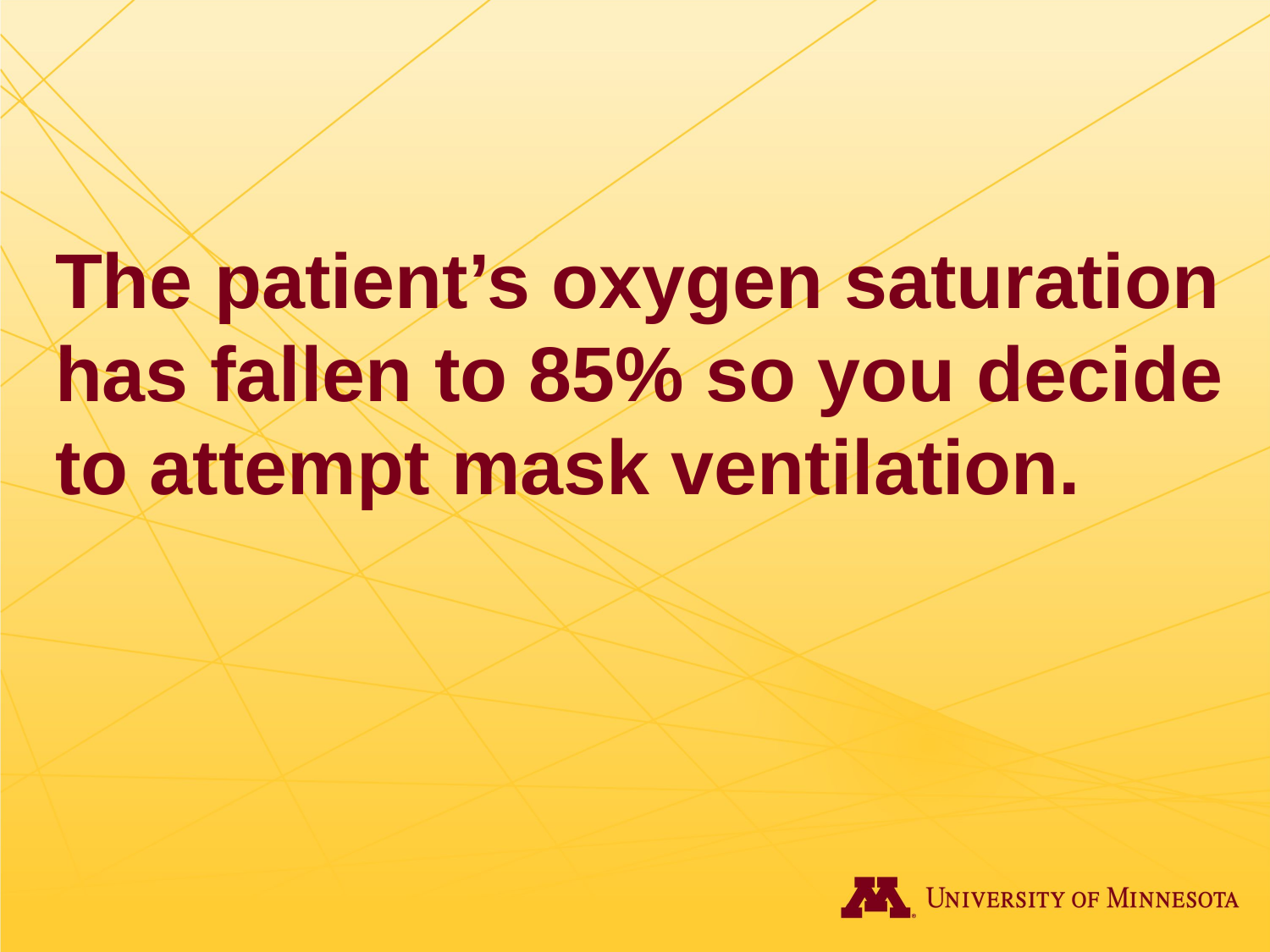

# The patient’s oxygen saturation has fallen to 85% so you decide to attempt mask ventilation.

## Slide 27
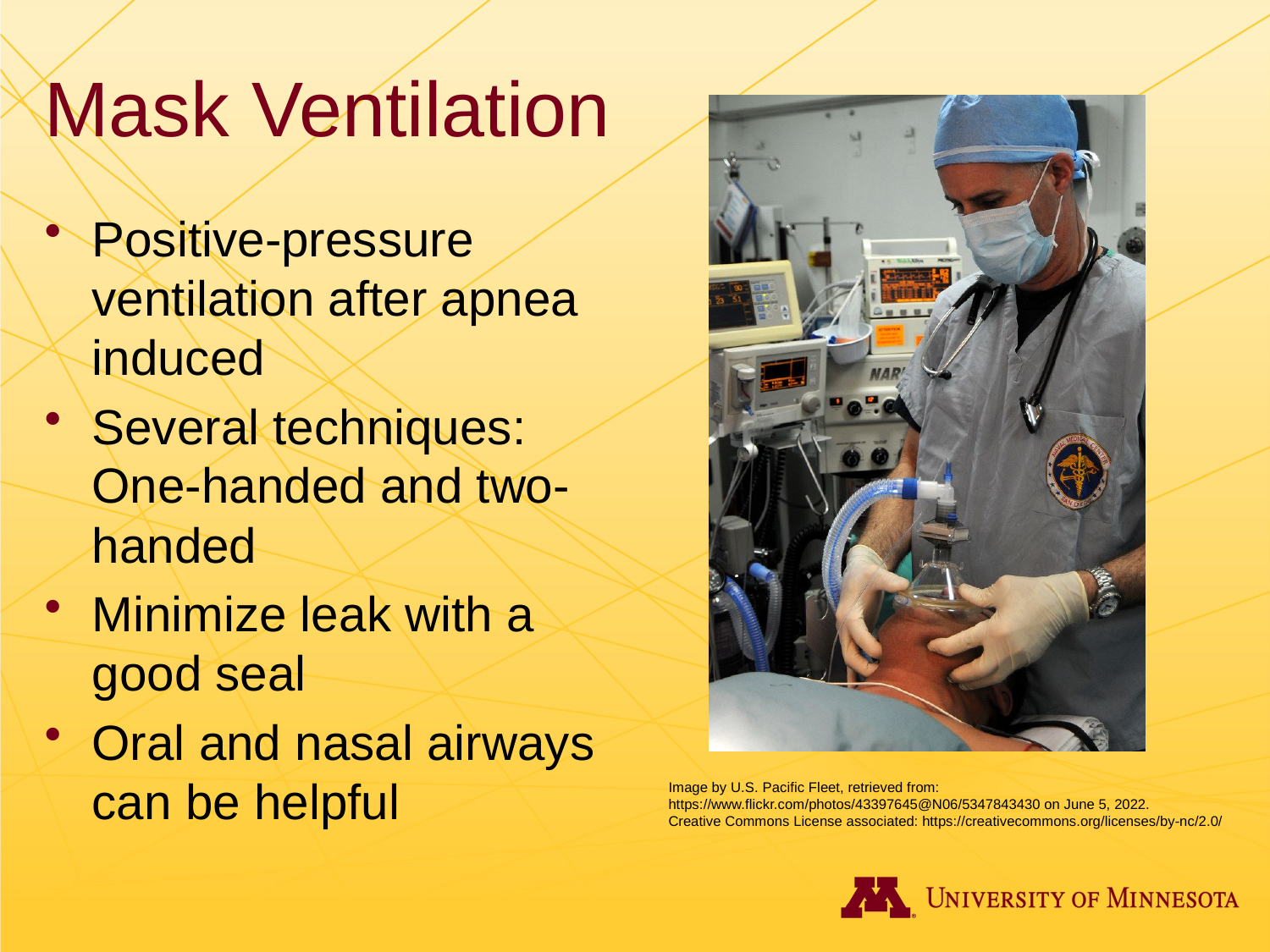

# Mask Ventilation
Positive-pressure ventilation after apnea induced
Several techniques: One-handed and two-handed
Minimize leak with a good seal
Oral and nasal airways can be helpful
Image by U.S. Pacific Fleet, retrieved from:
https://www.flickr.com/photos/43397645@N06/5347843430 on June 5, 2022.
Creative Commons License associated: https://creativecommons.org/licenses/by-nc/2.0/

## Slide 28
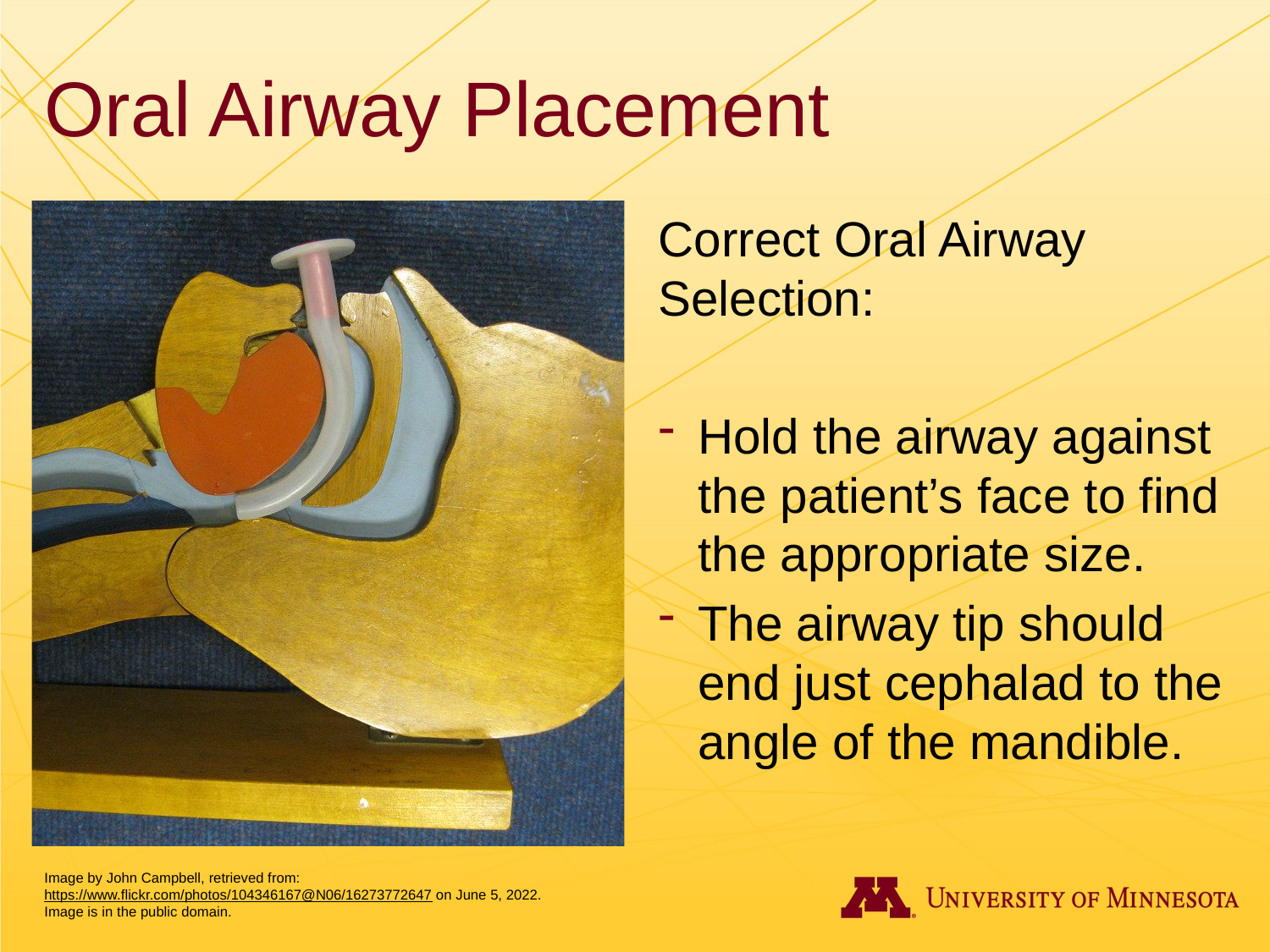

# Oral Airway Placement
Correct Oral Airway Selection:
Hold the airway against the patient’s face to find the appropriate size.
The airway tip should end just cephalad to the angle of the mandible.
Image by John Campbell, retrieved from:
https://www.flickr.com/photos/104346167@N06/16273772647 on June 5, 2022.
Image is in the public domain.

## Slide 29
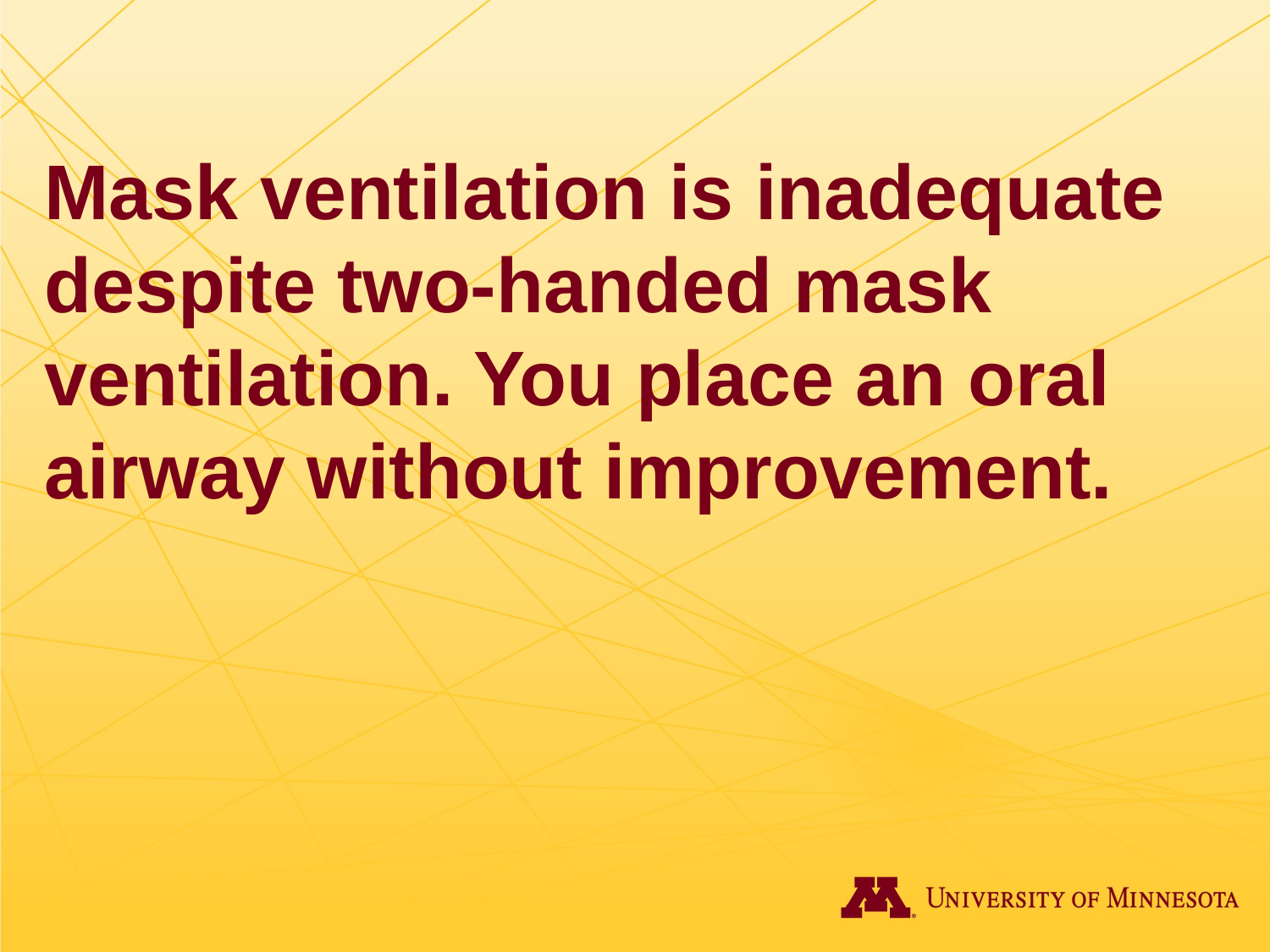

# Mask ventilation is inadequate despite two-handed mask ventilation. You place an oral airway without improvement.

## Slide 30
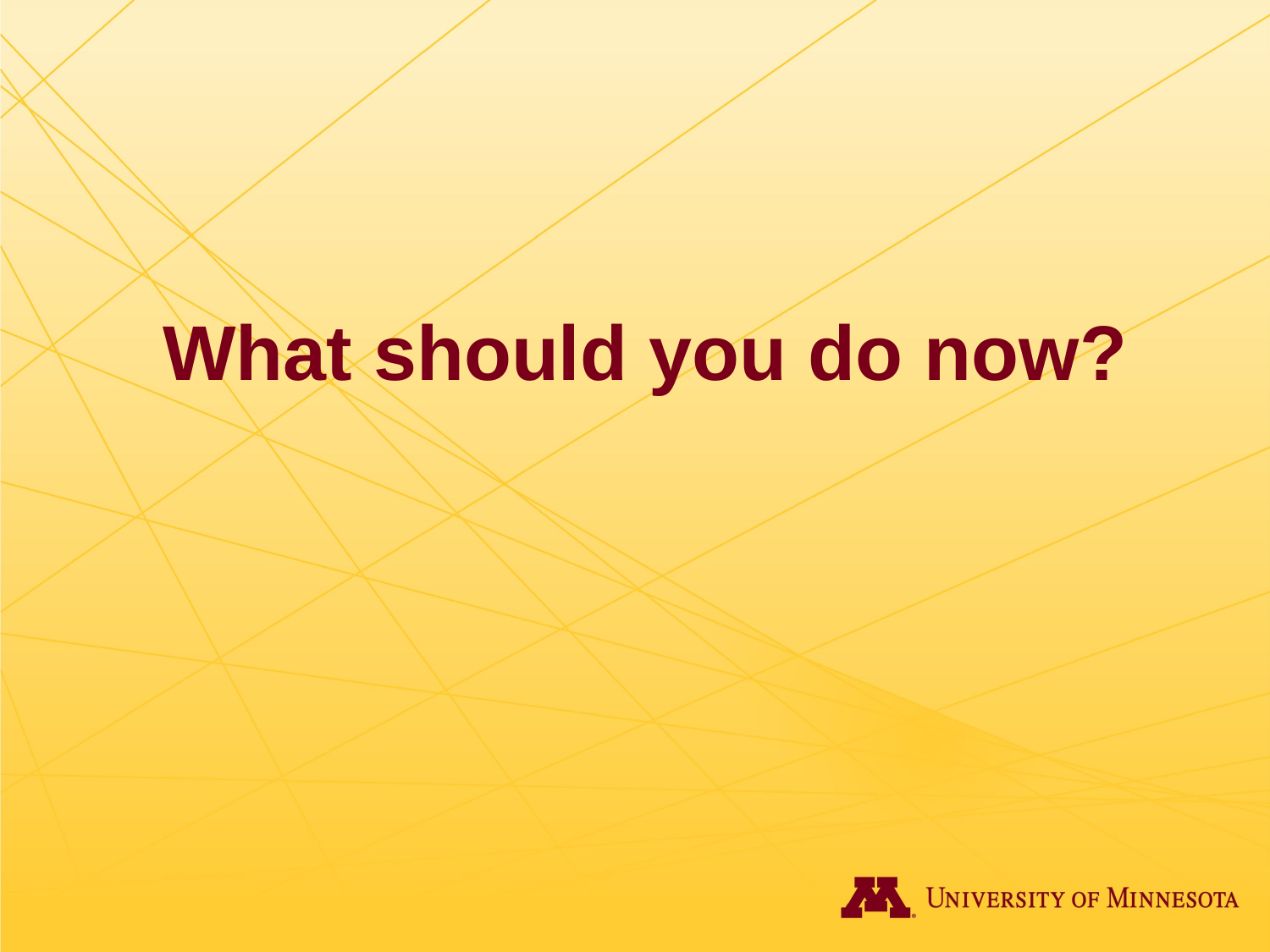

# What should you do now?

## Slide 31
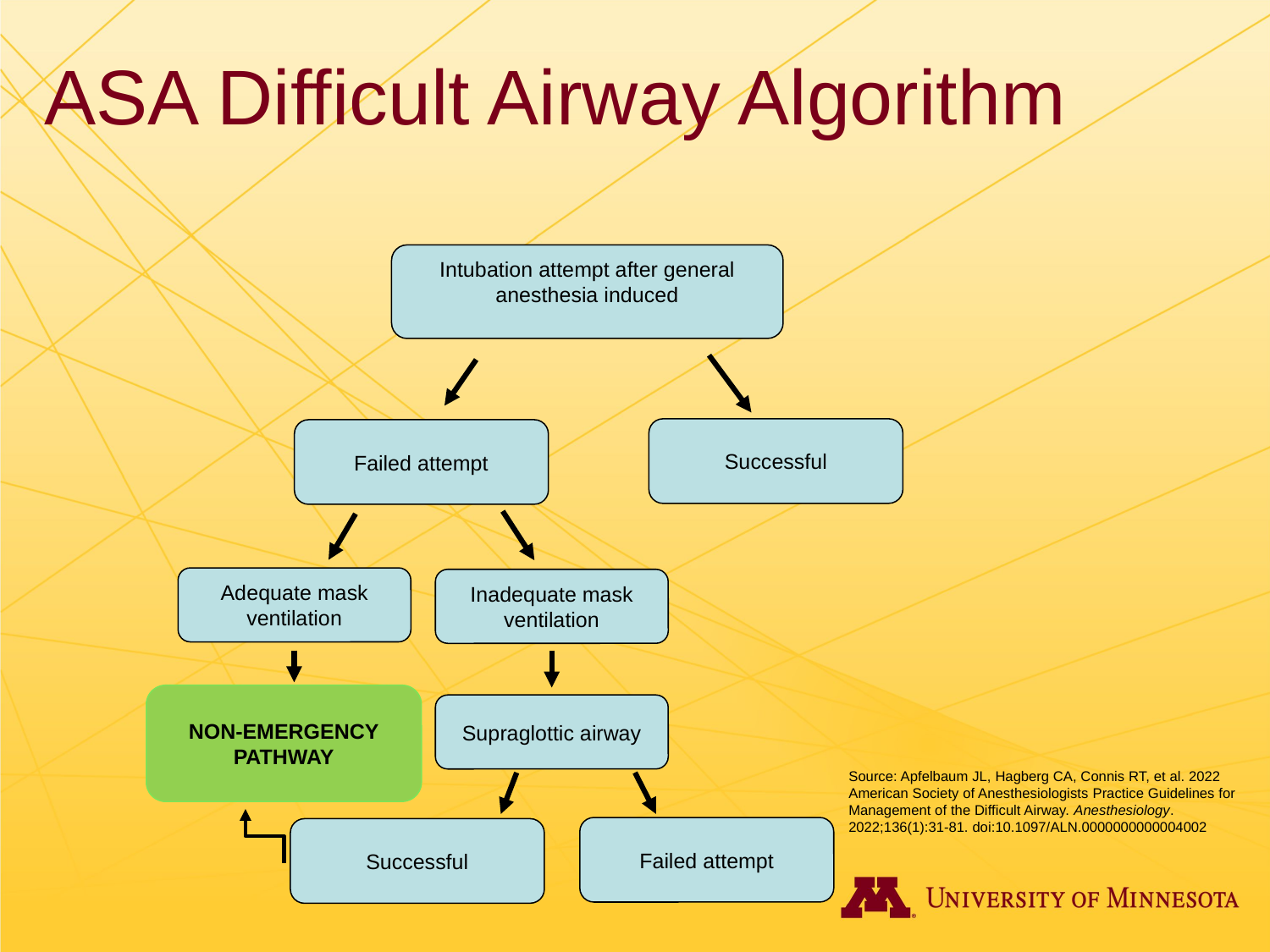

# ASA Difficult Airway Algorithm
Intubation attempt after general anesthesia induced
Successful
Failed attempt
Adequate mask ventilation
Inadequate mask ventilation
NON-EMERGENCY PATHWAY
Supraglottic airway
Source: Apfelbaum JL, Hagberg CA, Connis RT, et al. 2022 American Society of Anesthesiologists Practice Guidelines for Management of the Difficult Airway. Anesthesiology. 2022;136(1):31-81. doi:10.1097/ALN.0000000000004002
Failed attempt
Successful

## Slide 32
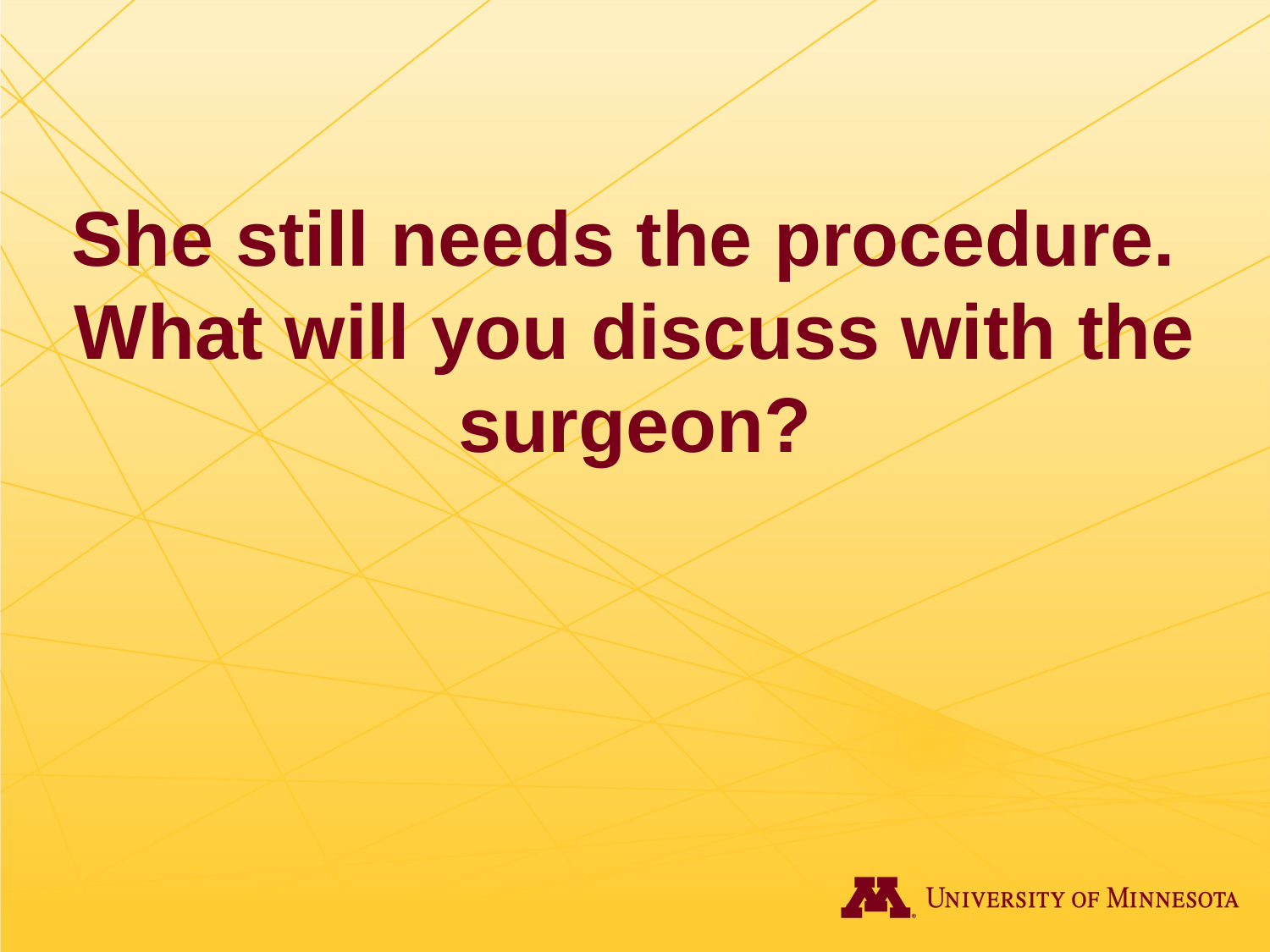

# She still needs the procedure. What will you discuss with the surgeon?

## Slide 33
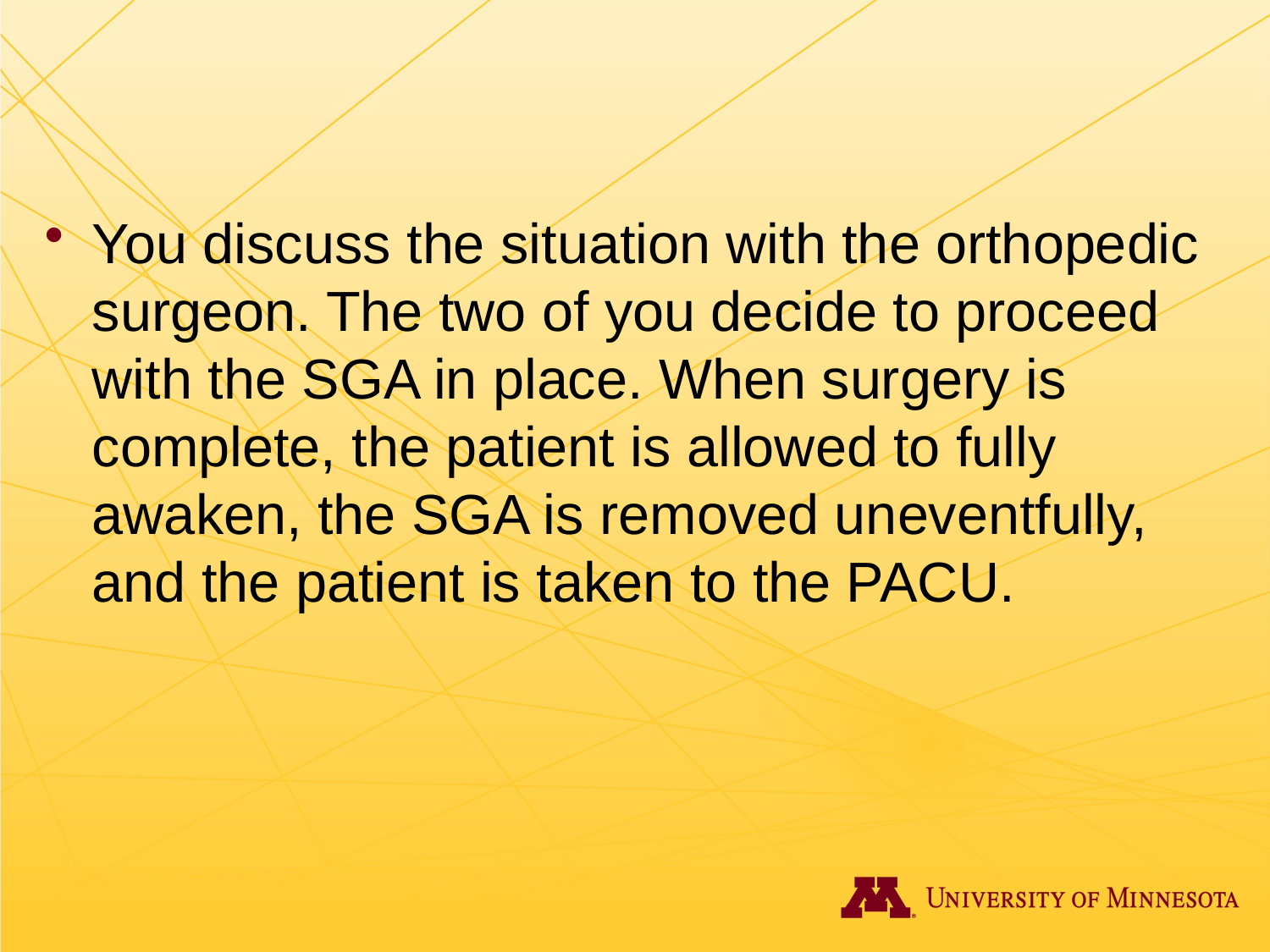

You discuss the situation with the orthopedic surgeon. The two of you decide to proceed with the SGA in place. When surgery is complete, the patient is allowed to fully awaken, the SGA is removed uneventfully, and the patient is taken to the PACU.

## Slide 34
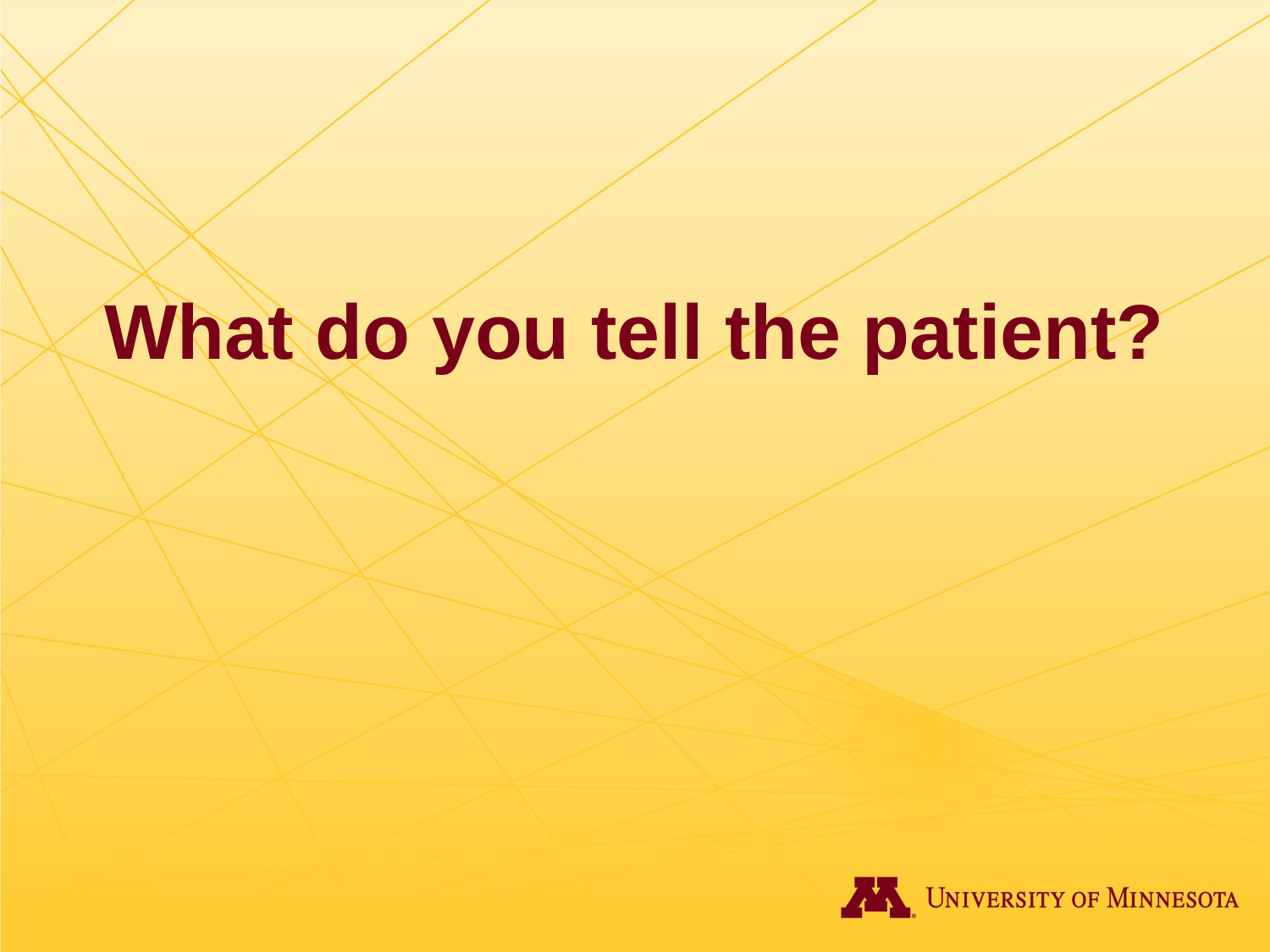

# What do you tell the patient?

## Slide 35
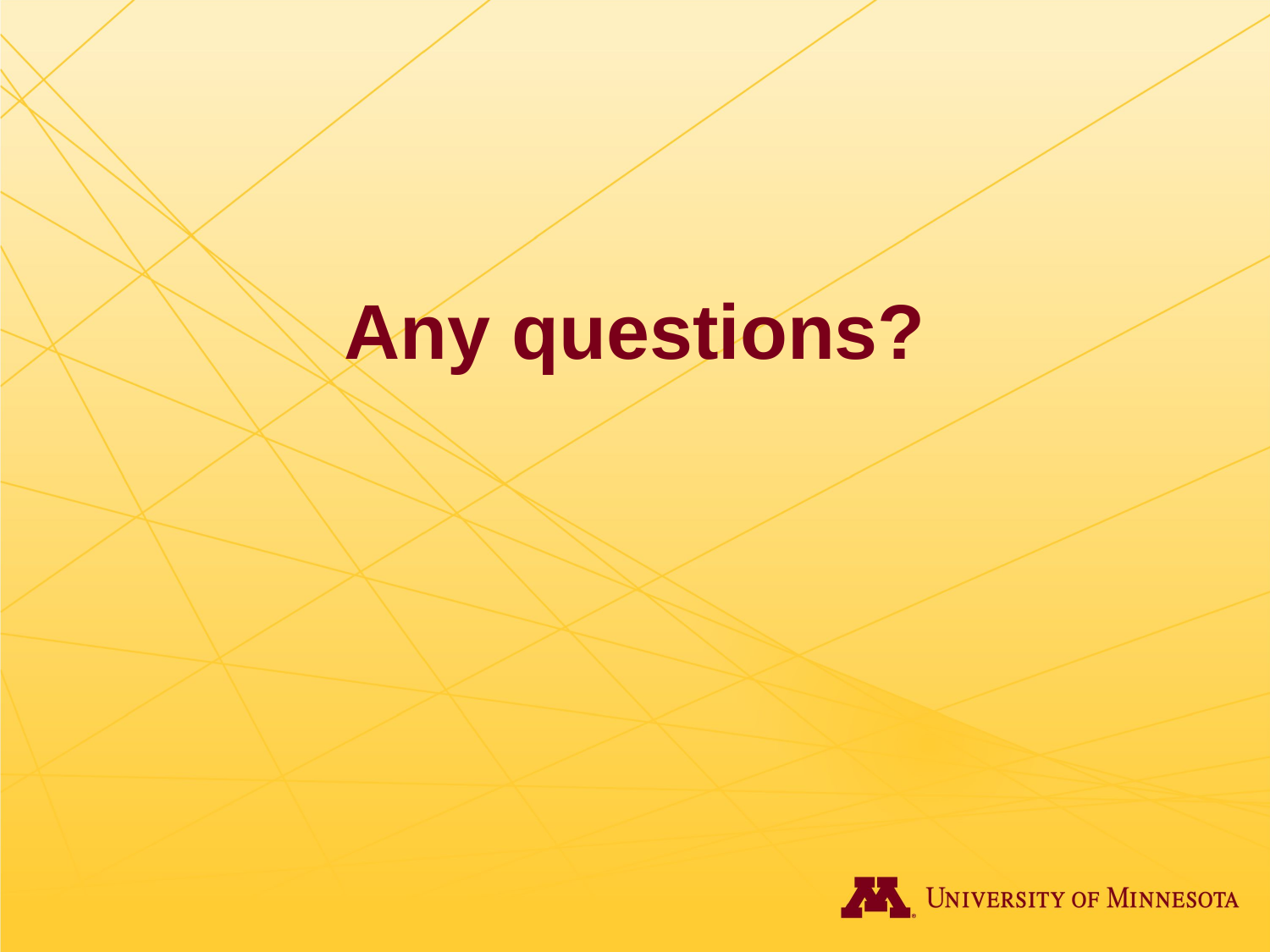

# Any questions?

## Slide 36
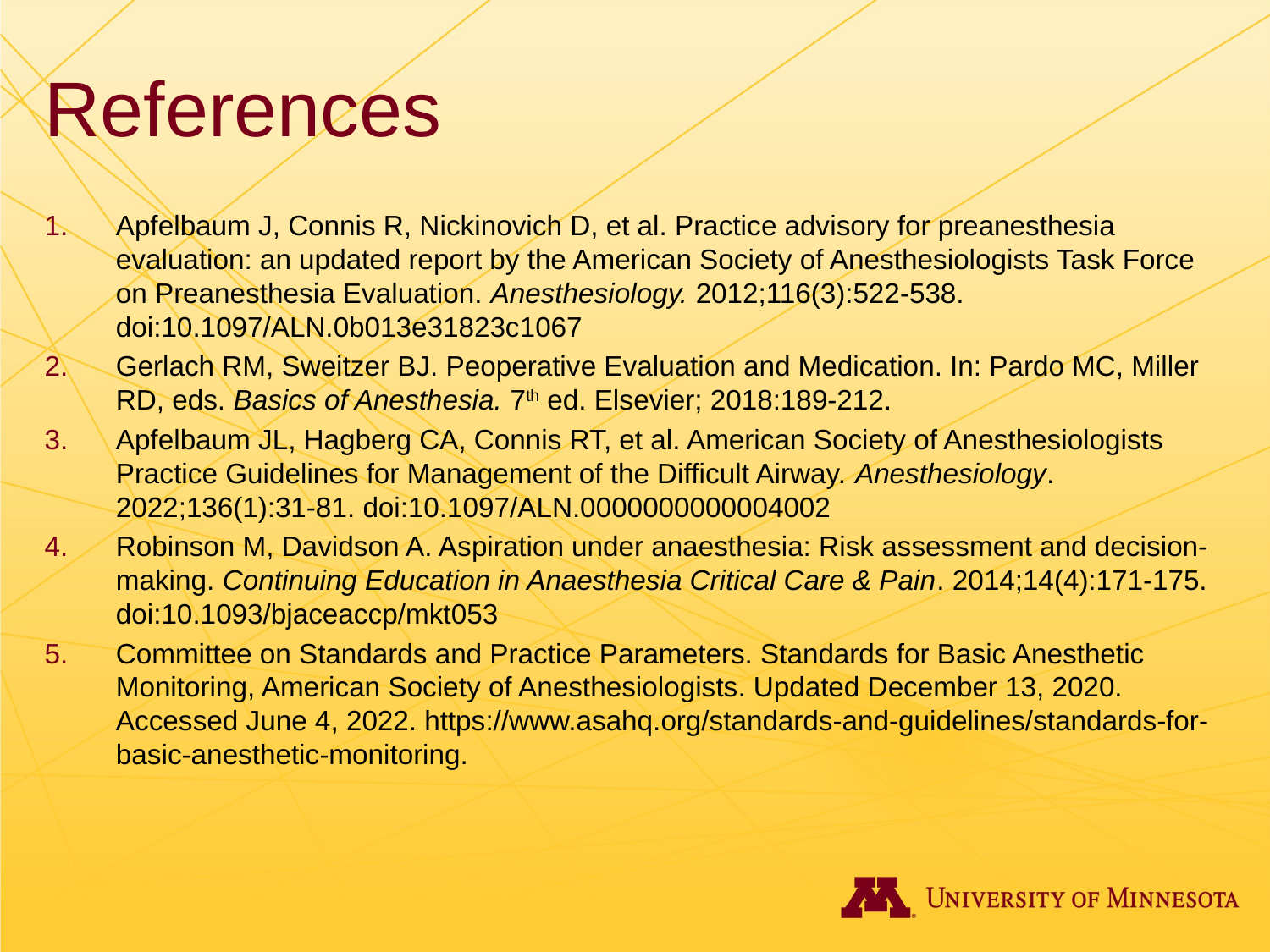

# References
Apfelbaum J, Connis R, Nickinovich D, et al. Practice advisory for preanesthesia evaluation: an updated report by the American Society of Anesthesiologists Task Force on Preanesthesia Evaluation. Anesthesiology. 2012;116(3):522-538. doi:10.1097/ALN.0b013e31823c1067
Gerlach RM, Sweitzer BJ. Peoperative Evaluation and Medication. In: Pardo MC, Miller RD, eds. Basics of Anesthesia. 7th ed. Elsevier; 2018:189-212.
Apfelbaum JL, Hagberg CA, Connis RT, et al. American Society of Anesthesiologists Practice Guidelines for Management of the Difficult Airway. Anesthesiology. 2022;136(1):31-81. doi:10.1097/ALN.0000000000004002
Robinson M, Davidson A. Aspiration under anaesthesia: Risk assessment and decision-making. Continuing Education in Anaesthesia Critical Care & Pain. 2014;14(4):171-175. doi:10.1093/bjaceaccp/mkt053
Committee on Standards and Practice Parameters. Standards for Basic Anesthetic Monitoring, American Society of Anesthesiologists. Updated December 13, 2020. Accessed June 4, 2022. https://www.asahq.org/standards-and-guidelines/standards-for-basic-anesthetic-monitoring.
