# Supplementary material for: Development and Implementation of Video-Recorded Simulation Scenarios to Facilitate Case-Based Learning Discussions for Medical Students' Virtual Anesthesiology Clerkship
Source: MedEdPORTAL. 2023 Apr 4;19:11306. doi: 10.15766/mep_2374-8265.11306 (PMC10070881; doi:10.15766/mep_2374-8265.11306)
Supplement: Supplementary file 1 — Preoperative Evaluation - CBLD 1.pptxInhaled and Intravenous Anesthetics - CBLD 2.pptxAirway Management - CBLD 3.pptxScenario 1.mp4Scenario 2.mp4Scenario 3.mp4Scenario Debrief 1.docxScenario Debrief 2.docxScenario Debrief 3.docxClerkship Survey Questions.docxCBLD-Specific Survey Questions.docx [file mep_2374-8265.11306-s001.zip › G. Scenario Debrief 1.docx]

**Appendix G**

**Anesthesiology Simulation Scenario No 1: Intraoperative Anaphylaxis**

**Video and Debriefing Guide**

**Prebrief:**

Simulations attempt to approximate a real-life operating room setting. Limitations exist, however, including the use of a mannequin, actors not carrying out the actions they say they are doing, and poor use of sterile technique. The goal of the simulation video is to give learners a glimpse into the OR setting and an opportunity to discuss key features of anesthetic management.

[Play]

**This is a 28 y/o healthy man who presented to the emergency department with abdominal pain and N/V. He is diagnosed with appendicitis and presents for laparoscopic appendectomy.**

***Video Time 00:13 -What other information do you want to know about the patient?***

[Stop]

Important information includes past medical history, surgical history, and social history with a focus on alcohol use, illicit drug use, and smoking history. Ascertaining whether there is a family history of problems with anesthesia or bleeding problems is important to screen out a possibility of heritable conditions such as malignant hyperthermia, pseudocholinesterase deficiency, or bleeding disorders.^1^

Other important information includes whether the patient has any allergies and any current medications.

Key physical exam findings include current vitals, trends in vitals, airway exam, cardiac and pulmonary exam. The number and types of vascular access should be noted as well.

[Play]

**Induction of Anesthesia was uneventful. We pick up right before the surgery starts.**

***Video Time 01:58 -What do you think is going on? How would you proceed?***

[Stop]

The differential diagnosis for hypotension with tachycardia includes myocardial ischemia, cardiac arrhythmias, pulmonary embolism, hemorrhage, sepsis, hypovolemia, anaphylaxis, and iatrogenic causes.^2^ In this scenario, the patient was administered opioids to blunt the sympathetic response to surgical stimulation. Given his subsequent development of mild hypotension with tachycardia, supporting the blood pressure with a fluid bolus and a mild vasoactive agent (such as phenylephrine, an alpha 1 agonist) is an appropriate step.

[Play]

***Video Time 02:19 -What is your Differential Diagnosis?***

[Stop]

There are various causes of an intraoperative rash.^3^ Most of the lesions are plaques with some maculopapular lesions. A broad differential diagnosis for this type of rash include contact dermatitis, cellulitis, fixed drug eruption, urticaria, exanthemas, bug-bite like reaction, Lyme disease, & erythema multiforme.^4^

[Play]

***Video Time 2:41 -How will you investigate the low oxygen desaturations? What do you make of the CO2 waveform?***

[Stop]

Initial steps include auscultating for breath sounds while hand ventilating to obtain further diagnostic information. Disconnections of the circuit from the endotracheal tube (ETT) will become apparent with hand ventilation. Another is a visible inspection for signs of kinking of the ETT or circuit. Increasing the FiO_2_ to 100% will mitigate worsening desaturation during the investigation.

The “shark fin” appearance of the end tidal CO_2_ tracing is concerning for obstruction. Causes of obstruction include asthma leading to bronchospasm, chronic obstructive pulmonary disease,

histamine release from medications, mucous plugging of the ETT, mechanical obstruction of the ETT and or circuit, pulmonary aspiration, pulmonary edema, pulmonary embolism, and pneumothorax.^2^

[Play]

***Video Time 3:15 -What is the most likely diagnosis?***

[Stop]

The hypotension with tachycardia, combined with rash and wheezing, makes an anaphylaxis reaction rise to the top of the list.

[Play]

***Video Time 3:34 -What are the initial treatments for Anaphylaxis?***

[Stop]

Anaphylaxis is life-threatening with a rapid onset, therefore calling for additional help is prudent to treat this patient in a timely manner. Intravenous (IV) epinephrine is the key medication to stabilize mast cells and stop the cascade of cytokines, thus mitigating the vasodilation and vascular leak which lead to hypotension. The dose ranges widely between 10 to 100 mcg depending on the severity of symptoms. If cardiac arrest is suspected, then one should administer 1 mg and then follow Advanced Cardiac Life Support (ACLS). ^3^ Other initial important measures include treating the hypotension due to vasodilation with rapid fluid administration and decreasing the volatile anesthetic concentration. The alpha-1 vasoconstrictive activity of IV epinephrine will also mitigate the hypotension. Frequent cycling of the non-invasive blood pressure cuff can help direct treatment for the time being. Treat the desaturation by increasing the FiO_2_ to 100%. The wheezing is likely due to bronchospasm caused by anaphylaxis and albuterol should be administered to the lungs via the endotracheal tube.

[Play]

***Video Time 5:05 -Are there any other treatments you should give? What other access should be obtained?***

[Stop]

Additional important treatments including a histamine blocker such as diphenhydramine. A steroid such as hydrocortisone, should be administered to mitigate the progression of the bronchospasm and prevent late-phase anaphylaxis.^5^ To treat the gastrointestinal effects of anaphylaxis, administer an H_2_ blocker, such as famotidine.

Given the hemodynamic instability that can persist with anaphylaxis, placing an arterial line would be prudent. An arterial line would also help obtain arterial blood gases (ABG) to ensure that bronchospasm is no longer affecting gas exchange.

A central line is necessary for administering an epinephrine infusion for ongoing treatment of anaphylaxis. If additional large bore peripheral intravenous lines are challenging to obtain, a central line may be used for resuscitation purposes.

[Play]

***Video Time 5:30 -What is the disposition of this patient? What do you tell the surgeon?***

[Stop]

An anaphylaxis reaction can reoccur after initial treatments. Therefore, transferring the patient intubated and sedated to the Intensive Care Unit for further management is prudent. If possible, the case should be canceled and take place when the patient is hemodynamically stable.

[Play]

***Video Time* 5:55 *-What labs should we obtain?***

[Stop]

Tryptase is a protease released from mast cells. An elevated tryptase level supports the diagnosis of an anaphylaxis reaction. It peaks between 15 and 120 minutes from the onset of the reaction.^2,5^ Other labs to help monitor the treatment include an ABG, and basic metabolic panel to monitor electrolyte derangements.

[Play]

***Video Time 6:38 -Do you want to order any infusions?***

[Stop]

A bolus dose of epinephrine IV lasts only up to 15 minutes. Therefore, to continually treat the evolving anaphylaxis reaction, starting an epinephrine infusion is prudent.

[Play]

***6:54 Time to Debrief***

[Stop]

Perioperative anaphylaxis can present as hemodynamic instability that can lead to cardiac arrest, and/or airway obstruction with or without rash.^5^ The diagnosis is more challenging while under anesthesia as symptoms of malaise, pruritus, dizziness cannot be ascertained. ^5^ Additionally, visualizing and diagnosing a rash is more challenging as the patient can be covered by surgical drapes.^2^ Anaphylaxis can be easily mistaken for other causes of hemodynamic instability. ^5^

Most reactions are immediate, but delayed reactions can occur. The mechanism of anaphylaxis involves an immunoglobulin (Ig) E mediated reaction leading to release of mediators such as histamine from basophils and mast cells.^2,5^ In non-IgE-mediated reactions, formerly known as anaphylactoid reactions, the primary driver is direct mediator release from mast cells and basophils.^2^ The mediators lead to capillary permeability, vasodilation, bronchoconstriction, and hypotension. The incidence of anaphylaxis is 1 in 1,250 and 1 in 1 in 20,000 and the mortality ranges between 3 to 9%.^2,5^ Most common causes of intraoperative anaphylaxis are neuromuscular blocking agents (NMBAs), antibiotics, disinfectants and latex. ^2^ Patients who experience perioperative anaphylaxis should be evaluated by an allergist to determine the cause and prevent the anaphylaxis from reoccurring.

[Stop]

**What went well in the simulation video? What would you change?**

**What was the most impactful learning point gained from the learning activity?**

**What will you incorporate into your future practice?**

**References:**

1. Gerlach RM and Sweitzer BJ. Preoperative Evaluation and Medication. In: Pardo Jr, MC and Miller RD, eds. *Basics of Anesthesia*. 7^th^ ed. Elsevier 2017:189-212.
2. Volcheck, GE and Hepner DL. Identification and Management of Perioperative Anaphylaxis. 2019;7(7):2134-2142.
3. Krishna P, and Shimabukuro D. Cardiopulmonary Resuscitation. In: Pardo Jr, MC and Miller RD, eds. *Basics of Anesthesia*. 7^th^ ed. Elsevier 2017:788-802.
4. Frigas, E and Park, MA. Acute Urticaria and Angeioedema: diagnostic and treatment considerations. Am J Clin Dermatol. 2019;10(4):239-50.
5. Kannan, JA and Bernstein, JA. Perioperative Anaphylaxis Diagnosis, Evaluation, and Management. Immunology and Allergy Clinics of North America. 2015;35:321–334.
