# Supplementary material for: Development and Implementation of Video-Recorded Simulation Scenarios to Facilitate Case-Based Learning Discussions for Medical Students' Virtual Anesthesiology Clerkship
Source: MedEdPORTAL. 2023 Apr 4;19:11306. doi: 10.15766/mep_2374-8265.11306 (PMC10070881; doi:10.15766/mep_2374-8265.11306)
Supplement: Supplementary file 1 — Preoperative Evaluation - CBLD 1.pptxInhaled and Intravenous Anesthetics - CBLD 2.pptxAirway Management - CBLD 3.pptxScenario 1.mp4Scenario 2.mp4Scenario 3.mp4Scenario Debrief 1.docxScenario Debrief 2.docxScenario Debrief 3.docxClerkship Survey Questions.docxCBLD-Specific Survey Questions.docx [file mep_2374-8265.11306-s001.zip › H. Scenario Debrief 2.docx]

Appendix H

Anesthesiology Simulation Scenario No 2: Malignant Hyperthermia

Video and Debriefing Guide

Prebrief:

Simulations attempt to approximate a real-life operating room setting. However, limitations exist including the use of a mannequin, actors not carrying out the actions they say they are doing, and poor use of sterile technique. The goal of the simulation video is to give learners a glimpse into the OR setting and an opportunity to discuss key features of anesthetic management.

[Play]

An 18-year-old man with no significant past medical history was involved in a motorcycle accident.

*Video Time 00:16: What else do you want to know about the patient?*

[Stop Video]

When performing a preoperative anesthesia evaluation, it is important to obtain the patient’s past medical history and determine what type of procedure the patient is having, along with the reason the patient is having the procedure and any symptoms related to said operation/issue. In this case, although the patient is only 18 years old and his reason for needing surgery is trauma related to a motor vehicle accident, we would still like to ensure he does not have any other comorbid medical conditions. If he does, we would like to know what medications he is currently taking and any symptoms related to this condition. A focused review of symptoms is a helpful way to quickly gain important information. We would also want to know if the patient has ever had an operation under anesthesia and if so what type of anesthetic (i.e., general, monitored anesthesia care (MAC), regional) and how the patient tolerated the anesthetic. Even if the patient has no history of anesthesia reactions in the past or has never undergone anesthesia, it is important to assess whether anyone in his family has had any anesthetic reactions or concerns, as some problems are heritable. In addition to medications, it is important to ask about whether he has any allergies to any medications, tape, or latex. Finally, we would want to ascertain the patient’s nil per os (NPO) time by asking when he last had something to eat and/or drink.

“He is taken to the OR for emergency surgery.”

[Play]

Induction of anesthesia and rapid sequence intubation was uneventful.

Medications Given: Propofol, Fentanyl & Rocuronium

The patient is maintained on sevoflurane and the surgeon has already started.

*1:10 -What is your differential diagnosis?*

[Stop video]

What is concerning to you? The patient’s heart rate has increased to the 140s while the surgeon is operating. What could this be? Tachycardia during anesthesia can be associated with many changes in the procedure or the anesthetic. First, the patient may simply deeper plane of anesthesia. We would either increase the minimum alveolar concentration (MAC) of the volatile anesthetic agent or give a bolus of an intravenous hypnotic agent or analgesic. In this scenario both of those things were done. Second, sudden onset of tachycardia could also be due to hemorrhage due to the trauma, cardiopulmonary in nature secondary to arrhythmia, tamponade, pulmonary embolism, or ischemia, or a metabolic derangement, such as thyroid storm, malignant hyperthermia, pheochromocytoma, serotonin syndrome, sepsis, or neuroleptic malignant syndrome.^1^

[Play]

*01:59 -What should you do?*

[Stop video]

After deepening the anesthetic, giving some analgesic medications (fentanyl), and increasing fluids, the heart rate has decreased. However, your end-tidal CO2 is now climbing, your oxygen saturation has decreased, and your temperature has increased. At this point I would be very concerned that something significant and dangerous is happening and would ask the surgeon to stop operating and call for help.^2^

[Play]

*02:39 -What do you think is going on? What should you do to treat?*

*[Stop video]*

Given the tachycardia, hyperthermia, and significantly elevated carbon dioxide, I would be very concerned for malignant hyperthermia (MH). Once MH is suspected, as much help as possible should be called for and treatment should start immediately. Early diagnosis and treatment of MH can lead to a significant reduction in mortality and morbidity. The most important steps^3^ to mitigate the effects of MH for the patient are:

1. Stop the triggering agent (likely the volatile anesthetic in this case)
2. Remove the vaporizers from the machine
3. Hyperventilate the patient with 100% oxygen with an ambu bag. The anesthesia machine should be flushed, a new circuit placed, and charcoal filters attached them to the inspiratory and expiratory limbs of the circuit
4. Switch to a total intravenous anesthetic (TIVA) anesthetic if the patient is stable enough to tolerate anesthesia
5. Administer dantrolene (found in the MH cart)
6. Cool the patient

[Play]

*03:40 -What do you want to do about access?*

[Stop video]

The combination of hypercarbia, sympathetic stimulation, hyperkalemia, and acidosis is highly arrhythmogenic and cardiac arrest should be anticipated. In order to monitor and treat these complications of MH, an arterial line should be placed for hemodynamic monitoring as well as for arterial blood gas and electrolyte sampling. A central line should also be placed for the administration of vasopressors and inotropes, if needed. Obtaining a few large bore (18 gauge or greater) peripheral IVs is important as an MH reaction predisposes patients to acute kidney injury secondary to the effects of free myoglobin from rhabdomyolysis; hydration and diuresis will be important to prevent worsening injury. MH can also lead to disseminated intravascular coagulation (DIC) which would require the administration of blood products.^2^

[Play]

*04:28 -Should the surgery continue? What is the disposition of the patient?*

[Stop video]

MH is a life threatening condition and must be the primary concern. If the surgeon is at a place in the surgery where there is no acute bleeding and the patient is stable from an operative perspective, the surgery should be stopped so the patient can be adequately treated and resuscitated. If this were not the case, then the surgeon should work to get the patient to this point as quickly as possible. MH is very serious and the patient should be monitored in a critical care environment even if he appears to be improving after treatment and discontinuation of the offending agent.

[Play]

*05:36 Why is the urine pink?*

[Stop video]

Malignant hyperthermia puts the body into a hypermetabolic state which leads to rhabdomyolysis, the breakdown of muscle into the blood. Myoglobinuria results when myoglobin, one of the products released by damaged muscle, is excreted in the urine, giving it a pinkish or brown color.

Time to Debrief

[Stop video]

Additional Discussion Information:

Malignant Hyperthermia is an autosomal dominant genetic disorder that caused by abnormal skeletal muscle receptors, most commonly the ryanodine receptor (RYR1).^3^ Susceptible individuals can go into a state of metabolic hyperactivity when exposed to triggering agents: volatile anesthetics and/or succinylcholine. This hypermetabolic crisis leads to muscle breakdown which then causes progressive hypercapnia, an increase in heart rate, hyperthermia, muscle rigidity, rhabdomyolysis, hyperkalemia, metabolic acidosis, hypoxia, and DIC.

Anytime malignant hyperthermia is even remotely suspected, the Malignant Hyperthermia Association of the United States (MHAUS) hotline should be called for guidance or if the diagnosis is unclear. Every operating room area has access to a Malignant Hyperthermia cart containing dantrolene and should also be called for at the time of concern.^4^

What went well in the simulation video? What would you do differently?

Points to bring up include:

A realistic workflow when treating MH involves calling for help and involves numerous OR staff performing multiple tasks and procedures to treat the patient.

In the video, the actors apply ice packs to the patient’s abdomen and violate sterile technique. Discuss appropriate timing (core temperature > 39C) and placement of the ice packs to the neck, groin, and armpits.

What was the most impactful learning point gained from the learning activity?

What will you incorporate into your future practice?

References:

1. Gupta PK, Hopkins PM. Diagnosis and Management of Malignant Hyperthermia. BJA Education. 2017;17(7): 249–254.
2. Glahn KP, Ellis FR, Halsall PJ, et al. Recognizing and managing a malignant hyperthermia crisis: guidelines from the European Malignant Hyperthermia Group. Br J Anaesth 2010;105: 417–20.
3. [Denborough M. Malignant hyperthermia. Lancet 1998; 352(9134):1131-1136.](https://www.uptodate.com/contents/malignant-hyperthermia-diagnosis-and-management-of-acute-crisis/abstract/2)
4. Healthcare Professionals. Malignant Hyperthermia Association of the United States. Accessed 6/4/22. https://www.mhaus.org/healthcare-professionals./
