# Supplementary material for: Development and Implementation of Video-Recorded Simulation Scenarios to Facilitate Case-Based Learning Discussions for Medical Students' Virtual Anesthesiology Clerkship
Source: MedEdPORTAL. 2023 Apr 4;19:11306. doi: 10.15766/mep_2374-8265.11306 (PMC10070881; doi:10.15766/mep_2374-8265.11306)
Supplement: Supplementary file 1 — Preoperative Evaluation - CBLD 1.pptxInhaled and Intravenous Anesthetics - CBLD 2.pptxAirway Management - CBLD 3.pptxScenario 1.mp4Scenario 2.mp4Scenario 3.mp4Scenario Debrief 1.docxScenario Debrief 2.docxScenario Debrief 3.docxClerkship Survey Questions.docxCBLD-Specific Survey Questions.docx [file mep_2374-8265.11306-s001.zip › I. Scenario Debrief 3.docx]

**Appendix I**

**Anesthesiology Simulation Scenario No 3: Difficult Airway**

**Video and Debriefing Guide**

**Prebrief:**

Simulations attempt to approximate a real-life operating room setting. However, limitations exist including the use of a mannequin, actors not carrying out the actions they say they are doing, and poor use of sterile technique. The goal of the simulation video is to give learners a glimpse into the OR setting and discuss key features of anesthetic management.

Be aware that there should not be a capnography waveform during attempts to intubate the patient. There is a delay in programming of the monitors and the actions of the actors within the simulation video. The learners should focus on the enlarged monitor on the right, rather than the monitor in the video with the actors.

[Play]

**This is a 24-year-old woman who presented to the ED with intermittent abdominal pain and N/V. She is suspected to have appendicitis and presents for laparoscopic appendectomy.**

***Video Time 0:19 - What else do you want to know about the patient?***

[Stop]

According to the American Society of Anesthesiologists’ (ASA) Practice Advisory for Preanesthesia Evaluation, the anesthesiologist should conduct a review of the patient’s relevant medical records, an interview, and a physical examination. Based on the patient’s history and the findings from the initial assessment, the anesthesiologist may wish to order additional labs, imaging, or consults. The physical examination should include an assessment of the heart, lungs, and the airway.^1^

In addition, the anesthesiologist will assess the patient’s aspiration risk, including the last oral intake, review medications and allergies, and ask the patient about use of alcohol, tobacco, and/or illicit drugs.^2^

[Play]

**The patient is in the OR ready for induction, EKG, SpO_2_, NIBP attached, with a PIV in place.**

***Video Time 0:56 - Where do you want end tidal oxygen (EtO_2_) concentration to be prior to induction of anesthesia?***

[Stop]

A reasonable goal for the end-tidal oxygen concentration after adequate preoxygenation would be in the mid 80s.^3^ This can be achieved by applying the circuit mask to the patient’s face with a tight seal and delivering an FiO_2_ of 100%. Various techniques exist for achieving optimal preoxygenation. In this scenario, the patient can be asked to breathe normally (tidal volume breathing) or to take 8 deep breaths (inspiratory capacity breaths).^4^

[Play]

***Video Time 1:15 - What type of induction should you do for this patient? What induction agents would you use?***

[Stop]

In this patient undergoing emergency abdominal surgery, a rapid-sequence induction (RSI) would be indicated to minimize the risk of aspiration. When anesthesia is induced, the patient is no longer able to protect their airway with protective mechanisms such as coughing. Placement of an endotracheal tube with an inflated cuff can protect the lungs from contamination even if there is emesis. A rapid sequence induction minimizes this vulnerable time between induction of anesthesia and placement of the endotracheal tube by omitting the mask ventilation step after induction of anesthesia. Cricoid pressure is often performed during the induction and until confirmation that the ETT is in the trachea.

For an RSI, it would be appropriate to use a hypnotic agent, such as propofol, followed immediately by a rapid-acting neuromuscular blocking agent such as succinylcholine (1.0 - 1.5 mg/kg) or a higher dose of rocuronium (1.0 - 1.2 mg/kg).^2^

[Play]

***Video Time 2:20 - What are options for the next intubation attempt?***

[Stop]

If cricoid pressure is still being held, it should be released. When performed as part of an RSI and intubation, cricoid pressure (also known as Sellick’s maneuver), is intended to prevent passive reflux from the stomach into the oropharynx. If it is impeding the laryngoscopic view, however, it should be released.

Options for the next intubation attempt include changing the person performing the intubation to the most experienced anesthesiologist, use of a different type of blade, optimizing the patient’s position, use of a gum elastic bougie, videolaryngoscopy, fiberoptic bronchoscopy or video endoscopy, or intubation via an intubating supraglottic airway (SGA), such as an intubating laryngeal mask airway (LMA).

[Play]

***Video Time 2:45 - You still cannot intubate, and oxygen saturation is decreasing. What should you do next?***

[Stop]

It would be appropriate to attempt mask ventilation since the patient’s oxygen saturation has begun to fall. To help minimize insufflation of the stomach and the risk of aspiration, one may attempt to keep positive pressures below 20 mmHg. However, if the patient is difficult to mask and requires higher positive pressure to appropriately ventilate her, then higher pressures should be applied.

[Play]

***Video Time 3:24 - You have now found yourself in a “Can’t Intubate, Can’t Ventilate” situation. According to the Difficult Airway Algorithm, what should you do next?***

[Stop]

According to the Difficult Airway Algorithm, this would be an appropriate time to attempt placement of an LMA. If successful, you have returned the patient to a Non-Emergency Pathway.^5^

[Play]

***Video Time 4:36 - You are now able to ventilate the patient, but it’s an emergency surgery and you need the patient to be intubated. What is your plan to help you secure the airway?***

[Stop]

One option would be to remove the LMA after ensuring adequate oxygenation and using a videolaryngoscope to intubate the patient. If this approach is taken, make sure you have the LMA available in case the intubation via videolaryngoscopy is unsuccessful. Alternatively, if the LMA in place is an intubating LMA, you may elect to bring a fiberoptic bronchoscope (or video endoscope) into the room and intubate via the LMA.

[Play]

***Video Time 6:01 - What grade view of the airway is this? What are you looking for when you intubate?***

[Stop]

According to the Cormack-Lehane grading system, this is a Grade 1 view, in which the entire laryngeal aperture is visible. Ideally, one would have either a Grade 1 or 2 view when visualizing the glottis with either direct or video- laryngoscopy.

[Stop]

***Time to Debrief***

**What went well in the video simulation? What would you change?**

**What was the most impactful learning point gained from the learning activity?**

**What will you incorporate into your future practice?**

**In-Video Image citations:**

At Video Time 2:09:

"Cormack-Lehane 1" by OpenAirway, retrieved from: <https://openairway.org/cormack-lehane-grading-examples/> on May 22, 2022. Creative Commons License associated: <https://creativecommons.org/licenses/by/2.0/>.

At Video Time 5:55:

"Cormack-Lehane 4" by OpenAirway, retrieved from: <https://openairway.org/cormack-lehane-grading-examples/> on May 22, 2022. Creative Commons License associated: <https://creativecommons.org/licenses/by/2.0/>.

**References:**

1. Apfelbaum J, Connis R, Nickinovich D, et al. Practice advisory for preanesthesia evaluation: an updated report by the American Society of Anesthesiologists Task Force on Preanesthesia Evaluation. Anesthesiology: the journal of the American Society of Anesthesiologists, Inc. 20123;116(3):522-538. doi:10.1097/ALN.0b013e31823c1067
2. Gerlach, RM, Sweitzer BJ. Chapter 13: Preoperative Evaluation and Medication. In: Pardo MC, Miller RD, eds. *Basics of Anesthesia.* 7^th^ ed. Elsevier; 2018:189-212.
3. Tulchinsky, Amir MD An End-Tidal Goal of 90% O2 May Be Unattainable During Preoxygenation, Anesthesia & Analgesia: July 2021 - Volume 133 - Issue 1 - p e11-e12

doi: 10.1213/ANE.0000000000005572

1. Nimmagadda U, Salem MR, Crystal GJ. Preoxygenation: Physiologic Basis, Benefits, and Potential Risks. Anesthesia & analgesia. 124(2):507-517. doi:10.1213/ANE.0000000000001589
2. Apfelbaum JL, Hagberg CA, Connis RT, et al. 2022 American Society of Anesthesiologists Practice Guidelines for Management of the Difficult Airway. *Anesthesiology*. 2022;136(1):31-81. doi:10.1097/ALN.0000000000004002
