# Supplementary material for: Development and Implementation of Video-Recorded Simulation Scenarios to Facilitate Case-Based Learning Discussions for Medical Students' Virtual Anesthesiology Clerkship
Source: MedEdPORTAL. 2023 Apr 4;19:11306. doi: 10.15766/mep_2374-8265.11306 (PMC10070881; doi:10.15766/mep_2374-8265.11306)
Supplement: Supplementary file 1 — Preoperative Evaluation - CBLD 1.pptxInhaled and Intravenous Anesthetics - CBLD 2.pptxAirway Management - CBLD 3.pptxScenario 1.mp4Scenario 2.mp4Scenario 3.mp4Scenario Debrief 1.docxScenario Debrief 2.docxScenario Debrief 3.docxClerkship Survey Questions.docxCBLD-Specific Survey Questions.docx [file mep_2374-8265.11306-s001.zip › J. Clerkship Survey Questions.docx]

**Appendix J: Clerkship Survey Questions**

| General Clerkship Survey Questions | Below Expectations | Meet Expectations | Exceeds Expectations |
| --- | --- | --- | --- |
| Learning Environment: Faculty, Residents and Staff demonstrate professional conduct and respect for students. |  |  |  |
| Organization of course: Including logistics; timely distribution of materials; accessibility of course director/site coordinator; clarity of communication about objectives, content roles, responsibilities, with structured time to attend student and departmental activities. |  |  |  |
| Educational Value: Including teaching/learning environment; clinical experiences and opportunities; assessments related to clerkship objectives. |  |  |  |
| Teaching: By attending physicians, residents, fellows, other health professionals and staff. |  |  |  |
| Evaluation and Feedback: Constructiveness and timeliness of feedback received. |  |  |  |

| Virtual Clerkship Survey Questions | Strongly Disagree | Disagree | Neutral | Agree | Strongly Agree |
| --- | --- | --- | --- | --- | --- |
| The objectives of the virtual section were made clear to me. |  |  |  |  |  |
| The assignments planned for the virtual learning sessions facilitated my learning of the virtual section material. |  |  |  |  |  |
| The resources provided for the class were useful in learning the material: (i.e. recommended readings, course packet, Canvas site, etc.). |  |  |  |  |  |
| Overall, I have acquired an understanding of the stated virtual section objectives. |  |  |  |  |  |
| Overall, I have found this virtual section to be valuable. |  |  |  |  |  |
