# Supplementary material for: Development and Implementation of Video-Recorded Simulation Scenarios to Facilitate Case-Based Learning Discussions for Medical Students' Virtual Anesthesiology Clerkship
Source: MedEdPORTAL. 2023 Apr 4;19:11306. doi: 10.15766/mep_2374-8265.11306 (PMC10070881; doi:10.15766/mep_2374-8265.11306)
Supplement: Supplementary file 1 — Preoperative Evaluation - CBLD 1.pptxInhaled and Intravenous Anesthetics - CBLD 2.pptxAirway Management - CBLD 3.pptxScenario 1.mp4Scenario 2.mp4Scenario 3.mp4Scenario Debrief 1.docxScenario Debrief 2.docxScenario Debrief 3.docxClerkship Survey Questions.docxCBLD-Specific Survey Questions.docx [file mep_2374-8265.11306-s001.zip › K. CBLD-Specific Survey Questions.docx]

**Appendix L: CBLD-Specific Survey Questions**

| Survey Questions | Disagree with this Statement | Neutral | Agree with this Statement |
| --- | --- | --- | --- |
| The faculty-led CBLD exercises are informative. |  |  |  |
| The simulation-based CBLDs are an effective learning modality. |  |  |  |
| The simulation-based CBLDs are more effective than traditional learning modalities. |  |  |  |
